# Two Lineages of *Pseudomonas aeruginosa* Filamentous Phages: Structural Uniformity over Integration Preferences

**DOI:** 10.1093/gbe/evaa146

**Published:** 2020-07-13

**Authors:** Krzysztof Fiedoruk, Magdalena Zakrzewska, Tamara Daniluk, Ewelina Piktel, Sylwia Chmielewska, Robert Bucki

**Affiliations:** Department of Medical Microbiology and Nanobiomedical Engineering, Medical University of Białystok, Poland

**Keywords:** *Pseudomonas aeruginosa*, filamentous bacteriophages, Pf phages, integrases, insertion sites, evolutionary lineages

## Abstract

*Pseudomonas aeruginosa* filamentous (Pf) bacteriophages are important factors contributing to the pathogenicity of this opportunistic bacterium, including biofilm formation and suppression of bacterial phagocytosis by macrophages. In addition, the capacity of Pf phages to form liquid crystal structures and their high negative charge density makes them potent sequesters of cationic antibacterial agents, such as aminoglycoside antibiotics or host antimicrobial peptides. Therefore, Pf phages have been proposed as a potential biomarker for risk of antibiotic resistance development. The majority of studies describing biological functions of Pf viruses have been performed with only three of them: Pf1, Pf4, and Pf5. However, our analysis revealed that Pf phages exist as two evolutionary lineages (I and II), characterized by substantially different structural/morphogenesis properties, despite sharing the same integration sites in the host chromosomes. All aforementioned model Pf phages are members of the lineage I. Hence, it is reasonable to speculate that their interactions with *P. aeruginosa* and impact on its pathogenicity may be not completely extrapolated to the lineage II members. Furthermore, in order to organize the present numerical nomenclature of Pf phages, we propose a more informative approach based on the insertion sites, that is, Pf-tRNA-Gly, -Met, -Sec, -tmRNA, and -DR (direct repeats), which are fully compatible with one of five types of tyrosine integrases/recombinases XerC/D carried by these viruses. Finally, we discuss possible evolutionary mechanisms behind this division and consequences from the perspective of virus–virus, virus–bacterium, and virus–human interactions.

## Introduction

*Pseudomonas aeruginosa* filamentous (Pf) bacteriophages are relatively long (∼3,700 nm in length and 6 nm in diameter) with respect to the size of their circular ssDNA genomes (7.3–13 kb) (Mai-Prochnow et al. 2015; Hay and Lithgow 2019). Likewise, other filamentous phages present in Gram-negative and Gram-positive bacteria, such as biotechnologically relevant *Escherichia coli* M13, Ff and Fd, or the cholera toxin-carrying phage CTXϕ from *Vibrio cholerae*, are classified into the Inoviridae family and *Inovirus* genus. To date, 56 filamentous phages have been described, but a recent genomic study increased their number to 5,964 distinct species, with a suggestion to reclassify this family to an order provisionally divided into six candidate families and 212 candidate subfamilies (Roux et al. 2019).

So far, eight *P. aeruginosa* Pf phages, numbered from Pf1 to Pf7, as well as Pf-LESB58 (the Liverpool Epidemic Strain B58) have been identified (King et al. 2011; Mai-Prochnow et al. 2015). However, only Pf1 (the natural host is unknown, but PAK strain is susceptible), Pf2 (from P28 strain), and Pf3 (the natural host is undefined) have species status, whereas the remaining have not yet been approved as species (King et al. 2011). The Pf2 phage, characterized in 1968, is no longer described in the literature, and the Pf3 is genetically entirely distinct from the other Pf phages (Mai-Prochnow et al. 2015). Therefore, only the Pf1, Pf4 (from PAO1 strain), and Pf5 (from PA14 strain) are commonly used as model viruses in research studies, and the genome annotation nomenclature of Pf4 prophage (genes from *PA0715* to *PA0729*) is widely accepted in the literature (Mai-Prochnow et al. 2015).

It is noteworthy that, in contrast to other bacteriophages, the filamentous phages generally do not kill their hosts during replication. Instead, they are characterized by the ability to establish persistent or chronic infections, being continuously released from the bacterial cell by extrusion without its lysis. Hence, their lifestyle resembles a rather symbiotic relationship than parasitism, and the well-known terms “lytic” and “lysogenic” are not fully suitable (King et al. 2011; Hay and Lithgow 2019). Furthermore, depending on a life cycle, filamentous phages can be divided into 1) nonintegrative, which exist and replicate only as extrachromosomal episomes (e.g., Pf1 or Pf3) and 2) integrative, that is, having an additional prophage state (“temperate,” “lysogenic,” or “lysogen”), for example, Pf4–Pf7. The latter process is mediated by endogenously encoded (e.g., Pf phages) or host encoded (e.g., CTXϕ) integrases. Nevertheless, under special circumstances, for example, in mature biofilms, conversion to a so-called superinfective (SI) state may occur (e.g., in Pf4 and Pf5), that is, able to reinfect and subsequently kill the host in spite of carrying its prophage variant. In other words, a Pf-SI variant can bypass the phage immunity ensured by the Pf lysogen.

In the genetic organization of filamentous phages at least three modules can be distinguished, specifically 1) replication, 2) structural, and 3) assembly/secretion modules, based on *E. coli* Ff phage as the reference (Mai-Prochnow et al. 2015). The first module covers genes encoding proteins responsible for binding ssDNA (G5P and G10P) and its rolling-circle replication (G2P). In Pf phages G2P is absent, because the replication is performed by the host UvrD helicase (one of the enzymes involved in DNA repair) and its initiation is mediated by an additional protein–replication initiation protein (PA0727 protein in Pf4) as in CTXϕ phage (Martinez and Campos-Gomez 2016). The genes encoding capsid proteins, such as major (G8P; 2,800 copies per capsid) and minor capsid proteins (G3P, G6P, G7P, and G9P; five copies of each per capsid) are localized in the structural module. It should be emphasized that G3P is responsible for recognition of the host receptors, for example, usually type IV pili. Furthermore, G8P subunits show α-helical structure overlapping each other forming Class I (e.g., Ff phage) or Class II symmetry (e.g., Pf phages; King et al. 2011). The last module involves proteins responsible for morphogenesis and secretion of viral particles (G1P, G4P, and G11P), where G4P (secretin) forms a channel in the outer membrane allowing the release of new virions, although some filamentous phages exploit host secretins (e.g., type II or IV secretion systems) to that end. The latter mechanism may also occur in Pf phages, as there is no homolog of G4P in Pf4, similarly not all counterparts of capsid proteins have been identified (Mai-Prochnow et al. 2015). Interestingly, G1P in CTXϕ phage possesses the zonula occludens toxin-specific domain (Zot) that mediates binding to the tight gap junctions in the intestine. Hence, it is considered as a factor contributing to the virulence of *V. cholerae* strains, beside of the cholera toxins genes located in the accessory part of the CTXϕ genome (Fasano et al. 1995). Similarly, other filamentous phages may carry virulence genes associated with the pathogenesis of different diseases including cystic fibrosis (CF), acute gastroenteritis, neonatal meningitis, gonorrhea, and plague (Mai-Prochnow et al. 2015) and thus may be treated as factors improving bacterial adaptation capabilities.

For example, Sweere et al. have recently used a murine wound model to show that Pf phages inhibit antibacterial defense mechanisms, such as phagocytosis and tumor necrosis factor (TNF) production, but instead as the result of their endocytosis by leukocytes induce antiviral immunity and type I interferon secretion through stimulation of Toll-like receptor 3 (Sweere et al. 2019). Accordingly, Pf phages have been characterized as immunomodulatory agents, which may impact production of several cytokines and the polarization of macrophages in infection sites (Secor et al. 2020).

From a physicochemical point of view, Pf phages represent long negatively charged macromolecules that may directly contribute to biofilm formation via interaction with host and bacterial biopolymers (mucin, actin, DNA, and glycosaminoglycans) to assemble into highly structured liquid crystals that enhance biofilm adhesion and tolerance to desiccation and cationic antibiotics (Secor et al. 2015). We recently showed that Pf1, like other natural polyelectrolytes (DNA, F-actin, neurofilaments, and vimentin), significantly increases biofilm mass formation not only in *P. aeruginosa* Xen5 but also in various bacteria and the fungus *Candida albicans*, as well as enhancing swarming motility in the former species (Bucki et al. 2015). Moreover, Pf phages may competitively affect growth of fungal pathogens, in particular *Aspergillus* and *Candida*, by iron sequestration (Penner et al. 2016; Nazik et al. 2017).

Furthermore, pathogenicity may be associated with activation of a Pf prophage state, followed by an extensive production of new virions, in particular in the context of a biofilm life cycle. For instance, Pf4 genes are strongly upregulated in *P. aeruginosa* biofilm to produce phages at a 100–1,000-fold greater rate than in planktonic culture (Whiteley et al. 2001). Pf4 upregulation is correlated with reduced oxygen levels and might be an important adaptive mechanism in lung colonization or infection in patients with CF (Platt et al. 2008), whose sputum samples contain ∼10^7^ Pf phage/ml (Secor et al. 2015). Indeed, in a murine pneumonia model, infection caused by *P. aeruginosa* PAO1 mutants lacking Pf4 showed attenuated virulence resulting in a survival rate significantly longer compared with the wild PAO1 strain (Rice et al. 2009). Specifically, this phenomenon seems to be linked with Pf4 conversion into the SI form followed by bacterial lysis and subsequent dispersal in mature biofilms as well as formation of small colony variants (SCVs) (Webb et al. 2003, 2004; Sauer et al. 2004; Rice et al. 2009). The SCVs are characterized by a slow growth on agar plates, hyperpiliated and autoaggregative phenotypes along with resistance against a broad spectrum of antipseudomonal agents, which altogether enhance biofilm formation capabilities of *P. aeruginosa* (Haussler 2004).

On the contrary, according to Secor et al. (2017), *P. aeruginosa* PAO1 producing Pf4 phage at levels comparable with those achieved in biofilms, that is, lower that those examined in vitro, is less invasive and less inflammatory as well as more resistant to phagocytosis by macrophages, suggesting its role as an immune evasion factor that helps *P. aeruginosa* in the establishment of chronic infections. Therefore, Pf phages may function as agents that prevent bacterial dissemination by sequestering bacteria within the lungs, possibly as a consequence of the loss or inactivation of type IV pili and in turn impaired twitching motility.

Pf phages appear to be important factors in various stages of the biofilm life cycle, by regulating its development and structural integrity, as was recently stressed by the study of Lee et al. (2018). The authors showed that a substrate-binding protein DppA1, involved in peptide utilization via the DppBCDF ABC transporter, increases *P. aeruginosa* PA14 biofilm formation 68-fold by direct or indirect repression of Pf5 prophage genes, which in turn prevents cell lysis mediated by the Pf5-SI state. Accordingly, the inactivation of the *dppA1* gene resulted in a 600-fold increase in Pf5 excision and a million-fold increase in phage production.

The relation between Pf phages and virulence of *P. aeruginosa* seems to be indirectly supported by their common prevalence in clinical *P. aeruginosa* isolates. For example, Knezevic et al. detected Pf prophages in 56% of 241 examined *P. aeruginosa* clinical isolates (Knezevic et al. 2015). Likewise, Pf prophages were identified in 36.2% and 52.1% of *P. aeruginosa* strains isolated from two independent (Danish and Stanford) cohorts of patients with CF (Burgener et al. 2019) characterized also by higher Pf phage concentrations in sputum and resistance to antipseudomonal antibiotics (amikacin, meropenem, and aztreonam). Thus, Pf phages were proposed as a potential biomarker for risk of antibiotic resistance development and a potential therapeutic target for treatment of chronic *P. aeruginosa* infections in CF (Burgener et al. 2019).

However, several aspects of Pf phage biology and genetics, as well as mechanisms responsible for their interactions with bacterial hosts, in particular those linked with *P. aeruginosa* pathogenicity are largely unknown. To address these issues, we performed comprehensive analysis of Pf (pro)phages based on a bioinformatic approach, in order to recognize their prevalence, diversity, and distribution among various *P. aeruginosa* strains.

## Materials and Methods

In total, 190 *P. aeruginosa* complete chromosomal sequences deposited in the GenBank were screened for prophages with the Phaster tool (Arndt et al. 2016), given the fact of Pf prophages variation in the same *P. aeruginosa* strains (Klockgether et al. 2010) genomes from repeatable strains intentionally were not excluded. Phaster’s hits for the PHAGE_Pseudo_Pf1_NC_001331, regardless of their completeness category, that is, intact, incomplete, and questionable, according to the software nomenclature, were included for further analysis. In the next step, chromosomal locations with Pf prophage loci were manually inspected for the integration sites (*attB* and *attP*), including previously recognized sites such as 1) tRNA-Gly (*attB*) and its 27-bp fragment (*attP*) and 2) two 10-bp direct repeats (DR) of -TTTGTGCGTA- sequence in Pf4 and Pf5 genomes, respectively (Seol-Hee et al. 2003), as well as tRNA-Met (*attB*) and its 46-bp fragment (*attP*) in the Pf6 genome (Klockgether et al. 2010). Subsequently, the chromosomal loci containing Pf prophages, that is, delimited by *attB* and *attP* sites were annotated with Prokka v1.3.13 software (Seemann 2014) using protein sequences (and nomenclature) from Pf4 (AE004091.2) as the reference.

Phylogenetic analysis of the Pf (pro)phages was performed with the MAFFT online service for multiple sequence alignment (Katoh et al. 2019), and differences between major types of Pf prophages were visualized using Easyfig v2.1 (Sullivan et al. 2011) and BRIG v0.95 tools (Alikhan et al. 2011). Furthermore, BLAST2GO software was used for functional annotation and analysis of accessory genes present in the Pf prophages (Conesa and Gotz 2008) that were also screened for the presence of virulence, antimicrobial, biocide, and metal resistance determinants based on VFDB (Chen et al. 2016), ResFinder (Zankari et al. 2012), ARG-ANNOT (Gupta et al. 2014), CARD (Jia et al. 2017), NCBI AMRFinderPlus (Feldgarden et al. 2019), and MEGARES (Doster et al. 2020) databases and *Abricate script* (https://github.com/tseemann/abricate; last accessed January 2020). Finally, CRISPR spacer sequences (*n* = 221,397) downloaded from CRISPR-Cas++ server (https://crisprcas.i2bc.paris-saclay.fr; last accessed March 2020) were used to analyze their prevalence in the Pf (pro)phages.

Structure prediction/3D modeling of proteins was determined with the SWISS-MODEL server (https://swissmodel.expasy.org; last accessed January 2020), and TM-score (https://zhanglab.ccmb.med.umich.edu/TM-score/; last accessed January 2020) was applied afterward for their structural comparison.

Sequence types (STs) of the *P. aeruginosa* strains were ascribed using *mlst script* (https://github.com/tseemann/mlst; last accessed February 2020).

## Results

### *Pseudomonas aeruginosa* Strains and Pf Prophages

Overall, *P. aeruginosa* strains in this study (*n* = 190) were represented by 91 STs, and Pf prophages (*n* = 184) were identified in 126 (66.3%) of the strains belonging to 61 various STs. In general, Pf prophage-negative strains pertained to distinct STs compared with the Pf-positive ones, except ST111, ST235, ST253, and ST316 (supplementary table S1, Supplementary Material online). The number of Pf prophages per chromosome ranged from one (*n* = 73) through two (*n* = 48) and three (*n* = 5). Although, in single Pf prophages, some core genes (supplementary table S1, Supplementary Material online) were absent as the result of insertion mobile genetic elements, all but two (severely incomplete), that is, 182 prophages, were included in further analyses (supplementary table S1, Supplementary Material online). According to the Phaster results, only 106 Pf prophages were intact, and 63 and 6 were classified as questionable or incomplete, respectively. In some cases, even for strains with the same ST and virtually identical Pf prophages, their classification was different, and for seven strains, detection of prophages was not possible (supplementary table S1, Supplementary Material online).

### Integration Sites of Pf Prophages

Overall, in addition to the three known insertion sites, that is, within tRNA-Gly or tRNA-Met and 10-bp DR, two more were identified, where tRNA-Sec (*PA4802.1* in PAO1) and tmRNA (*PA0826.2* in PAO1) genes serve as *attB*, and their 27-bp (-GGGTTCGACTCCCACTGCCTTCCGCCA-) and 28-bp fragments as *attP* (-GGGGGTTCAAATCCCCCCGGCTCCACCA-) sites, respectively. Pf prophages inserted within tRNA-Gly (*n* = 74) and tRNA-Met (*n* = 65) predominated, followed by those utilizing DR (*n* = 21), tRNA-Sec (*n* = 15), and tmRNA (*n* = 7).

**Fig. 1.**
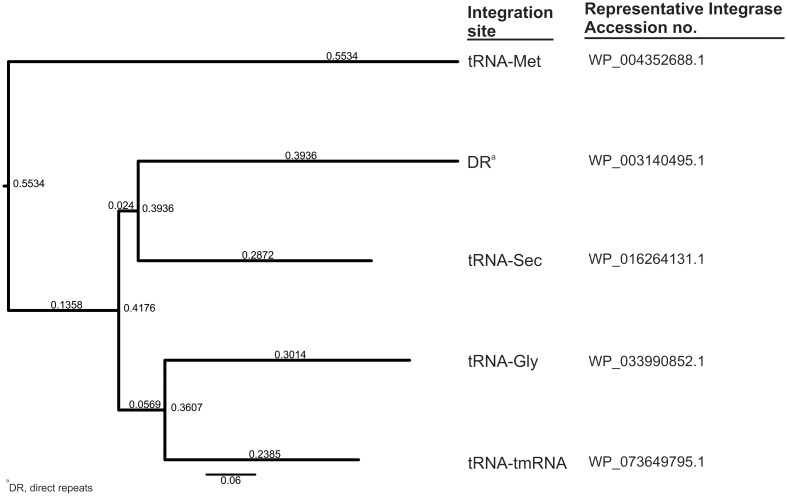
Neighbor-joining phylogenetic tree of five types of integrases (Int) present in the Pf (pro)phages. MAFFT online service was used to align protein sequences (MAFFT L-INS-I strategy) and create to dendrogram (neighbor-joining method with a bootstrap value of 500 for the conserved sites—316 amino acids) (Katoh et al. 2019).

**Fig. 2.**
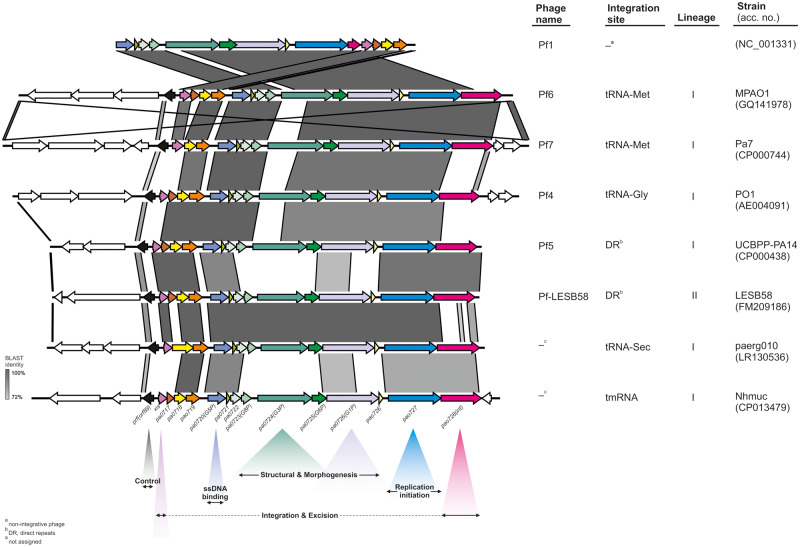
Comparison of five types of the Pf (pro)phages classified according to the integration site. The colored and white arrows represent core and accessory genes, respectively.

Accordingly, we observed a complete correlation between these five integration sites and the types of integrases (Int; PA0728 in Pf4) carried by the Pf prophages. In detail, integrases belong to the XerC superfamily (cl28330) and possess the same set of conserved domains (int—PHA02601, INT_Rci_Hp1_C—cd00796, recomb_XerC—TIGR02224, Phage_integrase—pfam00589, and XerD—COG4974) but share 40–62% identity of amino acid sequence (fig. 1). As the presence of a particular type of Int only partially overlaps with the current Pf phages nomenclature, that is, from Pf1 to Pf7, we introduced the following designations: 1) Pf-tRNA-Gly, 2) Pf-tRNA-Met, 3) Pf-tRNA-Sec, 4) Pf-tmRNA, and 5) Pf-DR, in order to underline this distinction between the Pf (pro)phages (fig. 2).

**Fig. 3.**
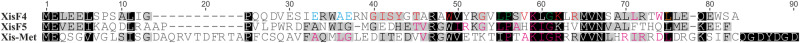
Comparison of amino acids sequences of the excisionases (Xis) present in the Pf prophages. XisF4 (present in 100%, 71%, and 15% of Pf-tRNA-Gly, Pf-tmRNA, and Pf-tRNA-Sec prophages, respectively), XisF5 (present in 100%, 85%, and 29% of Pf-tRNA-DR, Pf-tRNA-Sec, and Pf-tmRNA prophages, respectively) and Xis-Met (present in 100% of Pf-tRNA-Met prophages) are represented by the following protein sequences: WP_019372529.1, WP_022581007.1, and WP_025324668.1, respectively. Amino acids characteristic for COG2452 (COG2452), excise (TIGR01764), and HTH_17 (pfam12728) conserved domains are labeled by red, blue, and magenta colors, respectively; whereas green and orange colors indicate amino acids shared by all three domains or only the excise and HTH_17 ones.

At least partially, this division is supported by the distribution of excisionase (Xis) variants among the Pf prophages. Excisionases have been recently recognized in Pf4 (Pf-tRNA-Gly) and Pf5 (Pf-tRNA-Met) phages and designated as XisF4 and XisF5, respectively (Li et al. 2019). Briefly, XisF4 is specific for the Pf-tRNA-Gly group (100% of prophages) as well as 71.4% (5/7) and 15.4% (2/15) of prophages from the Pf-tmRNA and the Pf-tRNA-Sec group, respectively. XisF5 occurs in the Pf-DR group and the remaining Pf-tmRNA and Pf-tRNA-Sec prophages. Furthermore, we noted that a third variant of excisionase (denoted here as Xis-Met) is unique to the Pf-tRNA-Met prophages. In general, these three excisionase types show 19–34% identity at the amino acid sequence level and possess helix-turn-helix domains (HTH_17, HTH_MerR-SF superfamily; pfam12728) (fig. 3). XisF4 is characterized by two additional conserved domains, namely COG2452 and excise (TIGR01764).

**Fig. 4.**
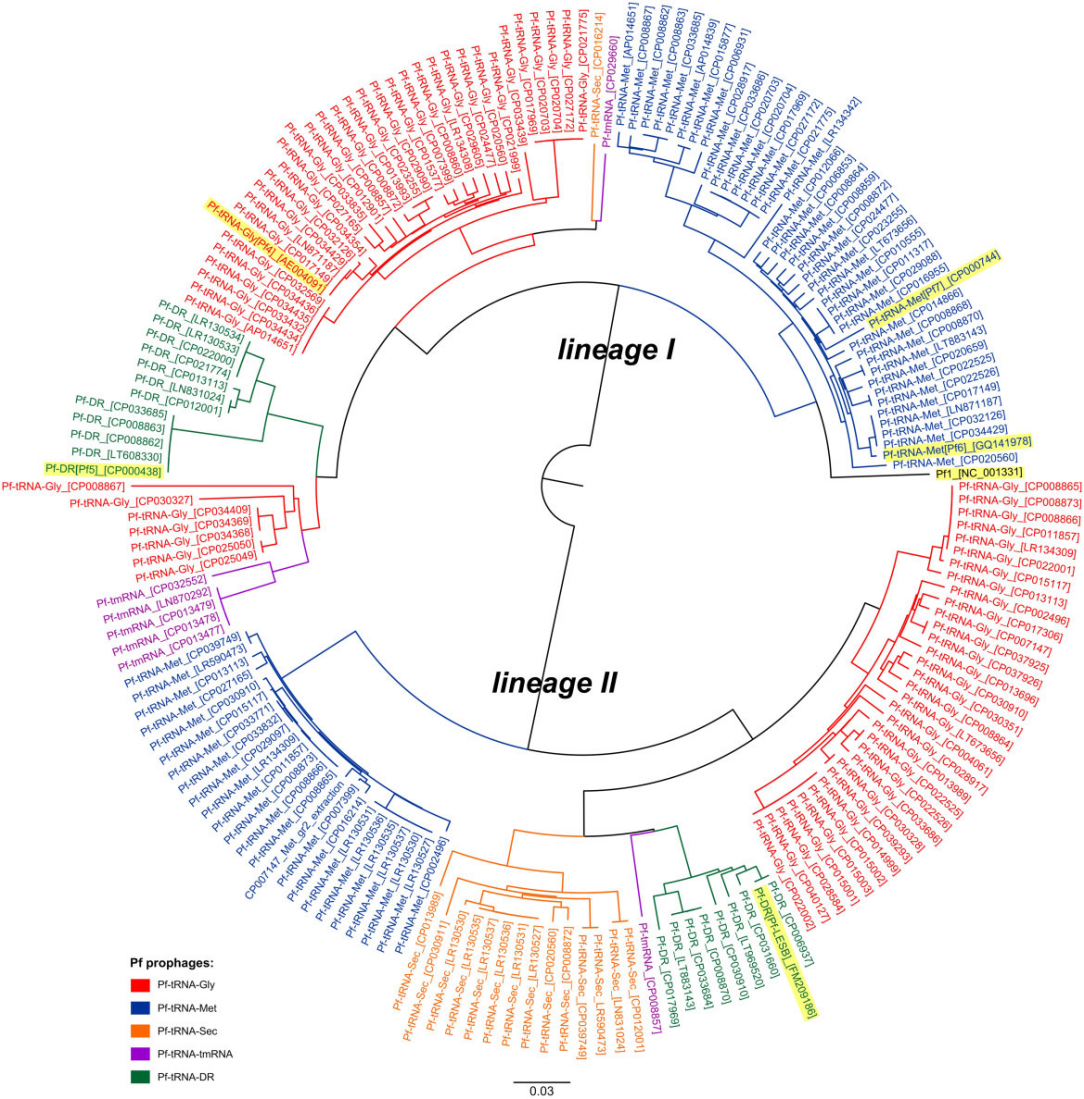
Neighbor-joining phylogenetic tree of the Pf (pro)phages (*n* = 184). MAFFT online service was used to align nucleotide sequences (MAFFT G-INS-1 strategy) and create dendrogram (neighbor-joining method with a bootstrap value of 500 for the conserved sites—5353 bases) (Katoh et al. 2019). The reference Pf phages (Pf1–Pf7 and Pf-LESB58) are highlighted in yellow.

Remarkably, regardless of an integration site all Pf prophages can be divided into two lineages, hereafter referred to as lineage I and lineage II, based on comparison of their DNA sequences (fig. 4), which results mainly from diversity of genes encoding prophages’ structural/morphogenesis proteins (see below). As a result, Pf prophages that utilize the same integration site, for example, Pf-tRNA-Gly, belonging to different lineages, that is, Pf-tRNA-Gly-I and Pf-tRNA-Gly-II, share less overall nucleotide sequence identity to each other, than with the remaining prophages from the same lineage, for example, Pf-tRNA-Met (fig. 4). In addition, Pf prophages from one lineage, for example, Pf-tRNA-Gly-II and Pf-tRNA-Met-II, that show even 100% identity of the major capsid protein (G8P) can exist in one *P. aeruginosa* strain (supplementary fig. S1, Supplementary Material online). On the other hand, we noted that prophages, which occupy one insertion site but represent different lineages, never occur in genomes of *P. aeruginosa* strains representing the same STs.

### Pf Prophages Genomic Structure

The Pf prophage loci estimated by the Phaster tool ranged from 6.2 to 68.9 kb (mean 17.5 kb; median 13.2 kb) and contained 8–49 CDSs (mean 14). In contrast, the size of Pf prophage loci delimited by *attB* and *attP* ranged from 7.9 to 17.9 kb (supplementary table S1, Supplementary Material online). However, it should be noted that in certain chromosomes, the *attP* site is duplicated and/or separated from each other as well as from the *attB* site by regions up to ∼140 kb (supplementary fig. S2, Supplementary Material online).

**Fig. 5.**
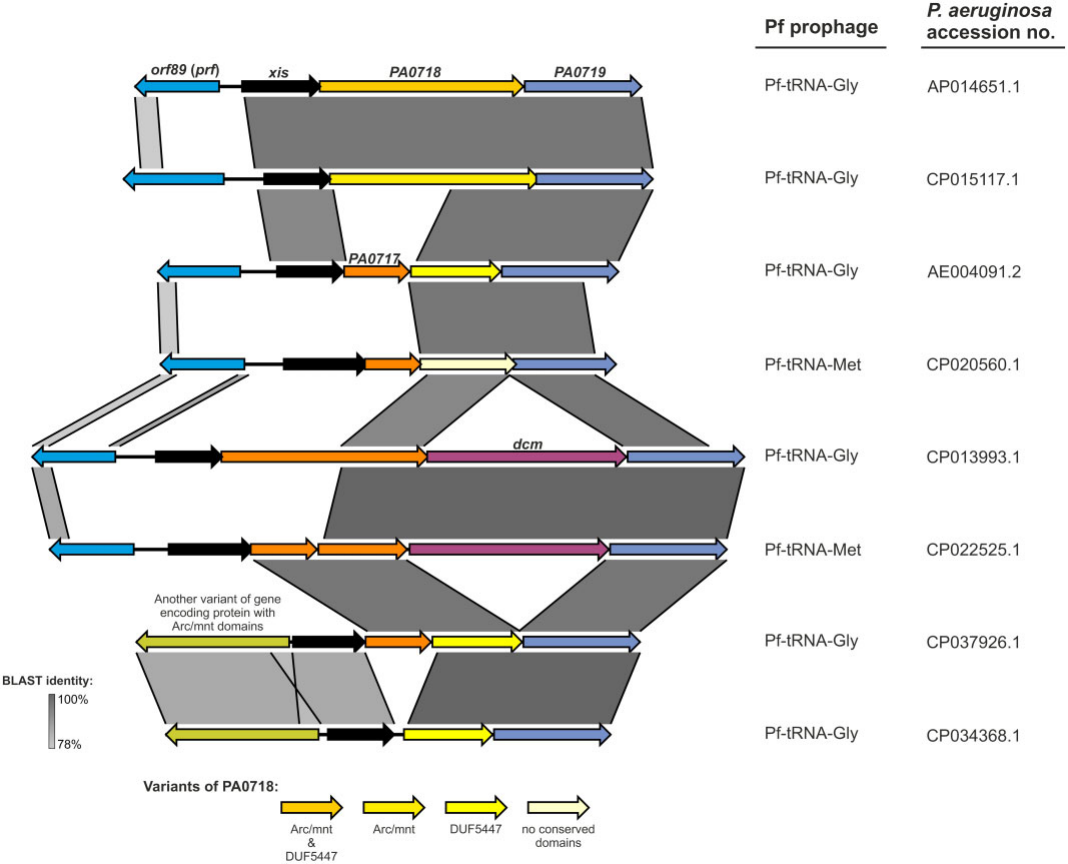
Variation of the region encoding *orf89(prf),xis,* and *PA0717–PA0719* of the Pf prophages.

Using the Pf4 prophage gene nomenclature as the reference, we noted that a set of 12 genes (from *PA0718* to *PA0728*), plus *orf89* (repressor c gene, *pfr*) and the *xis* gene, as well as a small *orf* downstream of *PA0726* encoding hypothetical proteins 33 and 36 amino acids (annotated here as *PA0726′*) are present in virtually all prophages, and hereinafter denoted as “core genes” (fig. 2). However, it appears that the *PA0718* gene in Pf4 is truncated by an insertion of the *PA0717* gene (or pseudogene) (fig. 5), which is present in 64.3% (117/182) of Pf prophages in two unrelated variants encoding hypothetical proteins sharing ∼19.4% amino acid identity (supplementary fig. S3, Supplementary Material online). As a consequence, the hypothetical protein encoded by *PA0718*, equipped with a DUF5447 domain (pfam17525) appears to originate from a disrupted gene encoding Arc family DNA-binding protein, characterized by Arc (pfam03869) and mnt (PHA01513) domains.

Pf prophages without the *PA0717* gene (*n* = 65) have in this position a various length *orf* encoding CDS possessing: 1) Arc and mnt domains (with length ranging from 145 to 225 amino acids, *n* = 30), 2) Arc, mnt, and DUF5447 domains (219 amino acids, *n* = 8), only a DUF5447 domain (115 amino acids, *n* = 5), or 3) without conserved domains (with length ranging from 112 to 174 amino acids, *n* = 22) (supplementary fig. S3, Supplementary Material online). It is noteworthy that the first two variants, that is, with Arc/mnt domains, are present exclusively in the Pf-tRNA-Gly group. Furthermore, an insertion of another variant of *orf* encoding an Arc family DNA-binding protein results in deletion of *orf89* (*pf4r*) in 6.6% (12/182) of Pf prophages (fig. 5). In addition, in 11.5% (21/182) of Pf-tRNA-Met (*n* = 11) and Pf-tRNA-Gly (*n* = 10) prophages from both lineages, the *PA0718* gene is followed by the *dcm* gene (*n* = 15) or pseudogene (*n* = 6) encoding DNA cytosine methyltransferase regardless of the presence of *PA0717* (fig. 5).

**Fig. 6.**
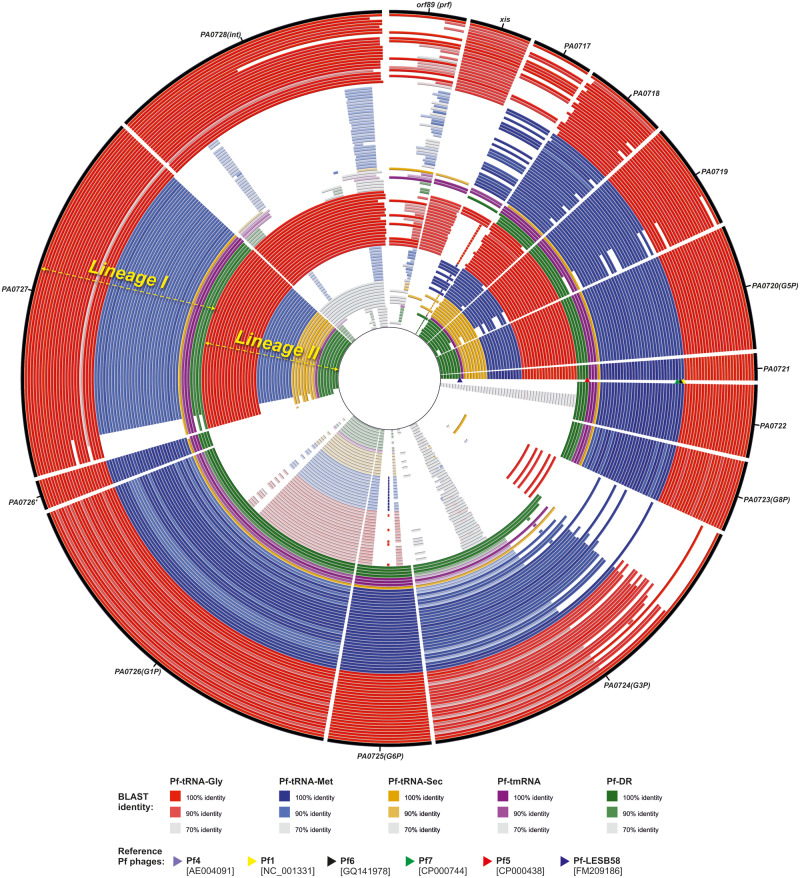
Comparison of core gene similarity of the Pf (pro)phages (*n* = 108) from the lineage I (outer rings) and the lineage II (inner ring). Genes from the Pf4 prophage (the outermost black circle) were used as references. The red, blue, orange, purple, and green circles represent Pf-tRNA-Gly, Pf-tRNA-Met, Pf-tRNA-Sec, Pf-tmRNA, and Pf-DR prophages, respectively. The comparison was performed using BRIG software with BLASTn algorithm (e-value 0.1, word size 7 options). Identical Pf prophages, occupying the same branch of the tree presented in figure 4, were excluded for the clarity. The position of reference Pf prophages, Pf1, Pf6, Pf5, Pf7, and Pf-LESB58, are indicated by colored triangles (yellow, black, green, red, and dark blue) at the PA0721 gene position. The list of Pf prophages was included in supplementary figure S4, Supplementary Material online.

Moreover, among this set of 14–16 core proteins, an amino acid sequence variation of PA0722 (hypothetical protein), PA0723 (G8P, major capsid protein), PA0724 (G3P, minor capsid protein), PA0725 (G6P, minor capsid protein), PA0726 (GIP, Zot), and PA0726′ (hypothetical protein) separates the Pf prophages into the aforementioned lineages I and II. PA0720 (G5P, DNA-binding protein) PA0721 (hypothetical protein) and to some degree also PA0719 (hypothetical protein) and PA0727 (replication initiation protein) have a higher level of sequence conservation. In contrast, Xis4F, Xis5F, and Xis-Met as well as two variants of PA0717 and ORF89 are unevenly distributed across both lineages of Pf prophages (fig. 6).

### Accessory Genes of Pf Prophages

In addition to the core genes, all Pf prophages carry from one to ten (mean 4.2, median 4) accessory *orfs*, that is, located upstream/downstream of the boundary core genes (*int* and *orf89*) and delimited by *attB* and *attP* sites. Thus, the accessory DNA increases the length of Pf prophages’ genomes by 15% to even 100% (mean 45.2%, median 51.0%), for example, from 8 to 16 kb (supplementary table S1, Supplementary Material online).

In total, 773 accessory open reading frames (ORFs) were noted in 182 prophage genomes. Unfortunately, a putative function of half of them is unknown, as 55.5% (429/773) have no conserved domains and 47.5% (367/773) are classified by BLAST analysis just as hypothetical/uncharacterized proteins (supplementary table S2, Supplementary Material online). Likewise, gene ontology category was assigned to 47.7% (369/773) of accessory proteins as follows: cellular component—210, biological function—95, and molecular function—168 proteins (supplementary figs. S5a–c, Supplementary Material online). No virulence or antimicrobial resistance traits were identified among the accessory *orfs*.

Overall, 17.2% (*n* = 133) of the accessory ORFs are unique for single Pf prophages, whereas the remaining 640 ORFs are common to 2–55 Pf prophages (supplementary table S4, Supplementary Material online). However, only 12 of them are shared by at least ten (≥5%) of the prophages (supplementary table S4, Supplementary Material online), including five being a part of two operons (supplementary figs. S6 and S7, Supplementary Material online).

### Distribution of CRISPR-Cas Spacer Sequences in Pf Prophages

We noted a correlation between the Pf prophage lineages and occurrence in their genes of so-called “prospacer” sequences, which are incorporated as “spacers” into the bacterial CRISPR-Cas systems providing sequence-specific immunity (i.e., bacterial adaptive immune system) against viruses and plasmids (Westra et al. 2016) (supplementary fig. S8, Supplementary Material online). In detail, the same prospacers are equally distributed over conserved genes, for example, *PA0719*, *PA0720*, and *PA0727*; whereas in structural/morphogenesis genes, that is, showing sequence variation between the lineages I and II, for example, *PA0723* (*G8P*, major capsid protein), *PA0724* (*G3P*, minor capsid protein), *PA0725*, and *PA0726′*, they are present only in the latter group.

## Discussion

Our analysis revealed that all Pf prophages identified in this study carry integrase (*int*) genes, encoding tyrosine integrases/recombinases XerC/D from the XerC superfamily that catalyze a site-specific cutting and rejoining of recombining DNA. In general, in this process, the integrase-DNA recognition is sequence specific, that is, mediated by complementarity of *attB* and *attP* sites present in the host and the phage genome, respectively. As the result, several bacteriophages, including *E. coli* lambdoid phages, are inserted only at one site and do not recombine with the other (Campbell 2003). Indeed, we noted the evident correlation between integration sites (*attB*), that is, tRNA-Gly, tRNA-Met, tRNA-Sec, tmRNA genes, or 10-bp DRs, and one of five types of integrases. Moreover, integrases utilizing tRNA-Gly and tRNA-Met show specificity for tRNA-Gly with the CCC anticodon (but not with TCC or GCC) and one variant of tRNA-Met among four present in *P. aeruginosa*, respectively. Therefore, the proposed nomenclature of Pf prophages: Pf-tRNA-Gly, -Met, -Sec, -tmRNA, and -DR, with suffix -I and -II, that indicates an affiliation with the lineage I or II (see below) appears to be more informative compared with the current one. The latter is based on 1) numerical approach (i.e., Pf1–Pf7), reflecting the order of their discovery, or 2) naming related to strain specificity (e.g., Pf-LESB58), which is inconsistent and not complete in these aspects (fig. 2). An integrase typing has been recently proposed as a useful method in assessing prophages’ diversity in *Salmonella enterica* genomes (Colavecchio et al. 2017). In view of the above, the presence of truncated *int* gene, a variant specific for the Pf-tRNA-Met prophages, in Pf phage possibly explains its origin and nonintegrative character, that is, additional supported by its excisionase gene (*xis-Met*) common with this group (figs. 2, 4, and 6). Furthermore, in certain Pf prophages, *attP* sites are duplicated and/or separated from *attB* sites by relatively large fragments of the chromosome (supplementary fig. S2, Supplementary Material online). Hence, the capacity of such Pf prophages for excision is questionable, and they may function as permanent lysogens. On the other hand, the replicated *attP* sites may function as “hot spots” that allow rearrangements of the *P. aeruginosa* chromosome.

It is noteworthy that the integration of the Pf phages seems to be unique among other groups of filamentous phages, where this process is mediated by 1) the host XerC/XerD recombinases and the dif (*attB*) site (e.g., in *V. cholerae* phage CTXϕ, *Ralsotonia solanacearum* phage φRSS1 or *Xanthomonas campestris* phage XacF1), 2) transposases and 20-bp inverted repeats serving as *attB* site (e.g., in *Neisseria* Nf phages), and 3) serine-type resolvases/invertases that utilize tRNA-Ser as *attB* site (e.g., *R. solanacearum* phage φRSM) (Askora et al. 2012). Thus, the presence of integrase genes in filamentous phages is not common, which may suggest their recent acquisition or loss in favor of exploiting the host recombinases (Mai-Prochnow et al. 2015).

Remarkably, regardless of the insertion site all Pf prophages are clearly separated into two lineages (I and II) mainly as the result of variation in genes encoding structural/morphogenesis proteins, that is, PA0723 (G8P), PA0724 (G3P), PA0725 (G6P), PA0726 (GIP), as well as PA0722 and PA0726′. Hence, one insertion site, for example, tRNA-Gly, in various *P. aeruginosa* strains can be occupied by the lineage I or II prophages, sharing less overall nucleotide sequence similarity to each other, than with prophages from other integration sites (fig. 4). On the contrary, the proteins responsible for Pf phages replication initiation (PA0727) and stabilization of ssDNA (PA0720, G5P), as well as PA0719 and PA0721 are generally common for both lineages. Likewise, two distinct evolutionary lines of ﬁlamentous phages were also recognized in the plant pathogen *R. solanacearum* (Askora and Yamada 2015).

Enzymes, responsible for excision of Pf prophages from the chromosome, that is, excisionases (Xis) (Li et al. 2019), show notable correlation with the insertion sites, in particular for tRNA-Gly, tRNA-Met, and tRNA-Sec/DR phages. The excision process can be solely Xis-specific, such as excision of Pf5 (Pf-DR) by XisF5, or performed by both excisionase (XisF4) and integrase, as in the case of Pf4 (Pf-tRNA-Gly) phage. Therefore, both enzymes show clear functional distinctness, as excision mediated by XisF4 cannot be reproduced by XisF5 and vice versa (Li et al. 2019). In addition, excisionases may function as transcriptional regulators being a part of system controlling lysogeny and production of Pf phages. For instance, XisF4 directly regulates replication of Pf4 through autoactivation of its own expression and upregulation of the replication initiation gene (*PA0727*) or repression of the phage repressor c gene (*pf4r*, *orf89*) (Li et al. 2019). On the other hand, the expression of *xisF4* is repressed by the Pf4r protein, and two *P. aeruginosa* histone-like nucleoid-structuring (H-NS) proteins: MvaT and MvaU (Castang et al. 2008; Li et al. 2009). Therefore, an increased production of XisF4 and phages may result in a severe growth inhibition of *P. aeruginosa* during late stages of biofilm formation (Li et al. 2019). Furthermore, such mature biofilm phenotype is correlated with the conversion of the Pf4 phage into an SI variant, which in turn may support formation of SCVs (Webb et al. 2003). However, it should be noted that the latter phenomenon was contested by Mooij et al. (2007). Nevertheless, considering the above, it will be interesting to study properties of outstanding from other excisionases the Xis-Met enzyme (fig. 3).

In contrast, the repressor C protein (Orf89, Pfr) that acts as an inhibitor of phage production shows as low as ∼33% inter- and intragroup sequence variation between the Pf prophages. In Pf4 phage, this protein (Pf4r) is not only required for the maintenance of its lysogenic state in PA1 strain but it also confers immunity to Pf4 reinfection (Li et al. 2019), which may be connected with development of an SI form of this phage (Webb et al. 2003). It has been suggested that this process in associated with mutation accumulation within the *pf4r* gene or its upstream region, which is shared with the *xisf4* gene (McElroy et al. 2014; Li et al. 2019). Taking into consideration that Pf4r autoactivates its own expression that depends also on XisF4 level, the mutations may affect its production. In line with this, Hui et al. (2014) suggested that the nucleotide composition of the *pf4r* gene is more prone to acquire single nucleotide polymorphisms than the host genome, which may affect the immunity function of the repressor C protein, allowing the mutant phage to subsequently reinfect hosts with wild-type immunity functions. However, because we noted a high level of sequence variability in the repressor C protein gene and its intergenic region among the Pf prophages, it implicates uneven significance of this phenomenon or its limited occurrence. On the other hand, assuming that ∼40% of the *P. aeruginosa* genomes carry more than one Pf prophage, this diversity may be perceived as an adaptation mechanism allowing the coexistence of various Pf phages in one host without interference between their regulatory processes.

Finally, according to Hui et al. (2014), formation of SI phages appears to be correlated with the DNA mismatch repair (MMR) system and the oxidative stress response mediated by OxyR, a positive regulator of the oxidative stress response gene expression (Wei et al. 2012). The relation between the SI and MMR systems seems to be general, as its inactivation results in a highly mutable state known as hypermutable (mutator) *P. aeruginosa* strains, observed for instance in patients with CF (Oliver et al. 2000). The oxidized form of OxyR directly binds to a 15-bp sequence (-ATAGAGCAAGACTAT-) within the *pf4r* gene and possibly represses its expression (Wei et al. 2012). Thus, it has been suggested that when *pf4r* acquires mutations, they may prevent binding of OxyR and in turn lead to overproduction of the phage particles (Hui et al. 2014). However, as we observed that the OxyR binding site in *pf4r* is perfectly matched with the Pf4-variant only in 12.7% of the Pf prophages, mostly the Pf-tRNA-Gly ones, it remains to be elucidated whether this is a universal mechanism or restricted to certain variants of *pfr*.

Another observation, possibly linked with regulation of Pf prophages’ life cycle, is the sequence diversity of the *PA0718* gene, which is derivative of the presence/lack of the *PA0717* gene. In 38 (20.9%) of the Pf prophages, this gene encodes a possibly complete Arc family DNA-binding protein (∼220 amino acids) characterized by Arc and Mnt domains (*n* = 30) or its variant with a fragment of additional domain of unknown function—DUF5447 (pfam17525) (*n* = 8) (fig. 5). Interestingly, in the *Salmonella* phage, P22 Arc and Mnt are short proteins (53 and 82 amino acids, respectively), which share ∼40% homology and act as repressors of the antirepressor protein Ant for lytic (Arc) and lysogenic cycle of infection (Mnt) (Youderian et al. 1982). During the lytic cycle, the Arc protein negatively regulates its own synthesis as well as production of the antirepressor. The Mnt repressor prevents synthesis of the Arc protein and antirepressor by superinfecting P22 phage (Youderian et al. 1982). Moreover, these repressors and Ant are part of the second regulatory region—*immI*, which together with *immC* is responsible for the establishment and maintenance of lysogeny, where the Ant inactivates the P22 *c2* repressor encoded by the latter region (Susskind and Botstein 1978).

Hence, it is possible that in Pf phages, Arc protein is implicated in regulation of lytic–lysogeny processes in concert with excisionase and repressor C proteins. However, as in 27 (14.8%) of the Pf prophages, it is shorter by the first 50 or more residues encompassing the Arc and Mnt domains, the functions of Arc, either regulatory or others, might have been lost or substantially limited during evolution of Pf phages. On the other hand, in 12 (6.6%) of the Pf prophages, the repressor C protein gene is absent as the result of another variant of Arc gene (fig. 5) insertion, which may implicate a distinct regulatory mechanism.

In the remaining Pf prophages, including Pf1, Pf4–Pf7, and Pf-LESB58, the *PA0718* gene is truncated by an insertion of the *PA0717* gene(s), and its *orf* is frameshifted by an additional ∼19 nucleotides. Thus, the resulting PA0718 protein shares no similarity with the Arc family DNA-binding protein, possessing the entire DUF5447 (92.0%) or no conserved domains (8.0%). Hence, a functionality of these ORFs is a highly questionable or what is less likely, they may represent completely different proteins. Indeed, DUF5447 domain is unique to the *Pseudomonas* genus*.* Moreover, in the genome of Pf1 phage, two alternative *orfs*, ORF96 (DUF5447) and ORF103 (no conserved domains), were annotated in this region, however, the latter was indicated as a possible pseudogene based on codon usage analysis (Hill et al. 1991). Similarly, the function of the *PA0717* gene has not be verified in Pf4 or other Pf phages. Interestingly, *PA0717* occurs in two unrelated variants, unevenly distributed among the Pf prophages, which may implicate a common recombination mechanism behind its insertion.

Summarizing the above observations, it will be interesting to investigate the dynamics and possible synergism or antagonism, between life cycles of ≥2 prophages present in one host. In the first scenario, one phage plays a dominant role over a “recessive” one, assuming that mixing of their capsid proteins during virion assembly is undesirable, and this control may be released with cell population aging, as in the case of mature biofilms and production Pf-SI forms. Alternatively, formation of “hybrid” virions is acceptable and replication of both viruses may occur simultaneously (Askora and Yamada 2015).

Additionally, in 21 (11.5%) of Pf prophages, *PA0718* is followed by the *dcm* gene (or its pseudogene) encoding DNA cytosine methyltransferase (Dcm). In general, methyltransferases (MTases) along with restriction endonucleases (REases) are part of restriction–modification systems (Vasu and Nagaraja 2013) that ensure protection of bacteria against invading bacteriophages and other mobile DNA elements. In this process, methylation of a specific DNA sequence of the host genome by MTases allow discrimination between self- and nonself-DNA by REases, which subsequently cleave unmethylated sequences on foreign DNA. The Dcm enzyme methylates cytosine residues in the sequence CC(A/T)GG, which, for example, occurs 31 times in the Pf1 genome. Interestingly, this motif is methylated also by a newly recognized antiphage module––DISARM (Defence Island System Associated with Restriction–Modification) (Ofir et al. 2018). MTases may occur alone, that is, without cognate REases, as so-called “orphan or solitary” MTases (Murphy et al. 2008). In bacterial cells such orphan MTases are connected with many regulatory activities, including replication, DNA repair, and population evolution of both the host and the bacteriophage genomes (Murphy et al. 2008; Marinus and Lobner-Olesen 2014). Similarly, many bacteriophages possess multi- and mono-specific orphan MTases that beside protecting them from restriction endonucleases of the bacterial host are linked with regulation of their life cycles (Murphy et al. 2008). For instance, the Dam enzyme in the *E. coli* temperate phage P1 is active only during its lytic stage, and the phage DNA encapsidation is dependent on methylation of seven Dam recognition motifs (-GATC-) localized in a 162-bp packaging region (Gladue et al. 2013). Furthermore, expression of the gene-encoding site-specific recombinase (*cre*), and possibly other P1 genes, is controlled by Dam methylation of its promoters (Lobocka et al. 2004). Accordingly, an orphan Dam enzyme of *P. aeruginosa* Mu-like phage B3 was suggested as a part of a regulatory mechanism to control the production of phage proteins (Braid et al. 2004). Likewise, Dam methylation of three GATC sites within a 43-bp region upstream of the *E. coli* bacteriophage Mu *mom* gene was found to participate in controlling its expression (Sun and Hattman 1996). This process prevents binding of the OxyR regulatory protein, which also was suggested to control the life cycle of Pf prophages (see above) (Wei et al. 2012). However, this mechanism is unlikely for Pf phages, as we recognized Dcm methylation motifs (CC(A/T)GG) upstream of the *orf89* gene only in 20 (none with *dcm*) of them. Dam-dependent methylation is necessary for the maintenance of lysogeny of the Shiga toxin carrying lambda-like prophage 933W, and inactivation of the *dam* gene is lethal for enterohemorrhagic *E. coli* strains (Murphy et al. 2008). Similarly, an orphan Dam was indicated as a key factor in controlling the switching between lysogenic and lytic states bacteriophage of the *Vibrio* Harvey myovirus like by methylation of the *rha* antirepressor gene (Bochow et al. 2012). Therefore, it remains to be elucidated whether the Dcm of the Pf phages acts by an antirestriction or regulatory mechanism. Nevertheless, carrying identical *dcm* genes by different Pf prophages present in one host likely indicates their ability to exchange (supplementary fig. S9, Supplementary Material online).

Considering the genetic structure of Pf prophages, the core genomic region, that is, from *orf89* (*pfr*) to *int*, may involve 14–16 *orfs*, including 14 present in all prophages and two, that is, *PA0717* and *dcm*, with limited occurrence. In addition, the *orfs* from the former group can be divided into: 1) conserved (e.g., *PA0727*), 2) group-specific (largely intragroup invariable, *int*, and variable, *orf89/prf*), 3) lineage-specific (e.g., *PA0723*–*PA0726′*). Generally, the most common difference in the gene content of this region is associated with the presence/lack of one of two types of *PA0717*, which results in disruption of the *PA0718* gene. However, the presence of *PA0717* is neither group nor lineage specific.

The intragroup diversity among structural proteins between lineages I and II of Pf prophages from the same group, for example, Pf-tRNA-Gly-I and Pf-tRNA-Gly-II, is self-explanatory from the evolutionary perspective. For instance, it may be interpreted as a consequence of an “arms race” between the bacterium and its virus, which generates a new quality in their mutual interactions. In contrast, a striking intergroup structural similarity of the Pf prophages belonging to the same lineage, for example, Pf-tRNA-Met-II and Pf-tRNA-Gly-II, is somewhat unexpected and difficult to explain from an evolutionary perspective. It implies that the two lineages of Pf prophages have evolved 1) independently from two ancestors by acquisition the same types of integrases (and excisionases) or 2) from one ancestor, for example, lineage I, by intragroup alterations in the structural proteins ultimately leading to the lineage II. Although the former is more natural, it assumes an independent evolution of exactly the same types of integrases (and excisionases) in both lineages, whereas the latter raises the question about reasons or forces behind identical alterations in the five groups of Pf prophages.

Taking into account the second scenario, we noted a correlation between the Pf prophage lineages and uneven distribution of CRISPR-Cas prospacer sequences in their structural/morphogenesis genes (supplementary fig. S8, Supplementary Material online). Interestingly, when the resistance to phages is mediated via multiple spacers, most phages counteract with deletions in genes encoding structural proteins (Watson et al. 2019). In this context, a selective pressure mediated through CRISPR-Cas immunity may act as force that uniformly affects a particular population of phages, hence supporting the “one ancestor” hypothesis of Pf phages evolution (Briner and Barrangou 2016). In general, CRISPR-Cas systems are considered as rapidly evolving defense mechanism and spacers tend to match mostly rare phages from the same (sympatric) rather than other (allopatric) populations, for example, *P. aeruginosa* population in lungs (Westra et al. 2016; England et al. 2018). Accumulation of large number spacers may be deleterious for the bacterium, for example, by triggering of self-targeting and autoimmunity processes. Correspondingly, it is believed that bacteria living together in large populations or bacteriophage-rich environments are more likely to evolve constitutive antiphage defense strategy (e.g., modification/masking of surface receptors) (Westra et al. 2015). Thus, it may explain an accumulation of several prospacers in the excisionase (*xis*) genes of all prophages (except Pf-tRNA-Gly) as well as in the integrase gene of the Pf-tRNA-Met group. On the other hand, these phages may characterize a more “aggressive” life cycle, analogous to the preferentially targeted by CRISPR-Cas phages that actively transcribe prospacer-positive genes and tolerance of temperate ones, so-called “selective immunity” (Obeng et al. 2016). In this light, the Pf-tRNA-Gly phages may represent the oldest, and in turn, the most “domesticated” group of Pf phages (Braga et al. 2018). Additionally, the CRISPR-Cas-mediated pressure is an attractive theory explaining a loss of the functional integrase gene in the Pf1 phage, which shows a close genetic relatedness with the Pf-tRNA-Met group.

As a population, the Pf prophages are characterized by an intergroup redundancy of certain structural proteins, including the major capsid protein (G8P) as well as receptor binding ones—a minor capsid protein (G3P). As a result, one host can be infected by structurally very similar phages, that is, carrying even identical alleles of G8P and G3P proteins, that actually differ only in the *int*/*xis* genes and the regulatory region involving the *orf89* (*prf*) gene (supplementary fig. S1, Supplementary Material online). Because both proteins, in particular G3P, have several alleles, this implies the possibility of an emergence of virions with hybrid capsids, that is, composed of their various alleles, if their assembly is not temporally separated. Furthermore, if the present view on the filamentous phages as bacterial symbionts (rather than parasites) is correct (Hay and Lithgow 2019), it might be speculated that benefits related to alterations in regulation of certain bacterial genes, for example, resulting from the fact of phage insertion at specific site of the bacterial chromosome and/or presence of accessory phage genes (see below), are the most relevant for the host and exceed the burdens of carrying such similar prophages.

At the same time, the dissimilarity of G8P alleles, for example, ∼15% of sequence identity between the dominant ones, in the lineages I and II, possibly may be translated into distinct physicochemical properties of the virions, which in turn are relevant from a perspective of *P. aeruginosa* pathogenicity, for example, biofilm formation (Secor et al. 2015; Burgener et al. 2019) or interactions with eukaryotic cells (Sweere et al. 2019; Secor et al. 2020). For instance, Secor et al. (2015) observed that Pf bacteriophages, as long and negatively charged filaments, spontaneously self-assembly into a liquid crystal structures through entropic interactions between polymers, for example, eDNA and mucin, present in *P. aeruginosa* biofilms, that in turn enhances biofilm adhesion and its stability, iron sequestration, as well as tolerance to desiccation and cationic antibacterial agents (antibiotics and antibacterial peptides) (Secor et al. 2020). The latter phenomenon was verified for cationic aminoglycoside antibiotics (tobramycin) and explained as the result of their enhanced attraction and binding by the liquid crystalline matrix characterized by a high negative charge density. Furthermore, Sweere et al. (2019) have showed that endocytosed Pf1 and Pf4 phages induce antiviral immunity in leukocytes and inhibit antibacterial mechanisms of defense, that is, phagocytosis and TNF production. Hence, Pf phages reduce the number of bacteria required to establish an infection, and anti-Pf antibodies play a protective role against infections caused by Pf-positive *P. aeruginosa* strains. Interestingly, these immune effects were not observed for another filamentous phage—Fd1 from *E. coli*, indicating that it is not universal property this group of viruses.

**Fig. 7.**
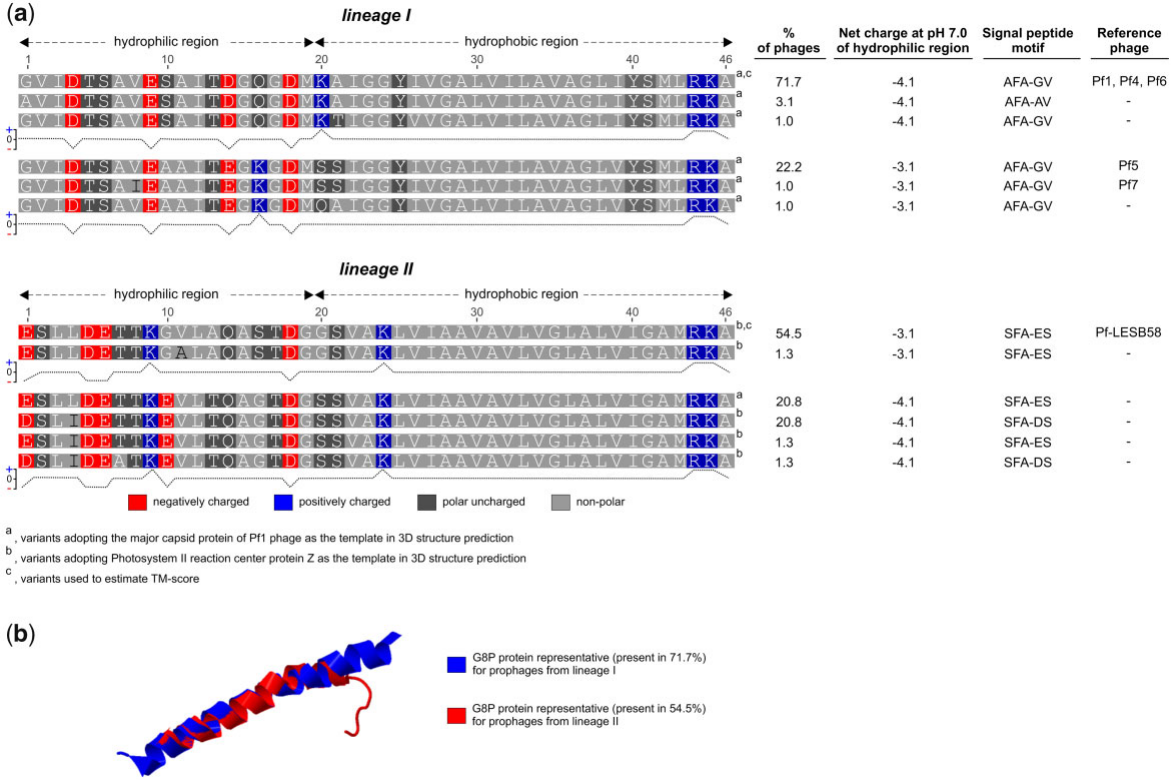
(a) Variants of the major capsid protein (PAO723, G8P) present in the Pf prophages and net charges of their hydrophilic regions, that is, exposed to the environment (Zimmermann et al. 1986). G8P proteins without 26-bp signal sequences were showed, and Protein Calculator v3.4 (http://protcalc.sourceforge.net/; last accessed January 2020) was used to estimate their net charges. (b) Visualization of TM-score (a metric for measuring the similarity of two protein structures) superposition of G8P proteins representative for the lineages I and II (https://zhanglab.ccmb.med.umich.edu/TM-score/). 3D models of G8P were predicted with SWISS-MODEL tool (https://swissmodel.expasy.org/).

Although the same amino acids, that is, aspartic and glutamic acid, are responsible for the negative charge of the outside part of G8P (Zimmermann et al. 1986), in both lineages of Pf prophages, their positions, and in some cases also the number, are different (fig. 7a). In addition, the presence in this region of cationic lysine in certain of G8P alleles contributes to its inter- as well as intralineage charge variation. Considering the above and the fact that one negative charge less per G8P subunit corresponds to ∼30% reduction in its surface charge density (Lyubartsev et al. 1998), it is reasonable to assume also interactions between Pf phages themselves and other molecules may vary. Similarly, also structural differences should be considered in this respect, because for ∼80% of G8P alleles in the lineage II phages, “photosystem II reaction center protein Z” (instead of the major capsid protein of Pf1 phage), is adopted as the closest model in prediction of their 3D structure (fig. 7b). Processing of G8P alleles by the host enzymes is also likely affected, for instance, their signal peptides have different cleavage motifs (fig. 7a). Hence, although in both cases, the cleavage is performed by the type I signal peptidase(s) (SPases), likely the primary *P. aeruginosa* SPase–LepB (PA0768) (Waite et al. 2012), the efficiency of this process may be different (Sakakibara et al. 1993). Additionally, recognition of possible antigenic differences between G8P alleles may be crucial from the perspective of the human immune system response and development of new therapies involving anti-G8P immunization (Secor et al. 2020).

Likewise, at least 12 and 7, signal motifs are present in G3P alleles among the lineage I and II prophages, respectively, but only two of them are shared by both (data not shown). Remarkably, despite substantial differences between G3P alleles, both lineages can infect the same host (supplementary fig. S10, Supplementary Material online). Thus, it may suggest 1) that the recognition of the primary host receptor, that is, type IV pili (PilY) (Martinez and Campos-Gomez 2016), by the lineage II G3P alleles is not (or just marginally) affected and/or 2) an ability of interaction with a new receptor (Azam and Tanji 2019). As the *pilY* gene shows sequence variability among the *P. aeruginosa* strains, in contrast to uniformity of a possible coreceptor—TolA (Ford et al. 2012; Houot et al. 2017), the former case may be an adaptation toward efficient recognition of its specific alleles. On the other hand, the intralineage alterations in G3P affect mostly its first part, that is, containing the N1 domain, responsible for interactions with TolA (data not shown), thus Pf phages may use other coreceptors than their counterparts from *E. coli* and *V. cholerae* TolA (Ford et al. 2012; Houot et al. 2017).

In addition to their core genes, all Pf prophages are equipped with one to ten accessory *orfs*, where 17% of them are unique for single Pf prophages and the remaining are present in ≥2–57 prophages, but only five (0.9%) are shared by at least 20% of the Pf prophages (supplementary table S4, Supplementary Material online).

In general, prophages’ accessory or additional genes are those dispensable for their life cycles, hence, acronymized as “morons”—derived simply from “more on” meaning or called “lysogenic conversion genes” (Brüssow et al. 2004; Tsao et al. 2018). However, from the host perspective, some of them are recognized as “fitness genes,” as they encode traits that (in)directly modify its phenotype or fitness, for example, virulence factors (Brüssow et al. 2004). Filamentous prophages are not an exception. For example, the *ctxAB* operon encoding cholera toxin by CTXϕ phage of *V. cholerae* or the Orf15 carried by ωRSM3 phage modifies the virulence of *R. solonacearum* (Askora and Yamada 2015; Mai-Prochnow et al. 2015).

Unfortunately, a biological function of almost half of the accessory ORFs present in Pf prophages is impossible to predict based on in silico characteristics, however, a substantial number of the remaining proteins is associated with nucleic acids processing.

A bicistronic *parDE* toxin–antitoxin (TA) operon is one of the most common morons present in the majority of Pf-tRNA-Gly, including Pf4 phage, as well as single Pf-tRNA-Sec and Pf-tmRNA prophages. Until recently, TAs have been considered as a rarity in filamentous phages, as the result of limited number of their genomic sequences (Mai-Prochnow et al. 2015). However, a recent comprehensive study focused on searching Pf phages in genomic databases revealed common prevalence of TAs in these viruses (Roux et al. 2019). Moreover, various combinations of TA systems’ genes, even from unrelated TAs, were observed and explained as an evidence of extensive recombination between them (Roux et al. 2019). TAs are small operons composed of a toxin gene and its cognate antitoxin common in bacteria (Fraikin et al. 2020), however, their distribution even across one species may be uneven (Fiedoruk et al. 2015). In general, the toxin is more stable than the antitoxin that prevents its activity through the formation of the antitoxin–toxin complex. TAs are divided into six classes (I–VI) based on the nature (protein or RNA) and mode of action of antitoxin. The TA from the Pf prophages, according to the TA database—TADB2.0 (Shao et al. 2011), shows the highest nucleotide sequence similarity with *parDE* system from class II TAs, thus its toxin—ParE (RelE/ParE family toxin)—affects DNA gyrase, whereas its antitoxin belongs to the Phd/YefM antitoxin family. Originally, TAs were described as stabilizers of plasmids, so-called plasmid “addiction” systems, and subsequently, other mobile DNA, such as superintegrons, conjugative transposons (Fraikin et al. 2020), and likely prophages, for example, CP933P prophage of *E. coli* O157:H7 (Hallez et al. 2010). However, the further recognition of abundance and variety of chromosomal TAs was eventually connected with stress response and adaptation, as elements responsible for the increase of overall fitness through the induction of such processes as 1) biofilm, 2) persister cell formation, and 3) a part of passive immunity as antiphage modules. The last function is motivated as a variant of a wider antiphage strategy known as abortive infection (Abi) phenotype, that is, “altruistic suicide” of the infected cells mediated in this case through toxins of TAs (Labrie et al. 2010). However, a mechanism involving slowing down the cell growth, rather than killing, has been recently suggested for this phenomenon (Song and Wood 2018). Certain phages can bypass TA-mediated Abi by encoding molecules that 1) mimic the bacterial antitoxin, for example, Dmd protein of *E. coli* T4 phage and pseudo-ToxI RNA of *Pectobacterium atrosepticum* phage ϕTE or 2) inhibit the bacterial proteases responsible for the antitoxin degradation, for example, Gp4.5, PinA, and RexB proteins of *E. coli* Y7, T4, and λ phages, respectively. On the other hand, carrying TAs by prophages may be perceived as an antiaddictive mechanism protecting the host from other mobile elements, such as plasmids possessing similar TAs. Furthermore, from the perspective of phages themselves, TAs may function as a competitive advantage, by analogy with TA-mediated plasmid displacement, where a plasmid equipped with TA can outcompete another plasmid belonging to the same incompatibility but without the TA (Fraikin et al. 2020). This may explain a limitation of the *parDE* operon mostly to Pf-tRNA-Gly phages.

On the other hand, Petrova et al. (2011) reported that cell lysis by the SI form of Pf4 phage is regulated by BfmR being a part of two-component signal transduction system via activation of expression of Phd (prevent-host-death, PA0691), that is, TA system antitoxin homolog, and its overexpression results in resistance to the SI infection. Therefore, BfmR and Phd were suggested as biofilm development regulators by reducing SI-mediated cell lysis and in turn releasing eDNA. Although amino acid sequence identity for the *P. aeruginosa* Phd and Pf Phd/YefM family antitoxin is low (13.3%), both proteins likely share some structural and possibly functional similarities (supplementary fig. S11, Supplementary Material online). Hence, the Pf TA may be perceived as an adaption mechanism to evade action of Phd by 1) blocking its promoter by the Pf Phd/YefM antitoxin or 2) its binding with ParE toxin. Finally, more recently Li et al. have suggested to name this TAs, PfiT/PfiA (Pf inhibition toxin and antitoxin), since it can control production of Pf4 phage as well as contribute to cell immunity to Pf4 phage infection in an autoregulatory manner (Li et al. 2020). In detail, the PfiT by binding with PfiA acts as its corepressor and the PfiAT complex controls *pfiAT* operon via interaction with a palindromic sequence overlapping the -35 region of this operon. However, the deletion of *pfiT* removes this autoregulatory control, which results in an activation of expression of the gene encoding Pf4 replication initiation protein and bypasses the immunity ensured by the phage repressor protein Pf4r (Li et al. 2020).

Nevertheless, regardless of the function, the accessory genes appear to significantly contribute to the diversity of Pf prophages, and their limitation to certain groups (supplementary table S4, Supplementary Material online) and/or variety seem to be natural consequence of the insertional specificity, amplified by occurrence of Pf prophages in other *Pseudomonas* species (supplementary fig. S6, Supplementary Material online), as well as genes exchange between phages (Mai-Prochnow et al. 2015; Roux et al. 2019).

In conclusion, several meaningful aspects of *P. aeruginosa* Pf (pro)phage biology await clarification, in particular from the perspective of their (dis)similarity and common coexistence in one host. So far, all research data came from experiments based on three model Pf phages, namely Pf1, Pf4 and Pf5, all representing the same evolutionary line, that is, lineage I. However, our study revealed the existence of the equally numerous lineage II, characterized by identical integration/excision features, but structurally different. Therefore, the population of Pf phages consists of two forms exploiting and likely competing for the same hosts and chromosomal “niches,” which in turn may lead to a variety of interactions, ranging from antagonistic to mutualistic, between phages themselves and bacteriophages. Both lineages are possibly represented by fractions of Pf phages that replicate exclusively as extrachromosomal elements, like Pf1, which in the context of 1) Pf prophages’ life cycle regulatory mechanisms and 2) their accessory genes, can be treated as somewhat defective Pf viruses. Knowledge about their prevalence and distribution among *P. aeruginosa* strains is necessary to properly decipher all the mechanisms behind interactions and evolution of Pf (pro)phages.

## Supplementary Material

Supplementary data are available at *Genome Biology and Evolution* online.

## Supplementary Material

evaa146_Supplementary_Data

## References

[evaa146-B1] Alikhan NF Petty NK Ben Zakour NL Beatson SA. 2011 BLAST Ring Image Generator (BRIG): simple prokaryote genome comparisons. BMC Genomics. 12(1):402. 21824423 10.1186/1471-2164-12-402 PMC3163573

[evaa146-B2] Arndt D, et al 2016 PHASTER: a better, faster version of the PHAST phage search tool. Nucleic Acids Res. 44(W1):W16–W21. 27141966 10.1093/nar/gkw387 PMC4987931

[evaa146-B3] Askora A Abdel-Haliem ME Yamada T. 2012 Site-specific recombination systems in filamentous phages. Mol Genet Genomics. 287(7):525–530. 22661259 10.1007/s00438-012-0700-1

[evaa146-B4] Askora A Yamada T. 2015 Two different evolutionary lines of filamentous phages in *Ralstonia solanacearum*: their effects on bacterial virulence. Front Genet. 6:217. 26150828 10.3389/fgene.2015.00217 PMC4471427

[evaa146-B5] Azam AH Tanji Y. 2019 Bacteriophage-host arm race: an update on the mechanism of phage resistance in bacteria and revenge of the phage with the perspective for phage therapy. Appl Microbiol Biotechnol. 103(5):2121–2131. 30680434 10.1007/s00253-019-09629-x

[evaa146-B6] Bochow S Elliman J Owens L. 2012 Bacteriophage adenine methyltransferase: a life cycle regulator? Modelled using Vibrio harveyi myovirus like. J Appl Microbiol. 113(5):1001–1013. 22681538 10.1111/j.1365-2672.2012.05358.x

[evaa146-B7] Braga PP, et al 2018 Bacterial diversification in the light of the interactions with phages: the genetic symbionts and their role in ecological speciation. Front Ecol Evol. 6. doi:10.3389/fevo.2018.00006

[evaa146-B8] Braid MD Silhavy JL Kitts CL Cano RJ Howe MM. 2004 Complete genomic sequence of bacteriophage B3, a Mu-like phage of *Pseudomonas aeruginosa*. J Bacteriol. 186(19):6560–6574. 15375138 10.1128/JB.186.19.6560-6574.2004 PMC516594

[evaa146-B9] Briner AE Barrangou R. 2016 Deciphering and shaping bacterial diversity through CRISPR. Curr Opin Microbiol. 31:101–108. 27045713 10.1016/j.mib.2016.03.006

[evaa146-B10] Brüssow H Canchaya C Hardt W-D. 2004 Phages and the evolution of bacterial pathogens: from genomic rearrangements to lysogenic conversion. Microbiol Mol Biol Rev. 68(3):560–602. 15353570 10.1128/MMBR.68.3.560-602.2004 PMC515249

[evaa146-B11] Bucki R, et al 2015 Polyelectrolyte-mediated increase of biofilm mass formation. BMC Microbiol. 15(1):117. 26048182 10.1186/s12866-015-0457-x PMC4458031

[evaa146-B12] Burgener EB, et al 2019 Filamentous bacteriophages are associated with chronic *Pseudomonas* lung infections and antibiotic resistance in cystic fibrosis. Sci Transl Med. 11(488):eaau9748. 30996083 10.1126/scitranslmed.aau9748 PMC7021451

[evaa146-B13] Campbell A. 2003 Prophage insertion sites. Res Microbiol. 154(4):277–282. 12798232 10.1016/S0923-2508(03)00071-8

[evaa146-B14] Castang S McManus HR Turner KH Dove SL. 2008 H-NS family members function coordinately in an opportunistic pathogen. Proc Natl Acad Sci U S A. 105(48):18947–18952. 19028873 10.1073/pnas.0808215105 PMC2596223

[evaa146-B15] Chen L Zheng D Liu B Yang J Jin Q. 2016 VFDB 2016: hierarchical and refined dataset for big data analysis—10 years on. Nucleic Acids Res. 44(D1):D694–D697. 26578559 10.1093/nar/gkv1239 PMC4702877

[evaa146-B16] Colavecchio A, et al 2017 Prophage integrase typing is a useful indicator of genomic diversity in *Salmonella enterica*. Front Microbiol. 8:1283. 28740489 10.3389/fmicb.2017.01283 PMC5502288

[evaa146-B17] Conesa A Gotz S. 2008 Blast2GO: a comprehensive suite for functional analysis in plant genomics. Int J Plant Genomics. 2008:1–12. 10.1155/2008/619832 PMC2375974 18483572

[evaa146-B18] Doster E, et al 2020 MEGARes 2.0: a database for classification of antimicrobial drug, biocide and metal resistance determinants in metagenomic sequence data. Nucleic Acids Res. 48(D1):D561–D569. 31722416 10.1093/nar/gkz1010 PMC7145535

[evaa146-B19] England WE Kim T Whitaker RJ. 2018 Metapopulation structure of CRISPR-Cas immunity in *Pseudomonas aeruginosa* and its viruses. mSystems. 3(5):e00075–18. 30374457 10.1128/mSystems.00075-18 PMC6199469

[evaa146-B20] Fasano A, et al 1995 Zonula occludens toxin modulates tight junctions through protein kinase C-dependent actin reorganization, in vitro. J Clin Invest. 96(2):710–720. 7635964 10.1172/JCI118114 PMC185254

[evaa146-B21] Feldgarden M, et al 2019 Validating the AMRFinder tool and resistance gene database by using antimicrobial resistance genotype-phenotype correlations in a collection of isolates. Antimicrob Agents Chemother. 63(11):e00483–19. 31427293 10.1128/AAC.00483-19 PMC6811410

[evaa146-B22] Fiedoruk K Daniluk T Swiecicka I Sciepuk M Leszczynska K. 2015 Type II toxin-antitoxin systems are unevenly distributed among *Escherichia coli* phylogroups. Microbiology 161(1):158–167. 25378561 10.1099/mic.0.082883-0

[evaa146-B23] Ford CG, et al 2012 Crystal structures of a CTXφ pIII domain unbound and in complex with a *Vibrio cholerae* TolA domain reveal novel interaction interfaces. J Biol Chem. 287(43):36258–36272. 22942280 10.1074/jbc.M112.403386 PMC3476293

[evaa146-B24] Fraikin N Goormaghtigh F Van Melderen L. 2020 Type II toxin-antitoxin systems: evolution and revolutions. J Bacteriol. 202(7). doi:10.1128/JB.00763-19 PMC7167474 31932311

[evaa146-B25] Gladue DP, et al 2013 Foot-and-mouth disease virus modulates cellular vimentin for virus survival. J Virol. 87(12):6794–6803. 23576498 10.1128/JVI.00448-13 PMC3676138

[evaa146-B26] Gupta SK, et al 2014 ARG-ANNOT, a new bioinformatic tool to discover antibiotic resistance genes in bacterial genomes. Antimicrob Agents Chemother. 58(1):212–220. 24145532 10.1128/AAC.01310-13 PMC3910750

[evaa146-B27] Hallez R, et al 2010 New toxins homologous to ParE belonging to three-component toxin-antitoxin systems in *Escherichia coli* O157:H7. Mol Microbiol. 76(3):719–732. 20345661 10.1111/j.1365-2958.2010.07129.x

[evaa146-B28] Haussler S. 2004 Biofilm formation by the small colony variant phenotype of *Pseudomonas aeruginosa*. Environ Microbiol. 6(6):546–551. 15142242 10.1111/j.1462-2920.2004.00618.x

[evaa146-B29] Hay ID Lithgow T. 2019 Filamentous phages: masters of a microbial sharing economy. EMBO Rep. 20(6):e47427. 30952693 10.15252/embr.201847427 PMC6549030

[evaa146-B30] Hill DF Short NJ Perham RN Petersen GB. 1991 DNA sequence of the filamentous bacteriophage Pf1. J Mol Biol. 218(2):349–364. 2010913 10.1016/0022-2836(91)90717-k

[evaa146-B31] Houot L, et al 2017 Electrostatic interactions between the CTX phage minor coat protein and the bacterial host receptor TolA drive the pathogenic conversion of *Vibrio cholerae*. J Biol Chem. 292(33):13584–13598. 28642371 10.1074/jbc.M117.786061 PMC5566518

[evaa146-B32] Hui JG Mai-Prochnow A Kjelleberg S McDougald D Rice SA. 2014 Environmental cues and genes involved in establishment of the superinfective Pf4 phage of *Pseudomonas aeruginosa*. Front Microbiol. 5:654. 25520708 10.3389/fmicb.2014.00654 PMC4251444

[evaa146-B33] Jia B, et al 2017 CARD 2017: expansion and model-centric curation of the comprehensive antibiotic resistance database. Nucleic Acids Res. 45(D1):D566–D573. 27789705 10.1093/nar/gkw1004 PMC5210516

[evaa146-B34] Katoh K Rozewicki J Yamada KD. 2019 MAFFT online service: multiple sequence alignment, interactive sequence choice and visualization. Brief Bioinform. 20(4):1160–1166. 28968734 10.1093/bib/bbx108 PMC6781576

[evaa146-B35] King A, Lefkowitz E, Adams M, Carstens E., editors. 2011. Family Inoviridae. In: *Virus taxonomy: Ninth Report of the International Committee on Taxonomy of Viruses.* 1st ed. London: Elsevier Academic Press. p. 375–381.

[evaa146-B36] Klockgether J, et al 2010 Genome diversity of *Pseudomonas aeruginosa* PAO1 laboratory strains. J Bacteriol. 192(4):1113–1121. 20023018 10.1128/JB.01515-09 PMC2812968

[evaa146-B37] Knezevic P Voet M Lavigne R. 2015 Prevalence of Pf1-like (pro)phage genetic elements among *Pseudomonas aeruginosa* isolates. Virology 483:64–71. 25965796 10.1016/j.virol.2015.04.008

[evaa146-B38] Labrie SJ Samson JE Moineau S. 2010 Bacteriophage resistance mechanisms. Nat Rev Microbiol. 8(5):317–327. 20348932 10.1038/nrmicro2315

[evaa146-B39] Lee Y, et al 2018 Substrate binding protein DppA1 of ABC transporter DppBCDF increases biofilm formation in *Pseudomonas aeruginosa* by inhibiting Pf5 prophage lysis. Front Microbiol. 9:30. 29416528 10.3389/fmicb.2018.00030 PMC5787571

[evaa146-B40] Li C Wally H Miller SJ Lu CD. 2009 The multifaceted proteins MvaT and MvaU, members of the H-NS family, control arginine metabolism, pyocyanin synthesis, and prophage activation in *Pseudomonas aeruginosa* PAO1. J Bacteriol. 191(20):6211–6218. 19684136 10.1128/JB.00888-09 PMC2753020

[evaa146-B41] Li Y, et al 2019 Excisionase in Pf filamentous prophage controls lysis-lysogeny decision-making in *Pseudomonas aeruginosa*. Mol Microbiol. 111:495–513. 30475408 10.1111/mmi.14170 PMC7379572

[evaa146-B42] Li Y, et al 2020 Prophage encoding toxin/antitoxin system PfiT/PfiA inhibits Pf4 production in *Pseudomonas aeruginosa*. Microb Biotechnol. 13:1132–1144. 32246813 10.1111/1751-7915.13570 PMC7264888

[evaa146-B43] Lobocka MB, et al 2004 Genome of bacteriophage P1. J Bacteriol. 186:7032–7068. 15489417 10.1128/JB.186.21.7032-7068.2004 PMC523184

[evaa146-B44] Lyubartsev AP Tang JX Janmey PA Nordenskiöld L. 1998 Electrostatically induced polyelectrolyte association of rodlike virus particles. Phys Rev Lett. 81(24):5465–5468.

[evaa146-B45] Mai-Prochnow A, et al 2015 Big things in small packages: the genetics of filamentous phage and effects on fitness of their host. FEMS Microbiol Rev. 39(4):465–487. 25670735 10.1093/femsre/fuu007

[evaa146-B46] Marinus MG Lobner-Olesen A. 2014 DNA methylation. EcoSal Plus 1(4). doi:10.1128/ecosalplus.ESP-0003 PMC4231299 26442938

[evaa146-B47] Martinez E Campos-Gomez J. 2016 Pf filamentous phage requires UvrD for replication in *Pseudomonas aeruginosa*. mSphere 1:e00104–e00115. 10.1128/mSphere.00104-15 PMC4863604 27303696

[evaa146-B48] McElroy KE, et al 2014 Strain-specific parallel evolution drives short-term diversification during *Pseudomonas aeruginosa* biofilm formation. Proc Natl Acad Sci U S A. 111(14):E1419–E1427. 24706926 10.1073/pnas.1314340111 PMC3986123

[evaa146-B49] Mooij MJ, et al 2007 Characterization of the integrated filamentous phage Pf5 and its involvement in small-colony formation. Microbiology 153(6):1790–1798. 17526836 10.1099/mic.0.2006/003533-0 PMC3820363

[evaa146-B50] Murphy KC Ritchie JM Waldor MK Løbner-Olesen A Marinus MG. 2008 Dam methyltransferase is required for stable lysogeny of the Shiga toxin (Stx2)-encoding bacteriophage 933W of enterohemorrhagic *Escherichia coli* O157:h 7. J Bacteriol. 190(1):438–441. 17981979 10.1128/JB.01373-07 PMC2223730

[evaa146-B51] Nazik H, et al 2017 *Pseudomonas* phage inhibition of *Candida albicans*. Microbiology 163(11):1568–1577. 28982395 10.1099/mic.0.000539

[evaa146-B52] Obeng N Pratama AA Elsas JDV. 2016 The significance of mutualistic phages for bacterial ecology and evolution. Trends Microbiol. 24(6):440–449. 26826796 10.1016/j.tim.2015.12.009

[evaa146-B53] Ofir G, et al 2018 DISARM is a widespread bacterial defence system with broad anti-phage activities. Nat Microbiol. 3(1):90–98. 29085076 10.1038/s41564-017-0051-0 PMC5739279

[evaa146-B54] Oliver A Canton R Campo P Baquero F Blazquez J. 2000 High frequency of hypermutable *Pseudomonas aeruginosa* in cystic fibrosis lung infection. Science 288(5469):1251–1254. 10818002 10.1126/science.288.5469.1251

[evaa146-B55] Penner JC, et al 2016 Pf4 bacteriophage produced by *Pseudomonas aeruginosa* inhibits *Aspergillus fumigatus* metabolism via iron sequestration. Microbiology 162(9):1583–1594. 27473221 10.1099/mic.0.000344

[evaa146-B56] Petrova OE Schurr JR Schurr MJ Sauer K. 2011 The novel *Pseudomonas aeruginosa* two-component regulator BfmR controls bacteriophage-mediated lysis and DNA release during biofilm development through PhdA. Mol Microbiol. 81(3):767–783. 21696457 10.1111/j.1365-2958.2011.07733.x PMC3214647

[evaa146-B57] Platt MD, et al 2008 Proteomic, microarray, and signature-tagged mutagenesis analyses of anaerobic *Pseudomonas aeruginosa* at pH 6.5, likely representing chronic, late-stage cystic fibrosis airway conditions. J Bacteriol. 190(8):2739–2758. 18203836 10.1128/JB.01683-07 PMC2293228

[evaa146-B58] Rice SA, et al 2009 The biofilm life cycle and virulence of *Pseudomonas aeruginosa* are dependent on a filamentous prophage. ISME J. 3(3):271–282. 19005496 10.1038/ismej.2008.109 PMC2648530

[evaa146-B59] Roux S, et al 2019 Cryptic inoviruses revealed as pervasive in bacteria and archaea across Earth’s biomes. Nat Microbiol. 4(11):1895–1906. 31332386 10.1038/s41564-019-0510-x PMC6813254

[evaa146-B60] Sakakibara Y Tsutsumi K Nakamura K Yamane K. 1993 Structural requirements of *Bacillus subtilis* alpha-amylase signal peptide for efficient processing: in vivo pulse-chase experiments with mutant signal peptides. J Bacteriol. 175(13):4203–4212. 8320234 10.1128/jb.175.13.4203-4212.1993 PMC204850

[evaa146-B61] Sauer K, et al 2004 Characterization of nutrient-induced dispersion in *Pseudomonas aeruginosa* PAO1 biofilm. J Bacteriol. 186(21):7312–7326. 15489443 10.1128/JB.186.21.7312-7326.2004 PMC523207

[evaa146-B62] Secor PR, et al 2020 Pf bacteriophage and their impact on *Pseudomonas* virulence, mammalian immunity, and chronic infections. Front Immunol. 11:244. 32153575 10.3389/fimmu.2020.00244 PMC7047154

[evaa146-B63] Secor PR, et al 2016 Biofilm assembly becomes crystal clear—filamentous bacteriophage organize the *Pseudomonas aeruginosa* biofilm matrix into a liquid crystal. Microb Cell. 3(1):49–52. 10.15698/mic2016.01.475 PMC5354590 28357315

[evaa146-B64] Secor PR, et al 2017 Filamentous bacteriophage produced by *Pseudomonas aeruginosa* alters the inflammatory response and promotes noninvasive infection in vivo. Infect Immun. 85(1):e00648–16. 27795361 10.1128/IAI.00648-16 PMC5203648

[evaa146-B65] Secor PR, et al 2015 Filamentous bacteriophage promote biofilm assembly and function. Cell Host Microbe. 18(5):549–559. 26567508 10.1016/j.chom.2015.10.013 PMC4653043

[evaa146-B66] Seemann T. 2014 Prokka: rapid prokaryotic genome annotation. Bioinformatics 30(14):2068–2069. 24642063 10.1093/bioinformatics/btu153

[evaa146-B67] Seol-Hee K Kyoung-Boon L Ji-Sun L You-Hee C. 2003 Genome diversification by phage-derived genomic islands in *Pseudomonas aeruginosa*. J Microbiol Biotechnol. 13:783–788.

[evaa146-B68] Shao Y, et al 2011 TADB: a web-based resource for Type 2 toxin-antitoxin loci in bacteria and archaea. Nucleic Acids Res. 39(Suppl 1):D606–D611. 20929871 10.1093/nar/gkq908 PMC3013778

[evaa146-B69] Song S Wood TK. 2018 Post-segregational killing and phage inhibition are not mediated by cell death through toxin/antitoxin systems. Front Microbiol. 9(814). 10.3389/fmicb.2018.00814 PMC5996881 29922242

[evaa146-B70] Sullivan MJ Petty NK Beatson SA. 2011 Easyfig: a genome comparison visualizer. Bioinformatics 27(7):1009–1010. 21278367 10.1093/bioinformatics/btr039 PMC3065679

[evaa146-B71] Sun W Hattman S. 1996 *Escherichia coli* OxyR protein represses the unmethylated bacteriophage Mu mom operon without blocking binding of the transcriptional activator C. Nucleic Acids Res. 24(20):4042–4049. 8918810 10.1093/nar/24.20.4042 PMC146201

[evaa146-B72] Susskind MM Botstein D. 1978 Molecular genetics of bacteriophage P22. Microbiol Rev. 42(2):385–413. 353481 10.1128/mr.42.2.385-413.1978 PMC281435

[evaa146-B73] Sweere JM, et al 2019 Bacteriophage trigger antiviral immunity and prevent clearance of bacterial infection. Science 363(6434):eaat9691. 30923196 10.1126/science.aat9691 PMC6656896

[evaa146-B74] Tsao YF, et al 2018 Phage morons play an important role in *Pseudomonas aeruginosa* phenotypes. J Bacteriol. 200(22). 10.1128/JB.00189-18 PMC6199475 30150232

[evaa146-B75] Vasu K Nagaraja V. 2013 Diverse functions of restriction-modification systems in addition to cellular defense. Microbiol Mol Biol Rev. 77(1):53–72. 23471617 10.1128/MMBR.00044-12 PMC3591985

[evaa146-B76] Waite RD, et al 2012 *Pseudomonas aeruginosa* possesses two putative type I signal peptidases, LepB and PA1303, each with distinct roles in physiology and virulence. J Bacteriol. 194(17):4521–4536. 22730125 10.1128/JB.06678-11 PMC3415513

[evaa146-B77] Watson BNJ, et al 2019 Different genetic and morphological outcomes for phages targeted by single or multiple CRISPR-Cas spacers. Philos Trans R Soc B. 374(1772):20180090. 10.1098/rstb.2018.0090 PMC6452268 30905290

[evaa146-B78] Webb JS Lau M Kjelleberg S. 2004 Bacteriophage and phenotypic variation in *Pseudomonas aeruginosa* biofilm development. J Bacteriol. 186(23):8066–8073. 15547279 10.1128/JB.186.23.8066-8073.2004 PMC529096

[evaa146-B79] Webb JS, et al 2003 Cell death in *Pseudomonas aeruginosa* biofilm development. J Bacteriol. 185(15):4585–4592. 12867469 10.1128/JB.185.15.4585-4592.2003 PMC165772

[evaa146-B80] Wei Q, et al 2012 Global regulation of gene expression by OxyR in an important human opportunistic pathogen. Nucleic Acids Res. 40(10):4320–4333. 22275523 10.1093/nar/gks017 PMC3378865

[evaa146-B81] Westra ER Dowling JA Broniewski JM van Houte S. 2016 Evolution and ecology of CRISPR. Annu Rev Ecol Evol Syst. 47:307–331.

[evaa146-B82] Westra ER, et al 2015 Parasite exposure drives selective evolution of constitutive versus inducible defense. Curr Biol. 25(8):1043–1049. 25772450 10.1016/j.cub.2015.01.065

[evaa146-B83] Whiteley M, et al 2001 Gene expression in *Pseudomonas aeruginosa* biofilms. Nature 413(6858):860–864. 11677611 10.1038/35101627

[evaa146-B84] Youderian P Chadwick SJ Susskind MM. 1982 Autogenous regulation by the bacteriophage P22 arc gene product. J Mol Biol. 154(3):449–464. 7042983 10.1016/s0022-2836(82)80006-5

[evaa146-B85] Zankari E, et al 2012 Identification of acquired antimicrobial resistance genes. J Antimicrob Chemother. 67(11):2640–2644. 22782487 10.1093/jac/dks261 PMC3468078

[evaa146-B86] Zimmermann K Hagedorn H Heuck CC Hinrichsen M Ludwig H. 1986 The ionic properties of the filamentous bacteriophages Pf1 and fd. J Biol Chem. 261(4):1653–1655. 3944103

