# Supplementary material for: Two Lineages of Pseudomonas aeruginosa Filamentous Phages: Structural Uniformity over Integration Preferences
Source: Genome Biol Evol. 2020 Jul 13;12(10):1765–81. doi: 10.1093/gbe/evaa146 (PMC7549136; doi:10.1093/gbe/evaa146)
Supplement: evaa146_Supplementary_Data [file evaa146_supplementary_data.pdf]

Two lineages of *Pseudomonas aeruginosa* filamentous phages – structural uniformity over integration preferences

Krzysztof Fiedoruk[1]*, Magdalena Zakrzewska[1], Tamara Daniluk[1], Ewelina Piktel[1], Sylwia Chmielewska[1], Robert Bucki[1]*

[1] Department of Medical Microbiology and Nanobiomedical Engineering, Medical University of Białystok, Mickiewicza 2c, 15-222 Białystok, Poland

Supplementary Material online

**Table S1**	*Pseudomonas aeruginosa* strains (n=190) screened for Pf prophages.
**Table S2**	Characteristics of the Pf prophages accessory proteins (n=773).
**Table S3**	Characteristics of accessory proteins (n=773) from the Pf prophages.
**Table S4**	Characteristics of 12 most common (i.e. present in ≥ 5% of Pf prophages) accessory proteins.
**Figure S1**	Comparison of two prophages from the lineage II (Pf-tRNA-Met-II and Pf-tRNA-Gly-II) present in *P. aeruginosa* YL84 (CP007147).
**Figure S2**	Pf prophages with duplicated and/or atypical location of *attP* sites.
**Figure S3**	Alignment of PA0718 variants present in the Pf prophages.
**Figure S4**	Comparison of core gene similarity of the Pf (pro)phages (n=108) from the lineage I (outer rings) and the lineage II (inner ring). Genes from the Pf4 prophage (the outermost black circle) were used as references. The red, blue, orange, purple and green circles represent Pf-tRNA-Gly, Pf-tRNA-Met, Pf-tRNA-Sec, Pf-tmRNA and Pf-DR prophages, respectively. The comparison was performed using BRIG software with blastn algorithm (evalue 0.1, word size 7 options). Identical Pf prophages, occupying the same branch of the tree presented in Fig. 4, were excluded for the clarity. The position of reference Pf prophages: Pf1, Pf6, Pf5, Pf7 and Pf-LESB58, are indicated by coloured triangles (yellow, black, green, red and dark blue) at the *PA0721* gene position.
**Figure S5a**	Accessory proteins of the Pf prophages classified into Cellular Component (CC) category (n=210).
**Figure S5b**	Accessory proteins of the Pf prophages classified into Biological Function (BF) category (n=95).
**Figure S5c**	Accessory proteins of the Pf prophages classified into Molecular Function (MF) category (n=168).
**Figure S6**	*ParDE* toxin-antitoxin operon and its possible origin.
**Figure S7**	Operon composed of a putative Cro/CI family transcriptional regulator, GNAT family N-acetyltransferase and adenylate kinase present in certain Pf-tRNA-Met prophages, and its possible origin.
**Figure S8**	Distribution of CRISPR-Cas spacer sequences in genes of the Pf (pro)phages (n=108) from the lineage I (outer rings) and the lineage II (inner rings). List of the spacer sequences, their affiliation to CRISPR-Cas type and *P. aeruginosa* strains carrying them are included in the table below. The red, blue, orange, purple and green circles represent Pf-tRNA-Gly, Pf-tRNA-Met, Pf-tRNA-Sec, Pf-tmRNA and Pf-DR prophages, respectively. The comparison was performed using BRIG software with blastn algorithm (evalue 0.1, word size 7 options). Identical Pf prophages, i.e. occupying the same branch of the tree presented in Fig. 4, were excluded for the clarity. The list of Pf prophages was included in supplementary figure S4, Supplementary Material online.
**Figure S9**	Identity of *dcm* genes carried by Pf-tRNA-Gly and Pf-tRNA-Met prophages present in *P. aerugionosa* isolate PcyII-1 (LT673656.1).
**Figure S10**	Comparison of three prophages from the lineage I (Pf-tRNA-Met-I and Pf-tRNA-Gly-I) and lineage II (Pf-tRNA-Sec-II) present in *P. aeruginosa* strain X78812 (CP008872).
**Figure S11**	Visualization of TM-score (a metric for measuring the similarity of two protein structures) superposition of *P. aeruginosa* Phd (white thick wirefames) and Phd/YefM family antitoxin from Pf prophages (white thin wirefames), red colour denotes the residue pairs of distance < 5.0 Angstrom (https://zhanglab.ccmb.med.umich.edu/TM-score/). 3D models of the proteins were predicted with SWISS-MODEL tool (https://swissmodel.expasy.org/).

Table S1. *Pseudomonas aeruginosa* strains (n=190) screened for Pf prophages.

			MLST								Phaster results						This study												
No.	Strain	Genbank Accession No.	ST	*acsA*	*aroE*	*guaA*	*mutL*	*nuoD*	*ppsA*	*trpE*	Region Length	Completeness	Score	# Total Proteins	Region Position	Most Common Phage	Integration site	Lineage	Core genome [kb]	Core genome GC%	# of CDS in core genome*	Type of *PA0717* gene	Conserved domains (CD) in PA0718	*dcm* gene	Comment to CDS	Whole genome [kb]	Whole genome GC%	Difference between the length of whole and core genome [kb]	Difference between the length of whole and core genome [%]
1	Pa PAO1	AE004091.2	549	7	5	12	3	4	1	7	15.7Kb	intact	117	14	780995-796776	PHAGE–Pseudo–Pf1–NC–001331(11)	tRNA-Gly	I	8.2	62	15	type 1	DUF5447	no		12.4	56.4	4.2	33.8
2	Pa strain: NCGM257	AP014651.1	357	2	4	5	3	1	6	11	8.3Kb	questionable	75	8	5330378-5338689	PHAGE–Pseudo–Pf1–NC–001331(7)	tRNA-Gly	II	8.5	61.5	14	no	Arc/mnt	no		12.7	56.7	4.2	33.1
	Pa strain: NCGM257	AP014651.1	357	2	4	5	3	1	6	11	14.9Kb	intact	107	12	5937899-5952858	PHAGE–Pseudo–Pf1–NC–001331(10)	tRNA-Met	I	8.4	61	15	type 1	DUF5447	no		15.0	53.9	6.6	44.0
3	Pa strain: 8380	AP014839.2	2619	17	5	1	3	4	4	3	14.1Kb	intact	117	18	5562886-5577039	PHAGE–Pseudo–Pf1–NC–001331(11)	tRNA-Met	I	8.4	61.8	15	type 1	DUF5447	no		13.7	56.5	5.3	38.8
4	Pa UCBPP-PA14	CP000438.1	253	4	4	16	12	1	6	3	18.9Kb	intact	117	20	4344257-4363246	PHAGE–Pseudo–Pf1–NC–001331(11)	DR	I	8.4	61.2	15	type 1	DUF5447	no		10.6	57.3	2.2	21.1
5	Pa PA7	CP000744.1	1195	87	34	43	37	53	107	126	16.6Kb	intact	96	17	5528487-5545105	PHAGE–Pseudo–Pf1–NC–001331(9)	tRNA-Met	I	8.2	61.2	14	no	DUF5447	no		12.9	56.9	4.7	36.2
6	Pa M18	CP002496.1	1239	16	5	1	3	4	15	7	10.8Kb	questionable	75	8	4757935-4768820	PHAGE–Pseudo–Pf1	tRNA-Gly	II	8.0	60.8	14	no	Arc/mnt	no		10.9	55.4	2.9	26.6
	Pa M18	CP002496.1	1239	16	5	1	3	4	15	7	12.9Kb	questionable	85	10	5282006-5294965	PHAGE–Pseudo–Pf1	tRNA-Met	II	8.8	60.6	16	type 2	DUF5447	yes		13.0	55	4.2	32.6
7	Pa B136-33	CP004061.1	1024	2	4	24	3	1	6	25	10.8Kb	questionable	75	9	4762518-4773416	PHAGE–Pseudo–Pf1–NC–001331(7)	tRNA-Gly	II	8.1	60.8	15	type 2	DUF5447	no		10.9	57.5	2.8	25.7
8	Pa MTB-1	CP006853.1	2689	5	8	3	5	1	11	3	11.2Kb	intact	96	12	5496778-5508057	PHAGE–Pseudo–Pf1–NC–001331(9)	tRNA-Met	I	8.3	62.3	14	type 1	DUF5447	no	disruption of PA0717 by stop codon (TAA); lack of orf89, an insertion of orf encoding DNA-binding protein	11.7	57.4	3.3	28.6
9	Pa SCV20265	CP006931.1	299	17	5	36	3	3	7	3	13.5Kb	intact	96	11	5642786-5656346	PHAGE–Pseudo–Pf1–NC–001331(9)	tRNA-Met	I	8.4	61.6	15	type 1	DUF5447	no		13.6	54	5.2	38.4
10	Pa LES431	CP006937.1	146	6	5	11	3	4	23	1	18.8Kb	questionable	85	12	4491563-4510446	PHAGE–Pseudo–Pf1–NC–001331(8)	DR	II	8.2	60	15	type 1	DUF5447	no		10.5	56.1	2.3	22.3
11	Pa YL84	CP007147.1	-	40	5	30	5	3	33	14	19.7Kb	questionable	75	11	2403725-2423458	PHAGE–Pseudo–Pf1–NC–001331(7)	tRNA-Gly	II	8.2	60.9	14	no	Arc/mnt	no		15.9	54.1	7.7	48.3
	Pa YL84	CP007147.1	-	40	5	30	5	3	33	14	15Kb	questionable	75	14	1861490-1876514	PHAGE–Pseudo–Pf1–NC–001331(7)	tRNA-Met	II	8.2	61.1	15	type 1	no CD	no		11.9	55.5	3.8	31.6
12	Pa strain F22031	CP007399.1	485	11	76	5	3	61	14	3	68.9Kb	intact	150	49	912749-981735	PHAGE–Pseudo–Pf1–NC–001331(8)	tRNA-Gly	II	9.7	61.5	15	no	Arc/mnt	no	disruption of PA0724 by IS3 family transposase IS222 (2 orfs)	13.4	58.3	3.7	27.9
	Pa strain F22031	CP007399.1	485	11	76	5	3	61	14	3	14.4Kb	intact	107	14	1484949-1499404	PHAGE–Pseudo–Pf1–NC–001331(10)	tRNA-Met	I	8.2	60.5	15	type 1	no CD	no		14.5	53.2	6.3	43.7
13	Pa strain F30658	CP008857.1	111	17	5	5	4	4	4	3	9.7Kb	incomplete	30	8	2568890-2578675	PHAGE–Pseudo–Pf1–NC–001331(5)	tmRNA	II	8.1	61	14	no	no CD	no		9.8	57.8	1.7	17.5
	Pa strain F30658	CP008857.1	111	17	5	5	4	4	4	3	23.4Kb	questionable	85	11	2795772-2819213	PHAGE–Pseudo–Pf1–NC–001331(8)	tRNA-Gly	I	8.2	62.1	15	type 2	DUF5447	no		11.3	59.1	3.1	27.3
14	Pa strain H5708	CP008859.2	3050	4	5	6	3	4	4	19	13.3Kb	intact	128	18	5287414-5300809	PHAGE–Pseudo–Pf1–NC–001331(12)	tRNA-Met	I	8.3	61.3	15	type 1	DUF5447	no		13.5	56.5	5.1	38.2
15	Pa strain H27930	CP008860.2	389	17	22	5	3	1	14	3	10.5Kb	intact	117	14	4896098-4906604	PHAGE–Pseudo–Pf1–NC–001331(11)	tRNA-Gly	I	8.4	61.6	15	type 1	DUF5447	no	lack of orf89, an insertion of another variant of orf encoding proteins with Arc/mnt domain	10.5	58.1	2.1	20.1
16	Pa strain M1608	CP008862.2	253	4	4	16	12	1	6	3	12.6Kb	intact	117	19	2235585-2248264	PHAGE–Pseudo–Pf1–NC–001331(11)	DR	I	8.4	61.2	15	type 1	DUF5447	no		10.6	57.3	2.2	21.1
	Pa strain M1608	CP008862.2	253	4	4	16	12	1	6	3	8.4Kb	intact	128	13	5423538-5431978	PHAGE–Pseudo–Pf1–NC–001331(12)	tRNA-Met	I	8.4	61.8	15	type 1	DUF5447	no		13.2	55.7	4.8	36.5
17	Pa strain M37351	CP008863.1	253	4	4	16	12	1	6	3	11.2Kb	intact	96	12	5788959-5800215	PHAGE–Pseudo–Pf1–NC–001331(9)	DR	I	8.4	61.8	15	type 1	DUF5447	no		13.2	55.7	4.8	36.5
	Pa strain M37351	CP008863.1	253	4	4	16	12	1	6	3	8.4Kb	questionable	85	9	2515450-2523890	PHAGE–Pseudo–Pf1–NC–001331(8)	tRNA-Met	I	8.4	61.2	15	type 1	DUF5447	no		10.6	57.3	2.2	21.1
18	Pa strain W60856	CP008864.2	959	6	5	11	7	3	70	19	25.6Kb	questionable	85	14	1555122-1580780	PHAGE–Pseudo–Pf1–NC–001331(8)	tRNA-Gly	II	8.2	61	14	no	Arc/mnt	no		12.9	54.5	4.6	36.1
	Pa strain W60856	CP008864.2	959	6	5	11	7	3	70	19	12.1Kb	intact	117	17	5851588-5863741	PHAGE–Pseudo–Pf1–NC–001331(11)	tRNA-Met	I	8.9	62	16	type 2	DUF5447	yes		11.7	58.2	2.8	23.8
19	Pa strain S86968	CP008865.2	155	28	5	36	3	3	13	7	13.1Kb	intact	96	14	5199097-5212228	PHAGE–Pseudo–Pf1–NC–001331(9)	tRNA-Gly	II	8.2	60.8	14	no	Arc/mnt, DUF5447	no		12.1	56.6	3.9	31.9
	Pa strain S86968	CP008865.2	155	28	5	36	3	3	13	7	17.3Kb	intact	107	24	5838264-5855652	PHAGE–Pseudo–Pf1–NC–001331(10)	tRNA-Met	II	8.1	61.1	15	type 1	DUF5447	no	disruption of PA0726 by stop codon (TGA)	14.2	56.8	6.1	42.9
20	Pa strain T38079	CP008866.2	155	28	5	36	3	3	13	7	13.1Kb	intact	96	14	5045962-5059093	PHAGE–Pseudo–Pf1–NC–001331(9)	tRNA-Gly	II	8.2	60.8	14	no	Arc/mnt, DUF5447	no		12.1	56.6	3.8	31.9
	Pa strain T38079	CP008866.2	155	28	5	36	3	3	13	7	15.1Kb	intact	107	21	5717600-5732738	PHAGE–Pseudo–Pf1–NC–001331(10)	tRNA-Met	II	8.1	61.1	15	type 1	DUF5447	no	disruption of PA0726 by stop codon (TGA)	12.0	56.2	3.9	32.1
21	Pa strain T52373	CP008867.1	-	16	10	5	3	~64	42	7	10.6Kb	questionable	75	8	927093-937700	PHAGE–Pseudo–Pf1–NC–001331(7)	tRNA-Gly	I	8.4	60.7	15	type 1	DUF5447	no	disruption of PA0724 by stop codon (TGA)	12.4	54.5	4.0	32.5
	Pa strain T52373	CP008867.1	-	16	10	5	3	~64	42	7	11.8Kb	questionable	85	10	1451116-1462948	PHAGE–Pseudo–Pf1–NC–001331(8)	tRNA-Met	I	8.4	61.6	15	type 1	DUF5447	no	disruption of PA0727 by stop codon (TGA)	11.9	56.8	3.5	29.7
22	Pa strain T63266	CP008868.1	132	6	20	1	3	4	4	2	11.7Kb	intact	96	10	609077-620826	PHAGE–Pseudo–Pf1–NC–001331(9)	tRNA-Met	I	8.3	62.1	15	type 1	DUF5447	no		11.8	56.9	3.5	29.9
23	Pa strain W36662	CP008870.2	17	11	5	1	7	9	4	7	17.8Kb	questionable	85	17	4576147-4594035	PHAGE–Pseudo–Pf1–NC–001331(8)	DR	II	8.2	61.1	16	no	no CD	no		15.5	56	7.3	46.9
	Pa strain W36662	CP008870.2	17	11	5	1	7	9	4	7	22.6Kb	intact	128	24	5744500-5767146	PHAGE–Pseudo–Pf1–NC–001331(12)	tRNA-Met	I	8.2	60.6	15	type 1	DUF5447	no		10.3	57.2	2.1	20.3
24	Pa strain X78812	CP008872.2	257	35	24	36	11	4	15	14	14.3Kb	intact	117	17	4751457-4765802	PHAGE–Pseudo–Pf1–NC–001331(11)	tRNA-Gly	I	8.4	61.7	14	no	Arc/mnt	no		14.4	54.4	5.9	41.4
	Pa strain X78812	CP008872.2	257	35	24	36	11	4	15	14	28.3Kb	intact	117	20	5267544-5295873	PHAGE–Pseudo–Pf1–NC–001331(11)	tRNA-Met	I	8.3	61.8	15	type 2	DUF5447	no		14.8	54.2	6.5	43.9
	Pa strain X78812	CP008872.2	257	35	24	36	11	4	15	14	22.2Kb	intact	96	12	5428772-5451066	PHAGE–Pseudo–Pf1–NC–001331(9)	tRNA-Sec	II	8.2	61.7	15	type 1	DUF5447	no		12.0	56.3	3.8	31.7
25	Pa strain F9670	CP008873.1	155	28	5	36	3	3	13	7	9.6Kb	questionable	75	12	3123786-3133458	PHAGE–Pseudo–Pf1–NC–001331(7)	tRNA-Gly	II	8.2	60.8	14	no	Arc/mnt, DUF5447	no		12.1	56.6	3.9	31.9
	Pa strain F9670	CP008873.1	155	28	5	36	3	3	13	7	10.6Kb	incomplete	40	10	2451247-2461942	PHAGE–Pseudo–Pf1–NC–001331(6)	tRNA-Met	II	8.1	61.1	15	type 1	DUF5447	no	disruption of PA0725 by stop codon (TGA)	12.0	56.2	3.9	32.1
26	Pa strain FRD1	CP010555.1	111	17	5	5	4	4	4	3	29.4Kb	intact	107	13	5306831-5336258	PHAGE–Pseudo–Pf1–NC–001331(10)	tRNA-Met	I	8.3	62.1	16	type 2	DUF5447	no	disruption of PA0724 by stop codon (TAG)	11.1	58.2	2.8	25.1
27	Pa strain Carb01 63	CP011317.1	111	17	5	5	4	4	4	3	11.5Kb	intact	117	15	6390124-6401628	PHAGE–Pseudo–Pf1–NC–001331(11)	tRNA-Met	I	8.3	62.1	15	type 2	DUF5447	no		11.1	58.2	2.7	24.7
28	Pa strain ATCC 27853	CP011857.1	155	28	5	36	3	3	13	7	10.7Kb	questionable	85	12	5780692-5791399	PHAGE–Pseudo–Pf1–NC–001331(8)	tRNA-Met	II	8.1	61.1	16	type 1	DUF5447	no	disruption of PA0726 by stop codon (TGA)	12.0	56.2	3.9	32.1
	Pa strain ATCC 27853	CP011857.1															tRNA-Gly	II	8.2	60.8	14	no	Arc/mnt, DUF5447	no		12.1	56.6	3.9	31.9
29	Pa DSM 50071	CP012001.1	-	175	5	1	11	3	6	7	19.1Kb	intact	107	12	4218555-4237752	PHAGE–Pseudo–Pf1–NC–001331(10)	DR	I	8.3	61.1	14	no	no CD	no		10.8	57.4	2.5	23.2
	Pa DSM 50071	CP012001.1	-	175	5	1	11	3	6	7	7.3Kb	questionable	85	10	5427322-5434673	PHAGE–Pseudo–Pf1–NC–001331(8)	tRNA-Sec	II	8.1	60.6	15	type 2	DUF5447	no		9.5	58	1.4	14.6
30	Pa strain F9676	CP012066.1	-	40	5	11	5	4	28	37	15.5Kb	intact	128	14	605406-620965	PHAGE–Pseudo–Pf1–NC–001331(12)	tRNA-Met	I	9.0	61.3	16	type 1	DUF5447	yes		15.6	54.2	6.6	42.4
31	Pa strain N15-01092	CP012901.1	654	17	5	26	3	4	4	26	13.7Kb	intact	96	13	1580595-1594352	PHAGE–Pseudo–Pf1–NC–001331(9)	tRNA-Gly	I	8.4	61.8	14	no	Arc/mnt	no		9.8	59.1	1.4	13.9
32	Pa strain PAER4–119	CP013113.1	260	14	5	10	7	4	13	7	18.2Kb	intact	96	16	4411192-4429408	PHAGE–Pseudo–Pf1–NC–001331(9)	DR	I	8.3	61.1	14	no	no CD	no		10.4	57.1	2.1	19.9
	Pa strain PAER4–119	CP013113.1	260	14	5	10	7	4	13	7	13.2Kb	questionable	85	11	4919710-4932989	PHAGE–Pseudo–Pf1–NC–001331(8)	tRNA-Gly	II	8.2	60.7	14	no	Arc/mnt	no		13.3	54.2	5.1	38.1
	Pa strain PAER4–119	CP013113.1	260	14	5	10	7	4	13	7	15.8Kb	intact	107	13	5448309-5464171	PHAGE–Pseudo–Pf1–NC–001331(10)	tRNA-Met	II	7.9	61	14	no	no CD	no		15.9	54.1	8.0	50.1
33	Pa strain SCVFeb	CP013477.1	387	28	5	11	11	4	12	3	7.6Kb	questionable	85	10	4563441-4571063	PHAGE–Pseudo–Pf1–NC–001331(8)	tmRNA	I	8.3	61	15	type 2	DUF5447	no		11.5	58.3	3.2	27.7
34	Pa strain SCVJan	CP013478.1	387	28	5	11	11	4	12	3	7.6Kb	questionable	85	10	4563444-4571066	PHAGE–Pseudo–Pf1–NC–001331(8)	tmRNA	I	8.3	61	15	type 2	DUF5447	no		11.5	58.3	3.2	27.7
35	Pa strain NHmuc	CP013479.1	387	28	5	11	11	4	12	3	12.1Kb	questionable	85	15	1359979-1372110	PHAGE–Pseudo–Pf1–NC–001331(8)	tmRNA	I	8.3	61	15	type 2	DUF5447	no		11.5	58.3	3.2	27.7
36	Pa strain 12-4-4(59)	CP013696.1	152	6	5	19	3	4	6	7	11.7Kb	questionable	85	11	6373125-6384863	PHAGE–Pseudo–Pf1–NC–001331(8)	tRNA-Gly	II	8.0	60.7	15	type 2	DUF5447	no		11.8	55.8	3.7	31.8
37	Pa strain USDA-ARS-USMARC-4163	CP013989.1	-	11	8	19	115	4	13	18	8.8Kb	questionable	85	10	4830978-4839876	PHAGE–Pseudo–Pf1–NC–001331(8)	tRNA-Gly	II	8.2	61.1	14	no	Arc/mnt	no		12.1	55.9	3.9	31.8
	Pa strain USDA-ARS-USMARC-4163	CP013989.1	-	11	8	19	115	4	13	18	16.6Kb	questionable	85	10	5492020-5508686	PHAGE–Pseudo–Pf1–NC–001331(8)	tRNA-Sec	II	8.2	60.9	15	type 2	DUF5447	no	lack of orf89, an insertion of another variant of orf encoding proteins with Arc/mnt domain	10.1	57.8	1.9	18.6
38	Pa DHS01	CP013993.1	395	6	5	1	1	1	12	1	15.6Kb	intact	107	14	5457600-5473274	PHAGE–Pseudo–Pf1–NC–001331(10)	tRNA-Gly	II	9.1	61.7	15	no	Arc/mnt	yes		14.5	55.9	5.4	37.3
39	Pa strain PA–154197	CP014866.1**	550	119	10	3	5	64	6	77	–	–	–	–	5403507-5411452	–	tRNA-Met	I	8.3	62.2	15	type 1	DUF5447	no		11.2	58	2.9	25.8
40	Pa strain PA7790	CP014999.1	277	39	5	9	11	27	5	2	30.2Kb	intact	96	21	5258959-5289256	PHAGE–Pseudo–Pf1–NC–001331(9)	tRNA-Gly	II	8.1	61.1	15	type 1	DUF5447	no		12.4	55.4	4.3	34.6
41	Pa strain PA1088	CP015001.1	277	39	5	9	11	27	5	2	12.3Kb	questionable	75	11	5013820-5026176	PHAGE–Pseudo–Pf1–NC–001331(7)	tRNA-Gly	II	8.1	61.1	15	type 1	DUF5447	no		12.4	55.4	4.3	34.6
42	Pa strain PA8281	CP015002.1	277	39	5	9	11	27	5	2	12.3Kb	intact	107	13	5203592-5215948	PHAGE–Pseudo–Pf1–NC–001331(10)	tRNA-Gly	II	8.1	61.1	15	type 1	DUF5447	no		12.4	55.4	4.3	34.6
43	Pa strain PA11803	CP015003.1	277	39	5	9	11	27	5	2	30.2Kb	intact	107	21	5394152-5424449	PHAGE–Pseudo–Pf1–NC–001331(10)	tRNA-Gly	II	8.1	61.1	15	type 1	DUF5447	no		12.4	55.4	4.3	34.6
44	Pa strain ATCC 27853	CP015117.1	155	28	5	36	3	3	13	7	10.5Kb	intact	96	13	2539900-2550496	PHAGE–Pseudo–Pf1–NC–001331(9)	tRNA-Met	II	8.1	61	15	type 1	DUF5447	no		12.0	56.1	3.9	32.1
	Pa strain ATCC 27853	CP015117.1															tRNA-Gly	II	8.2	60.7	14	no	Arc/mnt, DUF5447	no		12.1	56.6	3.9	31.9
45	Pa strain BAMCPA07-48	CP015377.1	313	47	8	7	6	8	11	40	11.3Kb	intact	107	11	329606-340955	PHAGE–Pseudo–Pf1–NC–001331(10)	tRNA-Gly	I	8.2	61.9	14	no	Arc/mnt	no		11.4	56.7	3.2	27.8
46	Pa SJTD-1	CP015877.1	2619	17	5	1	3	4	4	3	9.1Kb	intact	128	14	2045828-2055017	PHAGE–Pseudo–Pf1–NC–001331(12)	tRNA-Met	I	8.4	61.8	15	type 1	DUF5447	no		13.7	56.5	5.3	38.8
47	Pa strain PA121617	CP016214.1	389	17	22	5	3	1	14	3	13.2Kb	questionable	85	14	2162552-2175754	PHAGE–Pseudo–Pf1–NC–001331(8)	tRNA-Met	I	8.1	60.4	15	type 2	DUF5447	no		13.3	55.4	5.1	38.7
	Pa strain PA121617	CP016214.1	389	17	22	5	3	1	14	3	7.2Kb	intact	96	10	2323712-2330924	PHAGE–Pseudo–Pf1–NC–001331(9)	tRNA-Sec	II	8.5	61.7	14	no	Arc/mnt	no		11.0	58.6	2.6	23.3
48	Pa strain RIVM-EMC2982	CP016955.1	111	17	5	5	4	4	4	3	11.4Kb	intact	117	15	1096046-1107462	PHAGE–Pseudo–Pf1–NC–001331(11)	tRNA-Met	I	8.3	62.1	15	type 2	DUF5447	no	disruption of PA0724 by stop codon (TGA)	11.1	58.2	2.8	25.1
49	Pa strain ATCC 15692	CP017149.1	549	7	5	12	3	4	1	7	12.3Kb	intact	117	15	4717609-4729991	PHAGE–Pseudo–Pf1–NC–001331(11)	tRNA-Gly	I	8.2	62		type 1	DUF5447	no		12.4	56.3	4.2	33.6
	Pa strain ATCC 15692	CP017149.1	549	7	5	12	3	4	1	7	12Kb	intact	107	11	5241559-5253628	PHAGE–Pseudo–Pf1–NC–001331(10)	tRNA-Met	I	8.3	61.9	15	type 1	DUF5447	no	disruption of PA0724 by stop codon (TGA)	12.1	56	3.8	31.6
50	Pa strain PA–150577	CP017306.1	1239	16	5	1	3	4	15	7	11.2Kb	questionable	75	10	4765593-4776858	PHAGE–Pseudo–Pf1–NC–001331(7)	tRNA-Gly	II	8.3	60.6	14	no	Arc/mnt	no		11.3	56.3	3.0	26.9
51	Pa isolate B10W	CP017969.1**	308	13	4	5	5	12	7	15	–	–	–	–	4905130 .. 4910833	Pseudomonas phage Pf1	tRNA-Gly	I	9.5	61.3	16	no	Arc/mnt, DUF5447	yes	disruption of PA0717 and PA0728 by stop codon (TGA)	15.5	56	6.0	38.7
	Pa isolate B10W	CP017969.1**	308	13	4	5	5	12	7	15	–	–	–	–	5430515 .. 5438469	Pseudomonas phage Pf1	tRNA-Met	I	8.3	61.2	15	type 2	DUF5447	no		12.7	55.2	4.3	34.2
	Pa isolate B10W	CP017969.1															DR	II	8.3	60.3	14	no	no CD	no	lack of orf89, an insertion of another variant of orf encoding proteins with Arc/mnt domain	10.4	56.9	2.1	20.0
52	Pa strain CR1	CP020560.1	3198	87	148	15	35	53	109	177	11.5Kb	intact	107	11	4553204-4564721	PHAGE–Pseudo–Pf1–NC–001331(10)	tRNA-Gly	I	8.2	62	15	type 2	DUF5447	no		11.5	56.9	3.3	28.8
	Pa strain CR1	CP020560.1	3198	87	148	15	35	53	109	177	23.2Kb	intact	117	21	5080701-5103922	PHAGE–Pseudo–Pf1–NC–001331(11)	tRNA-Met	I	8.3	61.2	15	type 2	no CD	no		14.8	54	6.5	43.8
	Pa strain CR1	CP020560.1	3198	87	148	15	35	53	109	177	21.3Kb	intact	96	17	5239092-5260444	PHAGE–Pseudo–Pf1–NC–001331(9)	tRNA-Sec	II	8.1	61.3	14	no	no CD	no		10.1	57.7	2.0	19.5
53	Pa PAK	CP020659.1	693	11	5	11	11	4	4	14	12.4Kb	intact	96	15	4551159-4563600	PHAGE–Pseudo–Pf1–NC–001331(9)	tRNA-Met	I	8.4	60.8	15	type 1	DUF5447	no		12.0	56	3.6	30.3
54	Pa strain PASG–M345	CP020703.1	308	13	4	5	5	12	7	15	15.5Kb	intact	107	13	5278811-5294335	PHAGE–Pseudo–Pf1–NC–001331(10)	tRNA-Gly	I	9.5	61.3	17	no	Arc/mnt	yes	disruption of PA0728 by stop codon (TAG)	15.6	56	6.0	38.7
	Pa strain PASG–M345	CP020703.1	308	13	4	5	5	12	7	15	13.4Kb	intact	117	19	5805632-5819123	PHAGE–Pseudo–Pf1–NC–001331(11)	tRNA-Met	I	8.3	61.2	15	type 2	DUF5447	no	disruption of PA0720 by stop codon (TGA)	12.7	55.2	4.3	34.2
55	Pa strain PASG–M699	CP020704.1	308	13	4	5	5	12	7	15	15.5Kb	intact	107	13	5370748-5386272	PHAGE–Pseudo–Pf1–NC–001331(10)	tRNA-Gly	I	9.1	61.7	15	no	Arc/mnt	yes	disruption of PA0728 by stop codon (TAG)	15.6	56	6.4	41.4
	Pa strain PASG–M699	CP020704.1	308	13	4	5	5	12	7	15	13.4Kb	intact	117	19	5897568-5911059	PHAGE–Pseudo–Pf1–NC–001331(11)	tRNA-Met	I	8.3	61.2	15	type 2	DUF5447	no	disruption of PA0720 by stop codon (TAG)	12.7	55.2	4.3	34.2
56	Pa strain Pa124	CP021774.1	309	13	8	9	3	1	17	15	18.6Kb	intact	107	11	4770347-4788965	PHAGE–Pseudo–Pf1–NC–001331(10)	DR	I	8.3	60.8		no	no CD	no		11.0	55.7	2.7	24.2
57	Pa strain Pa58	CP021775.1	308	13	4	5	5	12	7	15	17.1Kb	intact	107	18	5532813-5549984	PHAGE–Pseudo–Pf1–NC–001331(10)	tRNA-Gly	I	9.1	61.6		no	Arc/mnt	yes		17.2	56.2	8.1	47.0
	Pa strain Pa58	CP021775.1	308	13	4	5	5	12	7	15	12.5Kb	intact	117	12	6189095-6201685	PHAGE–Pseudo–Pf1–NC–001331(11)	tRNA-Met	I	8.3	61.2	15	type 2	DUF5447	no	disruption of PA0724 by stop codon (TGA)	12.7	55.2	4.3	34.2
58	Pa strain Pa84	CP021999.1	-	11	5	37	3	1	60	182	13.4Kb	intact	107	12	4926130-4939591	PHAGE–Pseudo–Pf1–NC–001331(10)	tRNA-Gly	I	8.4	61.8	14	no	Arc/mnt	no		13.5	55	5.1	37.6
59	Pa strain Pa127	CP022000.1	309	13	8	9	3	1	17	15	18.6Kb	intact	107	11	4900838-4919456	PHAGE–Pseudo–Pf1–NC–001331(10)	DR	I	8.2	60.9	14	no	no CD	no		11.0	55.7	2.7	25.0
60	Pa strain Pa1207	CP022001.1	155	28	5	36	3	3	13	7	17.2Kb	intact	96	16	2191788-2209066	PHAGE–Pseudo–Pf1–NC–001331(9)	tRNA-Gly	II	8.2	60.8	14	no	Arc/mnt, DUF5447	no		13.5	56.9	5.2	38.9
61	Pa strain Pa1242	CP022002.1	277	39	5	9	11	27	5	2	17.2Kb	intact	107	19	2166528-2183783	PHAGE–Pseudo–Pf1–NC–001331(10)	tRNA-Gly	II	8.1	61.1	15	type 1	DUF5447	no		12.4	55.4	4.3	34.6
62	Pa strain Ocean-1175	CP022525.1	316	13	8	9	3	1	6	9	24.5Kb	intact	96	15	3538559-3563124	PHAGE–Pseudo–Pf1–NC–001331(9)	tRNA-Gly	II	8.2	61.3	14	no	Arc/mnt	no		11.8	57.3	3.6	30.1
	Pa strain Ocean-1175	CP022525.1	316	13	8	9	3	1	6	9	16.3Kb	intact	128	23	2934416-2950732	PHAGE–Pseudo–Pf1–NC–001331(12)	tRNA-Met	I	9.0	62.2	16	type 1	DUF5447	yes		15.5	55.5	6.5	42.2
63	Pa strain Ocean-1155	CP022526.1	316	13	8	9	3	1	6	9	24.5Kb	intact	96	15	2051456-2076021	PHAGE–Pseudo–Pf1–NC–001331(9)	tRNA-Gly	II	8.2	61.3	14	no	Arc/mnt	no		11.8	57.3	3.6	30.1
	Pa strain Ocean-1155	CP022526.1	316	13	8	9	3	1	6	9	16.3Kb	intact	128	23	2663863-2680176	PHAGE–Pseudo–Pf1–NC–001331(12)	tRNA-Met	I	9.0	62.2	16	type 1	DUF5447	yes		15.5	55.5	6.5	42.2
64	Pa strain CCUG 70744	CP023255.1	395	6	5	1	1	1	12	1	14.4Kb	intact	107	12	3119469-3133929	PHAGE–Pseudo–Pf1–NC–001331(10)	tRNA-Gly	I	9.1	61.7	15	no	Arc/mnt	yes		14.5	55.9	5.4	37.3
	Pa strain CCUG 70744	CP023255.1	395	6	5	1	1	1	12	1	25.2Kb	intact	117	18	3673134-3698374	PHAGE–Pseudo–Pf1–NC–001331(11)	tRNA-Met	I	8.3	62	15	type 2	DUF5447	no		11.7	56.8	3.4	29.2
65	Pa strain 12939	CP024477.1	1420	5	163	25	5	1	20	125	11.1Kb	questionable	85	9	4889671-4900780	PHAGE–Pseudo–Pf1–NC–001331(8)	tRNA-Gly	I	8.4	59.9	15	type 1	DUF5447	no	lack of orf89, an insertion of another variant of orf encoding proteins with Arc/mnt domain	11.1	58	2.7	24.5
	Pa strain 12939	CP024477.1	1420	5	163	25	5	1	20	125	14Kb	questionable	85	11	5506800-5520815	PHAGE–Pseudo–Pf1–NC–001331(8)	tRNA-Met	I	8.3	62	15	type 2	no CD	no		14.1	55.9	5.8	41.1
66	Pa strain PB369	CP025049.1	319	5	4	3	3	1	11	8	11.9Kb	intact	117	16	4338247-4350225	PHAGE–Pseudo–Pf1–NC–001331(11)	tRNA-Gly	I	8.3	61.5	15	type 1	DUF5447	no		12.0	56.4	3.7	30.5
	Pa strain PB369	CP025049.1	319	5	4	3	3	1	11	8	8.2Kb	questionable	75	17	5428116-5436391	PHAGE–Pseudo–Pf1–NC–001331(7)	tRNA-Met	I	–	0.0	–	–	–	–	–	–	0.0	0.0	
67	Pa strain PB368	CP025050.1	319	5	4	3	3	1	11	8	11.9Kb	intact	117	16	4370975-4382953	PHAGE–Pseudo–Pf1–NC–001331(11)	tRNA-Gly	I	8.3	61.5	15	type 1	DUF5447	no		12.0	56.4	#ARG!	#ARG!
	Pa strain PB368	CP025050.1	319	5	4	3	3	1	11	8	8.2Kb	questionable	75	17	5539347-5547622	PHAGE–Pseudo–Pf1–NC–001331(7)	tRNA-Met	I	–	0.0	–	–	–	–	–	–	0.0	0.0	
68	Pa strain AR–0360	CP027165.1	1712	16	5	30	11	4	31	7	12.5Kb	questionable	85	9	3576086-3588613	PHAGE–Pseudo–Pf1–NC–001331(8)	tRNA-Gly	I	8.3	62.1	15	type 1	DUF5447	no		12.6	56.1	#ARG!	#ARG!
	Pa strain AR–0360	CP027165.1	1712	16	5	30	11	4	31	7	11.9Kb	questionable	75	12	4201378-4213319	PHAGE–Pseudo–Pf1–NC–001331(7)	tRNA-Met	II	8.2	60.8	15	type 1	DUF5447	no		12.0	56	#ARG!	#ARG!
69	Pa strain AR–0353	CP027172.1	308	13	4	5	5	12	7	15	24Kb	questionable	75	10	1804529-1828528	PHAGE–Pseudo–Pf1–NC–001331(7)	tRNA-Gly	I	9.1	61.6	15	no	Arc/mnt	yes	disruption of PA0728 by stop codon (TAG)	15.6	56	#ARG!	#ARG!
	Pa strain AR–0353	CP027172.1	308	13	4	5	5	12	7	15	8.3Kb	questionable	85	10	1195588-1203937	PHAGE–Pseudo–Pf1–NC–001331(8)	tRNA-Met	I	8.3	61.2	15	type 2	DUF5447	no		12.7	55.2	#ARG!	#ARG!
70	Pa strain WCHPA075019	CP028584.2	277	39	5	9	11	27	5	2	12.3Kb	intact	107	12	5198198-5210554	PHAGE–Pseudo–Pf1–NC–001331(10)	tRNA-Gly	II	8.1	61.1	15	type 1	DUF5447	no		12.4	55.4	#ARG!	#ARG!
71	Pa strain JB2	CP028917.1	296	44	8	5	3	15	6	26	16.4Kb	incomplete	30	9	2142112-2158591	PHAGE–Pseudo–Pf1–NC–001331(5)	tRNA-Gly	II	8.1	61.1	15	type 2	DUF5447	no		12.8	55.5	#ARG!	#ARG!
	Pa strain JB2	CP028917.1	296	44	8	5	3	15	6	26	11.1Kb	intact	96	11	1533237-1544350	PHAGE–Pseudo–Pf1–NC–001331(9)	tRNA-Met	I	8.4	61.4	15	type 1	DUF5447	no		13.2	55.6	#ARG!	#ARG!
72	Pa strain AR445	CP029088.1	111	17	5	5	4	4	4	3	12.1Kb	questionable	85	14	6032512-6044696	PHAGE–Pseudo–Pf1–NC–001331(8)	tRNA-Met	I	8.3	62.1	15	type 2	DUF5447	no		11.1	58.2	#ARG!	#ARG!
73	Pa strain AR442	CP029090.1	395	6	5	1	1	1	12	1	14.4Kb	questionable	75	9	4518868-4533328	PHAGE–Pseudo–Pf1–NC–001331(7)	tRNA-Gly	I	9.1	61.7	15	no	Arc/mnt	yes		14.5	55.9	#ARG!	#ARG!
74	Pa strain AR439	CP029097.1	179	36	27	28	3	4	13	7	14.5Kb	questionable	75	16	1291429-1305940	PHAGE–Pseudo–Pf1–NC–001331(7)	tRNA-Met	II	8.1	61.1	15	type 1	DUF5447	no	disruption of PA0725 by stop codon (TGA)	12.0	56.2	#ARG!	#ARG!
75	Pa strain 24Pae112	CP029605.1	235	38	11	3	13	1	2	4	12.6Kb	intact	107	15	5346122-5358785	PHAGE–Pseudo–Pf1–NC–001331(10)	tRNA-Gly	I	8.3	61.7	15	type 1	DUF5447	no		11.5	56.7	#ARG!	#ARG!
76	Pa strain AR–0446	CP029660.1	-	89	30	64	26	4	24	32	31.5Kb	questionable	85	15	3425184-3456773	PHAGE–Pseudo–Pf1–NC–001331(8)	tmRNA	I	8.3	61.5	15	type 1	DUF5447	no		10.8	58.3	#ARG!	#ARG!
77	Pa strain AR–458	CP030327.1	274	23	5	11	7	1	12	7	24.6Kb	questionable	85	14	4607527-4632206	PHAGE–Pseudo–Pf1–NC–001331(8)	tRNA-Gly	I	7.8	61.7	15	type 1	DUF5447	no		10.3	61.8	#ARG!	#ARG!
78	Pa strain AR–455	CP030328.1	298	18	4	13	3	1	17	13	11.6Kb	incomplete	20	11	6165560-6177233	PHAGE–Pseudo–Pf1–NC–001331(6)	tRNA-Gly	II	8.1	61.1	15	type 2	DUF5447	no		11.7	56.6	#ARG!	#ARG!
79	Pa strain AR–460	CP030351.1	-	16	5	3	72	4	13	7	11Kb	incomplete	30	11	3076140-3087214	PHAGE–Pseudo–Pf1–NC–001331(5)	tRNA-Gly	II	8.1	60.8	15	type 2	DUF5447	no		11.1	56.5	#ARG!	#ARG!
80	Pa strain Y31	CP030910.1	-	1	5	11	5	4	15	~260	17Kb	questionable	85	14	5361006-5378043	PHAGE–Pseudo–Pf1–NC–001331(8)	DR	II	8.2	59.7	14	no	no CD	no		9.4	57.1	#ARG!	#ARG!
	Pa strain Y31	CP030910.1	-	1	5	11	5	4	15	~260	7.6Kb	questionable	85	11	796366-804017	PHAGE–Pseudo–Pf1–NC–001331(8)	tRNA-Gly	II	8.3	60.6	14	no	Arc/mnt	no		11.9	55.3	#ARG!	#ARG!
	Pa strain Y31	CP030910.1	-	1	5	11	5	4	15	~260	12.4Kb	intact	96	16	5756863-5769280	PHAGE–Pseudo–Pf1–NC–001331(9)	tRNA-Met	II	8.1	61.1	15	type 1	DUF5447	no		12.0	56.2	#ARG!	#ARG!
81	Pa strain Y71	CP030911.1	245	39	6	12	11	3	15	2	21.7Kb	intact	96	12	5929987-5951694	PHAGE–Pseudo–Pf1–NC–001331(9)	tRNA-Sec	II	8.4	60.6	15	type 1	DUF5447	no		11.4	55.6	#ARG!	#ARG!
82	Pa strain PABL017	CP031660.1	2167	11	143	1	7	3	94	26	18Kb	questionable	85	17	4327695-4345742	PHAGE–Pseudo–Pf1–NC–001331(8)	DR	II	8.1	60.2	15	type 2	DUF5447	no		10.4	56.1	#ARG!	#ARG!
83	Pa strain PAO1161	CP032126.1	549	7	5	12	3	4	1	7	12.3Kb	intact	117	16	4717664-4729990	PHAGE–Pseudo–Pf1–NC–001331(11)	tRNA-Gly	I	8.2	62.1	15	type 1	DUF5447	no		12.4	56.2	#ARG!	#ARG!
	Pa strain PAO1161	CP032126.1	549	7	5	12	3	4	1	7	12.8Kb	intact	128	20	5349640-5362440	PHAGE–Pseudo–Pf1–NC–001331(12)	tRNA-Met	I	8.3	61.9	15	type 1	DUF5447	no		12.1	56	#ARG!	#ARG!
84	Pa strain PA34	CP032552.1	1284	32	8	5	3	5	6	26	10.9Kb	intact	117	12	4895159-4906094	PHAGE–Pseudo–Pf1–NC–001331(11)	tmRNA	I	8.4	61.5	15	type 1	DUF5447	no		11.0	57.4	#ARG!	#ARG!
85	Pa strain BA7823	CP032569.1	-	2	4	5	3	1	174?	~201	12.6Kb	intact	107	11	4686669-4699320	PHAGE–Pseudo–Pf1–NC–001331(10)	tRNA-Gly	I	8.4	61.6	14	no	Arc/mnt	no	disruption of PA0728 by stop codon (TGA)	12.7	56.8	#ARG!	#ARG!
86	Pa strain BA15561	CP033432.1	357	2	4	5	3	1	6	11	7.8Kb	intact	96	11	6545983-6553793	PHAGE–Pseudo–Pf1–NC–001331(9)	tRNA-Gly	I	8.4	61.6	14	no	Arc/mnt	no	disruption of PA0724 by stop codon (TAG)	12.7	56.8	#ARG!	#ARG!
87	Pa strain SP4528	CP033439.1	357	2	4	5	3	1	6	11	13.6Kb	intact	107	13	1508597-1522217	PHAGE–Pseudo–Pf1–NC–001331(10)	tRNA-Gly	I	8.3	61.3	14	no	DUF5447	no	lack of orf89, an insertion of another variant of orf encoding proteins with Arc/mnt domain	10.3	58.2	#ARG!	#ARG!
88	Pa strain H26027	CP033684.1	17	11	5	1	7	9	4	7	17.8Kb	questionable	85	17	4721589-4739478	PHAGE–Pseudo–Pf1–NC–001331(8)	DR	II	8.2	60.6	14	no	no CD	no		10.3	57.2	#ARG!	#ARG!
89	Pa strain H26023	CP033685.1	253	4	4	16	12	1	6	3	18.2Kb	intact	117	21	4594215-4612444	PHAGE–Pseudo–Pf1–NC–001331(11)	DR	I	8.4	61.2	15	type 1	DUF5447	no		10.6	57.3	#ARG!	#ARG!
	Pa strain H26023	CP033685.1	253	4	4	16	12	1	6	3	26.7Kb	intact	128	20	5643266-5670047	PHAGE–Pseudo–Pf1–NC–001331(12)	tRNA-Met	I	8.4	61.8	15	type 1	DUF5447	no		13.2	55.7	#ARG!	#ARG!
90	Pa strain H25883	CP033686.1	1076	5	4	57	62	1	1	26	11.8Kb	intact	96	11	5040815-5052677	PHAGE–Pseudo–Pf1–NC–001331(9)	tRNA-Gly	II	8.3	61	14	no	Arc/mnt	no		11.9	56.7	#ARG!	#ARG!
	Pa strain H25883	CP033686.1	1076	5	4	57	62	1	1	26	13.4Kb	intact	128	21	5604580-5618063	PHAGE–Pseudo–Pf1–NC–001331(12)	tRNA-Met	I	8.4	61.4	15	type 1	DUF5447	no		13.2	55.5	#ARG!	#ARG!
91	Pa strain FDAARGOS–532	CP033771.1	179	36	27	28	3	4	13	7	16.2Kb	intact	107	22	3599080-3615327	PHAGE–Pseudo–Pf1–NC–001331(10)	tRNA-Met	II	8.1	61.1	15	type 1	DUF5447	no	disruption of PA0725 by stop codon (TGA)	13.1	56.7	#ARG!	#ARG!
92	Pa strain FDAARGOS–505	CP033832.1	179	36	27	28	3	4	13	7	15.1Kb	intact	107	21	301134-316278	PHAGE–Pseudo–Pf1–NC–001331(10)	tRNA-Met	II	8.1	61.1	16	type 1	DUF5447	no	disruption of PA0725 by stop codon (TGA)	12.0	56.2	#ARG!	#ARG!
93	Pa strain IMP-13	CP034354.1	621	15	5	20	5	1	4	25	15Kb	intact	117	16	1430598-1445603	PHAGE–Pseudo–Pf1–NC–001331(11)	tRNA-Gly	II	8.3	61.8	15	type 1	DUF5447	no		13.8	54	#ARG!	#ARG!
94	Pa strain B41226	CP034368.1	357	2	4	5	3	1	6	11	13.6Kb	intact	107	13	1040629-1054249	PHAGE–Pseudo–Pf1–NC–001331(10)	tRNA-Gly	I	8.3	61.3	14	no	DUF5447	no	disruption of PA0724 by stop codon (TGA); lack of orf89, an insertion of another variant of orf encoding proteins with Arc/mnt domain	10.3	58.3	#ARG!	#ARG!
95	Pa strain SP4371	CP034369.1	357	2	4	5	3	1	6	11	10.2Kb	intact	107	13	4339041-4349287	PHAGE–Pseudo–Pf1–NC–001331(10)	tRNA-Gly	I	8.3	61.3	14	no	DUF5447	no	disruption of PA0724 by stop codon (TGA); lack of orf89, an insertion of another variant of orf encoding proteins with Arc/mnt domain	10.3	58.3	#ARG!	#ARG!
96	Pa strain SP4527	CP034409.1	357	2	4	5	3	1	6	11	10.2Kb	intact	107	13	3435411-3445663	PHAGE–Pseudo–Pf1–NC–001331(10)	tRNA-Gly	I	8.3	61.3	14	no	DUF5447	no	lack of orf89, an insertion of another variant of orf encoding proteins with Arc/mnt domain	10.3	58.3	#ARG!	#ARG!
97	Pa strain GIMC5015:PAKB6	CP034429.1	549	7	5	12	3	4	1	7	12.3Kb	questionable	75	8	4705795-4718128	PHAGE–Pseudo–Pf1–NC–001331(7)	tRNA-Met	I	8.3	61.9	15	type 1	DUF5447	no		12.2	55.9	#ARG!	#ARG!
	Pa strain GIMC5015:PAKB6	CP034429.1	549	7	5	12	3	4	1	7	12Kb	questionable	75	8	5229750-5241829	PHAGE–Pseudo–Pf1–NC–001331(7)	tRNA-Gly	I	8.2	62	15	type 1	DUF5447	no		12.4	56.2	#ARG!	#ARG!
98	Pa strain SP2230	CP034434.1	357	2	4	5	3	1	6	11	14.7Kb	intact	107	16	4131114-4145911	PHAGE–Pseudo–Pf1–NC–001331(10)	tRNA-Gly	II	8.4	61.6	14	no	Arc/mnt	no		12.7	56.7	#ARG!	#ARG!
99	Pa strain B14130	CP034435.1	357	2	4	5	3	1	6	11	7.8Kb	intact	107	11	1451507-1459318	PHAGE–Pseudo–Pf1–NC–001331(10)	tRNA-Gly	I	8.4	61.6	14	no	Arc/mnt	no	disruption of PA0724 by stop codon (TGA)	12.7	56.8	#ARG!	#ARG!
100	Pa strain B17932	CP034436.1	357	2	4	5	3	1	6	11	7.8Kb	intact	107	11	1302774-1310586	PHAGE–Pseudo–Pf1–NC–001331(10)	tRNA-Gly	I	8.4	61.6	14	no	Arc/mnt	no		12.7	56.8	#ARG!	#ARG!
101	Pa strain AES1M	CP037925.1	649	11	84	11	3	4	4	7	7.4Kb	questionable	85	12	1249705-1257179	PHAGE–Pseudo–Pf1–NC–001331(8)	tRNA-Gly	II	8.2	60.8	15	type 1	DUF5447	no	lack of orf89, an insertion of another variant of orf encoding proteins with Arc/mnt domain	12.5	55.8	#ARG!	#ARG!
102	Pa strain AES1R	CP037926.1	649	11	84	11	3	4	4	7	12.4Kb	questionable	85	12	4819385-4831813	PHAGE–Pseudo–Pf1–NC–001331(8)	tRNA-Gly	II	8.2	60.8	15	type 1	DUF5447	no	lack of orf89, an insertion of another variant of orf encoding proteins with Arc/mnt domain	12.5	55.8	#ARG!	#ARG!
103	Pa strain PABL048	CP039293.1	298	18	4	13	3	1	17	13	31Kb	intact	96	20	5164270-5195348	PHAGE–Pseudo–Pf1–NC–001331(9)	tRNA-Gly	II	8.1	61.1	15	type 2	DUF5447	no		11.7	56.6	#ARG!	#ARG!
104	Pa strain PA298	CP040127.1	277	39	5	9	11	27	5	2	12.3Kb	intact	107	12	4882179-4894535	PHAGE–Pseudo–Pf1–NC–001331(10)	tRNA-Gly	II	8.1	61.1	15	type 1	DUF5447	no		12.4	55.4	#ARG!	#ARG!
105	Pa LESB58	FM209186.1	146	6	5	11	3	4	23	1	18.1Kb	intact	107	12	4545082-4563204	PHAGE–Pseudo–Pf1–NC–001331(10)	DR	II	8.2	60	15	type 1	DUF5447	no		10.5	56.1	#ARG!	#ARG!
106	Pa NCTC10332	LN831024.1	-	175	5	1	11	3	6	7	19.1Kb	intact	96	15	4217985-4237181	PHAGE–Pseudo–Pf1–NC–001331(9)	DR	I	8.3	61.1	14	no	no CD	no		10.8	57.4	#ARG!	#ARG!
	Pa NCTC10332	LN831024.1	-	175	5	1	11	3	6	7	6.5Kb	questionable	75	10	5427617-5434139	PHAGE–Pseudo–Pf1–NC–001331(7)	tRNA-Sec	II	8.1	60.6	15	type 2	DUF5447	no		9.5	58	#ARG!	#ARG!
107	Pa DK1	LN870292.1	387	28	5	11	11	4	12	3	7.6Kb	questionable	85	10	4564903-4572525	PHAGE–Pseudo–Pf1–NC–001331(8)	tmRNA	I	8.3	61	15	type 2	DUF5447	no		11.5	58.3	#ARG!	#ARG!
108	Pa PAO1OR	LN871187.1	549	7	5	12	3	4	1	7	12.3Kb	intact	107	13	4718101-4730483	PHAGE–Pseudo–Pf1–NC–001331(10)	tRNA-Gly	I	8.2	62	15	type 1	DUF5447	no		12.4	56.3	#ARG!	#ARG!
	Pa PAO1OR	LN871187.1	549	7	5	12	3	4	1	7	12.5Kb	intact	117	16	5242051-5254620	PHAGE–Pseudo–Pf1–NC–001331(11)	tRNA-Met	I	8.3	61.9	15	type 1	DUF5447	no		12.1	56	#ARG!	#ARG!
109	Pa isolate paerg002	LR130527.1	262	17	5	1	3	4	15	7	12.9Kb	incomplete	30	12	2058664-2071616	PHAGE–Pseudo–Pf1–NC–001331(6)	tRNA-Met	II	8.8	60.5	17	type 2	no CD	yes	disruption of PA0724 and dcm by stop codons (TGA)	13.0	54.9	#ARG!	#ARG!
	Pa isolate paerg002	LR130527.1	262	17	5	1	3	4	15	7	21Kb	questionable	85	13	2205331-2226417	PHAGE–Pseudo–Pf1–NC–001331(8)	tRNA-Sec	II	8.2	61	14	no	no CD	no		10.7	56.4	#ARG!	#ARG!
110	Pa isolate paerg003	LR130530.1	262	17	5	1	3	4	15	7	12.9Kb	questionable	75	12	5378063-5391022	PHAGE–Pseudo–Pf1–NC–001331(7)	tRNA-Met	II	8.8	60.5	15	type 2	no CD	yes	disruption of dcm by stop codons (TGA)	13.0	54.9	#ARG!	#ARG!
	Pa isolate paerg003	LR130530.1	262	17	5	1	3	4	15	7	21Kb	questionable	75	12	5524737-5545820	PHAGE–Pseudo–Pf1–NC–001331(7)	tRNA-Sec	II	8.2	61.1		no	no CD	no		10.7	56.5	#ARG!	#ARG!
111	Pa isolate paerg004	LR130531.1**	262	17	5	1	3	4	15	7	–	–	–	–	**2695745-2708774**	–	tRNA-Met	II	8.8	60.6	15	type 2	DUF5447	yes	disruption of dcm by stop codons (TGA)	13.0	55	#ARG!	#ARG!
	Pa isolate paerg004	LR130531.1	262	17	5	1	3	4	15	7	21Kb	questionable	85	13	2842446-2863533	PHAGE–Pseudo–Pf1–NC–001331(8)	tRNA-Sec	II	8.2	61.1	14	no	no CD	no		10.7	56.4	#ARG!	#ARG!
112	Pa isolate paerg009	LR130533.1	309	13	8	9	3	1	17	15	18.6Kb	intact	107	12	1167727-1186345	PHAGE–Pseudo–Pf1–NC–001331(10)	DR	I	8.3	60.8	14	no	no CD	no		11.0	55.7	#ARG!	#ARG!
113	Pa isolate paerg005	LR130534.1	309	13	8	9	3	1	17	15	18.6Kb	intact	107	12	4776091-4794709	PHAGE–Pseudo–Pf1–NC–001331(10)	DR	I	8.3	60.8	14	no	no CD	no		11.0	55.7	#ARG!	#ARG!
114	Pa isolate paerg011	LR130535.1	262	17	5	1	3	4	15	7	12.9Kb	questionable	75	12	5378196-5391157	PHAGE–Pseudo–Pf1–NC–001331(7)	tRNA-Met	II	8.8	60.6	15	type 2	no CD	yes	disruption of PA0724 and dcm by stop codons (TGA)	13.0	55	#ARG!	#ARG!
	Pa isolate paerg011	LR130535.1	262	17	5	1	3	4	15	7	21Kb	questionable	85	13	5524875-5545960	PHAGE–Pseudo–Pf1–NC–001331(8)	tRNA-Sec	II	8.2	61	14	no	no CD	no		10.7	56.4	#ARG!	#ARG!
115	Pa isolate paerg010	LR130536.1	262	17	5	1	3	4	15	7	12.9Kb	questionable	75	11	5378052-5391011	PHAGE–Pseudo–Pf1–NC–001331(7)	tRNA-Met	II	8.8	60.5	15	type 2	no CD	no	disruption of PA0724 and dcm by stop codons (TGA)	13.0	54.9	#ARG!	#ARG!
	Pa isolate paerg010	LR130536.1	262	17	5	1	3	4	15	7	21Kb	questionable	85	13	5524727-5545817	PHAGE–Pseudo–Pf1–NC–001331(8)	tRNA-Sec	II	8.2	61.1	14	no	no CD	no		10.8	56.4	#ARG!	#ARG!
116	Pa isolate paerg012	LR130537.1	262	17	5	1	3	4	15	7	12.9Kb	questionable	75	12	5378076-5391035	PHAGE–Pseudo–Pf1–NC–001331(7)	tRNA-Met	II	8.8	60.6	15	type 2	no CD	yes	disruption of dcm by stop codon (TGA)	13.0	55	#ARG!	#ARG!
	Pa isolate paerg012	LR130537.1	262	17	5	1	3	4	15	7	21Kb	questionable	85	13	5524752-5545843	PHAGE–Pseudo–Pf1–NC–001331(8)	tRNA-Sec	II	8.2	61.1	15	no	no CD	no	disruption of PA0724 by stop codon (TGA)	10.8	56.4	#ARG!	#ARG!
117	Pa strain NCTC11445	LR134308.1	-	11	5	12	~11	4	4	20	8Kb	intact	96	12	395349-403406	PHAGE–Pseudo–Pf1–NC–001331(9)	tRNA-Gly	I	8.4	61	14	no	Arc/mnt	no		11.1	57.1	#ARG!	#ARG!
118	Pa strain NCTC12903	LR134309.1	155	28	5	36	3	3	13	7	14.6Kb	intact	96	18	5787601-5802274	PHAGE–Pseudo–Pf1–NC–001331(9)	tRNA-Met	II	8.1	61.1	16	type 1	DUF5447	no	disruption of PA0725 by stop codon (TGA)	12.0	56.2	#ARG!	#ARG!
	Pa strain NCTC12903	LR134309.1**															tRNA-Gly	II	8.2	60.8	14	no	Arc/mnt, DUF5447	no		12.1	56.6	#ARG!	#ARG!
119	Pa strain NCTC10728	LR134342.1	1750	6	5	5	5	52	4	7	12Kb	intact	107	12	1932684-1944778	PHAGE–Pseudo–Pf1–NC–001331(10)	tRNA-Met	I	8.4	61.9	15	type 1	DUF5447	no		12.2	56.1	#ARG!	#ARG!
120	Pa isolate PA14Or	LT608330.1	253	4	4	16	12	1	6	3	18.2Kb	intact	117	19	4348175-4366403	PHAGE–Pseudo–Pf1–NC–001331(11)	DR	I	8.4	61.2	15	type 1	DUF5447	no		10.6	57.3	#ARG!	#ARG!
121	Pa isolate PcyII-1	LT673656.1	1233	17	5	11	3	4	6	7	11.9Kb	questionable	85	13	4713278-4725253	PHAGE–Pseudo–Pf1–NC–001331(8)	tRNA-Gly	II	8.9	61.1	16	type 1	DUF5447	yes	lack of orf89, an insertion of another variant of orf encoding proteins with Arc/mnt domain	12.0	55.4	#ARG!	#ARG!
	Pa isolate PcyII-1	LT673656.1	1233	17	5	11	3	4	6	7	17.7Kb	intact	96	13	5239282-5256988	PHAGE–Pseudo–Pf1–NC–001331(9)	tRNA-Met	I	9.0	61.8	16	type 1	DUF5447	yes		17.8	54.4	#ARG!	#ARG!
122	Pa C-NN2	LT883143.1	17	11	5	1	7	9	4	7	17.8Kb	questionable	85	17	4464501-4482390	PHAGE–Pseudo–Pf1–NC–001331(8)	DR	II	8.2	60.6	14	no	no CD	no		10.3	57.2	#ARG!	#ARG!
	Pa C-NN2	LT883143.1	17	11	5	1	7	9	4	7	18.6Kb	intact	117	20	5855586-5874193	PHAGE–Pseudo–Pf1–NC–001331(11)	tRNA-Met	I	8.4	61.5	15	type 1	DUF5447	no		13.9	55.9	#ARG!	#ARG!
123	Pa isolate RW109	LT969520.1	111	17	5	5	4	4	4	3	18Kb	questionable	85	11	4637833-4655921	PHAGE–Pseudo–Pf1–NC–001331(8)	DR	II	8.1	60.5	14	no	no CD	no		10.5	56.6	#ARG!	#ARG!
124	Pa strain FDAARGOS–570	CP033835.1	244	17	5	12	3	14	4	7	–	–	–	–	–		tRNA-Gly	I	8.5	61.3	15	type 1	DUF5447	no	lack of orf89, an insertion of another variant of orf encoding proteins with Arc/mnt domain	9.8	58.5	#ARG!	#ARG!
125	PRD-10	CP039749.1	252	6	28	4	3	3	4	7	12.1Kb	intact	117	20	5019226-5031392	PHAGE_Pseudo_Pf1_NC_001331(11)	tRNA-Met	II	8.2	60.6	15	type 1	DUF5447	no		11.3	56.2	#ARG!	#ARG!
	PRD-10	CP039749.1	252	6	28	4	3	3	4	7	7.4Kb	intact	96	11	5176958-5184445 info_outlir	PHAGE_Pseudo_Pf1_NC_001331(9)	tRNA-Sec	II	8.3	60.4	15	type 2	DUF5447	no		9.9	57.4	#ARG!	#ARG!
126	NCTC13359	LR590473.1	252	6	28	4	3	3	4	7	11.2Kb	intact	107	15	6130145-6141407 info_outlir	PHAGE_Pseudo_Pf1_NC_001331(10)	tRNA-Met	II	8.2	60.2	15	type 1	DUF5447	no		11.3	56.2	#ARG!	#ARG!
	NCTC13359	LR590473.1	252	6	28	4	3	3	4	7	6.5Kb	questionable	75	10	6288931-6295441 info_outlir	PHAGE_Pseudo_Pf1_NC_001331(7)	tRNA-Sec	II	8.3	60.4	15	type 2	DUF5447	no		9.9	57.4	#ARG!	#ARG!
127	Pa NCGM2.S1	AP012280.1	235	38	11	3	13	1	2	4	–	–	–	–	–	–	– –	–	–	–	–	–	–	–	–	–	–	–	–
128	strain: NCGM 1900	AP014622.1	235	38	11	3	13	1	2	4	–	–	–	–	–	–	– –	–	–	–	–	–	–	–	–	–	–	–	–
129	strain: NCGM 1984	AP014646.1	235	38	11	3	13	1	2	4	–	–	–	–	–	–	– –	–	–	–	–	–	–	–	–	–	–	–	–
130	strain: IOMTU 133	AP017302.1	1047	18	8	5	5	1	6	4	–	–	–	–	–	–	– –	–	–	–	–	–	–	–	–	–	–	–	–
131	Pa DK2	CP003149.1	386	17	5	11	18	4	10	3	–	–	–	–	–	–	– –	–	–	–	–	–	–	–	–	–	–	–	–
132	Pa PA1	CP004054.2	782	15	3	3	11	1	15	1	–	–	–	–	–	–	– –	–	–	–	–	–	–	–	–	–	–	–	–
133	Pa PA1R	CP004055.1	-	15	3	3	11	1	15	1	–	–	–	–	–	–	– –	–	–	–	–	–	–	–	–	–	–	–	–
134	Pa RP73	CP006245.1	198	11	5	11	11	3	27	7	–	–	–	–	–	–	– –	–	–	–	–	–	–	–	–	–	–	–	–
135	Pa VRFPA04	CP008739.2	-	32	13	24	13	1	6	25	–	–	–	–	–	–	– –	–	–	–	–	–	–	–	–	–	–	–	–
136	Pa strain F23197	CP008856.2	1295	11	5	124	67	4	115	3	–	–	–	–	–	–	– –	–	–	–	–	–	–	–	–	–	–	–	–
137	Pa strain F63912	CP008858.2	198	11	5	11	11	3	27	7	–	–	–	–	–	–	– –	–	–	–	–	–	–	–	–	–	–	–	–
138	Pa strain H47921	CP008861.1	1105	23	5	12	30	1	4	7	–	–	–	–	–	–	– –	–	–	–	–	–	–	–	–	–	–	–	–
139	Pa strain W16407	CP008869.2	244	17	5	12	3	14	4	7	–	–	–	–	–	–	– –	–	–	–	–	–	–	–	–	–	–	–	–
140	Pa strain W45909	CP008871.2	27	6	5	6	7	4	6	7	–	–	–	–	–	–	– –	–	–	–	–	–	–	–	–	–	–	–	–
141	Pa strain PA–D2	CP012578.1	1971	32	190	3	62	8	7	26	–	–	–	–	–	–	– –	–	–	–	–	–	–	–	–	–	–	–	–
142	Pa strain PA–D5	CP012579.1	1971	32	190	3	62	8	7	26	–	–	–	–	–	–	– –	–	–	–	–	–	–	–	–	–	–	–	–
143	Pa strain PA–D9	CP012580.1	1971	32	190	3	62	8	7	26	–	–	–	–	–	–	– –	–	–	–	–	–	–	–	–	–	–	–	–
144	Pa strain PA–D16	CP012581.1	1971	32	190	3	62	8	7	26	–	–	–	–	–	–	– –	–	–	–	–	–	–	–	–	–	–	–	–
145	Pa strain PA–D21	CP012582.1	1971	32	190	3	62	8	7	26	–	–	–	–	–	–	– –	–	–	–	–	–	–	–	–	–	–	–	–
146	Pa strain PA–D22	CP012583.1	1971	32	190	3	62	8	7	26	–	–	–	–	–	–	– –	–	–	–	–	–	–	–	–	–	–	–	–
147	Pa strain PA–D25	CP012584.1	1971	32	190	3	62	8	7	26	–	–	–	–	–	–	– –	–	–	–	–	–	–	–	–	–	–	–	–
148	Pa strain PA–D1	CP012585.1	1971	32	190	3	62	8	7	26	–	–	–	–	–	–	– –	–	–	–	–	–	–	–	–	–	–	–	–
149	Pa strain PA1RG	CP012679.1	782	15	3	3	11	1	15	1	–	–	–	–	–	–	– –	–	–	–	–	–	–	–	–	–	–	–	–
150	Pa strain Cu1510	CP013144.1	137	31	5	57	13	1	40	3	–	–	–	–	–	–	– –	–	–	–	–	–	–	–	–	–	–	–	–
151	Pa strain VA-134	CP013245.1	3090	40	5	36	153	3	7	19	–	–	–	–	–	–	– –	–	–	–	–	–	–	–	–	–	–	–	–
152	Pa strain N17-1	CP014948.1	2362	6	5	1	29	92	4	68	–	–	–	–	–	–	– –	–	–	–	–	–	–	–	–	–	–	–	–
153	Pa strain DN1	CP017099.1	316	13	8	9	3	1	6	9	–	–	–	–	–	–	– –	–	–	–	–	–	–	–	–	–	–	–	–
154	Pa strain PA83	CP017293.1	233	16	5	30	11	4	31	41	–	–	–	–	–	–	– –	–	–	–	–	–	–	–	–	–	–	–	–
155	Pa strain FA-HZ1	CP017353.1	27	6	5	6	7	4	6	7	–	–	–	–	–	–	– –	–	–	–	–	–	–	–	–	–	–	–	–
156	Pa strain L10	CP019338.1	253	4	4	16	12	1	6	3	–	–	–	–	–	–	– –	–	–	–	–	–	–	–	–	–	–	–	–
157	Pa strain E6130952	CP020603.1	235	38	11	3	13	1	2	4	–	–	–	–	–	–	– –	–	–	–	–	–	–	–	–	–	–	–	–
158	Pa strain LW	CP022478.1	1182	5	1	109	54	1	1	47	–	–	–	–	–	–	– –	–	–	–	–	–	–	–	–	–	–	–	–
159	Pa strain PPF-1	CP023316.1	-	13	13	9	3	1	6	9	–	–	–	–	–	–	– –	–	–	–	–	–	–	–	–	–	–	–	–
160	Pa strain PB353	CP025051.1	-	11	62	17	5	2	4	7	–	–	–	–	–	–	– –	–	–	–	–	–	–	–	–	–	–	–	–
161	Pa strain PB354	CP025053.1	-	11	62	17	5	2	4	7	–	–	–	–	–	–	– –	–	–	–	–	–	–	–	–	–	–	–	–
162	Pa strain PB350	CP025055.1	235	38	11	3	13	1	2	4	–	–	–	–	–	–	– –	–	–	–	–	–	–	–	–	–	–	–	–
163	Pa strain PB367	CP025056.1	235	38	11	3	13	1	2	4	–	–	–	–	–	–	– –	–	–	–	–	–	–	–	–	–	–	–	–
164	Pa strain F5677	CP026680.1	111	17	5	5	4	4	4	3	–	–	–	–	–	–	– –	–	–	–	–	–	–	–	–	–	–	–	–
165	Pa strain AR–0357	CP027166.1	235	38	11	3	13	1	2	4	–	–	–	–	–	–	– –	–	–	–	–	–	–	–	–	–	–	–	–
166	Pa strain AR–0356	CP027169.1	1006	87	42	114	37	53	97	129	–	–	–	–	–	–	– –	–	–	–	–	–	–	–	–	–	–	–	–
167	Pa strain AR–0354	CP027171.1	235	38	11	3	13	1	2	4	–	–	–	–	–	–	– –	–	–	–	–	–	–	–	–	–	–	–	–
168	Pa strain AR–0230	CP027174.1	233	16	5	30	11	4	31	41	–	–	–	–	–	–	– –	–	–	–	–	–	–	–	–	–	–	–	–
169	Pa strain AR–0095	CP027538.1	620	9	7	63	13	8	7	8	–	–	–	–	–	–	– –	–	–	–	–	–	–	–	–	–	–	–	–
170	Pa strain MRSN12280	CP028162.1	-	38	~11	3	13	1	2	4	–	–	–	–	–	–	– –	–	–	–	–	–	–	–	–	–	–	–	–
171	Pa strain AR444	CP029089.1	233	16	5	30	11	4	31	41	–	–	–	–	–	–	– –	–	–	–	–	–	–	–	–	–	–	–	–
172	Pa strain AR441	CP029093.1	1006	87	42	114	37	53	97	129	–	–	–	–	–	–	– –	–	–	–	–	–	–	–	–	–	–	–	–
173	Pa strain K34-7	CP029707.1	233	16	5	30	11	4	31	41	–	–	–	–	–	–	– –	–	–	–	–	–	–	–	–	–	–	–	–
174	Pa strain AR–0110	CP029745.1	233	16	5	30	11	4	31	41	–	–	–	–	–	–	– –	–	–	–	–	–	–	–	–	–	–	–	–
175	Pa strain HS9	CP030861.1	27	6	5	6	7	4	6	7	–	–	–	–	–	–	– –	–	–	–	–	–	–	–	–	–	–	–	–
176	Pa strain Y82	CP030912.1	111	17	5	5	4	4	4	3	–	–	–	–	–	–	– –	–	–	–	–	–	–	–	–	–	–	–	–
177	Pa strain Y89	CP030913.1	245	39	6	12	11	3	15	2	–	–	–	–	–	–	– –	–	–	–	–	–	–	–	–	–	–	–	–
178	Pa strain 97	CP031449.2	234	1	5	11	3	4	10	3	–	–	–	–	–	–	– –	–	–	–	–	–	–	–	–	–	–	–	–
179	Pa strain PABL012	CP031659.1	-	11	3	11	3	1	~4	60	–	–	–	–	–	–	– –	–	–	–	–	–	–	–	–	–	–	–	–
180	Pa strain E80	CP031677.1	245	39	6	12	11	3	15	2	–	–	–	–	–	–	– –	–	–	–	–	–	–	–	–	–	–	–	–
181	Pa strain AR–0111	CP032257.1	233	16	5	30	11	4	31	41	–	–	–	–	–	–	– –	–	–	–	–	–	–	–	–	–	–	–	–
182	Pa strain 268	CP032761.1	235	38	11	3	13	1	2	4	–	–	–	–	–	–	– –	–	–	–	–	–	–	–	–	–	–	–	–
183	Pa strain PA-3	CP033084.1	1135	6	8	11	63	4	15	2	–	–	–	–	–	–	– –	–	–	–	–	–	–	–	–	–	–	–	–
184	Pa strain FDAARGOS–571	CP033833.1	235	38	11	3	13	1	2	4	–	–	–	–	–	–	– –	–	–	–	–	–	–	–	–	–	–	–	–
185	Pa strain FDAARGOS–501	CP033843.1	235	38	11	3	13	1	2	4	–	–	–	–	–	–	– –	–	–	–	–	–	–	–	–	–	–	–	–
186	Pa strain 1334/14	CP035739.1	234	1	5	11	3	4	10	3	–	–	–	–	–	–	– –	–	–	–	–	–	–	–	–	–	–	–	–
187	Pa isolate paerg000	LR130528.1	27	6	5	6	7	4	6	7	–	–	–	–	–	–	– –	–	–	–	–	–	–	–	–	–	–	–	–
188	Pa strain NCTC13715	LR134330.1	773	5	4	5	5	5	7	8	–	–	–	–	–	–	– –	–	–	–	–	–	–	–	–	–	–	–	–
189	Pa strain NCTC9433	LS483497.1	1920	27	13	9	156	1	7	192	–	–	–	–	–	–	– –	–	–	–	–	–	–	–	–	–	–	–	–
190	Pa isolate 1	LS998783.1	111	17	5	5	4	4	4	3	–	–	–	–	–	–	– –	–	–	–	–	–	–	–	–	–	–	–	–

*, disrupted ORFs are counted as one ORF

**, Pf prophages not detected by Phaster

Table S2. Characteristics of accessory proteins (n=773) from the Pf prophages.

No.	*P. aeruginosa* genome	Pf prophage	Lineage	Protein	Lenght	Hits	E-Value	sim mean	#GO	GO Ids	GO Names	EC	Enzyme name	InterPro Ids	InterPro GO Ids	InterPro GO names
1	AE004091.2	Pf-tRNA-Gly	I	RNA-directed DNA polymerase	306	20	0	79.15	4	P:GO:0006259; P:GO:0009058; F:GO:0003723; F:GO:0016779	P:DNA metabolic process; P:biosynthetic process; F:RNA binding; F:nucleotidyltransferase activity	EC:2.7.7.49	RNA-directed DNA polymerase	IPR000123 (PRINTS); IPR000477 (PFAM); PTHR34047 (PANTHER); PTHR34047:SF6 (PANTHER); IPR000477 (PROSITE_PROFILES); cd03487 (CDD); SSF56672 (SUPERFAMILY)	P:GO:0006278; F:GO:0003723; F:GO:0003964	P:RNA-dependent DNA biosynthetic process; F:RNA binding; F:RNA-directed DNA polymerase activity
2	AE004091.2	Pf-tRNA-Gly	I	AAA family ATPase	441	20	0	78.07	1	F:GO:0043167	F:ion binding			IPR003593 (SMART); G3DSA:3.40.50.300 (GENE3D); IPR038729 (PFAM); IPR003959 (PFAM); PTHR43581:SF2 (PANTHER); PTHR43581 (PANTHER); IPR027417 (SUPERFAMILY)	F:GO:0005524	F:ATP binding
3	AE004091.2	Pf-tRNA-Gly	I	type II toxin-antitoxin system RelE/ParE family toxin	115	20	2.09E-76	98.57						IPR007712 (PFAM); IPR035093 (G3DSA:3.30.2310.GENE3D)	no GO terms	no GO terms
4	AE004091.2	Pf-tRNA-Gly	I	type II toxin-antitoxin system Phd/YefM family antitoxin	83	20	6.81E-53	96.37						PTHR33713:SF7 (PANTHER); PTHR33713 (PANTHER); IPR036165 (SUPERFAMILY)	no GO terms	no GO terms
5	AE004091.2	Pf-tRNA-Gly	I	cobalamin biosynthesis protein CobN	169	20	2.61E-12	80.06	7	P:GO:0006259; P:GO:0006950; F:GO:0003677; F:GO:0004386; F:GO:0004518; C:GO:0005737; C:GO:0032991	P:DNA metabolic process; P:response to stress; F:DNA binding; F:helicase activity; F:nuclease activity; C:cytoplasm; C:protein-containing complex	EC:3.6.1.3; EC:3.6.1.15	Adenosinetriphosphatase; Nucleoside-triphosphate phosphatase	mobidb-lite (MOBIDB_LITE); mobidb-lite (MOBIDB_LITE); SIGNAL_PEPTIDE_N_REGION (PHOBIUS); SIGNAL_PEPTIDE (PHOBIUS); NON_CYTOPLASMIC_DOMAIN (PHOBIUS); SIGNAL_PEPTIDE_H_REGION (PHOBIUS); SIGNAL_PEPTIDE_C_REGION (PHOBIUS); SignalP-noTM (SIGNALP_EUK)	no GO terms	no GO terms
6	AP014651.1	Pf-tRNA-Gly	I	type II toxin-antitoxin system RelE/ParE family toxin	115	20	1.41E-76	98.87						IPR035093 (G3DSA:3.30.2310.GENE3D); IPR007712 (PFAM)	no GO terms	no GO terms
7	AP014651.1	Pf-tRNA-Gly	I	type II toxin-antitoxin system Phd/YefM family antitoxin	83	20	6.81E-53	96.37						PTHR33713:SF7 (PANTHER); PTHR33713 (PANTHER); IPR036165 (SUPERFAMILY)	no GO terms	no GO terms
8	AP014651.1	Pf-tRNA-Gly	I	Uncharacterised protein	265	20	0	66.81						no IPS match	no IPS match	no IPS match
9	AP014651.1	Pf-tRNA-Gly	I	Uncharacterised protein	242	20	1.94E-177	65.51	1	C:GO:0005575	C:cellular_component			Coil (COILS); Coil (COILS); SIGNAL_PEPTIDE_H_REGION (PHOBIUS); SIGNAL_PEPTIDE_C_REGION (PHOBIUS); SIGNAL_PEPTIDE_N_REGION (PHOBIUS); CYTOPLASMIC_DOMAIN (PHOBIUS); TRANSMEMBRANE (PHOBIUS); NON_CYTOPLASMIC_DOMAIN (PHOBIUS); SIGNAL_PEPTIDE (PHOBIUS); TMhelix (TMHMM); TMhelix (TMHMM)	no GO terms	no GO terms
10	AP014651.1	Pf-tRNA-Gly	I	Uncharacterised protein	256	20	0	85.4	1	C:GO:0005575	C:cellular_component			mobidb-lite (MOBIDB_LITE); mobidb-lite (MOBIDB_LITE); CYTOPLASMIC_DOMAIN (PHOBIUS); NON_CYTOPLASMIC_DOMAIN (PHOBIUS); TRANSMEMBRANE (PHOBIUS); TMhelix (TMHMM)	no GO terms	no GO terms
11	AP014651.1	Pf-tRNA-Gly	I	Uncharacterised protein	94	3	2.60E-63	68.62						no IPS match	no IPS match	no IPS match
12	AP014651.1	Pf-tRNA-Met	I	hypothetical protein	136	20	1.37E-94	99.74	1	C:GO:0005575	C:cellular_component			CYTOPLASMIC_DOMAIN (PHOBIUS); CYTOPLASMIC_DOMAIN (PHOBIUS); TRANSMEMBRANE (PHOBIUS); NON_CYTOPLASMIC_DOMAIN (PHOBIUS); NON_CYTOPLASMIC_DOMAIN (PHOBIUS); TRANSMEMBRANE (PHOBIUS); TRANSMEMBRANE (PHOBIUS); TMhelix (TMHMM); TMhelix (TMHMM)	no GO terms	no GO terms
13	AP014651.1	Pf-tRNA-Met	I	sce7726 family protein	285	20	0	74.17						no IPS match	no IPS match	no IPS match
14	AP014651.1	Pf-tRNA-Met	I	sce7725 family protein	317	20	0	71.61						no IPS match	no IPS match	no IPS match
15	AP014651.1	Pf-tRNA-Met	I	DUF2290 domain-containing protein	224	20	6.70E-165	67.3						IPR018742 (PFAM)	no GO terms	no GO terms
16	AP014651.1	Pf-tRNA-Met	I	Superfamily I DNA and RNA helicases	735	20	0	79.23	4	P:GO:0051276; F:GO:0003677; F:GO:0004386; F:GO:0043167	P:chromosome organization; F:DNA binding; F:helicase activity; F:ion binding	EC:3.6.1.3; EC:3.6.4.12; EC:3.6.1.15	Adenosinetriphosphatase; DNA helicase; Nucleoside-triphosphate phosphatase	IPR027785 (PFAM); G3DSA:3.40.50.300 (GENE3D); G3DSA:3.40.50.300 (GENE3D); IPR000212 (PANTHER); PTHR11070:SF3 (PANTHER); cd18807 (CDD); IPR027417 (SUPERFAMILY)	F:GO:0003677; F:GO:0003678; F:GO:0005524	F:DNA binding; F:DNA helicase activity; F:ATP binding
17	AP014651.1	Pf-tRNA-Met	I	ankyrin repeat domain-containing protein	186	20	8.96E-133	62.69	1	F:GO:0003674	F:molecular_function			IPR002110 (SMART); IPR036770 (G3DSA:1.25.40.GENE3D); IPR020683 (PFAM); IPR025309 (PFAM); PTHR24134 (PANTHER); IPR020683 (PROSITE_PROFILES); IPR002110 (PROSITE_PROFILES); IPR036770 (SUPERFAMILY)	F:GO:0005515	F:protein binding
18	AP014839.2	Pf-tRNA-Met	I	MULTISPECIES: hypothetical protein	136	20	1.03E-93	98.01	1	C:GO:0005575	C:cellular_component			CYTOPLASMIC_DOMAIN (PHOBIUS); TRANSMEMBRANE (PHOBIUS); TRANSMEMBRANE (PHOBIUS); CYTOPLASMIC_DOMAIN (PHOBIUS); NON_CYTOPLASMIC_DOMAIN (PHOBIUS); NON_CYTOPLASMIC_DOMAIN (PHOBIUS); TRANSMEMBRANE (PHOBIUS); TMhelix (TMHMM); TMhelix (TMHMM); TMhelix (TMHMM)	no GO terms	no GO terms
19	AP014839.2	Pf-tRNA-Met	I	hypothetical protein	111	20	3.04E-75	98.47						SIGNAL_PEPTIDE_H_REGION (PHOBIUS); SIGNAL_PEPTIDE_N_REGION (PHOBIUS); SIGNAL_PEPTIDE_C_REGION (PHOBIUS); NON_CYTOPLASMIC_DOMAIN (PHOBIUS); SIGNAL_PEPTIDE (PHOBIUS); SignalP-noTM (SIGNALP_GRAM_NEGATIVE); SignalP-noTM (SIGNALP_EUK); SignalP-TM (SIGNALP_GRAM_POSITIVE)	no GO terms	no GO terms
20	AP014839.2	Pf-tRNA-Met	I	restriction endonuclease	245	20	0	95.39	1	F:GO:0004518	F:nuclease activity			Coil (COILS); IPR003615 (SMART); G3DSA:3.30.40.60 (GENE3D); IPR002711 (PFAM); mobidb-lite (MOBIDB_LITE); mobidb-lite (MOBIDB_LITE); PTHR33877:SF1 (PANTHER); PTHR33877 (PANTHER); IPR003615 (CDD)	F:GO:0003676; F:GO:0004519	F:nucleic acid binding; F:endonuclease activity
21	AP014839.2	Pf-tRNA-Met	I	DNA-processing protein DprA	323	20	0	75.71	1	P:GO:0008150	P:biological_process			G3DSA:3.40.50.450 (GENE3D); IPR003488 (PFAM); IPR003488 (PANTHER); SSF102405 (SUPERFAMILY)	P:GO:0009294	P:DNA mediated transformation
22	AP014839.2	Pf-tRNA-Met	I	hypothetical protein	125	20	3.18E-85	99.04						no IPS match	no IPS match	no IPS match
23	AP014839.2	Pf-tRNA-Met	I	(deoxy)nucleoside triphosphate pyrophosphohydrolase	129	20	6.12E-88	91.12	4	P:GO:0006281; F:GO:0008413	P:DNA repair; F:8-oxo-7,8-dihydroguanosine triphosphate pyrophosphatase activity	EC:3.6.1.55; EC:3.6.1.65; EC:3.6.1.9	8-oxo-dGTP diphosphatase; (d)CTP diphosphatase; Nucleotide diphosphatase	IPR020476 (PRINTS); IPR000086 (PFAM); G3DSA:3.90.79.10 (GENE3D); PTHR47707 (PANTHER); IPR020084 (PROSITE_PATTERNS); IPR000086 (PROSITE_PROFILES); cd03425 (CDD); IPR015797 (SUPERFAMILY)	F:GO:0016787	F:hydrolase activity
24	AP014839.2	Pf-tRNA-Met	I	HAD hydrolase-like protein	411	20	0	73.22	4	P:GO:0034641; P:GO:0044281	P:cellular nitrogen compound metabolic process; P:small molecule metabolic process	EC:3.1.3.18; EC:3.6.4.12	Phosphoglycolate phosphatase; DNA helicase	G3DSA:3.40.50.2020 (GENE3D); IPR023214 (G3DSA:3.40.50.GENE3D); IPR041492 (PFAM); IPR000836 (CDD); cd01427 (CDD); IPR029057 (SUPERFAMILY); IPR036412 (SUPERFAMILY)	P:GO:0009116	P:nucleoside metabolic process
25	CP000438.1	Pf-DR	I	ParA family protein	343	20	0	82.15	4	P:GO:0071941; F:GO:0016491; C:GO:0005622; C:GO:0032991	P:nitrogen cycle metabolic process; F:oxidoreductase activity; C:intracellular; C:protein-containing complex	EC:1.18.6.1; EC:1.18.6	Nitrogenase; Acting on iron-sulfur proteins as donors	Coil (COILS); IPR025669 (PFAM); G3DSA:3.40.50.300 (GENE3D); PTHR13696:SF52 (PANTHER); PTHR13696 (PANTHER); cd02042 (CDD); IPR027417 (SUPERFAMILY)	no GO terms	no GO terms
26	CP000438.1	Pf-DR	I	Uncharacterised protein	175	20	4.41E-125	76.51						IPR041519 (PFAM)	no GO terms	no GO terms
27	CP000744.1	Pf-tRNA-Met	I	type II toxin-antitoxin system VapC family toxin	139	20	1.22E-95	96.53	2	F:GO:0004518; F:GO:0043167	F:nuclease activity; F:ion binding			G3DSA:3.40.50.1010 (GENE3D); IPR002716 (PFAM); PTHR33653:SF1 (PANTHER); PTHR33653 (PANTHER); IPR022907 (HAMAP); cd18731 (CDD); IPR029060 (SUPERFAMILY)	F:GO:0004540	F:ribonuclease activity
28	CP000744.1	Pf-tRNA-Met	I	Arc family DNA-binding protein	81	20	1.01E-49	96.42	3	P:GO:0009058; P:GO:0034641; F:GO:0003677	P:biosynthetic process; P:cellular nitrogen compound metabolic process; F:DNA binding			IPR005569 (PFAM); IPR013321 (G3DSA:1.10.1220.GENE3D); IPR010985 (SUPERFAMILY)	P:GO:0006355; F:GO:0003677	P:regulation of transcription, DNA-templated; F:DNA binding
29	CP000744.1	Pf-tRNA-Met	I	Uncharacterised protein	121	20	9.62E-74	81.34						NON_CYTOPLASMIC_DOMAIN (PHOBIUS); SIGNAL_PEPTIDE_N_REGION (PHOBIUS); SIGNAL_PEPTIDE (PHOBIUS); SIGNAL_PEPTIDE_H_REGION (PHOBIUS); SIGNAL_PEPTIDE_C_REGION (PHOBIUS); SignalP-noTM (SIGNALP_GRAM_NEGATIVE); SignalP-TM (SIGNALP_GRAM_POSITIVE)	no GO terms	no GO terms
30	CP000744.1	Pf-tRNA-Met	I	Uncharacterised protein	221	20	8.48E-161	71.03						no IPS match	no IPS match	no IPS match
31	CP000744.1	Pf-tRNA-Met	I	AAA family ATPase	443	20	0	84.99	4	P:GO:0071941; F:GO:0016491; C:GO:0005622; C:GO:0032991	P:nitrogen cycle metabolic process; F:oxidoreductase activity; C:intracellular; C:protein-containing complex	EC:1.18.6.1; EC:1.18.6	Nitrogenase; Acting on iron-sulfur proteins as donors	G3DSA:3.40.50.300 (GENE3D); IPR025669 (PFAM); mobidb-lite (MOBIDB_LITE); mobidb-lite (MOBIDB_LITE); PTHR13696 (PANTHER); cd02040 (CDD); cd02042 (CDD); IPR027417 (SUPERFAMILY)	no GO terms	no GO terms
32	CP000744.1	Pf-tRNA-Met	I	restriction endonuclease	282	20	0	83.66	8	P:GO:0006265; P:GO:0009307; P:GO:0090305; F:GO:0003677; F:GO:0003916; F:GO:0004519; C:GO:0005694; C:GO:0016021	P:DNA topological change; P:DNA restriction-modification system; P:nucleic acid phosphodiester bond hydrolysis; F:DNA binding; F:DNA topoisomerase activity; F:endonuclease activity; C:chromosome; C:integral component of membrane			IPR007560 (PFAM); IPR011856 (G3DSA:3.40.1350.GENE3D); PTHR30015 (PANTHER); TRANSMEMBRANE (PHOBIUS); CYTOPLASMIC_DOMAIN (PHOBIUS); TRANSMEMBRANE (PHOBIUS); NON_CYTOPLASMIC_DOMAIN (PHOBIUS); CYTOPLASMIC_DOMAIN (PHOBIUS); SSF57783 (SUPERFAMILY); IPR011335 (SUPERFAMILY); TMhelix (TMHMM); TMhelix (TMHMM)	P:GO:0009307; F:GO:0003676; F:GO:0003677; F:GO:0004518; F:GO:0004519	P:DNA restriction-modification system; F:nucleic acid binding; F:DNA binding; F:nuclease activity; F:endonuclease activity
33	CP002496.1	Pf-tRNA-Gly	II	DUF3892 domain-containing protein	94	20	8.95E-64	93.21						IPR024997 (PFAM); mobidb-lite (MOBIDB_LITE)	no GO terms	no GO terms
34	CP002496.1	Pf-tRNA-Gly	II	Uncharacterized conserved protein	493	20	0	66.04						IPR041685 (PFAM); G3DSA:3.40.50.300 (GENE3D); G3DSA:3.40.50.300 (GENE3D); PTHR43581 (PANTHER); IPR027417 (SUPERFAMILY)	no GO terms	no GO terms
35	CP002496.1	Pf-tRNA-Gly	II	hypothetical protein	201	20	2.11E-127	64.05						Coil (COILS)	no GO terms	no GO terms
36	CP002496.1	Pf-tRNA-Met	II	hypothetical protein	136	20	1.37E-94	99.74	1	C:GO:0005575	C:cellular_component			CYTOPLASMIC_DOMAIN (PHOBIUS); CYTOPLASMIC_DOMAIN (PHOBIUS); TRANSMEMBRANE (PHOBIUS); NON_CYTOPLASMIC_DOMAIN (PHOBIUS); NON_CYTOPLASMIC_DOMAIN (PHOBIUS); TRANSMEMBRANE (PHOBIUS); TRANSMEMBRANE (PHOBIUS); TMhelix (TMHMM); TMhelix (TMHMM)	no GO terms	no GO terms
37	CP002496.1	Pf-tRNA-Met	II	hypothetical protein	111	20	3.66E-75	97.57						SIGNAL_PEPTIDE_H_REGION (PHOBIUS); SIGNAL_PEPTIDE_N_REGION (PHOBIUS); SIGNAL_PEPTIDE_C_REGION (PHOBIUS); NON_CYTOPLASMIC_DOMAIN (PHOBIUS); SIGNAL_PEPTIDE (PHOBIUS); SignalP-noTM (SIGNALP_EUK); SignalP-noTM (SIGNALP_GRAM_NEGATIVE); SignalP-TM (SIGNALP_GRAM_POSITIVE)	no GO terms	no GO terms
38	CP002496.1	Pf-tRNA-Met	II	hypothetical protein	228	20	2.36E-158	63.94						no IPS match	no IPS match	no IPS match
39	CP002496.1	Pf-tRNA-Met	II	hypothetical protein	375	20	0	75.37						Coil (COILS); mobidb-lite (MOBIDB_LITE)	no GO terms	no GO terms
40	CP002496.1	Pf-tRNA-Met	II	hypothetical protein	325	20	0	77.3						no IPS match	no IPS match	no IPS match
41	CP004061.1	Pf-tRNA-Gly	II	Uncharacterised protein	74	20	7.08E-47	85.88						G3DSA:2.30.30.1060 (GENE3D)	no GO terms	no GO terms
42	CP004061.1	Pf-tRNA-Gly	II	DNA-binding protein	88	20	1.29E-57	98.07	1	F:GO:0003677	F:DNA binding			G3DSA:1.10.260.40 (GENE3D); mobidb-lite (MOBIDB_LITE); mobidb-lite (MOBIDB_LITE)	no GO terms	no GO terms
43	CP004061.1	Pf-tRNA-Gly	II	Uncharacterised protein	242	20	4.32E-174	97.58	1	C:GO:0005575	C:cellular_component			mobidb-lite (MOBIDB_LITE); mobidb-lite (MOBIDB_LITE); mobidb-lite (MOBIDB_LITE); mobidb-lite (MOBIDB_LITE); TRANSMEMBRANE (PHOBIUS); CYTOPLASMIC_DOMAIN (PHOBIUS); NON_CYTOPLASMIC_DOMAIN (PHOBIUS); TMhelix (TMHMM)	no GO terms	no GO terms
44	CP004061.1	Pf-tRNA-Gly	II	DUF4238 domain-containing protein	344	20	0	82.47						IPR025332 (PFAM)	no GO terms	no GO terms
45	CP006853.1	Pf-tRNA-Met	I	Uncharacterised protein	145	20	3.37E-100	93.45	1	C:GO:0005575	C:cellular_component			Coil (COILS); TRANSMEMBRANE (PHOBIUS); NON_CYTOPLASMIC_DOMAIN (PHOBIUS); CYTOPLASMIC_DOMAIN (PHOBIUS); TRANSMEMBRANE (PHOBIUS); CYTOPLASMIC_DOMAIN (PHOBIUS); TMhelix (TMHMM); TMhelix (TMHMM)	no GO terms	no GO terms
46	CP006853.1	Pf-tRNA-Met	I	DUF262 domain-containing protein	538	20	0	75.87						IPR004919 (PFAM); PTHR35149 (PANTHER); PTHR35149:SF2 (PANTHER)	no GO terms	no GO terms
47	CP006853.1	Pf-tRNA-Met	I	hypothetical protein	267	20	0	67.23						IPR032557 (PFAM); IPR017930 (PROSITE_PROFILES); cd18714 (CDD)	no GO terms	no GO terms
48	CP006931.1	Pf-tRNA-Met	I	HAD-IA family hydrolase	478	20	0	76.69	6	P:GO:0006259; P:GO:0044281; P:GO:0051276; F:GO:0003677; F:GO:0016853; C:GO:0005694	P:DNA metabolic process; P:small molecule metabolic process; P:chromosome organization; F:DNA binding; F:isomerase activity; C:chromosome			Coil (COILS); IPR013498 (PFAM); G3DSA:3.40.50.2020 (GENE3D); IPR023214 (G3DSA:3.40.50.GENE3D); IPR041492 (PFAM); IPR006439 (TIGRFAM); IPR000836 (PFAM); PTHR43434 (PANTHER); IPR000836 (CDD); IPR029057 (SUPERFAMILY); IPR036412 (SUPERFAMILY)	P:GO:0006265; P:GO:0009116; F:GO:0003677; F:GO:0003916; F:GO:0016787; C:GO:0005694	P:DNA topological change; P:nucleoside metabolic process; F:DNA binding; F:DNA topoisomerase activity; F:hydrolase activity; C:chromosome
49	CP006931.1	Pf-tRNA-Met	I	hypothetical protein	136	20	1.37E-94	99.74	1	C:GO:0005575	C:cellular_component			CYTOPLASMIC_DOMAIN (PHOBIUS); CYTOPLASMIC_DOMAIN (PHOBIUS); TRANSMEMBRANE (PHOBIUS); NON_CYTOPLASMIC_DOMAIN (PHOBIUS); NON_CYTOPLASMIC_DOMAIN (PHOBIUS); TRANSMEMBRANE (PHOBIUS); TRANSMEMBRANE (PHOBIUS); TMhelix (TMHMM); TMhelix (TMHMM)	no GO terms	no GO terms
50	CP006931.1	Pf-tRNA-Met	I	DNA-processing protein DprA	298	20	0	71.89	1	P:GO:0008150	P:biological_process			IPR003488 (PFAM); G3DSA:3.40.50.450 (GENE3D); IPR003488 (PANTHER); SSF102405 (SUPERFAMILY)	P:GO:0009294	P:DNA mediated transformation
51	CP006931.1	Pf-tRNA-Met	I	DUF4868 domain-containing protein	290	20	0	85.42						IPR032359 (PFAM)	no GO terms	no GO terms
52	CP006931.1	Pf-tRNA-Met	I	hypothetical protein	175	20	2.09E-113	78.48	1	C:GO:0005575	C:cellular_component			CYTOPLASMIC_DOMAIN (PHOBIUS); TRANSMEMBRANE (PHOBIUS); TRANSMEMBRANE (PHOBIUS); NON_CYTOPLASMIC_DOMAIN (PHOBIUS); CYTOPLASMIC_DOMAIN (PHOBIUS); CYTOPLASMIC_DOMAIN (PHOBIUS); TRANSMEMBRANE (PHOBIUS); TRANSMEMBRANE (PHOBIUS); NON_CYTOPLASMIC_DOMAIN (PHOBIUS); TMhelix (TMHMM); TMhelix (TMHMM); TMhelix (TMHMM); TMhelix (TMHMM)	no GO terms	no GO terms
53	CP006937.1	Pf-DR	II	ATP-binding protein	598	20	0	83.27	1	F:GO:0043167	F:ion binding			Coil (COILS); G3DSA:3.40.50.300 (GENE3D); IPR041685 (PFAM); IPR027417 (SUPERFAMILY)	no GO terms	no GO terms
54	CP006937.1	Pf-DR	II	Uncharacterised protein	62	20	6.48E-38	89.21						no IPS match	no IPS match	no IPS match
55	CP007147.1	Pf-tRNA-Gly	II	sensor histidine kinase	731	20	0	74.28	1	F:GO:0016301	F:kinase activity			Coil (COILS); IPR003594 (SMART); G3DSA:1.10.287.130 (GENE3D); PF13589 (PFAM); IPR036890 (G3DSA:3.30.565.GENE3D); IPR003594 (PFAM); IPR036890 (G3DSA:3.30.565.GENE3D); PTHR44936 (PANTHER); IPR005467 (PROSITE_PROFILES); cd00075 (CDD); cd16926 (CDD); IPR036890 (SUPERFAMILY); IPR036890 (SUPERFAMILY)	no GO terms	no GO terms
56	CP007147.1	Pf-tRNA-Gly	II	Uncharacterised protein	399	20	0	59.59						no IPS match	no IPS match	no IPS match
57	CP007147.1	Pf-tRNA-Gly	II	Uncharacterised protein	236	14	2.81E-175	56.39						no IPS match	no IPS match	no IPS match
58	CP007147.1	Pf-tRNA-Gly	II	HNH endonuclease	154	20	1.82E-76	78.64	1	F:GO:0004518	F:nuclease activity			Coil (COILS)	no GO terms	no GO terms
59	CP007147.1	Pf-tRNA-Gly	II	type II toxin-antitoxin system Phd/YefM family antitoxin	83	20	6.81E-53	96.37						PTHR33713:SF7 (PANTHER); PTHR33713 (PANTHER); IPR036165 (SUPERFAMILY)	no GO terms	no GO terms
60	CP007147.1	Pf-tRNA-Gly	II	type II toxin-antitoxin system RelE/ParE family toxin	115	20	1.41E-76	98.87						IPR035093 (G3DSA:3.30.2310.GENE3D); IPR007712 (PFAM)	no GO terms	no GO terms
61	CP007147.1	Pf-tRNA-Gly	II	DNA cytosine methyltransferase	347	20	0	92.58	2	P:GO:0090116; F:GO:0003886	P:C-5 methylation of cytosine; F:DNA (cytosine-5-)-methyltransferase activity	EC:2.1.1.37	DNA (cytosine-5-)-methyltransferase	IPR001525 (PRINTS); G3DSA:3.90.120.10 (GENE3D); IPR001525 (PFAM); G3DSA:3.40.50.150 (GENE3D); IPR001525 (TIGRFAM); PTHR10629 (PANTHER); PTHR10629:SF11 (PANTHER); IPR031303 (PROSITE_PATTERNS); IPR018117 (PROSITE_PATTERNS); IPR001525 (PROSITE_PROFILES); IPR029063 (SUPERFAMILY)	F:GO:0008168	F:methyltransferase activity
62	CP007147.1	Pf-tRNA-Met	II	hypothetical protein	136	20	1.76E-94	99.74	1	C:GO:0005575	C:cellular_component			CYTOPLASMIC_DOMAIN (PHOBIUS); CYTOPLASMIC_DOMAIN (PHOBIUS); TRANSMEMBRANE (PHOBIUS); NON_CYTOPLASMIC_DOMAIN (PHOBIUS); NON_CYTOPLASMIC_DOMAIN (PHOBIUS); TRANSMEMBRANE (PHOBIUS); TRANSMEMBRANE (PHOBIUS); TMhelix (TMHMM); TMhelix (TMHMM)	no GO terms	no GO terms
63	CP007147.1	Pf-tRNA-Met	II	Uncharacterised protein	110	20	9.07E-74	91.24						SIGNAL_PEPTIDE_H_REGION (PHOBIUS); NON_CYTOPLASMIC_DOMAIN (PHOBIUS); SIGNAL_PEPTIDE_N_REGION (PHOBIUS); SIGNAL_PEPTIDE_C_REGION (PHOBIUS); SIGNAL_PEPTIDE (PHOBIUS); SignalP-TM (SIGNALP_GRAM_POSITIVE); SignalP-noTM (SIGNALP_EUK); SignalP-noTM (SIGNALP_GRAM_NEGATIVE)	no GO terms	no GO terms
64	CP007147.1	Pf-tRNA-Met	II	Uncharacterised protein	823	20	0	70.11						IPR039444 (PFAM); IPR027417 (SUPERFAMILY)	no GO terms	no GO terms
65	CP007399.1	Pf-tRNA-Met	II	hypothetical protein	136	20	1.37E-94	99.74	1	C:GO:0005575	C:cellular_component			CYTOPLASMIC_DOMAIN (PHOBIUS); CYTOPLASMIC_DOMAIN (PHOBIUS); TRANSMEMBRANE (PHOBIUS); NON_CYTOPLASMIC_DOMAIN (PHOBIUS); NON_CYTOPLASMIC_DOMAIN (PHOBIUS); TRANSMEMBRANE (PHOBIUS); TRANSMEMBRANE (PHOBIUS); TMhelix (TMHMM); TMhelix (TMHMM)	no GO terms	no GO terms
66	CP007399.1	Pf-tRNA-Met	II	AAA family ATPase	784	20	0	72.39	3	P:GO:0008150; F:GO:0003677; F:GO:0043167	P:biological_process; F:DNA binding; F:ion binding			IPR038729 (PFAM); IPR003959 (PFAM); G3DSA:3.40.50.300 (GENE3D); G3DSA:3.40.50.300 (GENE3D); PTHR32182 (PANTHER); CYTOPLASMIC_DOMAIN (PHOBIUS); TRANSMEMBRANE (PHOBIUS); IPR027417 (SUPERFAMILY)	F:GO:0005524	F:ATP binding
67	CP007399.1	Pf-tRNA-Met	II	hypothetical protein	229	20	1.70E-167	61.06						no IPS match	no IPS match	no IPS match
68	CP007399.1	Pf-tRNA-Met	II	Uncharacterised protein	425	20	0	68.15						no IPS match	no IPS match	no IPS match
69	CP007399.1	Pf-tRNA-Met	II	hypothetical protein	258	20	0	70.72	1	C:GO:0005575	C:cellular_component			mobidb-lite (MOBIDB_LITE); mobidb-lite (MOBIDB_LITE); mobidb-lite (MOBIDB_LITE); NON_CYTOPLASMIC_DOMAIN (PHOBIUS); CYTOPLASMIC_DOMAIN (PHOBIUS); TRANSMEMBRANE (PHOBIUS); TMhelix (TMHMM)	no GO terms	no GO terms
70	CP007399.1c	Pf-tRNA-Gly	I	Uncharacterised protein	74	20	4.51E-47	86.4						no IPS match	no IPS match	no IPS match
71	CP007399.1c	Pf-tRNA-Gly	I	hypothetical protein	242	20	1.04E-173	96.23	1	C:GO:0005575	C:cellular_component			mobidb-lite (MOBIDB_LITE); mobidb-lite (MOBIDB_LITE); mobidb-lite (MOBIDB_LITE); mobidb-lite (MOBIDB_LITE); mobidb-lite (MOBIDB_LITE); mobidb-lite (MOBIDB_LITE); TRANSMEMBRANE (PHOBIUS); CYTOPLASMIC_DOMAIN (PHOBIUS); NON_CYTOPLASMIC_DOMAIN (PHOBIUS); TMhelix (TMHMM)	no GO terms	no GO terms
72	CP007399.1c	Pf-tRNA-Gly	I	hypothetical protein	311	20	0	82.3						no IPS match	no IPS match	no IPS match
73	CP007399.1c	Pf-tRNA-Gly	I	hypothetical protein	297	20	0	75.34						no IPS match	no IPS match	no IPS match
74	CP008857.1	Pf-tRNA-Gly	I	Uncharacterised protein	74	20	1.99E-46	84.41						G3DSA:2.30.30.1060 (GENE3D)	no GO terms	no GO terms
75	CP008857.1	Pf-tRNA-Gly	I	Uncharacterised protein	232	20	3.10E-174	98.36	1	C:GO:0005575	C:cellular_component			mobidb-lite (MOBIDB_LITE); mobidb-lite (MOBIDB_LITE); mobidb-lite (MOBIDB_LITE); TRANSMEMBRANE (PHOBIUS); NON_CYTOPLASMIC_DOMAIN (PHOBIUS); CYTOPLASMIC_DOMAIN (PHOBIUS); TMhelix (TMHMM)	no GO terms	no GO terms
76	CP008857.1	Pf-tRNA-Gly	I	ATP-binding protein	412	20	0	76.22						IPR027417 (SUPERFAMILY)	no GO terms	no GO terms
77	CP008857.1	Pf-tmRNA	II	SinR family protein	91	20	9.16E-61	70.77						no IPS match	no IPS match	no IPS match
78	CP008857.1	Pf-tmRNA	II	DUF2806 domain-containing protein	347	20	0	72.62						IPR021254 (PFAM)	no GO terms	no GO terms
79	CP008859.2	Pf-tRNA-Met	I	Uncharacterised protein	145	20	1.57E-99	93.83	1	C:GO:0005575	C:cellular_component			TRANSMEMBRANE (PHOBIUS); TRANSMEMBRANE (PHOBIUS); CYTOPLASMIC_DOMAIN (PHOBIUS); NON_CYTOPLASMIC_DOMAIN (PHOBIUS); CYTOPLASMIC_DOMAIN (PHOBIUS); TMhelix (TMHMM); TMhelix (TMHMM)	no GO terms	no GO terms
80	CP008859.2	Pf-tRNA-Met	I	hypothetical protein	111	20	3.04E-75	98.47						SIGNAL_PEPTIDE_H_REGION (PHOBIUS); SIGNAL_PEPTIDE_N_REGION (PHOBIUS); SIGNAL_PEPTIDE_C_REGION (PHOBIUS); NON_CYTOPLASMIC_DOMAIN (PHOBIUS); SIGNAL_PEPTIDE (PHOBIUS); SignalP-TM (SIGNALP_GRAM_POSITIVE); SignalP-noTM (SIGNALP_EUK); SignalP-noTM (SIGNALP_GRAM_NEGATIVE)	no GO terms	no GO terms
81	CP008859.2	Pf-tRNA-Met	I	ATP-binding protein	742	20	0	81.74	1	F:GO:0016301	F:kinase activity			PF13589 (PFAM); IPR036890 (G3DSA:3.30.565.GENE3D); cd16926 (CDD); IPR036890 (SUPERFAMILY)	no GO terms	no GO terms
82	CP008859.2	Pf-tRNA-Met	I	(deoxy)nucleoside triphosphate pyrophosphohydrolase	80	20	3.35E-50	90.31	3	P:GO:0006281; F:GO:0003674; F:GO:0016787	P:DNA repair; F:molecular_function; F:hydrolase activity	EC:3.6.1.55; EC:3.6.1.65	8-oxo-dGTP diphosphatase; (d)CTP diphosphatase	IPR020476 (PRINTS); IPR000086 (PFAM); G3DSA:3.90.79.10 (GENE3D); PTHR47707 (PANTHER); IPR020084 (PROSITE_PATTERNS); IPR000086 (PROSITE_PROFILES); IPR015797 (SUPERFAMILY)	F:GO:0016787	F:hydrolase activity
83	CP008859.2	Pf-tRNA-Met	I	DNA (cytosine-5-)-methyltransferase	361	20	0	78.78	2	P:GO:0090116; F:GO:0003886	P:C-5 methylation of cytosine; F:DNA (cytosine-5-)-methyltransferase activity	EC:2.1.1.37	DNA (cytosine-5-)-methyltransferase	IPR001525 (PRINTS); G3DSA:3.40.50.150 (GENE3D); IPR001525 (TIGRFAM); IPR001525 (PFAM); G3DSA:3.90.120.10 (GENE3D); PTHR10629:SF11 (PANTHER); PTHR10629 (PANTHER); IPR018117 (PROSITE_PATTERNS); IPR001525 (PROSITE_PROFILES); IPR029063 (SUPERFAMILY)	F:GO:0008168	F:methyltransferase activity
84	CP008860.2	Pf-tRNA-Gly	I	Uncharacterised protein	74	20	4.51E-47	86.4						no IPS match	no IPS match	no IPS match
85	CP008860.2	Pf-tRNA-Gly	I	hypothetical protein	230	20	5.62E-166	55.31	1	C:GO:0005575	C:cellular_component			CYTOPLASMIC_DOMAIN (PHOBIUS); TRANSMEMBRANE (PHOBIUS); NON_CYTOPLASMIC_DOMAIN (PHOBIUS); CYTOPLASMIC_DOMAIN (PHOBIUS); TRANSMEMBRANE (PHOBIUS); NON_CYTOPLASMIC_DOMAIN (PHOBIUS); TRANSMEMBRANE (PHOBIUS); TMhelix (TMHMM); TMhelix (TMHMM); TMhelix (TMHMM)	no GO terms	no GO terms
86	CP008860.2	Pf-tRNA-Gly	I	type 1 glutamine amidotransferase	119	7	2.02E-83	80.02	1	P:GO:0006520	P:cellular amino acid metabolic process			no IPS match	no IPS match	no IPS match
87	CP008862.2	Pf-tRNA-Met	I	Uncharacterised protein	145	20	3.37E-100	93.45	1	C:GO:0005575	C:cellular_component			Coil (COILS); TRANSMEMBRANE (PHOBIUS); CYTOPLASMIC_DOMAIN (PHOBIUS); TRANSMEMBRANE (PHOBIUS); CYTOPLASMIC_DOMAIN (PHOBIUS); NON_CYTOPLASMIC_DOMAIN (PHOBIUS); TMhelix (TMHMM); TMhelix (TMHMM)	no GO terms	no GO terms
88	CP008862.2	Pf-tRNA-Met	I	sce7726 family protein	193	20	4.42E-138	76.55						no IPS match	no IPS match	no IPS match
89	CP008862.2	Pf-tRNA-Met	I	Uncharacterised protein	356	20	0	87.11						IPR025683 (PFAM)	no GO terms	no GO terms
90	CP008862.2	Pf-tRNA-Met	I	ImmA/IrrE family metallo-endopeptidase	278	20	0	89.91						IPR010359 (PFAM)	no GO terms	no GO terms
91	CP008862.2	Pf-tRNA-Met	I	N-acetyltransferase	224	20	7.43E-168	70.71	1	F:GO:0003674	F:molecular_function			IPR016181 (SUPERFAMILY)	no GO terms	no GO terms
92	CP008862.2	Pf-tRNA-Met	I	MULTISPECIES: hypothetical protein	135	20	1.99E-94	98.03						no IPS match	no IPS match	no IPS match
93	CP008862.2	Pf-DR	I	Uncharacterised protein	202	20	1.41E-145	74.69						IPR041519 (PFAM)	no GO terms	no GO terms
94	CP008862.2	Pf-DR	I	ParA family protein	343	20	0	82.15	4	P:GO:0071941; F:GO:0016491; C:GO:0005622; C:GO:0032991	P:nitrogen cycle metabolic process; F:oxidoreductase activity; C:intracellular; C:protein-containing complex	EC:1.18.6.1; EC:1.18.6	Nitrogenase; Acting on iron-sulfur proteins as donors	Coil (COILS); IPR025669 (PFAM); G3DSA:3.40.50.300 (GENE3D); PTHR13696:SF52 (PANTHER); PTHR13696 (PANTHER); cd02042 (CDD); IPR027417 (SUPERFAMILY)	no GO terms	no GO terms
95	CP008863.1	Pf-tRNA-Met	I	Uncharacterised protein	145	20	3.37E-100	93.45	1	C:GO:0005575	C:cellular_component			Coil (COILS); TRANSMEMBRANE (PHOBIUS); CYTOPLASMIC_DOMAIN (PHOBIUS); TRANSMEMBRANE (PHOBIUS); CYTOPLASMIC_DOMAIN (PHOBIUS); NON_CYTOPLASMIC_DOMAIN (PHOBIUS); TMhelix (TMHMM); TMhelix (TMHMM)	no GO terms	no GO terms
96	CP008863.1	Pf-tRNA-Met	I	sce7726 family protein	193	20	4.42E-138	76.55						no IPS match	no IPS match	no IPS match
97	CP008863.1	Pf-tRNA-Met	I	Uncharacterised protein	356	20	0	87.11						IPR025683 (PFAM)	no GO terms	no GO terms
98	CP008863.1	Pf-tRNA-Met	I	ImmA/IrrE family metallo-endopeptidase	278	20	0	89.91						IPR010359 (PFAM)	no GO terms	no GO terms
99	CP008863.1	Pf-tRNA-Met	I	N-acetyltransferase	224	20	7.43E-168	70.71	1	F:GO:0003674	F:molecular_function			IPR016181 (SUPERFAMILY)	no GO terms	no GO terms
100	CP008863.1	Pf-tRNA-Met	I	MULTISPECIES: hypothetical protein	135	20	1.99E-94	98.03						no IPS match	no IPS match	no IPS match
101	CP008863.1	Pf-DR	I	Uncharacterised protein	202	20	1.41E-145	74.69						IPR041519 (PFAM)	no GO terms	no GO terms
102	CP008863.1	Pf-DR	I	ParA family protein	343	20	0	82.15	4	P:GO:0071941; F:GO:0016491; C:GO:0005622; C:GO:0032991	P:nitrogen cycle metabolic process; F:oxidoreductase activity; C:intracellular; C:protein-containing complex	EC:1.18.6.1; EC:1.18.6	Nitrogenase; Acting on iron-sulfur proteins as donors	Coil (COILS); IPR025669 (PFAM); G3DSA:3.40.50.300 (GENE3D); PTHR13696:SF52 (PANTHER); PTHR13696 (PANTHER); cd02042 (CDD); IPR027417 (SUPERFAMILY)	no GO terms	no GO terms
103	CP008864.2	Pf-tRNA-Gly	II	DUF4365 domain-containing protein	528	20	0	63.87						no IPS match	no IPS match	no IPS match
104	CP008864.2	Pf-tRNA-Gly	II	---NA---	75									SIGNAL_PEPTIDE_H_REGION (PHOBIUS); SIGNAL_PEPTIDE (PHOBIUS); SIGNAL_PEPTIDE_N_REGION (PHOBIUS); SIGNAL_PEPTIDE_C_REGION (PHOBIUS); NON_CYTOPLASMIC_DOMAIN (PHOBIUS)	no GO terms	no GO terms
105	CP008864.2	Pf-tRNA-Gly	II	DUF4238 domain-containing protein	349	20	0	70.17						IPR025332 (PFAM)	no GO terms	no GO terms
106	CP008864.2	Pf-tRNA-Gly	II	Uncharacterised protein	230	20	2.72E-173	94.43	1	C:GO:0005575	C:cellular_component			mobidb-lite (MOBIDB_LITE); mobidb-lite (MOBIDB_LITE); mobidb-lite (MOBIDB_LITE); mobidb-lite (MOBIDB_LITE); TRANSMEMBRANE (PHOBIUS); NON_CYTOPLASMIC_DOMAIN (PHOBIUS); CYTOPLASMIC_DOMAIN (PHOBIUS); TMhelix (TMHMM)	no GO terms	no GO terms
107	CP008864.2	Pf-tRNA-Gly	II	Uncharacterised protein	74	20	4.51E-47	86.4						no IPS match	no IPS match	no IPS match
108	CP008864.2	Pf-tRNA-Met	I	Uncharacterised protein	145	20	3.37E-100	93.45	1	C:GO:0005575	C:cellular_component			Coil (COILS); TRANSMEMBRANE (PHOBIUS); CYTOPLASMIC_DOMAIN (PHOBIUS); CYTOPLASMIC_DOMAIN (PHOBIUS); TRANSMEMBRANE (PHOBIUS); NON_CYTOPLASMIC_DOMAIN (PHOBIUS); TMhelix (TMHMM); TMhelix (TMHMM)	no GO terms	no GO terms
109	CP008864.2	Pf-tRNA-Met	I	hypothetical protein	111	20	4.93E-75	96.71						mobidb-lite (MOBIDB_LITE); mobidb-lite (MOBIDB_LITE); SIGNAL_PEPTIDE_H_REGION (PHOBIUS); SIGNAL_PEPTIDE_N_REGION (PHOBIUS); SIGNAL_PEPTIDE_C_REGION (PHOBIUS); NON_CYTOPLASMIC_DOMAIN (PHOBIUS); SIGNAL_PEPTIDE (PHOBIUS); SignalP-TM (SIGNALP_GRAM_POSITIVE); SignalP-noTM (SIGNALP_EUK); SignalP-noTM (SIGNALP_GRAM_NEGATIVE)	no GO terms	no GO terms
110	CP008864.2	Pf-tRNA-Met	I	Uncharacterised protein	363	20	0	72.34	1	C:GO:0005575	C:cellular_component			CYTOPLASMIC_DOMAIN (PHOBIUS); TRANSMEMBRANE (PHOBIUS); NON_CYTOPLASMIC_DOMAIN (PHOBIUS); CYTOPLASMIC_DOMAIN (PHOBIUS); TRANSMEMBRANE (PHOBIUS); TMhelix (TMHMM); TMhelix (TMHMM)	no GO terms	no GO terms
111	CP008864.2	Pf-tRNA-Met	I	hypothetical protein	78	20	7.30E-50	96.29						no IPS match	no IPS match	no IPS match
112	CP008865.2	Pf-tRNA-Gly	II	type II toxin-antitoxin system RelE/ParE family toxin	115	20	5.90E-77	98.35						IPR007712 (PFAM); IPR035093 (G3DSA:3.30.2310.GENE3D)	no GO terms	no GO terms
113	CP008865.2	Pf-tRNA-Gly	II	type II toxin-antitoxin system Phd/YefM family antitoxin	83	20	6.81E-53	96.37						PTHR33713:SF7 (PANTHER); PTHR33713 (PANTHER); IPR036165 (SUPERFAMILY)	no GO terms	no GO terms
114	CP008865.2	Pf-tRNA-Gly	II	Uncharacterised protein	230	20	2.58E-173	96.26	1	C:GO:0005575	C:cellular_component			mobidb-lite (MOBIDB_LITE); mobidb-lite (MOBIDB_LITE); mobidb-lite (MOBIDB_LITE); TRANSMEMBRANE (PHOBIUS); NON_CYTOPLASMIC_DOMAIN (PHOBIUS); CYTOPLASMIC_DOMAIN (PHOBIUS); TMhelix (TMHMM)	no GO terms	no GO terms
115	CP008865.2	Pf-tRNA-Gly	II	Uncharacterised protein	504	20	0	74.14						mobidb-lite (MOBIDB_LITE)	no GO terms	no GO terms
116	CP008865.2	Pf-tRNA-Met	II	MULTISPECIES: hypothetical protein	136	20	1.03E-93	98.01	1	C:GO:0005575	C:cellular_component			CYTOPLASMIC_DOMAIN (PHOBIUS); TRANSMEMBRANE (PHOBIUS); CYTOPLASMIC_DOMAIN (PHOBIUS); NON_CYTOPLASMIC_DOMAIN (PHOBIUS); NON_CYTOPLASMIC_DOMAIN (PHOBIUS); TRANSMEMBRANE (PHOBIUS); TRANSMEMBRANE (PHOBIUS); TMhelix (TMHMM); TMhelix (TMHMM); TMhelix (TMHMM)	no GO terms	no GO terms
117	CP008865.2	Pf-tRNA-Met	II	Cro/CI family transcriptional regulator	264	20	0	86.3	7	P:GO:0001666; P:GO:0019217; P:GO:0045892; P:GO:0045926; P:GO:0045927; F:GO:0003677	P:response to hypoxia; P:regulation of fatty acid metabolic process; P:negative regulation of transcription, DNA-templated; P:negative regulation of growth; P:positive regulation of growth; F:DNA binding			IPR001387 (SMART); IPR007374 (SMART); IPR007374 (PFAM); IPR001387 (PFAM); G3DSA:1.10.260.40 (GENE3D); G3DSA:2.30.130.30 (GENE3D); IPR001387 (PROSITE_PROFILES); IPR001387 (CDD); IPR015947 (SUPERFAMILY); IPR010982 (SUPERFAMILY)	F:GO:0003677	F:DNA binding
118	CP008865.2	Pf-tRNA-Met	II	GNAT family N-acetyltransferase	348	20	0	98.72	1	F:GO:0016746	F:transferase activity, transferring acyl groups	EC:2.3.1.5	Arylamine N-acetyltransferase	G3DSA:3.40.630.30 (GENE3D); IPR000182 (PFAM); IPR000182 (PROSITE_PROFILES); cd18699 (CDD); IPR016181 (SUPERFAMILY)	F:GO:0008080	F:N-acetyltransferase activity
119	CP008865.2	Pf-tRNA-Met	II	GNAT family N-acetyltransferase	179	20	3.11E-123	99.38	1	F:GO:0016746	F:transferase activity, transferring acyl groups	EC:2.3.1.5	Arylamine N-acetyltransferase	no IPS match	no IPS match	no IPS match
120	CP008865.2	Pf-tRNA-Met	II	AAA family ATPase	175	20	2.76E-124	83.12				EC:2.7.4.3	Adenylate kinase	PF13207 (PFAM); G3DSA:3.40.50.300 (GENE3D); IPR027417 (SUPERFAMILY)	no GO terms	no GO terms
121	CP008865.2	Pf-tRNA-Met	II	IS30 family transposase	342	20	0	98.58	4	P:GO:0006313; P:GO:0015074; F:GO:0003677; F:GO:0004803	P:transposition, DNA-mediated; P:DNA integration; F:DNA binding; F:transposase activity			IPR036397 (G3DSA:3.30.420.GENE3D); IPR025246 (PFAM); IPR001584 (PFAM); PTHR10948 (PANTHER); IPR001598 (PROSITE_PATTERNS); IPR001584 (PROSITE_PROFILES); IPR012337 (SUPERFAMILY)	P:GO:0006313; P:GO:0015074; F:GO:0003676; F:GO:0003677; F:GO:0004803	P:transposition, DNA-mediated; P:DNA integration; F:nucleic acid binding; F:DNA binding; F:transposase activity
122	CP008865.2	Pf-tRNA-Met	II	IS30 family transposase	342	20	0	98.58	4	P:GO:0006313; P:GO:0015074; F:GO:0003677; F:GO:0004803	P:transposition, DNA-mediated; P:DNA integration; F:DNA binding; F:transposase activity			IPR036397 (G3DSA:3.30.420.GENE3D); IPR025246 (PFAM); IPR001584 (PFAM); PTHR10948 (PANTHER); IPR001598 (PROSITE_PATTERNS); IPR001584 (PROSITE_PROFILES); IPR012337 (SUPERFAMILY)	P:GO:0006313; P:GO:0015074; F:GO:0003676; F:GO:0003677; F:GO:0004803	P:transposition, DNA-mediated; P:DNA integration; F:nucleic acid binding; F:DNA binding; F:transposase activity
123	CP008866.2	Pf-tRNA-Gly	II	type II toxin-antitoxin system RelE/ParE family toxin	115	20	5.90E-77	98.35						IPR007712 (PFAM); IPR035093 (G3DSA:3.30.2310.GENE3D)	no GO terms	no GO terms
124	CP008866.2	Pf-tRNA-Gly	II	type II toxin-antitoxin system Phd/YefM family antitoxin	83	20	6.81E-53	96.37						PTHR33713:SF7 (PANTHER); PTHR33713 (PANTHER); IPR036165 (SUPERFAMILY)	no GO terms	no GO terms
125	CP008866.2	Pf-tRNA-Gly	II	Uncharacterised protein	230	20	2.58E-173	96.26	1	C:GO:0005575	C:cellular_component			mobidb-lite (MOBIDB_LITE); mobidb-lite (MOBIDB_LITE); mobidb-lite (MOBIDB_LITE); TRANSMEMBRANE (PHOBIUS); NON_CYTOPLASMIC_DOMAIN (PHOBIUS); CYTOPLASMIC_DOMAIN (PHOBIUS); TMhelix (TMHMM)	no GO terms	no GO terms
126	CP008866.2	Pf-tRNA-Gly	II	Uncharacterised protein	504	20	0	74.14						mobidb-lite (MOBIDB_LITE)	no GO terms	no GO terms
127	CP008866.2	Pf-tRNA-Met	II	MULTISPECIES: hypothetical protein	136	20	1.03E-93	98.01	1	C:GO:0005575	C:cellular_component			CYTOPLASMIC_DOMAIN (PHOBIUS); TRANSMEMBRANE (PHOBIUS); CYTOPLASMIC_DOMAIN (PHOBIUS); NON_CYTOPLASMIC_DOMAIN (PHOBIUS); NON_CYTOPLASMIC_DOMAIN (PHOBIUS); TRANSMEMBRANE (PHOBIUS); TRANSMEMBRANE (PHOBIUS); TMhelix (TMHMM); TMhelix (TMHMM); TMhelix (TMHMM)	no GO terms	no GO terms
128	CP008866.2	Pf-tRNA-Met	II	Cro/CI family transcriptional regulator	264	20	0	86.3	7	P:GO:0001666; P:GO:0019217; P:GO:0045892; P:GO:0045926; P:GO:0045927; F:GO:0003677	P:response to hypoxia; P:regulation of fatty acid metabolic process; P:negative regulation of transcription, DNA-templated; P:negative regulation of growth; P:positive regulation of growth; F:DNA binding			IPR001387 (SMART); IPR007374 (SMART); IPR007374 (PFAM); IPR001387 (PFAM); G3DSA:1.10.260.40 (GENE3D); G3DSA:2.30.130.30 (GENE3D); IPR001387 (PROSITE_PROFILES); IPR001387 (CDD); IPR015947 (SUPERFAMILY); IPR010982 (SUPERFAMILY)	F:GO:0003677	F:DNA binding
129	CP008866.2	Pf-tRNA-Met	II	GNAT family N-acetyltransferase	512	20	0	98.42	1	F:GO:0016746	F:transferase activity, transferring acyl groups	EC:2.3.1.5	Arylamine N-acetyltransferase	G3DSA:3.40.630.30 (GENE3D); IPR000182 (PFAM); IPR000182 (PROSITE_PROFILES); cd18699 (CDD); IPR016181 (SUPERFAMILY)	F:GO:0008080	F:N-acetyltransferase activity
130	CP008866.2	Pf-tRNA-Met	II	AAA family ATPase	175	20	2.76E-124	83.12				EC:2.7.4.3	Adenylate kinase	PF13207 (PFAM); G3DSA:3.40.50.300 (GENE3D); IPR027417 (SUPERFAMILY)	no GO terms	no GO terms
131	CP008867.1	Pf-tRNA-Gly	I	ppGpp synthetase catalytic domain-containing protein (RelA/SpoT-type nucleotidyltranferase)	366	20	0	70.22	4	P:GO:0034641; P:GO:0044281	P:cellular nitrogen compound metabolic process; P:small molecule metabolic process	EC:2.7.6.5	GTP diphosphokinase	Coil (COILS); IPR007685 (SMART); G3DSA:3.30.460.10 (GENE3D); G3DSA:1.10.287.860 (GENE3D); IPR007685 (PFAM); PTHR41773 (PANTHER); IPR007685 (CDD); SSF81301 (SUPERFAMILY)	P:GO:0015969	P:guanosine tetraphosphate metabolic process
132	CP008867.1	Pf-tRNA-Gly	I	DUF262 domain-containing protein	392	20	0	79.88						Coil (COILS)	no GO terms	no GO terms
133	CP008867.1	Pf-tRNA-Gly	I	Uncharacterised protein	391	20	0	58.64						no IPS match	no IPS match	no IPS match
134	CP008867.1	Pf-tRNA-Gly	I	cyclic nucleotide-binding domain-containing protein	43	1	6.13415	63.16	3	P:GO:0009058; P:GO:0034641; F:GO:0003677	P:biosynthetic process; P:cellular nitrogen compound metabolic process; F:DNA binding			SIGNAL_PEPTIDE_H_REGION (PHOBIUS); NON_CYTOPLASMIC_DOMAIN (PHOBIUS); SIGNAL_PEPTIDE (PHOBIUS); SIGNAL_PEPTIDE_C_REGION (PHOBIUS); SIGNAL_PEPTIDE_N_REGION (PHOBIUS); SignalP-noTM (SIGNALP_GRAM_NEGATIVE); TMhelix (TMHMM)	no GO terms	no GO terms
135	CP008867.1	Pf-tRNA-Met	I	hypothetical protein	136	20	8.43E-95	99.08	1	C:GO:0005575	C:cellular_component			CYTOPLASMIC_DOMAIN (PHOBIUS); CYTOPLASMIC_DOMAIN (PHOBIUS); TRANSMEMBRANE (PHOBIUS); NON_CYTOPLASMIC_DOMAIN (PHOBIUS); NON_CYTOPLASMIC_DOMAIN (PHOBIUS); TRANSMEMBRANE (PHOBIUS); TRANSMEMBRANE (PHOBIUS); TMhelix (TMHMM); TMhelix (TMHMM)	no GO terms	no GO terms
136	CP008867.1	Pf-tRNA-Met	I	hypothetical protein	111	20	2.85E-75	97.57						SIGNAL_PEPTIDE_H_REGION (PHOBIUS); SIGNAL_PEPTIDE_N_REGION (PHOBIUS); SIGNAL_PEPTIDE_C_REGION (PHOBIUS); NON_CYTOPLASMIC_DOMAIN (PHOBIUS); SIGNAL_PEPTIDE (PHOBIUS); SignalP-noTM (SIGNALP_GRAM_NEGATIVE); SignalP-noTM (SIGNALP_EUK); SignalP-TM (SIGNALP_GRAM_POSITIVE)	no GO terms	no GO terms
137	CP008867.1	Pf-tRNA-Met	I	restriction endonuclease	394	20	0	77.64	2	P:GO:0006259; F:GO:0008168	P:DNA metabolic process; F:methyltransferase activity	EC:2.1.1.72	Site-specific DNA-methyltransferase (adenine-specific)	IPR012327 (PFAM); IPR029063 (SUPERFAMILY)	P:GO:0032775; F:GO:0009007	P:DNA methylation on adenine; F:site-specific DNA-methyltransferase (adenine-specific) activity
138	CP008867.1	Pf-tRNA-Met	I	helix-turn-helix transcriptional regulator	97	20	2.87E-63	77.26	1	F:GO:0003677	F:DNA binding			IPR001387 (SMART); IPR001387 (PFAM); G3DSA:1.10.260.40 (GENE3D); PTHR43236 (PANTHER); IPR001387 (PROSITE_PROFILES); IPR001387 (CDD); IPR010982 (SUPERFAMILY)	F:GO:0003677	F:DNA binding
139	CP008867.1	Pf-tRNA-Met	I	restriction endonuclease	268	20	0	81.94	3	P:GO:0006259; P:GO:0006950; F:GO:0004518	P:DNA metabolic process; P:response to stress; F:nuclease activity	EC:3.1.30; EC:3.1.21; EC:3.1.21.4	Acting on ester bonds; Acting on ester bonds; Type II site-specific deoxyribonuclease	IPR015278 (PFAM); IPR011335 (SUPERFAMILY)	P:GO:0009307; F:GO:0009036	P:DNA restriction-modification system; F:type II site-specific deoxyribonuclease activity
140	CP008868.1	Pf-tRNA-Met	I	hypothetical protein	136	20	8.16E-95	99.08	1	C:GO:0005575	C:cellular_component			CYTOPLASMIC_DOMAIN (PHOBIUS); CYTOPLASMIC_DOMAIN (PHOBIUS); TRANSMEMBRANE (PHOBIUS); NON_CYTOPLASMIC_DOMAIN (PHOBIUS); NON_CYTOPLASMIC_DOMAIN (PHOBIUS); TRANSMEMBRANE (PHOBIUS); TRANSMEMBRANE (PHOBIUS); TMhelix (TMHMM); TMhelix (TMHMM)	no GO terms	no GO terms
141	CP008868.1	Pf-tRNA-Met	I	hypothetical protein	111	20	2.02E-75	98.38						SIGNAL_PEPTIDE_H_REGION (PHOBIUS); SIGNAL_PEPTIDE_N_REGION (PHOBIUS); SIGNAL_PEPTIDE_C_REGION (PHOBIUS); NON_CYTOPLASMIC_DOMAIN (PHOBIUS); SIGNAL_PEPTIDE (PHOBIUS); SignalP-noTM (SIGNALP_GRAM_NEGATIVE); SignalP-TM (SIGNALP_GRAM_POSITIVE); SignalP-noTM (SIGNALP_EUK)	no GO terms	no GO terms
142	CP008868.1	Pf-tRNA-Met	I	hypothetical protein	253	20	2.42E-180	65.08	1	C:GO:0005575	C:cellular_component			TRANSMEMBRANE (PHOBIUS); NON_CYTOPLASMIC_DOMAIN (PHOBIUS); NON_CYTOPLASMIC_DOMAIN (PHOBIUS); TRANSMEMBRANE (PHOBIUS); CYTOPLASMIC_DOMAIN (PHOBIUS); TRANSMEMBRANE (PHOBIUS); CYTOPLASMIC_DOMAIN (PHOBIUS); TMhelix (TMHMM); TMhelix (TMHMM); TMhelix (TMHMM)	no GO terms	no GO terms
143	CP008868.1	Pf-tRNA-Met	I	RNA-directed DNA polymerase	402	20	0	80.85	4	P:GO:0006259; P:GO:0009058; F:GO:0003723; F:GO:0016779	P:DNA metabolic process; P:biosynthetic process; F:RNA binding; F:nucleotidyltransferase activity	EC:2.7.7.49	RNA-directed DNA polymerase	IPR000123 (PRINTS); IPR000477 (PFAM); PTHR34047 (PANTHER); PTHR34047:SF6 (PANTHER); IPR000477 (PROSITE_PROFILES); cd03487 (CDD); SSF56672 (SUPERFAMILY)	P:GO:0006278; F:GO:0003723; F:GO:0003964	P:RNA-dependent DNA biosynthetic process; F:RNA binding; F:RNA-directed DNA polymerase activity
144	CP008870.2	Pf-tRNA-Met	I	Uncharacterised protein	294	20	0	77.63	1	C:GO:0005575	C:cellular_component			NON_CYTOPLASMIC_DOMAIN (PHOBIUS); NON_CYTOPLASMIC_DOMAIN (PHOBIUS); TRANSMEMBRANE (PHOBIUS); NON_CYTOPLASMIC_DOMAIN (PHOBIUS); CYTOPLASMIC_DOMAIN (PHOBIUS); TRANSMEMBRANE (PHOBIUS); CYTOPLASMIC_DOMAIN (PHOBIUS); CYTOPLASMIC_DOMAIN (PHOBIUS); TRANSMEMBRANE (PHOBIUS); NON_CYTOPLASMIC_DOMAIN (PHOBIUS); TRANSMEMBRANE (PHOBIUS); TRANSMEMBRANE (PHOBIUS); SignalP-TM (SIGNALP_EUK); TMhelix (TMHMM); TMhelix (TMHMM); TMhelix (TMHMM); TMhelix (TMHMM); TMhelix (TMHMM); TMhelix (TMHMM)	no GO terms	no GO terms
145	CP008870.2	Pf-tRNA-Met	I	Uncharacterised protein	111	20	6.27E-75	97.52						mobidb-lite (MOBIDB_LITE); mobidb-lite (MOBIDB_LITE); SIGNAL_PEPTIDE_H_REGION (PHOBIUS); SIGNAL_PEPTIDE_N_REGION (PHOBIUS); SIGNAL_PEPTIDE_C_REGION (PHOBIUS); NON_CYTOPLASMIC_DOMAIN (PHOBIUS); SIGNAL_PEPTIDE (PHOBIUS); SignalP-noTM (SIGNALP_EUK); SignalP-noTM (SIGNALP_GRAM_NEGATIVE); SignalP-TM (SIGNALP_GRAM_POSITIVE)	no GO terms	no GO terms
146	CP008870.2	Pf-tRNA-Met	I	restriction endonuclease	220	20	6.99E-158	85.85	8	P:GO:0006265; P:GO:0009307; P:GO:0090305; F:GO:0003677; F:GO:0003916; F:GO:0004519; C:GO:0005694; C:GO:0016021	P:DNA topological change; P:DNA restriction-modification system; P:nucleic acid phosphodiester bond hydrolysis; F:DNA binding; F:DNA topoisomerase activity; F:endonuclease activity; C:chromosome; C:integral component of membrane			IPR007560 (PFAM); IPR011856 (G3DSA:3.40.1350.GENE3D); PTHR30015 (PANTHER); TRANSMEMBRANE (PHOBIUS); TRANSMEMBRANE (PHOBIUS); CYTOPLASMIC_DOMAIN (PHOBIUS); NON_CYTOPLASMIC_DOMAIN (PHOBIUS); CYTOPLASMIC_DOMAIN (PHOBIUS); IPR011335 (SUPERFAMILY); TMhelix (TMHMM); TMhelix (TMHMM)	P:GO:0009307; F:GO:0003676; F:GO:0003677; F:GO:0004518; F:GO:0004519	P:DNA restriction-modification system; F:nucleic acid binding; F:DNA binding; F:nuclease activity; F:endonuclease activity
147	CP008870.2	Pf-tRNA-Met	I	MULTISPECIES: hypothetical protein	137	20	1.21E-95	97.88						no IPS match	no IPS match	no IPS match
148	CP008870.2	Pf-tRNA-Met	I	IS4 family transposase	423	20	0	99.79	3	P:GO:0006259; P:GO:0032196; F:GO:0003677	P:DNA metabolic process; P:transposition; F:DNA binding			IPR002559 (PFAM); PTHR37529 (PANTHER); IPR012337 (SUPERFAMILY)	P:GO:0006313; F:GO:0003677; F:GO:0004803	P:transposition, DNA-mediated; F:DNA binding; F:transposase activity
149	CP008870.2	Pf-tRNA-Met	I	cytidine deaminase	588	20	0	88.11	4	F:GO:0008270; F:GO:0016787	F:zinc ion binding; F:hydrolase activity	EC:3.5.4.12; EC:3.5.4.5	dCMP deaminase; Cytidine deaminase	G3DSA:3.40.140.10 (GENE3D); IPR002125 (PFAM); G3DSA:3.40.50.300 (GENE3D); IPR015517 (PANTHER); PTHR11086:SF18 (PANTHER); IPR016192 (PROSITE_PATTERNS); IPR002125 (PROSITE_PROFILES); IPR035105 (CDD); IPR016193 (SUPERFAMILY)	F:GO:0003824; F:GO:0008270; F:GO:0016787	F:catalytic activity; F:zinc ion binding; F:hydrolase activity
150	CP008870.2	Pf-DR	II	ATPase	413	20	0	91.07	1	F:GO:0043167	F:ion binding			IPR041685 (PFAM); G3DSA:3.40.50.300 (GENE3D); G3DSA:3.40.50.300 (GENE3D); IPR014555 (PIRSF); IPR003959 (PFAM); PTHR40396 (PANTHER); IPR027417 (SUPERFAMILY)	F:GO:0005524	F:ATP binding
151	CP008870.2	Pf-DR	II	DUF4276 family protein	200	20	4.62E-145	84.59						IPR025455 (PFAM)	no GO terms	no GO terms
152	CP008872.2	Pf-tRNA-Gly	I	ATP-binding protein	466	20	0	66.71	1	F:GO:0043167	F:ion binding			IPR003959 (PFAM); PTHR43581:SF2 (PANTHER); PTHR43581 (PANTHER); CYTOPLASMIC_DOMAIN (PHOBIUS); CYTOPLASMIC_DOMAIN (PHOBIUS); TRANSMEMBRANE (PHOBIUS); NON_CYTOPLASMIC_DOMAIN (PHOBIUS); TRANSMEMBRANE (PHOBIUS); IPR027417 (SUPERFAMILY)	F:GO:0005524	F:ATP binding
153	CP008872.2	Pf-tRNA-Gly	I	HNH endonuclease	212	20	2.52E-156	73.76	1	F:GO:0004518	F:nuclease activity			G3DSA:3.30.40.60 (GENE3D); IPR003615 (PFAM)	no GO terms	no GO terms
154	CP008872.2	Pf-tRNA-Gly	I	---NA---	33									no IPS match	no IPS match	no IPS match
155	CP008872.2	Pf-tRNA-Gly	I	Uncharacterised protein	73	20	3.96E-48	89.56						no IPS match	no IPS match	no IPS match
156	CP008872.2	Pf-tRNA-Gly	I	DNA-binding protein	71	20	7.35E-45	93.24	1	F:GO:0003677	F:DNA binding			no IPS match	no IPS match	no IPS match
157	CP008872.2	Pf-tRNA-Gly	I	DNA-binding protein	77	20	4.93E-48	90.17	1	F:GO:0003677	F:DNA binding			G3DSA:1.10.260.40 (GENE3D)	no GO terms	no GO terms
158	CP008872.2	Pf-tRNA-Gly	I	pentapeptide repeat-containing protein	356	20	0	71.52	1	C:GO:0005575	C:cellular_component			IPR001646 (PFAM); G3DSA:2.160.20.100 (GENE3D); CYTOPLASMIC_DOMAIN (PHOBIUS); NON_CYTOPLASMIC_DOMAIN (PHOBIUS); TRANSMEMBRANE (PHOBIUS); TRANSMEMBRANE (PHOBIUS); TRANSMEMBRANE (PHOBIUS); CYTOPLASMIC_DOMAIN (PHOBIUS); NON_CYTOPLASMIC_DOMAIN (PHOBIUS); CYTOPLASMIC_DOMAIN (PHOBIUS); SSF141571 (SUPERFAMILY); TMhelix (TMHMM); TMhelix (TMHMM); TMhelix (TMHMM)	no GO terms	no GO terms
159	CP008872.2	Pf-tRNA-Gly	I	nucleotidyltransferase domain-containing protein	231	20	1.99E-171	66.05	1	F:GO:0003674	F:molecular_function			G3DSA:3.30.460.10 (GENE3D); cd05403 (CDD); SSF81301 (SUPERFAMILY)	no GO terms	no GO terms
160	CP008872.2	Pf-tRNA-Met	I	Uncharacterised protein	145	20	4.02E-100	94.17	1	C:GO:0005575	C:cellular_component			TRANSMEMBRANE (PHOBIUS); TRANSMEMBRANE (PHOBIUS); CYTOPLASMIC_DOMAIN (PHOBIUS); NON_CYTOPLASMIC_DOMAIN (PHOBIUS); CYTOPLASMIC_DOMAIN (PHOBIUS); TMhelix (TMHMM); TMhelix (TMHMM)	no GO terms	no GO terms
161	CP008872.2	Pf-tRNA-Met	I	Uncharacterised protein	111	20	4.51E-75	97.48						mobidb-lite (MOBIDB_LITE); mobidb-lite (MOBIDB_LITE); SIGNAL_PEPTIDE_H_REGION (PHOBIUS); SIGNAL_PEPTIDE_N_REGION (PHOBIUS); SIGNAL_PEPTIDE_C_REGION (PHOBIUS); NON_CYTOPLASMIC_DOMAIN (PHOBIUS); SIGNAL_PEPTIDE (PHOBIUS); SignalP-TM (SIGNALP_GRAM_POSITIVE); SignalP-noTM (SIGNALP_EUK); SignalP-noTM (SIGNALP_GRAM_NEGATIVE)	no GO terms	no GO terms
162	CP008872.2	Pf-tRNA-Met	I	Uncharacterised protein	256	20	0	66.62	1	C:GO:0005575	C:cellular_component			Coil (COILS); Coil (COILS); TRANSMEMBRANE (PHOBIUS); CYTOPLASMIC_DOMAIN (PHOBIUS); CYTOPLASMIC_DOMAIN (PHOBIUS); NON_CYTOPLASMIC_DOMAIN (PHOBIUS); TRANSMEMBRANE (PHOBIUS); TMhelix (TMHMM); TMhelix (TMHMM)	no GO terms	no GO terms
163	CP008872.2	Pf-tRNA-Met	I	DNA-binding protein	89	20	1.43E-58	98.14	1	F:GO:0003677	F:DNA binding			G3DSA:1.10.260.40 (GENE3D)	no GO terms	no GO terms
164	CP008872.2	Pf-tRNA-Met	I	hypothetical protein	111	20	1.44E-75	97.66						SIGNAL_PEPTIDE_H_REGION (PHOBIUS); SIGNAL_PEPTIDE_N_REGION (PHOBIUS); SIGNAL_PEPTIDE_C_REGION (PHOBIUS); NON_CYTOPLASMIC_DOMAIN (PHOBIUS); SIGNAL_PEPTIDE (PHOBIUS); SignalP-noTM (SIGNALP_EUK); SignalP-noTM (SIGNALP_GRAM_NEGATIVE); SignalP-TM (SIGNALP_GRAM_POSITIVE)	no GO terms	no GO terms
165	CP008872.2	Pf-tRNA-Met	I	DUF3732 domain-containing protein	641	20	0	68						Coil (COILS); Coil (COILS); Coil (COILS); Coil (COILS); IPR022205 (PFAM); IPR038729 (PFAM); G3DSA:3.40.50.300 (GENE3D); CYTOPLASMIC_DOMAIN (PHOBIUS); NON_CYTOPLASMIC_DOMAIN (PHOBIUS); TRANSMEMBRANE (PHOBIUS); IPR027417 (SUPERFAMILY)	no GO terms	no GO terms
166	CP008872.2	Pf-tRNA-Met	I	hypothetical protein	155	20	3.17E-107	60.65						TRANSMEMBRANE (PHOBIUS); NON_CYTOPLASMIC_DOMAIN (PHOBIUS); CYTOPLASMIC_DOMAIN (PHOBIUS)	no GO terms	no GO terms
167	CP008872.2	Pf-tRNA-Met	I	Uncharacterised protein	345	20	0	61.67						no IPS match	no IPS match	no IPS match
168	CP008872.2	Pf-tRNA-Met	I	restriction endonuclease	56	20	4.82E-18	78.57	7	P:GO:0006259; P:GO:0006950; P:GO:0051276; F:GO:0003677; F:GO:0004518; F:GO:0016853; C:GO:0005694	P:DNA metabolic process; P:response to stress; P:chromosome organization; F:DNA binding; F:nuclease activity; F:isomerase activity; C:chromosome			TRANSMEMBRANE (PHOBIUS); NON_CYTOPLASMIC_DOMAIN (PHOBIUS); CYTOPLASMIC_DOMAIN (PHOBIUS); TMhelix (TMHMM)	no GO terms	no GO terms
169	CP008872.2	Pf-tRNA-Sec	II	hypothetical protein	91	20	2.87E-60	66.17	1	C:GO:0005575	C:cellular_component			SIGNAL_PEPTIDE (PHOBIUS); SIGNAL_PEPTIDE_N_REGION (PHOBIUS); NON_CYTOPLASMIC_DOMAIN (PHOBIUS); SIGNAL_PEPTIDE_H_REGION (PHOBIUS); SIGNAL_PEPTIDE_C_REGION (PHOBIUS); SignalP-TM (SIGNALP_GRAM_POSITIVE); SignalP-noTM (SIGNALP_EUK)	no GO terms	no GO terms
170	CP008872.2	Pf-tRNA-Sec	II	RES domain-containing protein	342	20	0	89.13						IPR014914 (SMART); IPR014914 (PFAM)	no GO terms	no GO terms
171	CP008872.2	Pf-tRNA-Sec	II	sce7725 family protein	302	20	0	84.95						no IPS match	no IPS match	no IPS match
172	CP008872.2	Pf-tRNA-Sec	II	sce7726 family protein	295	20	0	81.23						no IPS match	no IPS match	no IPS match
173	CP008873.1	Pf-tRNA-Gly	II	type II toxin-antitoxin system RelE/ParE family toxin	115	20	5.90E-77	98.35						IPR007712 (PFAM); IPR035093 (G3DSA:3.30.2310.GENE3D)	no GO terms	no GO terms
174	CP008873.1	Pf-tRNA-Gly	II	type II toxin-antitoxin system Phd/YefM family antitoxin	83	20	6.81E-53	96.37						PTHR33713:SF7 (PANTHER); PTHR33713 (PANTHER); IPR036165 (SUPERFAMILY)	no GO terms	no GO terms
175	CP008873.1	Pf-tRNA-Gly	II	Uncharacterised protein	230	20	2.58E-173	96.26	1	C:GO:0005575	C:cellular_component			mobidb-lite (MOBIDB_LITE); mobidb-lite (MOBIDB_LITE); mobidb-lite (MOBIDB_LITE); TRANSMEMBRANE (PHOBIUS); NON_CYTOPLASMIC_DOMAIN (PHOBIUS); CYTOPLASMIC_DOMAIN (PHOBIUS); TMhelix (TMHMM)	no GO terms	no GO terms
176	CP008873.1	Pf-tRNA-Gly	II	Uncharacterised protein	504	20	0	74.14						mobidb-lite (MOBIDB_LITE)	no GO terms	no GO terms
177	CP008873.1	Pf-tRNA-Met	II	MULTISPECIES: hypothetical protein	136	20	1.03E-93	98.01	1	C:GO:0005575	C:cellular_component			CYTOPLASMIC_DOMAIN (PHOBIUS); TRANSMEMBRANE (PHOBIUS); CYTOPLASMIC_DOMAIN (PHOBIUS); NON_CYTOPLASMIC_DOMAIN (PHOBIUS); NON_CYTOPLASMIC_DOMAIN (PHOBIUS); TRANSMEMBRANE (PHOBIUS); TRANSMEMBRANE (PHOBIUS); TMhelix (TMHMM); TMhelix (TMHMM); TMhelix (TMHMM)	no GO terms	no GO terms
178	CP008873.1	Pf-tRNA-Met	II	Cro/CI family transcriptional regulator	264	20	0	86.3	7	P:GO:0001666; P:GO:0019217; P:GO:0045892; P:GO:0045926; P:GO:0045927; F:GO:0003677	P:response to hypoxia; P:regulation of fatty acid metabolic process; P:negative regulation of transcription, DNA-templated; P:negative regulation of growth; P:positive regulation of growth; F:DNA binding			IPR001387 (SMART); IPR007374 (SMART); IPR007374 (PFAM); IPR001387 (PFAM); G3DSA:1.10.260.40 (GENE3D); G3DSA:2.30.130.30 (GENE3D); IPR001387 (PROSITE_PROFILES); IPR001387 (CDD); IPR015947 (SUPERFAMILY); IPR010982 (SUPERFAMILY)	F:GO:0003677	F:DNA binding
179	CP008873.1	Pf-tRNA-Met	II	GNAT family N-acetyltransferase	512	20	0	98.42	1	F:GO:0016746	F:transferase activity, transferring acyl groups	EC:2.3.1.5	Arylamine N-acetyltransferase	G3DSA:3.40.630.30 (GENE3D); IPR000182 (PFAM); IPR000182 (PROSITE_PROFILES); cd18699 (CDD); IPR016181 (SUPERFAMILY)	F:GO:0008080	F:N-acetyltransferase activity
180	CP008873.1	Pf-tRNA-Met	II	AAA family ATPase	175	20	2.76E-124	83.12				EC:2.7.4.3	Adenylate kinase	PF13207 (PFAM); G3DSA:3.40.50.300 (GENE3D); IPR027417 (SUPERFAMILY)	no GO terms	no GO terms
181	CP010555.1	Pf-tRNA-Met	I	hypothetical protein	136	20	1.37E-94	99.74	1	C:GO:0005575	C:cellular_component			CYTOPLASMIC_DOMAIN (PHOBIUS); CYTOPLASMIC_DOMAIN (PHOBIUS); TRANSMEMBRANE (PHOBIUS); NON_CYTOPLASMIC_DOMAIN (PHOBIUS); NON_CYTOPLASMIC_DOMAIN (PHOBIUS); TRANSMEMBRANE (PHOBIUS); TRANSMEMBRANE (PHOBIUS); TMhelix (TMHMM); TMhelix (TMHMM)	no GO terms	no GO terms
182	CP010555.1	Pf-tRNA-Met	I	hypothetical protein	111	20	1.24E-74	98.56						mobidb-lite (MOBIDB_LITE); mobidb-lite (MOBIDB_LITE); SIGNAL_PEPTIDE_H_REGION (PHOBIUS); SIGNAL_PEPTIDE_N_REGION (PHOBIUS); SIGNAL_PEPTIDE_C_REGION (PHOBIUS); NON_CYTOPLASMIC_DOMAIN (PHOBIUS); SIGNAL_PEPTIDE (PHOBIUS); SignalP-TM (SIGNALP_GRAM_POSITIVE); SignalP-noTM (SIGNALP_GRAM_NEGATIVE); SignalP-noTM (SIGNALP_EUK)	no GO terms	no GO terms
183	CP010555.1	Pf-tRNA-Met	I	Uncharacterised protein	363	20	0	72.55	1	C:GO:0005575	C:cellular_component			CYTOPLASMIC_DOMAIN (PHOBIUS); TRANSMEMBRANE (PHOBIUS); NON_CYTOPLASMIC_DOMAIN (PHOBIUS); CYTOPLASMIC_DOMAIN (PHOBIUS); TRANSMEMBRANE (PHOBIUS); TMhelix (TMHMM); TMhelix (TMHMM)	no GO terms	no GO terms
184	CP010555.1	Pf-tRNA-Met	I	hypothetical protein	130	20	2.19E-89	98.14						no IPS match	no IPS match	no IPS match
185	CP011317.1	Pf-tRNA-Met	I	hypothetical protein	136	20	1.37E-94	99.74	1	C:GO:0005575	C:cellular_component			CYTOPLASMIC_DOMAIN (PHOBIUS); CYTOPLASMIC_DOMAIN (PHOBIUS); TRANSMEMBRANE (PHOBIUS); NON_CYTOPLASMIC_DOMAIN (PHOBIUS); NON_CYTOPLASMIC_DOMAIN (PHOBIUS); TRANSMEMBRANE (PHOBIUS); TRANSMEMBRANE (PHOBIUS); TMhelix (TMHMM); TMhelix (TMHMM)	no GO terms	no GO terms
186	CP011317.1	Pf-tRNA-Met	I	hypothetical protein	111	20	1.24E-74	98.56						mobidb-lite (MOBIDB_LITE); mobidb-lite (MOBIDB_LITE); SIGNAL_PEPTIDE_H_REGION (PHOBIUS); SIGNAL_PEPTIDE_N_REGION (PHOBIUS); SIGNAL_PEPTIDE_C_REGION (PHOBIUS); NON_CYTOPLASMIC_DOMAIN (PHOBIUS); SIGNAL_PEPTIDE (PHOBIUS); SignalP-noTM (SIGNALP_GRAM_NEGATIVE); SignalP-noTM (SIGNALP_EUK); SignalP-TM (SIGNALP_GRAM_POSITIVE)	no GO terms	no GO terms
187	CP011317.1	Pf-tRNA-Met	I	Uncharacterised protein	363	20	0	72.55	1	C:GO:0005575	C:cellular_component			CYTOPLASMIC_DOMAIN (PHOBIUS); TRANSMEMBRANE (PHOBIUS); NON_CYTOPLASMIC_DOMAIN (PHOBIUS); CYTOPLASMIC_DOMAIN (PHOBIUS); TRANSMEMBRANE (PHOBIUS); TMhelix (TMHMM); TMhelix (TMHMM)	no GO terms	no GO terms
188	CP011317.1	Pf-tRNA-Met	I	hypothetical protein	130	20	2.19E-89	98.14						no IPS match	no IPS match	no IPS match
189	CP011857.1	Pf-tRNA-Met	II	MULTISPECIES: hypothetical protein	136	20	1.03E-93	98.01	1	C:GO:0005575	C:cellular_component			CYTOPLASMIC_DOMAIN (PHOBIUS); TRANSMEMBRANE (PHOBIUS); CYTOPLASMIC_DOMAIN (PHOBIUS); NON_CYTOPLASMIC_DOMAIN (PHOBIUS); NON_CYTOPLASMIC_DOMAIN (PHOBIUS); TRANSMEMBRANE (PHOBIUS); TRANSMEMBRANE (PHOBIUS); TMhelix (TMHMM); TMhelix (TMHMM); TMhelix (TMHMM)	no GO terms	no GO terms
190	CP011857.1	Pf-tRNA-Met	II	Cro/CI family transcriptional regulator	264	20	0	86.3	7	P:GO:0001666; P:GO:0019217; P:GO:0045892; P:GO:0045926; P:GO:0045927; F:GO:0003677	P:response to hypoxia; P:regulation of fatty acid metabolic process; P:negative regulation of transcription, DNA-templated; P:negative regulation of growth; P:positive regulation of growth; F:DNA binding			IPR001387 (SMART); IPR007374 (SMART); IPR007374 (PFAM); IPR001387 (PFAM); G3DSA:1.10.260.40 (GENE3D); G3DSA:2.30.130.30 (GENE3D); IPR001387 (PROSITE_PROFILES); IPR001387 (CDD); IPR015947 (SUPERFAMILY); IPR010982 (SUPERFAMILY)	F:GO:0003677	F:DNA binding
191	CP011857.1	Pf-tRNA-Met	II	GNAT family N-acetyltransferase	512	20	0	98.42	1	F:GO:0016746	F:transferase activity, transferring acyl groups	EC:2.3.1.5	Arylamine N-acetyltransferase	G3DSA:3.40.630.30 (GENE3D); IPR000182 (PFAM); IPR000182 (PROSITE_PROFILES); cd18699 (CDD); IPR016181 (SUPERFAMILY)	F:GO:0008080	F:N-acetyltransferase activity
192	CP011857.1	Pf-tRNA-Met	II	AAA family ATPase	175	20	2.76E-124	83.12				EC:2.7.4.3	Adenylate kinase	PF13207 (PFAM); G3DSA:3.40.50.300 (GENE3D); IPR027417 (SUPERFAMILY)	no GO terms	no GO terms
193	CP011857.1b	Pf-tRNA-Gly	II	type II toxin-antitoxin system RelE/ParE family toxin	115	20	5.90E-77	98.35						IPR007712 (PFAM); IPR035093 (G3DSA:3.30.2310.GENE3D)	no GO terms	no GO terms
194	CP011857.1b	Pf-tRNA-Gly	II	type II toxin-antitoxin system Phd/YefM family antitoxin	83	20	6.81E-53	96.37						PTHR33713:SF7 (PANTHER); PTHR33713 (PANTHER); IPR036165 (SUPERFAMILY)	no GO terms	no GO terms
195	CP011857.1b	Pf-tRNA-Gly	II	Uncharacterised protein	230	20	2.58E-173	96.26	1	C:GO:0005575	C:cellular_component			mobidb-lite (MOBIDB_LITE); mobidb-lite (MOBIDB_LITE); mobidb-lite (MOBIDB_LITE); TRANSMEMBRANE (PHOBIUS); NON_CYTOPLASMIC_DOMAIN (PHOBIUS); CYTOPLASMIC_DOMAIN (PHOBIUS); TMhelix (TMHMM)	no GO terms	no GO terms
196	CP011857.1b	Pf-tRNA-Gly	II	Uncharacterised protein	504	20	0	74.14						mobidb-lite (MOBIDB_LITE)	no GO terms	no GO terms
197	CP012001.1	Pf-DR	I	hypothetical protein	137	20	3.07E-96	84.52	1	C:GO:0005575	C:cellular_component			Coil (COILS); mobidb-lite (MOBIDB_LITE); mobidb-lite (MOBIDB_LITE); mobidb-lite (MOBIDB_LITE)	no GO terms	no GO terms
198	CP012001.1	Pf-DR	I	RNA-directed DNA polymerase	325	20	0	76.85	4	P:GO:0006259; P:GO:0009058; F:GO:0003723; F:GO:0016779	P:DNA metabolic process; P:biosynthetic process; F:RNA binding; F:nucleotidyltransferase activity	EC:2.7.7.49	RNA-directed DNA polymerase	IPR000123 (PRINTS); IPR000477 (PFAM); PTHR34047:SF6 (PANTHER); PTHR34047 (PANTHER); cd03487 (CDD)	P:GO:0006278; F:GO:0003723; F:GO:0003964	P:RNA-dependent DNA biosynthetic process; F:RNA binding; F:RNA-directed DNA polymerase activity
199	CP012001.1	Pf-DR	I	helix-turn-helix transcriptional regulator	108	20	1.77E-71	87.52	1	F:GO:0003677	F:DNA binding			IPR001387 (SMART); IPR001387 (PFAM); G3DSA:1.10.260.40 (GENE3D); PTHR46558 (PANTHER); IPR001387 (PROSITE_PROFILES); IPR001387 (CDD); IPR010982 (SUPERFAMILY)	F:GO:0003677	F:DNA binding
200	CP012001.1	Pf-DR	I	DNA polymerase III subunit alpha	55	3	1.04E-10	55.76	1	C:GO:0005575	C:cellular_component			CYTOPLASMIC_DOMAIN (PHOBIUS); NON_CYTOPLASMIC_DOMAIN (PHOBIUS); TRANSMEMBRANE (PHOBIUS); TMhelix (TMHMM)	no GO terms	no GO terms
201	CP012001.1	Pf-tRNA-Sec	II	Uncharacterised protein	227	20	3.23E-161	79.68						no IPS match	no IPS match	no IPS match
202	CP012066.1	Pf-tRNA-Met	I	Uncharacterised protein	145	20	6.04E-100	95.38	1	C:GO:0005575	C:cellular_component			TRANSMEMBRANE (PHOBIUS); CYTOPLASMIC_DOMAIN (PHOBIUS); TRANSMEMBRANE (PHOBIUS); CYTOPLASMIC_DOMAIN (PHOBIUS); NON_CYTOPLASMIC_DOMAIN (PHOBIUS); TMhelix (TMHMM); TMhelix (TMHMM)	no GO terms	no GO terms
203	CP012066.1	Pf-tRNA-Met	I	sce7726 family protein	285	20	0	74.17						no IPS match	no IPS match	no IPS match
204	CP012066.1	Pf-tRNA-Met	I	sce7725 family protein	317	20	0	71.61						no IPS match	no IPS match	no IPS match
205	CP012066.1	Pf-tRNA-Met	I	DUF2290 domain-containing protein	224	20	3.74E-165	67.64						IPR018742 (PFAM)	no GO terms	no GO terms
206	CP012066.1	Pf-tRNA-Met	I	Superfamily I DNA and RNA helicases	735	20	0	79.23	4	P:GO:0051276; F:GO:0003677; F:GO:0004386; F:GO:0043167	P:chromosome organization; F:DNA binding; F:helicase activity; F:ion binding	EC:3.6.1.3; EC:3.6.4.12; EC:3.6.1.15	Adenosinetriphosphatase; DNA helicase; Nucleoside-triphosphate phosphatase	G3DSA:3.40.50.300 (GENE3D); IPR027785 (PFAM); G3DSA:3.40.50.300 (GENE3D); PTHR11070:SF3 (PANTHER); IPR000212 (PANTHER); cd18807 (CDD); IPR027417 (SUPERFAMILY)	F:GO:0003677; F:GO:0003678; F:GO:0005524	F:DNA binding; F:DNA helicase activity; F:ATP binding
207	CP012066.1	Pf-tRNA-Met	I	ankyrin repeat domain-containing protein	128	20	2.01E-85	63.98	1	F:GO:0003674	F:molecular_function			IPR002110 (SMART); PF13637 (PFAM); IPR036770 (G3DSA:1.25.40.GENE3D); mobidb-lite (MOBIDB_LITE); PTHR24134 (PANTHER); IPR020683 (PROSITE_PROFILES); IPR002110 (PROSITE_PROFILES); IPR036770 (SUPERFAMILY)	F:GO:0005515	F:protein binding
208	CP012901.1b	Pf-tRNA-Gly	I	DUF4393 domain-containing protein	263	20	0	69.57						IPR025506 (PFAM); G3DSA:1.10.10.2280 (GENE3D)	no GO terms	no GO terms
209	CP012901.1b	Pf-tRNA-Gly	I	Uncharacterised protein	74	20	4.51E-47	86.4						no IPS match	no IPS match	no IPS match
210	CP013113.1	Pf-tRNA-Gly	II	type II toxin-antitoxin system RelE/ParE family toxin	115	20	8.96E-77	98.35						IPR035093 (G3DSA:3.30.2310.GENE3D); IPR007712 (PFAM)	no GO terms	no GO terms
211	CP013113.1	Pf-tRNA-Gly	II	type II toxin-antitoxin system Phd/YefM family antitoxin	83	20	6.81E-53	96.37						PTHR33713:SF7 (PANTHER); PTHR33713 (PANTHER); IPR036165 (SUPERFAMILY)	no GO terms	no GO terms
212	CP013113.1	Pf-tRNA-Gly	II	Uncharacterised protein	230	20	2.17E-172	95.22	1	C:GO:0005575	C:cellular_component			mobidb-lite (MOBIDB_LITE); mobidb-lite (MOBIDB_LITE); mobidb-lite (MOBIDB_LITE); mobidb-lite (MOBIDB_LITE); TRANSMEMBRANE (PHOBIUS); NON_CYTOPLASMIC_DOMAIN (PHOBIUS); CYTOPLASMIC_DOMAIN (PHOBIUS); TMhelix (TMHMM)	no GO terms	no GO terms
213	CP013113.1	Pf-tRNA-Gly	II	NYN domain-containing protein	219	20	2.08E-162	60.38						no IPS match	no IPS match	no IPS match
214	CP013113.1	Pf-tRNA-Gly	II	NACHT domain-containing protein	714	20	0	74.51						IPR027417 (SUPERFAMILY)	no GO terms	no GO terms
215	CP013113.1	Pf-tRNA-Met	II	hypothetical protein	136	20	1.37E-94	99.74	1	C:GO:0005575	C:cellular_component			NON_CYTOPLASMIC_DOMAIN (PHOBIUS); TRANSMEMBRANE (PHOBIUS); TRANSMEMBRANE (PHOBIUS); CYTOPLASMIC_DOMAIN (PHOBIUS); CYTOPLASMIC_DOMAIN (PHOBIUS); TRANSMEMBRANE (PHOBIUS); NON_CYTOPLASMIC_DOMAIN (PHOBIUS); TMhelix (TMHMM); TMhelix (TMHMM); TMhelix (TMHMM)	no GO terms	no GO terms
216	CP013113.1	Pf-tRNA-Met	II	hypothetical protein	111	20	4.18E-75	98.02						SIGNAL_PEPTIDE_H_REGION (PHOBIUS); SIGNAL_PEPTIDE_N_REGION (PHOBIUS); SIGNAL_PEPTIDE_C_REGION (PHOBIUS); NON_CYTOPLASMIC_DOMAIN (PHOBIUS); SIGNAL_PEPTIDE (PHOBIUS); SignalP-noTM (SIGNALP_EUK); SignalP-noTM (SIGNALP_GRAM_NEGATIVE); SignalP-TM (SIGNALP_GRAM_POSITIVE)	no GO terms	no GO terms
217	CP013113.1	Pf-tRNA-Met	II	AAA family ATPase	88	20	1.54E-40	74.72						no IPS match	no IPS match	no IPS match
218	CP013113.1	Pf-tRNA-Met	II	hypothetical protein	569	20	0	69.18						no IPS match	no IPS match	no IPS match
219	CP013113.1	Pf-tRNA-Met	II	ATP-binding protein	998	20	0	81.81	4	F:GO:0016301; F:GO:0043167	F:kinase activity; F:ion binding	EC:2.7.13.3	Histidine kinase	Coil (COILS); Coil (COILS); Coil (COILS); IPR003594 (SMART); IPR036890 (G3DSA:3.30.565.GENE3D); PF13589 (PFAM); IPR036890 (G3DSA:3.30.565.GENE3D); IPR003594 (PFAM); PTHR43065:SF26 (PANTHER); PTHR43065 (PANTHER); TRANSMEMBRANE (PHOBIUS); NON_CYTOPLASMIC_DOMAIN (PHOBIUS); CYTOPLASMIC_DOMAIN (PHOBIUS); IPR005467 (PROSITE_PROFILES); cd00075 (CDD); IPR036890 (SUPERFAMILY); IPR036890 (SUPERFAMILY)	no GO terms	no GO terms
220	CP013113.1	Pf-tRNA-Met	II	very short patch repair endonuclease	148	20	3.58E-106	79.75	3	P:GO:0006259; P:GO:0006950; F:GO:0004518	P:DNA metabolic process; P:response to stress; F:nuclease activity			IPR004603 (PFAM); G3DSA:3.40.960.10 (GENE3D); IPR004603 (TIGRFAM); mobidb-lite (MOBIDB_LITE); IPR004603 (CDD); IPR011335 (SUPERFAMILY)	P:GO:0006298; F:GO:0004519	P:mismatch repair; F:endonuclease activity
221	CP013113.1	Pf-tRNA-Met	II	DNA cytosine methyltransferase	341	20	0	88.72	2	P:GO:0090116; F:GO:0003886	P:C-5 methylation of cytosine; F:DNA (cytosine-5-)-methyltransferase activity	EC:2.1.1.37	DNA (cytosine-5-)-methyltransferase	IPR001525 (PRINTS); G3DSA:3.90.120.10 (GENE3D); IPR001525 (PFAM); IPR001525 (TIGRFAM); G3DSA:3.40.50.150 (GENE3D); PTHR10629:SF11 (PANTHER); PTHR10629 (PANTHER); IPR018117 (PROSITE_PATTERNS); IPR031303 (PROSITE_PATTERNS); IPR001525 (PROSITE_PROFILES); IPR029063 (SUPERFAMILY)	F:GO:0008168	F:methyltransferase activity
222	CP013113.1	Pf-DR	II	hypothetical protein	207	20	3.11E-150	84.4						Coil (COILS); IPR040704 (PFAM)	no GO terms	no GO terms
223	CP013113.1	Pf-DR	II	restriction endonuclease	328	20	0	90.12						no IPS match	no IPS match	no IPS match
224	CP013477.1	Pf-tmRNA	I	Uncharacterised protein	75	20	9.13E-48	78.93						no IPS match	no IPS match	no IPS match
225	CP013477.1	Pf-tmRNA	I	Uncharacterised protein	232	20	3.10E-174	98.36	1	C:GO:0005575	C:cellular_component			mobidb-lite (MOBIDB_LITE); mobidb-lite (MOBIDB_LITE); mobidb-lite (MOBIDB_LITE); TRANSMEMBRANE (PHOBIUS); NON_CYTOPLASMIC_DOMAIN (PHOBIUS); CYTOPLASMIC_DOMAIN (PHOBIUS); TMhelix (TMHMM)	no GO terms	no GO terms
226	CP013477.1	Pf-tmRNA	I	ATP-binding protein	412	20	0	76.22						IPR027417 (SUPERFAMILY)	no GO terms	no GO terms
227	CP013478.1	Pf-tmRNA	I	Uncharacterised protein	75	20	9.13E-48	78.93						no IPS match	no IPS match	no IPS match
228	CP013478.1	Pf-tmRNA	I	Uncharacterised protein	232	20	3.10E-174	98.36	1	C:GO:0005575	C:cellular_component			mobidb-lite (MOBIDB_LITE); mobidb-lite (MOBIDB_LITE); mobidb-lite (MOBIDB_LITE); TRANSMEMBRANE (PHOBIUS); NON_CYTOPLASMIC_DOMAIN (PHOBIUS); CYTOPLASMIC_DOMAIN (PHOBIUS); TMhelix (TMHMM)	no GO terms	no GO terms
229	CP013478.1	Pf-tmRNA	I	ATP-binding protein	412	20	0	76.22						IPR027417 (SUPERFAMILY)	no GO terms	no GO terms
230	CP013479.1	Pf-tmRNA	I	Uncharacterised protein	75	20	9.13E-48	78.93						no IPS match	no IPS match	no IPS match
231	CP013479.1	Pf-tmRNA	I	Uncharacterised protein	232	20	3.10E-174	98.36	1	C:GO:0005575	C:cellular_component			mobidb-lite (MOBIDB_LITE); mobidb-lite (MOBIDB_LITE); mobidb-lite (MOBIDB_LITE); TRANSMEMBRANE (PHOBIUS); NON_CYTOPLASMIC_DOMAIN (PHOBIUS); CYTOPLASMIC_DOMAIN (PHOBIUS); TMhelix (TMHMM)	no GO terms	no GO terms
232	CP013479.1	Pf-tmRNA	I	ATP-binding protein	412	20	0	76.22						IPR027417 (SUPERFAMILY)	no GO terms	no GO terms
233	CP013696.1	Pf-tRNA-Gly	II	GntR family transcriptional regulator	74	12	0.100572	53.29	4	P:GO:0009058; P:GO:0034641; F:GO:0003677; F:GO:0003700	P:biosynthetic process; P:cellular nitrogen compound metabolic process; F:DNA binding; F:DNA-binding transcription factor activity			mobidb-lite (MOBIDB_LITE); mobidb-lite (MOBIDB_LITE)	no GO terms	no GO terms
234	CP013696.1	Pf-tRNA-Gly	II	Uncharacterised protein	230	20	1.02E-172	95.57	1	C:GO:0005575	C:cellular_component			mobidb-lite (MOBIDB_LITE); mobidb-lite (MOBIDB_LITE); NON_CYTOPLASMIC_DOMAIN (PHOBIUS); TRANSMEMBRANE (PHOBIUS); CYTOPLASMIC_DOMAIN (PHOBIUS); TMhelix (TMHMM)	no GO terms	no GO terms
235	CP013696.1	Pf-tRNA-Gly	II	RloB domain-containing protein	208	20	1.23E-153	71.97						IPR025591 (PFAM)	no GO terms	no GO terms
236	CP013696.1	Pf-tRNA-Gly	II	ATP-binding protein	429	20	0	73.51	1	F:GO:0043167	F:ion binding			IPR003959 (PFAM); G3DSA:3.40.50.300 (GENE3D); PTHR40396 (PANTHER); IPR027417 (SUPERFAMILY)	F:GO:0005524	F:ATP binding
237	CP013989.1	Pf-tRNA-Gly	II	type II toxin-antitoxin system RelE/ParE family toxin	115	20	8.96E-77	98.35						IPR035093 (G3DSA:3.30.2310.GENE3D); IPR007712 (PFAM)	no GO terms	no GO terms
238	CP013989.1	Pf-tRNA-Gly	II	type II toxin-antitoxin system Phd/YefM family antitoxin	83	20	6.81E-53	96.37						PTHR33713:SF7 (PANTHER); PTHR33713 (PANTHER); IPR036165 (SUPERFAMILY)	no GO terms	no GO terms
239	CP013989.1	Pf-tRNA-Gly	II	Uncharacterised protein	216	20	9.66E-161	65.56						no IPS match	no IPS match	no IPS match
240	CP013989.1	Pf-tRNA-Gly	II	Uncharacterised protein	318	20	0	68.05						no IPS match	no IPS match	no IPS match
241	CP013989.1	Pf-tRNA-Sec	II	hypothetical protein	230	20	4.05E-165	55.26	1	C:GO:0005575	C:cellular_component			CYTOPLASMIC_DOMAIN (PHOBIUS); TRANSMEMBRANE (PHOBIUS); NON_CYTOPLASMIC_DOMAIN (PHOBIUS); CYTOPLASMIC_DOMAIN (PHOBIUS); TRANSMEMBRANE (PHOBIUS); NON_CYTOPLASMIC_DOMAIN (PHOBIUS); TRANSMEMBRANE (PHOBIUS); TMhelix (TMHMM); TMhelix (TMHMM); TMhelix (TMHMM)	no GO terms	no GO terms
242	CP013989.1	Pf-tRNA-Sec	II	type 1 glutamine amidotransferase	119	7	2.02E-83	80.02	1	P:GO:0006520	P:cellular amino acid metabolic process			no IPS match	no IPS match	no IPS match
243	CP013993.1	Pf-tRNA-Gly	I	Fis family transcriptional regulator	369	20	0	88.35	2	F:GO:0003677; F:GO:0043167	F:DNA binding; F:ion binding			Coil (COILS); IPR003593 (SMART); G3DSA:3.40.50.300 (GENE3D); IPR003959 (PFAM); IPR025246 (PFAM); PTHR23073:SF29 (PANTHER); PTHR23073 (PANTHER); cd00009 (CDD); IPR027417 (SUPERFAMILY)	F:GO:0005524	F:ATP binding
244	CP013993.1	Pf-tRNA-Gly	I	Uncharacterised protein	74	20	3.18E-47	84.96						no IPS match	no IPS match	no IPS match
245	CP013993.1	Pf-tRNA-Gly	I	Uncharacterised protein	230	20	3.91E-173	96.98	1	C:GO:0005575	C:cellular_component			mobidb-lite (MOBIDB_LITE); mobidb-lite (MOBIDB_LITE); mobidb-lite (MOBIDB_LITE); TRANSMEMBRANE (PHOBIUS); NON_CYTOPLASMIC_DOMAIN (PHOBIUS); CYTOPLASMIC_DOMAIN (PHOBIUS); TMhelix (TMHMM)	no GO terms	no GO terms
246	CP013993.1	Pf-tRNA-Gly	I	Uncharacterised protein	165	20	5.14E-96	63.27	1	C:GO:0005575	C:cellular_component			CYTOPLASMIC_DOMAIN (PHOBIUS); NON_CYTOPLASMIC_DOMAIN (PHOBIUS); TRANSMEMBRANE (PHOBIUS)	no GO terms	no GO terms
247	CP013993.1	Pf-tRNA-Gly	I	S8 family peptidase	732	20	0	88.19	1	F:GO:0008233	F:peptidase activity	EC:3.4.21	Acting on peptide bonds (peptidases)	IPR036852 (G3DSA:3.40.50.GENE3D); IPR000209 (PFAM); IPR034074 (CDD); IPR036852 (SUPERFAMILY)	P:GO:0006508; F:GO:0004252	P:proteolysis; F:serine-type endopeptidase activity
248	CP014866.1	Pf-tRNA-Met	I	hypothetical protein	136	20	1.76E-94	99.74	1	C:GO:0005575	C:cellular_component			CYTOPLASMIC_DOMAIN (PHOBIUS); CYTOPLASMIC_DOMAIN (PHOBIUS); TRANSMEMBRANE (PHOBIUS); NON_CYTOPLASMIC_DOMAIN (PHOBIUS); NON_CYTOPLASMIC_DOMAIN (PHOBIUS); TRANSMEMBRANE (PHOBIUS); TRANSMEMBRANE (PHOBIUS); TMhelix (TMHMM); TMhelix (TMHMM)	no GO terms	no GO terms
249	CP014866.1	Pf-tRNA-Met	I	Uncharacterised protein	112	20	7.77E-76	96.9						mobidb-lite (MOBIDB_LITE); mobidb-lite (MOBIDB_LITE); SIGNAL_PEPTIDE_H_REGION (PHOBIUS); NON_CYTOPLASMIC_DOMAIN (PHOBIUS); SIGNAL_PEPTIDE_N_REGION (PHOBIUS); SIGNAL_PEPTIDE_C_REGION (PHOBIUS); SIGNAL_PEPTIDE (PHOBIUS); SignalP-noTM (SIGNALP_EUK); SignalP-noTM (SIGNALP_GRAM_NEGATIVE); SignalP-TM (SIGNALP_GRAM_POSITIVE)	no GO terms	no GO terms
250	CP014866.1	Pf-tRNA-Met	I	RNA-directed DNA polymerase	356	20	0	78.24	4	P:GO:0006259; P:GO:0009058; F:GO:0003723; F:GO:0016779	P:DNA metabolic process; P:biosynthetic process; F:RNA binding; F:nucleotidyltransferase activity	EC:2.7.7.49	RNA-directed DNA polymerase	IPR000123 (PRINTS); IPR000477 (PFAM); PTHR34047:SF6 (PANTHER); PTHR34047 (PANTHER); IPR000477 (PROSITE_PROFILES); cd03487 (CDD)	P:GO:0006278; F:GO:0003723; F:GO:0003964	P:RNA-dependent DNA biosynthetic process; F:RNA binding; F:RNA-directed DNA polymerase activity
251	CP014866.1	Pf-tRNA-Met	I	phosphoribosyl 1,2-cyclic phosphodiesterase	72	20	4.71E-45	66.04	1	C:GO:0005575	C:cellular_component			NON_CYTOPLASMIC_DOMAIN (PHOBIUS); TRANSMEMBRANE (PHOBIUS); CYTOPLASMIC_DOMAIN (PHOBIUS)	no GO terms	no GO terms
252	CP014999.1	Pf-tRNA-Gly	II	type II toxin-antitoxin system RelE/ParE family toxin	115	20	1.69E-76	97.87						IPR035093 (G3DSA:3.30.2310.GENE3D); IPR007712 (PFAM)	no GO terms	no GO terms
253	CP014999.1	Pf-tRNA-Gly	II	type II toxin-antitoxin system Phd/YefM family antitoxin	83	20	6.81E-53	96.37						PTHR33713:SF7 (PANTHER); PTHR33713 (PANTHER); IPR036165 (SUPERFAMILY)	no GO terms	no GO terms
254	CP014999.1	Pf-tRNA-Gly	II	hypothetical protein	155	20	2.89E-104	76.22	1	C:GO:0005575	C:cellular_component			TRANSMEMBRANE (PHOBIUS); NON_CYTOPLASMIC_DOMAIN (PHOBIUS); TRANSMEMBRANE (PHOBIUS); TRANSMEMBRANE (PHOBIUS); CYTOPLASMIC_DOMAIN (PHOBIUS); CYTOPLASMIC_DOMAIN (PHOBIUS); CYTOPLASMIC_DOMAIN (PHOBIUS); TRANSMEMBRANE (PHOBIUS); NON_CYTOPLASMIC_DOMAIN (PHOBIUS); TMhelix (TMHMM); TMhelix (TMHMM); TMhelix (TMHMM); TMhelix (TMHMM)	no GO terms	no GO terms
255	CP014999.1	Pf-tRNA-Gly	II	Uncharacterised protein	242	20	1.41E-174	96.08	1	C:GO:0005575	C:cellular_component			mobidb-lite (MOBIDB_LITE); mobidb-lite (MOBIDB_LITE); mobidb-lite (MOBIDB_LITE); mobidb-lite (MOBIDB_LITE); mobidb-lite (MOBIDB_LITE); TRANSMEMBRANE (PHOBIUS); CYTOPLASMIC_DOMAIN (PHOBIUS); NON_CYTOPLASMIC_DOMAIN (PHOBIUS); TMhelix (TMHMM)	no GO terms	no GO terms
256	CP014999.1	Pf-tRNA-Gly	II	Uncharacterised protein	200	20	1.75E-145	70.47						no IPS match	no IPS match	no IPS match
257	CP014999.1	Pf-tRNA-Gly	II	Uncharacterised protein	211	20	4.45E-155	73.36						no IPS match	no IPS match	no IPS match
258	CP015001.1	Pf-tRNA-Gly	II	type II toxin-antitoxin system RelE/ParE family toxin	115	20	1.69E-76	97.87						IPR035093 (G3DSA:3.30.2310.GENE3D); IPR007712 (PFAM)	no GO terms	no GO terms
259	CP015001.1	Pf-tRNA-Gly	II	type II toxin-antitoxin system Phd/YefM family antitoxin	83	20	6.81E-53	96.37						PTHR33713:SF7 (PANTHER); PTHR33713 (PANTHER); IPR036165 (SUPERFAMILY)	no GO terms	no GO terms
260	CP015001.1	Pf-tRNA-Gly	II	Uncharacterised protein	242	20	1.41E-174	96.08	1	C:GO:0005575	C:cellular_component			mobidb-lite (MOBIDB_LITE); mobidb-lite (MOBIDB_LITE); mobidb-lite (MOBIDB_LITE); mobidb-lite (MOBIDB_LITE); NON_CYTOPLASMIC_DOMAIN (PHOBIUS); TRANSMEMBRANE (PHOBIUS); CYTOPLASMIC_DOMAIN (PHOBIUS); TMhelix (TMHMM)	no GO terms	no GO terms
261	CP015001.1	Pf-tRNA-Gly	II	Uncharacterised protein	200	20	1.75E-145	70.47						no IPS match	no IPS match	no IPS match
262	CP015001.1	Pf-tRNA-Gly	II	Uncharacterised protein	211	20	4.45E-155	73.36						no IPS match	no IPS match	no IPS match
263	CP015002.1	Pf-tRNA-Gly	II	type II toxin-antitoxin system RelE/ParE family toxin	115	20	1.69E-76	97.87						IPR035093 (G3DSA:3.30.2310.GENE3D); IPR007712 (PFAM)	no GO terms	no GO terms
264	CP015002.1	Pf-tRNA-Gly	II	type II toxin-antitoxin system Phd/YefM family antitoxin	83	20	6.81E-53	96.37						PTHR33713:SF7 (PANTHER); PTHR33713 (PANTHER); IPR036165 (SUPERFAMILY)	no GO terms	no GO terms
265	CP015002.1	Pf-tRNA-Gly	II	hypothetical protein	155	20	2.89E-104	76.22	1	C:GO:0005575	C:cellular_component			TRANSMEMBRANE (PHOBIUS); NON_CYTOPLASMIC_DOMAIN (PHOBIUS); NON_CYTOPLASMIC_DOMAIN (PHOBIUS); TRANSMEMBRANE (PHOBIUS); CYTOPLASMIC_DOMAIN (PHOBIUS); TRANSMEMBRANE (PHOBIUS); CYTOPLASMIC_DOMAIN (PHOBIUS); CYTOPLASMIC_DOMAIN (PHOBIUS); TRANSMEMBRANE (PHOBIUS); TMhelix (TMHMM); TMhelix (TMHMM); TMhelix (TMHMM); TMhelix (TMHMM)	no GO terms	no GO terms
266	CP015002.1	Pf-tRNA-Gly	II	Uncharacterised protein	242	20	1.41E-174	96.08	1	C:GO:0005575	C:cellular_component			mobidb-lite (MOBIDB_LITE); mobidb-lite (MOBIDB_LITE); mobidb-lite (MOBIDB_LITE); mobidb-lite (MOBIDB_LITE); mobidb-lite (MOBIDB_LITE); TRANSMEMBRANE (PHOBIUS); CYTOPLASMIC_DOMAIN (PHOBIUS); NON_CYTOPLASMIC_DOMAIN (PHOBIUS); TMhelix (TMHMM)	no GO terms	no GO terms
267	CP015002.1	Pf-tRNA-Gly	II	Uncharacterised protein	200	20	1.75E-145	70.47						no IPS match	no IPS match	no IPS match
268	CP015002.1	Pf-tRNA-Gly	II	Uncharacterised protein	211	20	4.45E-155	73.36						no IPS match	no IPS match	no IPS match
269	CP015003.1	Pf-tRNA-Gly	II	type II toxin-antitoxin system RelE/ParE family toxin	115	20	1.69E-76	97.87						IPR035093 (G3DSA:3.30.2310.GENE3D); IPR007712 (PFAM)	no GO terms	no GO terms
270	CP015003.1	Pf-tRNA-Gly	II	type II toxin-antitoxin system Phd/YefM family antitoxin	83	20	6.81E-53	96.37						PTHR33713:SF7 (PANTHER); PTHR33713 (PANTHER); IPR036165 (SUPERFAMILY)	no GO terms	no GO terms
271	CP015003.1	Pf-tRNA-Gly	II	hypothetical protein	155	20	2.89E-104	76.22	1	C:GO:0005575	C:cellular_component			TRANSMEMBRANE (PHOBIUS); NON_CYTOPLASMIC_DOMAIN (PHOBIUS); TRANSMEMBRANE (PHOBIUS); TRANSMEMBRANE (PHOBIUS); CYTOPLASMIC_DOMAIN (PHOBIUS); CYTOPLASMIC_DOMAIN (PHOBIUS); CYTOPLASMIC_DOMAIN (PHOBIUS); TRANSMEMBRANE (PHOBIUS); NON_CYTOPLASMIC_DOMAIN (PHOBIUS); TMhelix (TMHMM); TMhelix (TMHMM); TMhelix (TMHMM); TMhelix (TMHMM)	no GO terms	no GO terms
272	CP015003.1	Pf-tRNA-Gly	II	Uncharacterised protein	242	20	1.41E-174	96.08	1	C:GO:0005575	C:cellular_component			mobidb-lite (MOBIDB_LITE); mobidb-lite (MOBIDB_LITE); mobidb-lite (MOBIDB_LITE); mobidb-lite (MOBIDB_LITE); mobidb-lite (MOBIDB_LITE); TRANSMEMBRANE (PHOBIUS); CYTOPLASMIC_DOMAIN (PHOBIUS); NON_CYTOPLASMIC_DOMAIN (PHOBIUS); TMhelix (TMHMM)	no GO terms	no GO terms
273	CP015003.1	Pf-tRNA-Gly	II	Uncharacterised protein	200	20	1.75E-145	70.47						no IPS match	no IPS match	no IPS match
274	CP015003.1	Pf-tRNA-Gly	II	Uncharacterised protein	211	20	4.45E-155	73.36						no IPS match	no IPS match	no IPS match
275	CP015117.1	Pf-tRNA-Gly	II	type II toxin-antitoxin system RelE/ParE family toxin	115	20	5.90E-77	98.35						IPR007712 (PFAM); IPR035093 (G3DSA:3.30.2310.GENE3D)	no GO terms	no GO terms
276	CP015117.1	Pf-tRNA-Gly	II	type II toxin-antitoxin system Phd/YefM family antitoxin	83	20	6.81E-53	96.37						PTHR33713:SF7 (PANTHER); PTHR33713 (PANTHER); IPR036165 (SUPERFAMILY)	no GO terms	no GO terms
277	CP015117.1	Pf-tRNA-Gly	II	Uncharacterised protein	230	20	2.58E-173	96.26	1	C:GO:0005575	C:cellular_component			mobidb-lite (MOBIDB_LITE); mobidb-lite (MOBIDB_LITE); mobidb-lite (MOBIDB_LITE); TRANSMEMBRANE (PHOBIUS); NON_CYTOPLASMIC_DOMAIN (PHOBIUS); CYTOPLASMIC_DOMAIN (PHOBIUS); TMhelix (TMHMM)	no GO terms	no GO terms
278	CP015117.1	Pf-tRNA-Gly	II	Uncharacterised protein	504	20	0	74.14						mobidb-lite (MOBIDB_LITE)	no GO terms	no GO terms
279	CP015117.1	Pf-tRNA-Met	II	MULTISPECIES: hypothetical protein	136	20	1.03E-93	98.01	1	C:GO:0005575	C:cellular_component			CYTOPLASMIC_DOMAIN (PHOBIUS); TRANSMEMBRANE (PHOBIUS); CYTOPLASMIC_DOMAIN (PHOBIUS); NON_CYTOPLASMIC_DOMAIN (PHOBIUS); NON_CYTOPLASMIC_DOMAIN (PHOBIUS); TRANSMEMBRANE (PHOBIUS); TRANSMEMBRANE (PHOBIUS); TMhelix (TMHMM); TMhelix (TMHMM); TMhelix (TMHMM)	no GO terms	no GO terms
280	CP015117.1	Pf-tRNA-Met	II	Cro/CI family transcriptional regulator	264	20	0	86.3	7	P:GO:0001666; P:GO:0019217; P:GO:0045892; P:GO:0045926; P:GO:0045927; F:GO:0003677	P:response to hypoxia; P:regulation of fatty acid metabolic process; P:negative regulation of transcription, DNA-templated; P:negative regulation of growth; P:positive regulation of growth; F:DNA binding			IPR001387 (SMART); IPR007374 (SMART); IPR007374 (PFAM); IPR001387 (PFAM); G3DSA:1.10.260.40 (GENE3D); G3DSA:2.30.130.30 (GENE3D); IPR001387 (PROSITE_PROFILES); IPR001387 (CDD); IPR015947 (SUPERFAMILY); IPR010982 (SUPERFAMILY)	F:GO:0003677	F:DNA binding
281	CP015117.1	Pf-tRNA-Met	II	GNAT family N-acetyltransferase	512	20	0	98.42	1	F:GO:0016746	F:transferase activity, transferring acyl groups	EC:2.3.1.5	Arylamine N-acetyltransferase	G3DSA:3.40.630.30 (GENE3D); IPR000182 (PFAM); IPR000182 (PROSITE_PROFILES); cd18699 (CDD); IPR016181 (SUPERFAMILY)	F:GO:0008080	F:N-acetyltransferase activity
282	CP015117.1	Pf-tRNA-Met	II	AAA family ATPase	175	20	2.76E-124	83.12				EC:2.7.4.3	Adenylate kinase	PF13207 (PFAM); G3DSA:3.40.50.300 (GENE3D); IPR027417 (SUPERFAMILY)	no GO terms	no GO terms
283	CP015377.1	Pf-tRNA-Gly	I	Uncharacterised protein	74	20	4.51E-47	86.4						no IPS match	no IPS match	no IPS match
284	CP015377.1	Pf-tRNA-Gly	I	Uncharacterised protein	232	20	1.50E-174	96.94	1	C:GO:0005575	C:cellular_component			mobidb-lite (MOBIDB_LITE); mobidb-lite (MOBIDB_LITE); TRANSMEMBRANE (PHOBIUS); NON_CYTOPLASMIC_DOMAIN (PHOBIUS); CYTOPLASMIC_DOMAIN (PHOBIUS); TMhelix (TMHMM)	no GO terms	no GO terms
285	CP015377.1	Pf-tRNA-Gly	I	DUF4276 family protein	186	20	7.14E-134	64.81						IPR025455 (PFAM)	no GO terms	no GO terms
286	CP015377.1	Pf-tRNA-Gly	I	AAA family ATPase	407	20	0	67.77	1	F:GO:0043167	F:ion binding			IPR003959 (PFAM); IPR014555 (PIRSF); PTHR40396 (PANTHER); IPR027417 (SUPERFAMILY)	F:GO:0005524	F:ATP binding
287	CP015877.1	Pf-tRNA-Met	I	(deoxy)nucleoside triphosphate pyrophosphohydrolase	129	20	6.12E-88	91.12	4	P:GO:0006281; F:GO:0008413	P:DNA repair; F:8-oxo-7,8-dihydroguanosine triphosphate pyrophosphatase activity	EC:3.6.1.55; EC:3.6.1.65; EC:3.6.1.9	8-oxo-dGTP diphosphatase; (d)CTP diphosphatase; Nucleotide diphosphatase	IPR020476 (PRINTS); G3DSA:3.90.79.10 (GENE3D); IPR000086 (PFAM); PTHR47707 (PANTHER); IPR020084 (PROSITE_PATTERNS); IPR000086 (PROSITE_PROFILES); cd03425 (CDD); IPR015797 (SUPERFAMILY)	F:GO:0016787	F:hydrolase activity
288	CP015877.1	Pf-tRNA-Met	I	hypothetical protein	125	20	3.18E-85	99.04						no IPS match	no IPS match	no IPS match
289	CP015877.1	Pf-tRNA-Met	I	MULTISPECIES: hypothetical protein	136	20	1.03E-93	98.01	1	C:GO:0005575	C:cellular_component			CYTOPLASMIC_DOMAIN (PHOBIUS); TRANSMEMBRANE (PHOBIUS); CYTOPLASMIC_DOMAIN (PHOBIUS); NON_CYTOPLASMIC_DOMAIN (PHOBIUS); NON_CYTOPLASMIC_DOMAIN (PHOBIUS); TRANSMEMBRANE (PHOBIUS); TRANSMEMBRANE (PHOBIUS); TMhelix (TMHMM); TMhelix (TMHMM); TMhelix (TMHMM)	no GO terms	no GO terms
290	CP015877.1	Pf-tRNA-Met	I	hypothetical protein	111	20	3.04E-75	98.47						SIGNAL_PEPTIDE_H_REGION (PHOBIUS); SIGNAL_PEPTIDE_N_REGION (PHOBIUS); SIGNAL_PEPTIDE_C_REGION (PHOBIUS); NON_CYTOPLASMIC_DOMAIN (PHOBIUS); SIGNAL_PEPTIDE (PHOBIUS); SignalP-TM (SIGNALP_GRAM_POSITIVE); SignalP-noTM (SIGNALP_EUK); SignalP-noTM (SIGNALP_GRAM_NEGATIVE)	no GO terms	no GO terms
291	CP015877.1	Pf-tRNA-Met	I	restriction endonuclease	245	20	0	95.39	1	F:GO:0004518	F:nuclease activity			Coil (COILS); IPR003615 (SMART); G3DSA:3.30.40.60 (GENE3D); IPR002711 (PFAM); mobidb-lite (MOBIDB_LITE); mobidb-lite (MOBIDB_LITE); PTHR33877 (PANTHER); PTHR33877:SF1 (PANTHER); IPR003615 (CDD)	F:GO:0003676; F:GO:0004519	F:nucleic acid binding; F:endonuclease activity
292	CP015877.1	Pf-tRNA-Met	I	DNA-processing protein DprA	323	20	0	75.71	1	P:GO:0008150	P:biological_process			G3DSA:3.40.50.450 (GENE3D); IPR003488 (PFAM); IPR003488 (PANTHER); SSF102405 (SUPERFAMILY)	P:GO:0009294	P:DNA mediated transformation
293	CP015877.1	Pf-tRNA-Met	I	HAD hydrolase-like protein	411	20	0	73.22	4	P:GO:0034641; P:GO:0044281	P:cellular nitrogen compound metabolic process; P:small molecule metabolic process	EC:3.1.3.18; EC:3.6.4.12	Phosphoglycolate phosphatase; DNA helicase	G3DSA:3.40.50.2020 (GENE3D); IPR023214 (G3DSA:3.40.50.GENE3D); IPR041492 (PFAM); IPR000836 (CDD); cd01427 (CDD); IPR029057 (SUPERFAMILY); IPR036412 (SUPERFAMILY)	P:GO:0009116	P:nucleoside metabolic process
294	CP016214.1	Pf-tRNA-Met	I	hypothetical protein	136	20	1.37E-94	99.74	1	C:GO:0005575	C:cellular_component			CYTOPLASMIC_DOMAIN (PHOBIUS); CYTOPLASMIC_DOMAIN (PHOBIUS); TRANSMEMBRANE (PHOBIUS); NON_CYTOPLASMIC_DOMAIN (PHOBIUS); NON_CYTOPLASMIC_DOMAIN (PHOBIUS); TRANSMEMBRANE (PHOBIUS); TRANSMEMBRANE (PHOBIUS); TMhelix (TMHMM); TMhelix (TMHMM)	no GO terms	no GO terms
295	CP016214.1	Pf-tRNA-Met	II	hypothetical protein	98	20	1.80E-56	96.76						SIGNAL_PEPTIDE_H_REGION (PHOBIUS); NON_CYTOPLASMIC_DOMAIN (PHOBIUS); SIGNAL_PEPTIDE_N_REGION (PHOBIUS); SIGNAL_PEPTIDE_C_REGION (PHOBIUS); SIGNAL_PEPTIDE (PHOBIUS); SignalP-noTM (SIGNALP_EUK); SignalP-TM (SIGNALP_GRAM_POSITIVE); SignalP-noTM (SIGNALP_GRAM_NEGATIVE)	no GO terms	no GO terms
296	CP016214.1	Pf-tRNA-Met	II	IS30 family transposase IS1394	338	20	0	91.47	4	P:GO:0006313; P:GO:0015074; F:GO:0003677; F:GO:0004803	P:transposition, DNA-mediated; P:DNA integration; F:DNA binding; F:transposase activity			IPR025246 (PFAM); IPR036397 (G3DSA:3.30.420.GENE3D); IPR001584 (PFAM); PTHR10948 (PANTHER); IPR001598 (PROSITE_PATTERNS); IPR001584 (PROSITE_PROFILES); IPR012337 (SUPERFAMILY)	P:GO:0006313; P:GO:0015074; F:GO:0003676; F:GO:0003677; F:GO:0004803	P:transposition, DNA-mediated; P:DNA integration; F:nucleic acid binding; F:DNA binding; F:transposase activity
297	CP016214.1	Pf-tRNA-Met	II	S1 RNA-binding domain-containing protein	852	20	0	79.88	4	P:GO:0006304; F:GO:0003677; F:GO:0004519; C:GO:0005840	P:DNA modification; F:DNA binding; F:endonuclease activity; C:ribosome			IPR022967 (SMART); IPR007409 (PFAM); G3DSA:2.40.50.140 (GENE3D); G3DSA:3.90.1570.30 (GENE3D); IPR003029 (PFAM); PTHR10724:SF7 (PANTHER); PTHR10724 (PANTHER); IPR003029 (PROSITE_PROFILES); IPR012340 (SUPERFAMILY); IPR027417 (SUPERFAMILY)	P:GO:0006304; F:GO:0003676; F:GO:0003677; F:GO:0004519	P:DNA modification; F:nucleic acid binding; F:DNA binding; F:endonuclease activity
298	CP016214.1	Pf-tRNA-Sec	I	Uncharacterised protein	59	20	1.67E-22	88.93	1	C:GO:0005575	C:cellular_component			no IPS match	no IPS match	no IPS match
299	CP016214.1	Pf-tRNA-Sec	I	Uncharacterised protein	242	20	4.52E-174	98.02						mobidb-lite (MOBIDB_LITE); mobidb-lite (MOBIDB_LITE); mobidb-lite (MOBIDB_LITE); mobidb-lite (MOBIDB_LITE); mobidb-lite (MOBIDB_LITE); TRANSMEMBRANE (PHOBIUS); CYTOPLASMIC_DOMAIN (PHOBIUS); NON_CYTOPLASMIC_DOMAIN (PHOBIUS); TMhelix (TMHMM)	no GO terms	no GO terms
300	CP016214.1	Pf-tRNA-Sec	I	DUF4238 domain-containing protein	344	20	0	82.31						IPR025332 (PFAM)	no GO terms	no GO terms
301	CP016214.1	Pf-tRNA-Sec	I	---NA---	37									no IPS match	no IPS match	no IPS match
302	CP016955.1	Pf-tRNA-Met	I	hypothetical protein	136	20	1.37E-94	99.74	1	C:GO:0005575	C:cellular_component			CYTOPLASMIC_DOMAIN (PHOBIUS); CYTOPLASMIC_DOMAIN (PHOBIUS); TRANSMEMBRANE (PHOBIUS); NON_CYTOPLASMIC_DOMAIN (PHOBIUS); NON_CYTOPLASMIC_DOMAIN (PHOBIUS); TRANSMEMBRANE (PHOBIUS); TRANSMEMBRANE (PHOBIUS); TMhelix (TMHMM); TMhelix (TMHMM)	no GO terms	no GO terms

303	CP016955.1	Pf-tRNA-Met	I	hypothetical protein	111	20	1.24E-74	98.56						mobidb-lite (MOBIDB_LITE); mobidb-lite (MOBIDB_LITE); NON_CYTOPLASMIC_DOMAIN (PHOBIUS); SIGNAL_PEPTIDE (PHOBIUS); SIGNAL_PEPTIDE_C_REGION (PHOBIUS); SIGNAL_PEPTIDE_N_REGION (PHOBIUS); SIGNAL_PEPTIDE_H_REGION (PHOBIUS); SignalP-noTM (SIGNALP_EUK); SignalP-noTM (SIGNALP_GRAM_NEGATIVE); SignalP-TM (SIGNALP_GRAM_POSITIVE)	no GO terms	no GO terms
304	CP016955.1	Pf-tRNA-Met	I	Uncharacterised protein	363	20	0	72.55	1	C:GO:0005575	C:cellular_component			CYTOPLASMIC_DOMAIN (PHOBIUS); TRANSMEMBRANE (PHOBIUS); NON_CYTOPLASMIC_DOMAIN (PHOBIUS); CYTOPLASMIC_DOMAIN (PHOBIUS); TRANSMEMBRANE (PHOBIUS); TMhelix (TMHMM); TMhelix (TMHMM)	no GO terms	no GO terms
305	CP016955.1	Pf-tRNA-Met	I	hypothetical protein	130	20	2.19E-89	98.14						no IPS match	no IPS match	no IPS match
306	CP017149.1	Pf-tRNA-Gly	I	type II toxin-antitoxin system RelE/ParE family toxin	115	20	2.09E-76	98.57						IPR007712 (PFAM); IPR035093 (G3DSA:3.30.2310.GENE3D)	no GO terms	no GO terms
307	CP017149.1	Pf-tRNA-Gly	I	type II toxin-antitoxin system Phd/YefM family antitoxin	83	20	6.81E-53	96.37						PTHR33713:SF7 (PANTHER); PTHR33713 (PANTHER); IPR036165 (SUPERFAMILY)	no GO terms	no GO terms
308	CP017149.1	Pf-tRNA-Gly	I	AAA family ATPase	441	20	0	78.07	1	F:GO:0043167	F:ion binding			IPR003593 (SMART); G3DSA:3.40.50.300 (GENE3D); IPR038729 (PFAM); IPR003959 (PFAM); PTHR43581:SF2 (PANTHER); PTHR43581 (PANTHER); IPR027417 (SUPERFAMILY)	F:GO:0005524	F:ATP binding
309	CP017149.1	Pf-tRNA-Gly	I	RNA-directed DNA polymerase	318	20	0	79.16	4	P:GO:0006259; P:GO:0009058; F:GO:0003723; F:GO:0016779	P:DNA metabolic process; P:biosynthetic process; F:RNA binding; F:nucleotidyltransferase activity	EC:2.7.7.49	RNA-directed DNA polymerase	IPR000123 (PRINTS); IPR000477 (PFAM); PTHR34047:SF6 (PANTHER); PTHR34047 (PANTHER); IPR000477 (PROSITE_PROFILES); cd03487 (CDD); SSF56672 (SUPERFAMILY)	P:GO:0006278; F:GO:0003723; F:GO:0003964	P:RNA-dependent DNA biosynthetic process; F:RNA binding; F:RNA-directed DNA polymerase activity
310	CP017149.1	Pf-tRNA-Gly	I	DNA mismatch repair protein MutS	162	20	1.01E-11	74.28	9	P:GO:0006259; P:GO:0006810; P:GO:0006950; P:GO:0051276; F:GO:0003677; F:GO:0004386; F:GO:0004518; F:GO:0043167; C:GO:0005739	P:DNA metabolic process; P:transport; P:response to stress; P:chromosome organization; F:DNA binding; F:helicase activity; F:nuclease activity; F:ion binding; C:mitochondrion	EC:3.6.1.3; EC:3.6.4.12; EC:3.6.1.15	Adenosinetriphosphatase; DNA helicase; Nucleoside-triphosphate phosphatase	mobidb-lite (MOBIDB_LITE); mobidb-lite (MOBIDB_LITE); SIGNAL_PEPTIDE_N_REGION (PHOBIUS); SIGNAL_PEPTIDE (PHOBIUS); NON_CYTOPLASMIC_DOMAIN (PHOBIUS); SIGNAL_PEPTIDE_H_REGION (PHOBIUS); SIGNAL_PEPTIDE_C_REGION (PHOBIUS); SignalP-noTM (SIGNALP_EUK)	no GO terms	no GO terms
311	CP017149.1	Pf-tRNA-Met	I	serine/threonine-protein phosphatase	365	20	0	63.24	2	P:GO:0006470; F:GO:0004722	P:protein dephosphorylation; F:protein serine/threonine phosphatase activity	EC:3.1.3.16	Protein-serine/threonine phosphatase	IPR001932 (SMART); IPR036457 (G3DSA:3.60.40.GENE3D); PF13672 (PFAM); mobidb-lite (MOBIDB_LITE); mobidb-lite (MOBIDB_LITE); mobidb-lite (MOBIDB_LITE); IPR015655 (PANTHER); PTHR13832:SF278 (PANTHER); IPR001932 (PROSITE_PROFILES); IPR001932 (CDD); IPR036457 (SUPERFAMILY)	P:GO:0006470; F:GO:0003824; F:GO:0004722	P:protein dephosphorylation; F:catalytic activity; F:protein serine/threonine phosphatase activity
312	CP017149.1	Pf-tRNA-Met	I	serine/threonine protein kinase	349	20	0	69.97	3	P:GO:0006468; F:GO:0004674; F:GO:0005524	P:protein phosphorylation; F:protein serine/threonine kinase activity; F:ATP binding	EC:2.7.11	Transferring phosphorus-containing groups	IPR000719 (SMART); G3DSA:1.10.510.10 (GENE3D); IPR000719 (PFAM); PTHR24346 (PANTHER); IPR008271 (PROSITE_PATTERNS); IPR000719 (PROSITE_PROFILES); cd14014 (CDD); IPR011009 (SUPERFAMILY)	P:GO:0006468; F:GO:0004672; F:GO:0005524	P:protein phosphorylation; F:protein kinase activity; F:ATP binding
313	CP017149.1	Pf-tRNA-Met	I	serine/threonine protein kinase	357	20	0	78.68	3	P:GO:0006464; F:GO:0016301; F:GO:0043167	P:cellular protein modification process; F:kinase activity; F:ion binding			IPR000719 (SMART); IPR000719 (PFAM); G3DSA:1.10.510.10 (GENE3D); PIRSF000654 (PIRSF); G3DSA:3.30.200.20 (GENE3D); PTHR43289:SF11 (PANTHER); PTHR43289 (PANTHER); IPR008271 (PROSITE_PATTERNS); IPR000719 (PROSITE_PROFILES); cd14014 (CDD); IPR012340 (SUPERFAMILY); IPR011009 (SUPERFAMILY)	P:GO:0006468; F:GO:0004672; F:GO:0005524	P:protein phosphorylation; F:protein kinase activity; F:ATP binding
314	CP017306.1	Pf-tRNA-Gly	II	YfbU family protein	167	20	1.65E-120	62.69	6	P:GO:0006950; P:GO:0006974; C:GO:0005623; C:GO:0005829	P:response to stress; P:cellular response to DNA damage stimulus; C:cell; C:cytosol			IPR023146 (G3DSA:1.10.3190.GENE3D); IPR005587 (PFAM); SSF116960 (SUPERFAMILY)	no GO terms	no GO terms
315	CP017306.1	Pf-tRNA-Gly	II	hypothetical protein	232	20	8.06E-174	95.6	1	C:GO:0005575	C:cellular_component			mobidb-lite (MOBIDB_LITE); mobidb-lite (MOBIDB_LITE); TRANSMEMBRANE (PHOBIUS); NON_CYTOPLASMIC_DOMAIN (PHOBIUS); CYTOPLASMIC_DOMAIN (PHOBIUS); TMhelix (TMHMM)	no GO terms	no GO terms
316	CP017306.1	Pf-tRNA-Gly	II	FRG domain-containing protein	394	20	0	72						IPR014966 (SMART); G3DSA:2.30.29.80 (GENE3D); IPR010879 (PFAM); IPR014966 (PFAM); PTHR40606 (PANTHER); PTHR40606:SF1 (PANTHER); IPR036913 (SUPERFAMILY); IPR036913 (SUPERFAMILY)	no GO terms	no GO terms
317	CP017969.1	Pf-tRNA-Gly	I	type II toxin-antitoxin system RelE/ParE family toxin	115	20	2.09E-76	98.57						IPR007712 (PFAM); IPR035093 (G3DSA:3.30.2310.GENE3D)	no GO terms	no GO terms
318	CP017969.1	Pf-tRNA-Gly	I	type II toxin-antitoxin system Phd/YefM family antitoxin	83	20	6.81E-53	96.37						PTHR33713:SF7 (PANTHER); PTHR33713 (PANTHER); IPR036165 (SUPERFAMILY)	no GO terms	no GO terms
319	CP017969.1	Pf-tRNA-Gly	I	Uncharacterised protein	97	20	1.56E-65	92.06						no IPS match	no IPS match	no IPS match
320	CP017969.1	Pf-tRNA-Gly	I	Uncharacterised protein	232	20	1.20E-174	83.08	1	C:GO:0005575	C:cellular_component			mobidb-lite (MOBIDB_LITE); NON_CYTOPLASMIC_DOMAIN (PHOBIUS); CYTOPLASMIC_DOMAIN (PHOBIUS); TRANSMEMBRANE (PHOBIUS); TMhelix (TMHMM)	no GO terms	no GO terms
321	CP017969.1	Pf-tRNA-Gly	I	Uncharacterised protein	275	20	0	73.59						no IPS match	no IPS match	no IPS match
322	CP017969.1	Pf-tRNA-Gly	I	Uncharacterised protein	244	20	3.24E-180	67.32	1	C:GO:0005575	C:cellular_component			Coil (COILS); SIGNAL_PEPTIDE_C_REGION (PHOBIUS); SIGNAL_PEPTIDE_N_REGION (PHOBIUS); TRANSMEMBRANE (PHOBIUS); CYTOPLASMIC_DOMAIN (PHOBIUS); NON_CYTOPLASMIC_DOMAIN (PHOBIUS); SIGNAL_PEPTIDE (PHOBIUS); SIGNAL_PEPTIDE_H_REGION (PHOBIUS); SignalP-TM (SIGNALP_EUK); TMhelix (TMHMM); TMhelix (TMHMM)	no GO terms	no GO terms
323	CP017969.1	Pf-tRNA-Gly	I	Uncharacterised protein	227	20	5.69E-163	62.41	1	C:GO:0005575	C:cellular_component			Coil (COILS); NON_CYTOPLASMIC_DOMAIN (PHOBIUS); CYTOPLASMIC_DOMAIN (PHOBIUS); TRANSMEMBRANE (PHOBIUS); TRANSMEMBRANE (PHOBIUS); TMhelix (TMHMM); TMhelix (TMHMM)	no GO terms	no GO terms
324	CP017969.1	Pf-tRNA-Gly	I	Uncharacterised protein	98	20	1.43E-64	56.51						no IPS match	no IPS match	no IPS match
325	CP017969.1	Pf-tRNA-Met	I	Uncharacterised protein	145	20	6.89E-100	95.34	1	C:GO:0005575	C:cellular_component			TRANSMEMBRANE (PHOBIUS); CYTOPLASMIC_DOMAIN (PHOBIUS); TRANSMEMBRANE (PHOBIUS); CYTOPLASMIC_DOMAIN (PHOBIUS); NON_CYTOPLASMIC_DOMAIN (PHOBIUS); TMhelix (TMHMM); TMhelix (TMHMM)	no GO terms	no GO terms
326	CP017969.1	Pf-tRNA-Met	I	Uncharacterised protein	111	20	3.44E-74	91.97						SIGNAL_PEPTIDE_C_REGION (PHOBIUS); SIGNAL_PEPTIDE_H_REGION (PHOBIUS); SIGNAL_PEPTIDE_N_REGION (PHOBIUS); NON_CYTOPLASMIC_DOMAIN (PHOBIUS); SIGNAL_PEPTIDE (PHOBIUS); SignalP-TM (SIGNALP_GRAM_POSITIVE); SignalP-noTM (SIGNALP_GRAM_NEGATIVE); SignalP-noTM (SIGNALP_EUK)	no GO terms	no GO terms
327	CP017969.1	Pf-tRNA-Met	I	SLATT domain-containing protein	196	20	2.86E-141	81.86	1	C:GO:0005575	C:cellular_component			IPR041119 (PFAM); NON_CYTOPLASMIC_DOMAIN (PHOBIUS); CYTOPLASMIC_DOMAIN (PHOBIUS); TRANSMEMBRANE (PHOBIUS); TRANSMEMBRANE (PHOBIUS); NON_CYTOPLASMIC_DOMAIN (PHOBIUS); TRANSMEMBRANE (PHOBIUS); CYTOPLASMIC_DOMAIN (PHOBIUS); TMhelix (TMHMM); TMhelix (TMHMM); TMhelix (TMHMM)	no GO terms	no GO terms
328	CP017969.1	Pf-tRNA-Met	I	nucleotidyltransferase	421	20	0	89.25	1	F:GO:0003674	F:molecular_function			PF18144 (PFAM); G3DSA:3.30.460.10 (GENE3D); IPR040511 (PFAM); cd05400 (CDD); SSF81301 (SUPERFAMILY)	no GO terms	no GO terms
329	CP017969.1	Pf-tRNA-Met	I	Uncharacterised protein	330	20	0	84.71						no IPS match	no IPS match	no IPS match
330	CP017969.1	Pf-DR	II	hypothetical protein	206	20	3.04E-150	73.41						no IPS match	no IPS match	no IPS match
331	CP017969.1	Pf-DR	II	DUF262 domain-containing protein	349	20	0	84.49						Coil (COILS); IPR004919 (PFAM); PTHR39639 (PANTHER); cd17157 (CDD)	no GO terms	no GO terms
332	CP020560.1	Pf-tRNA-Gly	I	type II toxin-antitoxin system RelE/ParE family toxin	115	20	2.09E-76	98.57						IPR007712 (PFAM); IPR035093 (G3DSA:3.30.2310.GENE3D)	no GO terms	no GO terms
333	CP020560.1	Pf-tRNA-Gly	I	type II toxin-antitoxin system Phd/YefM family antitoxin	83	20	6.81E-53	96.37						PTHR33713:SF7 (PANTHER); PTHR33713 (PANTHER); IPR036165 (SUPERFAMILY)	no GO terms	no GO terms
334	CP020560.1	Pf-tRNA-Gly	I	non-ribosomal peptide synthetase modules	702	20	0	66.42						no IPS match	no IPS match	no IPS match
335	CP020560.1	Pf-tRNA-Met	I	plasmid maintenance system killer protein	92	20	3.90E-62	99.08						IPR035093 (G3DSA:3.30.2310.GENE3D); IPR007711 (PFAM); IPR007711 (PANTHER); IPR035093 (SUPERFAMILY)	no GO terms	no GO terms
336	CP020560.1	Pf-tRNA-Met	I	addiction module antidote protein, HigA family	101	20	2.97E-68	97.48	1	F:GO:0003677	F:DNA binding			IPR001387 (SMART); IPR001387 (PFAM); G3DSA:1.10.260.40 (GENE3D); IPR013430 (TIGRFAM); IPR013430 (PANTHER); IPR001387 (PROSITE_PROFILES); IPR001387 (CDD); IPR010982 (SUPERFAMILY)	F:GO:0003677	F:DNA binding
337	CP020560.1	Pf-tRNA-Met	I	hypothetical protein	230	20	2.41E-167	84.6				EC:2.1.1.72	Site-specific DNA-methyltransferase (adenine-specific)	no IPS match	no IPS match	no IPS match
338	CP020560.1	Pf-tRNA-Met	I	DUF4433 domain-containing protein	173	20	1.01E-125	74.96						IPR029494 (PFAM)	no GO terms	no GO terms
339	CP020560.1	Pf-tRNA-Met	I	DEAD/DEAH box helicase	703	20	0	82.67	4	P:GO:0010212; P:GO:0051607; F:GO:0016853	P:response to ionizing radiation; P:defense response to virus; F:isomerase activity			IPR014001 (SMART); G3DSA:3.40.50.300 (GENE3D); G3DSA:3.40.50.300 (GENE3D); PTHR12131:SF17 (PANTHER); PTHR12131 (PANTHER); IPR014001 (PROSITE_PROFILES); IPR001650 (PROSITE_PROFILES); cd18795 (CDD); cd17921 (CDD); IPR027417 (SUPERFAMILY)	no GO terms	no GO terms
340	CP020560.1	Pf-tRNA-Met	I	DUF1837 domain-containing protein	306	20	0	83.02						IPR014976 (PFAM)	no GO terms	no GO terms
341	CP020560.1	Pf-tRNA-Met	I	hypothetical protein SAMN05216209_1853	235	20	1.15E-139	67.81	1	C:GO:0005575	C:cellular_component			mobidb-lite (MOBIDB_LITE); NON_CYTOPLASMIC_DOMAIN (PHOBIUS); CYTOPLASMIC_DOMAIN (PHOBIUS); TRANSMEMBRANE (PHOBIUS); TMhelix (TMHMM)	no GO terms	no GO terms
342	CP020560.1	Pf-tRNA-Sec	II	DUF262 domain-containing protein	512	20	0	78.9	1	F:GO:0004518	F:nuclease activity			IPR003615 (SMART); IPR004919 (PFAM); IPR002711 (PFAM); G3DSA:3.30.40.60 (GENE3D); IPR003615 (CDD); IPR036086 (SUPERFAMILY)	F:GO:0003676; F:GO:0004519	F:nucleic acid binding; F:endonuclease activity
343	CP020659.1	Pf-tRNA-Met	I	hypothetical protein	136	20	1.56E-93	98.01	1	C:GO:0005575	C:cellular_component			CYTOPLASMIC_DOMAIN (PHOBIUS); TRANSMEMBRANE (PHOBIUS); CYTOPLASMIC_DOMAIN (PHOBIUS); NON_CYTOPLASMIC_DOMAIN (PHOBIUS); NON_CYTOPLASMIC_DOMAIN (PHOBIUS); TRANSMEMBRANE (PHOBIUS); TRANSMEMBRANE (PHOBIUS); TMhelix (TMHMM); TMhelix (TMHMM); TMhelix (TMHMM)	no GO terms	no GO terms
344	CP020659.1	Pf-tRNA-Met	I	hypothetical protein	111	20	9.31E-75	98.29						SIGNAL_PEPTIDE_H_REGION (PHOBIUS); SIGNAL_PEPTIDE_N_REGION (PHOBIUS); SIGNAL_PEPTIDE_C_REGION (PHOBIUS); NON_CYTOPLASMIC_DOMAIN (PHOBIUS); SIGNAL_PEPTIDE (PHOBIUS); SignalP-noTM (SIGNALP_EUK); SignalP-TM (SIGNALP_GRAM_POSITIVE); SignalP-noTM (SIGNALP_GRAM_NEGATIVE)	no GO terms	no GO terms
345	CP020659.1	Pf-tRNA-Met	I	DUF262 domain-containing protein	381	20	0	72.5						IPR004919 (PFAM); mobidb-lite (MOBIDB_LITE); PTHR39639 (PANTHER)	no GO terms	no GO terms
346	CP020659.1	Pf-tRNA-Met	I	Uncharacterised protein	255	20	0	58.19						IPR041519 (PFAM)	no GO terms	no GO terms
347	CP020703.1	Pf-tRNA-Gly	I	Uncharacterised protein	109	16	2.61E-73	69.06						no IPS match	no IPS match	no IPS match
348	CP020703.1	Pf-tRNA-Gly	I	type II toxin-antitoxin system RelE/ParE family toxin	115	20	2.09E-76	98.57						IPR007712 (PFAM); IPR035093 (G3DSA:3.30.2310.GENE3D)	no GO terms	no GO terms
349	CP020703.1	Pf-tRNA-Gly	I	type II toxin-antitoxin system Phd/YefM family antitoxin	83	20	6.81E-53	96.37						PTHR33713:SF7 (PANTHER); PTHR33713 (PANTHER); IPR036165 (SUPERFAMILY)	no GO terms	no GO terms
350	CP020703.1	Pf-tRNA-Gly	I	Uncharacterised protein	97	20	1.56E-65	92.06						no IPS match	no IPS match	no IPS match
351	CP020703.1	Pf-tRNA-Gly	I	Uncharacterised protein	232	20	1.20E-174	83.08	1	C:GO:0005575	C:cellular_component			mobidb-lite (MOBIDB_LITE); CYTOPLASMIC_DOMAIN (PHOBIUS); TRANSMEMBRANE (PHOBIUS); NON_CYTOPLASMIC_DOMAIN (PHOBIUS); TMhelix (TMHMM)	no GO terms	no GO terms
352	CP020703.1	Pf-tRNA-Gly	I	Uncharacterised protein	275	20	0	73.59						no IPS match	no IPS match	no IPS match
353	CP020703.1	Pf-tRNA-Gly	I	Uncharacterised protein	244	20	3.24E-180	67.32	1	C:GO:0005575	C:cellular_component			Coil (COILS); SIGNAL_PEPTIDE (PHOBIUS); NON_CYTOPLASMIC_DOMAIN (PHOBIUS); SIGNAL_PEPTIDE_C_REGION (PHOBIUS); SIGNAL_PEPTIDE_N_REGION (PHOBIUS); TRANSMEMBRANE (PHOBIUS); SIGNAL_PEPTIDE_H_REGION (PHOBIUS); CYTOPLASMIC_DOMAIN (PHOBIUS); SignalP-TM (SIGNALP_EUK); TMhelix (TMHMM); TMhelix (TMHMM)	no GO terms	no GO terms
354	CP020703.1	Pf-tRNA-Gly	I	Uncharacterised protein	98	20	1.43E-64	56.51						no IPS match	no IPS match	no IPS match
355	CP020703.1	Pf-tRNA-Gly	I	Uncharacterised protein	227	20	5.69E-163	62.41	1	C:GO:0005575	C:cellular_component			Coil (COILS); TRANSMEMBRANE (PHOBIUS); NON_CYTOPLASMIC_DOMAIN (PHOBIUS); TRANSMEMBRANE (PHOBIUS); NON_CYTOPLASMIC_DOMAIN (PHOBIUS); CYTOPLASMIC_DOMAIN (PHOBIUS); TMhelix (TMHMM); TMhelix (TMHMM)	no GO terms	no GO terms
356	CP020703.1	Pf-tRNA-Met	I	Uncharacterised protein	145	20	6.89E-100	95.34	1	C:GO:0005575	C:cellular_component			TRANSMEMBRANE (PHOBIUS); CYTOPLASMIC_DOMAIN (PHOBIUS); TRANSMEMBRANE (PHOBIUS); CYTOPLASMIC_DOMAIN (PHOBIUS); NON_CYTOPLASMIC_DOMAIN (PHOBIUS); TMhelix (TMHMM); TMhelix (TMHMM)	no GO terms	no GO terms
357	CP020703.1	Pf-tRNA-Met	I	Uncharacterised protein	111	20	3.44E-74	91.97						SIGNAL_PEPTIDE_C_REGION (PHOBIUS); SIGNAL_PEPTIDE_H_REGION (PHOBIUS); SIGNAL_PEPTIDE_N_REGION (PHOBIUS); NON_CYTOPLASMIC_DOMAIN (PHOBIUS); SIGNAL_PEPTIDE (PHOBIUS); SignalP-noTM (SIGNALP_GRAM_NEGATIVE); SignalP-TM (SIGNALP_GRAM_POSITIVE); SignalP-noTM (SIGNALP_EUK)	no GO terms	no GO terms
358	CP020703.1	Pf-tRNA-Met	I	SLATT domain-containing protein	196	20	2.86E-141	81.86	1	C:GO:0005575	C:cellular_component			IPR041119 (PFAM); NON_CYTOPLASMIC_DOMAIN (PHOBIUS); CYTOPLASMIC_DOMAIN (PHOBIUS); TRANSMEMBRANE (PHOBIUS); TRANSMEMBRANE (PHOBIUS); NON_CYTOPLASMIC_DOMAIN (PHOBIUS); TRANSMEMBRANE (PHOBIUS); CYTOPLASMIC_DOMAIN (PHOBIUS); TMhelix (TMHMM); TMhelix (TMHMM); TMhelix (TMHMM)	no GO terms	no GO terms
359	CP020703.1	Pf-tRNA-Met	I	nucleotidyltransferase	421	20	0	89.25	1	F:GO:0003674	F:molecular_function			PF18144 (PFAM); G3DSA:3.30.460.10 (GENE3D); IPR040511 (PFAM); cd05400 (CDD); SSF81301 (SUPERFAMILY)	no GO terms	no GO terms
360	CP020703.1	Pf-tRNA-Met	I	Uncharacterised protein	330	20	0	84.71						no IPS match	no IPS match	no IPS match
361	CP020704.1	Pf-tRNA-Gly	I	type II toxin-antitoxin system RelE/ParE family toxin	115	20	2.09E-76	98.57						IPR007712 (PFAM); IPR035093 (G3DSA:3.30.2310.GENE3D)	no GO terms	no GO terms
362	CP020704.1	Pf-tRNA-Gly	I	type II toxin-antitoxin system Phd/YefM family antitoxin	83	20	6.81E-53	96.37						PTHR33713:SF7 (PANTHER); PTHR33713 (PANTHER); IPR036165 (SUPERFAMILY)	no GO terms	no GO terms
363	CP020704.1	Pf-tRNA-Gly	I	Uncharacterised protein	109	16	2.61E-73	69.06						no IPS match	no IPS match	no IPS match
364	CP020704.1	Pf-tRNA-Gly	I	Uncharacterised protein	97	20	1.56E-65	92.06						no IPS match	no IPS match	no IPS match
365	CP020704.1	Pf-tRNA-Gly	I	Uncharacterised protein	232	20	1.20E-174	83.08	1	C:GO:0005575	C:cellular_component			mobidb-lite (MOBIDB_LITE); NON_CYTOPLASMIC_DOMAIN (PHOBIUS); CYTOPLASMIC_DOMAIN (PHOBIUS); TRANSMEMBRANE (PHOBIUS); TMhelix (TMHMM)	no GO terms	no GO terms
366	CP020704.1	Pf-tRNA-Gly	I	Uncharacterised protein	275	20	0	73.59						no IPS match	no IPS match	no IPS match
367	CP020704.1	Pf-tRNA-Gly	I	Uncharacterised protein	244	20	3.24E-180	67.32	1	C:GO:0005575	C:cellular_component			Coil (COILS); SIGNAL_PEPTIDE_C_REGION (PHOBIUS); SIGNAL_PEPTIDE_N_REGION (PHOBIUS); TRANSMEMBRANE (PHOBIUS); CYTOPLASMIC_DOMAIN (PHOBIUS); NON_CYTOPLASMIC_DOMAIN (PHOBIUS); SIGNAL_PEPTIDE (PHOBIUS); SIGNAL_PEPTIDE_H_REGION (PHOBIUS); SignalP-TM (SIGNALP_EUK); TMhelix (TMHMM); TMhelix (TMHMM)	no GO terms	no GO terms
368	CP020704.1	Pf-tRNA-Gly	I	Uncharacterised protein	98	20	1.43E-64	56.51						no IPS match	no IPS match	no IPS match
369	CP020704.1	Pf-tRNA-Gly	I	Uncharacterised protein	227	20	5.69E-163	62.41	1	C:GO:0005575	C:cellular_component			Coil (COILS); TRANSMEMBRANE (PHOBIUS); NON_CYTOPLASMIC_DOMAIN (PHOBIUS); TRANSMEMBRANE (PHOBIUS); NON_CYTOPLASMIC_DOMAIN (PHOBIUS); CYTOPLASMIC_DOMAIN (PHOBIUS); TMhelix (TMHMM); TMhelix (TMHMM)	no GO terms	no GO terms
370	CP020704.1	Pf-tRNA-Met	I	Uncharacterised protein	145	20	6.89E-100	95.34	1	C:GO:0005575	C:cellular_component			TRANSMEMBRANE (PHOBIUS); CYTOPLASMIC_DOMAIN (PHOBIUS); TRANSMEMBRANE (PHOBIUS); CYTOPLASMIC_DOMAIN (PHOBIUS); NON_CYTOPLASMIC_DOMAIN (PHOBIUS); TMhelix (TMHMM); TMhelix (TMHMM)	no GO terms	no GO terms
371	CP020704.1	Pf-tRNA-Met	I	Uncharacterised protein	111	20	3.44E-74	91.97						SIGNAL_PEPTIDE_C_REGION (PHOBIUS); SIGNAL_PEPTIDE_H_REGION (PHOBIUS); SIGNAL_PEPTIDE_N_REGION (PHOBIUS); NON_CYTOPLASMIC_DOMAIN (PHOBIUS); SIGNAL_PEPTIDE (PHOBIUS); SignalP-noTM (SIGNALP_EUK); SignalP-TM (SIGNALP_GRAM_POSITIVE); SignalP-noTM (SIGNALP_GRAM_NEGATIVE)	no GO terms	no GO terms
372	CP020704.1	Pf-tRNA-Met	I	SLATT domain-containing protein	196	20	2.86E-141	81.86	1	C:GO:0005575	C:cellular_component			IPR041119 (PFAM); NON_CYTOPLASMIC_DOMAIN (PHOBIUS); CYTOPLASMIC_DOMAIN (PHOBIUS); TRANSMEMBRANE (PHOBIUS); TRANSMEMBRANE (PHOBIUS); NON_CYTOPLASMIC_DOMAIN (PHOBIUS); TRANSMEMBRANE (PHOBIUS); CYTOPLASMIC_DOMAIN (PHOBIUS); TMhelix (TMHMM); TMhelix (TMHMM); TMhelix (TMHMM)	no GO terms	no GO terms
373	CP020704.1	Pf-tRNA-Met	I	nucleotidyltransferase	421	20	0	89.25	1	F:GO:0003674	F:molecular_function			G3DSA:3.30.460.10 (GENE3D); IPR040511 (PFAM); PF18144 (PFAM); cd05400 (CDD); SSF81301 (SUPERFAMILY)	no GO terms	no GO terms
374	CP020704.1	Pf-tRNA-Met	I	Uncharacterised protein	330	20	0	84.71						no IPS match	no IPS match	no IPS match
375	CP021774.1	Pf-DR	I	hypothetical protein	159	20	1.01E-111	79.81	1	C:GO:0005575	C:cellular_component			NON_CYTOPLASMIC_DOMAIN (PHOBIUS); SIGNAL_PEPTIDE_C_REGION (PHOBIUS); SIGNAL_PEPTIDE_N_REGION (PHOBIUS); SIGNAL_PEPTIDE_H_REGION (PHOBIUS); SIGNAL_PEPTIDE (PHOBIUS); TMhelix (TMHMM)	no GO terms	no GO terms
376	CP021774.1	Pf-DR	I	Uncharacterised protein	543	20	0	74.49	1	C:GO:0005575	C:cellular_component			TRANSMEMBRANE (PHOBIUS); TRANSMEMBRANE (PHOBIUS); CYTOPLASMIC_DOMAIN (PHOBIUS); NON_CYTOPLASMIC_DOMAIN (PHOBIUS); NON_CYTOPLASMIC_DOMAIN (PHOBIUS); CYTOPLASMIC_DOMAIN (PHOBIUS); TRANSMEMBRANE (PHOBIUS); TMhelix (TMHMM); TMhelix (TMHMM); TMhelix (TMHMM)	no GO terms	no GO terms
377	CP021775.1	Pf-tRNA-Gly	I	type II toxin-antitoxin system RelE/ParE family toxin	115	20	2.09E-76	98.57						IPR007712 (PFAM); IPR035093 (G3DSA:3.30.2310.GENE3D)	no GO terms	no GO terms
378	CP021775.1	Pf-tRNA-Gly	I	type II toxin-antitoxin system Phd/YefM family antitoxin	83	20	6.81E-53	96.37						PTHR33713:SF7 (PANTHER); PTHR33713 (PANTHER); IPR036165 (SUPERFAMILY)	no GO terms	no GO terms
379	CP021775.1	Pf-tRNA-Gly	I	Uncharacterised protein	97	20	1.56E-65	92.06						no IPS match	no IPS match	no IPS match
380	CP021775.1	Pf-tRNA-Gly	I	Uncharacterised protein	134	20	7.46E-88	97.01						no IPS match	no IPS match	no IPS match
381	CP021775.1	Pf-tRNA-Gly	I	Uncharacterised protein	232	20	1.20E-174	83.08	1	C:GO:0005575	C:cellular_component			mobidb-lite (MOBIDB_LITE); NON_CYTOPLASMIC_DOMAIN (PHOBIUS); CYTOPLASMIC_DOMAIN (PHOBIUS); TRANSMEMBRANE (PHOBIUS); TMhelix (TMHMM)	no GO terms	no GO terms
382	CP021775.1	Pf-tRNA-Gly	I	Uncharacterised protein	275	20	0	73.59						no IPS match	no IPS match	no IPS match
383	CP021775.1	Pf-tRNA-Gly	I	Uncharacterised protein	244	20	3.24E-180	67.32	1	C:GO:0005575	C:cellular_component			Coil (COILS); SIGNAL_PEPTIDE_C_REGION (PHOBIUS); SIGNAL_PEPTIDE_N_REGION (PHOBIUS); TRANSMEMBRANE (PHOBIUS); CYTOPLASMIC_DOMAIN (PHOBIUS); NON_CYTOPLASMIC_DOMAIN (PHOBIUS); SIGNAL_PEPTIDE (PHOBIUS); SIGNAL_PEPTIDE_H_REGION (PHOBIUS); SignalP-TM (SIGNALP_EUK); TMhelix (TMHMM); TMhelix (TMHMM)	no GO terms	no GO terms
384	CP021775.1	Pf-tRNA-Gly	I	Uncharacterised protein	227	20	5.69E-163	62.41	1	C:GO:0005575	C:cellular_component			Coil (COILS); TRANSMEMBRANE (PHOBIUS); NON_CYTOPLASMIC_DOMAIN (PHOBIUS); TRANSMEMBRANE (PHOBIUS); NON_CYTOPLASMIC_DOMAIN (PHOBIUS); CYTOPLASMIC_DOMAIN (PHOBIUS); TMhelix (TMHMM); TMhelix (TMHMM)	no GO terms	no GO terms
385	CP021775.1	Pf-tRNA-Gly	I	Uncharacterised protein	98	20	1.43E-64	56.51						no IPS match	no IPS match	no IPS match
386	CP021775.1	Pf-tRNA-Gly	I	IS4 family transposase	295	20	0	99.97	3	P:GO:0006259; P:GO:0032196; F:GO:0003677	P:DNA metabolic process; P:transposition; F:DNA binding			IPR002559 (PFAM); PTHR33258 (PANTHER); IPR012337 (SUPERFAMILY)	P:GO:0006313; F:GO:0003677; F:GO:0004803	P:transposition, DNA-mediated; F:DNA binding; F:transposase activity
387	CP021775.1	Pf-tRNA-Met	I	Uncharacterised protein	145	20	6.89E-100	95.34	1	C:GO:0005575	C:cellular_component			TRANSMEMBRANE (PHOBIUS); CYTOPLASMIC_DOMAIN (PHOBIUS); TRANSMEMBRANE (PHOBIUS); CYTOPLASMIC_DOMAIN (PHOBIUS); NON_CYTOPLASMIC_DOMAIN (PHOBIUS); TMhelix (TMHMM); TMhelix (TMHMM)	no GO terms	no GO terms
388	CP021775.1	Pf-tRNA-Met	I	Uncharacterised protein	111	20	3.44E-74	91.97						SIGNAL_PEPTIDE_C_REGION (PHOBIUS); SIGNAL_PEPTIDE_H_REGION (PHOBIUS); SIGNAL_PEPTIDE_N_REGION (PHOBIUS); NON_CYTOPLASMIC_DOMAIN (PHOBIUS); SIGNAL_PEPTIDE (PHOBIUS); SignalP-noTM (SIGNALP_GRAM_NEGATIVE); SignalP-noTM (SIGNALP_EUK); SignalP-TM (SIGNALP_GRAM_POSITIVE)	no GO terms	no GO terms
389	CP021775.1	Pf-tRNA-Met	I	SLATT domain-containing protein	196	20	2.86E-141	81.86	1	C:GO:0005575	C:cellular_component			IPR041119 (PFAM); NON_CYTOPLASMIC_DOMAIN (PHOBIUS); CYTOPLASMIC_DOMAIN (PHOBIUS); TRANSMEMBRANE (PHOBIUS); TRANSMEMBRANE (PHOBIUS); NON_CYTOPLASMIC_DOMAIN (PHOBIUS); TRANSMEMBRANE (PHOBIUS); CYTOPLASMIC_DOMAIN (PHOBIUS); TMhelix (TMHMM); TMhelix (TMHMM); TMhelix (TMHMM)	no GO terms	no GO terms
390	CP021775.1	Pf-tRNA-Met	I	nucleotidyltransferase	421	20	0	89.25	1	F:GO:0003674	F:molecular_function			PF18144 (PFAM); G3DSA:3.30.460.10 (GENE3D); IPR040511 (PFAM); cd05400 (CDD); SSF81301 (SUPERFAMILY)	no GO terms	no GO terms
391	CP021775.1	Pf-tRNA-Met	I	Uncharacterised protein	330	20	0	84.71						no IPS match	no IPS match	no IPS match
392	CP021999.1	Pf-tRNA-Gly	I	type II toxin-antitoxin system RelE/ParE family toxin	115	20	6.73E-77	97.56						IPR007712 (PFAM); IPR035093 (G3DSA:3.30.2310.GENE3D)	no GO terms	no GO terms
393	CP021999.1	Pf-tRNA-Gly	I	type II toxin-antitoxin system Phd/YefM family antitoxin	83	20	6.81E-53	96.37						PTHR33713:SF7 (PANTHER); PTHR33713 (PANTHER); IPR036165 (SUPERFAMILY)	no GO terms	no GO terms
394	CP021999.1	Pf-tRNA-Gly	I	Uncharacterised protein	230	20	2.17E-172	95.22	1	C:GO:0005575	C:cellular_component			mobidb-lite (MOBIDB_LITE); mobidb-lite (MOBIDB_LITE); mobidb-lite (MOBIDB_LITE); mobidb-lite (MOBIDB_LITE); TRANSMEMBRANE (PHOBIUS); NON_CYTOPLASMIC_DOMAIN (PHOBIUS); CYTOPLASMIC_DOMAIN (PHOBIUS); TMhelix (TMHMM)	no GO terms	no GO terms
395	CP021999.1	Pf-tRNA-Gly	I	NYN domain-containing protein	219	20	2.08E-162	60.38						no IPS match	no IPS match	no IPS match
396	CP021999.1	Pf-tRNA-Gly	I	NACHT domain-containing protein	714	20	0	74.51						IPR027417 (SUPERFAMILY)	no GO terms	no GO terms
397	CP022000.1	Pf-DR	I	hypothetical protein	159	20	1.01E-111	79.81	1	C:GO:0005575	C:cellular_component			NON_CYTOPLASMIC_DOMAIN (PHOBIUS); SIGNAL_PEPTIDE_C_REGION (PHOBIUS); SIGNAL_PEPTIDE_N_REGION (PHOBIUS); SIGNAL_PEPTIDE_H_REGION (PHOBIUS); SIGNAL_PEPTIDE (PHOBIUS); TMhelix (TMHMM)	no GO terms	no GO terms
398	CP022000.1	Pf-DR	I	Uncharacterised protein	543	20	0	74.49	1	C:GO:0005575	C:cellular_component			TRANSMEMBRANE (PHOBIUS); TRANSMEMBRANE (PHOBIUS); CYTOPLASMIC_DOMAIN (PHOBIUS); NON_CYTOPLASMIC_DOMAIN (PHOBIUS); NON_CYTOPLASMIC_DOMAIN (PHOBIUS); CYTOPLASMIC_DOMAIN (PHOBIUS); TRANSMEMBRANE (PHOBIUS); TMhelix (TMHMM); TMhelix (TMHMM); TMhelix (TMHMM)	no GO terms	no GO terms
399	CP022001.1	Pf-tRNA-Gly	II	Uncharacterised protein	230	20	2.58E-173	96.26	1	C:GO:0005575	C:cellular_component			mobidb-lite (MOBIDB_LITE); mobidb-lite (MOBIDB_LITE); mobidb-lite (MOBIDB_LITE); TRANSMEMBRANE (PHOBIUS); NON_CYTOPLASMIC_DOMAIN (PHOBIUS); CYTOPLASMIC_DOMAIN (PHOBIUS); TMhelix (TMHMM)	no GO terms	no GO terms
400	CP022001.1	Pf-tRNA-Gly	II	Uncharacterised protein	94	20	6.00E-53	82.83						mobidb-lite (MOBIDB_LITE)	no GO terms	no GO terms
401	CP022001.1	Pf-tRNA-Gly	II	Uncharacterised protein	430	20	0	73.9						no IPS match	no IPS match	no IPS match
402	CP022001.1	Pf-tRNA-Gly	II	type II toxin-antitoxin system Phd/YefM family antitoxin	83	20	6.81E-53	96.37						PTHR33713:SF7 (PANTHER); PTHR33713 (PANTHER); IPR036165 (SUPERFAMILY)	no GO terms	no GO terms
403	CP022001.1	Pf-tRNA-Gly	II	type II toxin-antitoxin system RelE/ParE family toxin	115	20	5.90E-77	98.35						IPR007712 (PFAM); IPR035093 (G3DSA:3.30.2310.GENE3D)	no GO terms	no GO terms
404	CP022001.1	Pf-tRNA-Gly	II	IS256 family transposase	398	20	0	98.56	3	P:GO:0006259; P:GO:0032196; F:GO:0003677	P:DNA metabolic process; P:transposition; F:DNA binding			IPR001207 (PFAM); IPR001207 (PANTHER); PTHR33217:SF7 (PANTHER)	P:GO:0006313; F:GO:0003677; F:GO:0004803	P:transposition, DNA-mediated; F:DNA binding; F:transposase activity
405	CP022002.1a	Pf-tRNA-Gly	II	Uncharacterised protein	242	20	1.41E-174	96.08	1	C:GO:0005575	C:cellular_component			mobidb-lite (MOBIDB_LITE); mobidb-lite (MOBIDB_LITE); mobidb-lite (MOBIDB_LITE); mobidb-lite (MOBIDB_LITE); mobidb-lite (MOBIDB_LITE); TRANSMEMBRANE (PHOBIUS); CYTOPLASMIC_DOMAIN (PHOBIUS); NON_CYTOPLASMIC_DOMAIN (PHOBIUS); TMhelix (TMHMM)	no GO terms	no GO terms
406	CP022002.1a	Pf-tRNA-Gly	II	Uncharacterised protein	200	20	1.75E-145	70.47						no IPS match	no IPS match	no IPS match
407	CP022002.1a	Pf-tRNA-Gly	II	Uncharacterised protein	211	20	4.45E-155	73.36						no IPS match	no IPS match	no IPS match
408	CP022002.1a	Pf-tRNA-Gly	II	type II toxin-antitoxin system Phd/YefM family antitoxin	83	20	6.81E-53	96.37						PTHR33713:SF7 (PANTHER); PTHR33713 (PANTHER); IPR036165 (SUPERFAMILY)	no GO terms	no GO terms
409	CP022002.1a	Pf-tRNA-Gly	II	type II toxin-antitoxin system RelE/ParE family toxin	115	20	1.69E-76	97.87						IPR035093 (G3DSA:3.30.2310.GENE3D); IPR007712 (PFAM)	no GO terms	no GO terms
410	CP022525.1	Pf-tRNA-Gly	II	type II toxin-antitoxin system Phd/YefM family antitoxin	83	20	6.81E-53	96.37						PTHR33713:SF7 (PANTHER); PTHR33713 (PANTHER); IPR036165 (SUPERFAMILY)	no GO terms	no GO terms
411	CP022525.1	Pf-tRNA-Gly	II	type II toxin-antitoxin system RelE/ParE family toxin	115	20	9.26E-77	97.91						IPR007712 (PFAM); IPR035093 (G3DSA:3.30.2310.GENE3D)	no GO terms	no GO terms
412	CP022525.1	Pf-tRNA-Gly	II	MULTISPECIES: hypothetical protein	232	20	1.39E-174	95.54	1	C:GO:0005575	C:cellular_component			mobidb-lite (MOBIDB_LITE); mobidb-lite (MOBIDB_LITE); TRANSMEMBRANE (PHOBIUS); NON_CYTOPLASMIC_DOMAIN (PHOBIUS); CYTOPLASMIC_DOMAIN (PHOBIUS); TMhelix (TMHMM)	no GO terms	no GO terms
413	CP022525.1	Pf-tRNA-Gly	II	Uncharacterised protein	360	20	0	67.47						no IPS match	no IPS match	no IPS match
414	CP022525.1	Pf-tRNA-Met	I	hypothetical protein	136	20	1.37E-94	99.74	1	C:GO:0005575	C:cellular_component			CYTOPLASMIC_DOMAIN (PHOBIUS); CYTOPLASMIC_DOMAIN (PHOBIUS); TRANSMEMBRANE (PHOBIUS); NON_CYTOPLASMIC_DOMAIN (PHOBIUS); NON_CYTOPLASMIC_DOMAIN (PHOBIUS); TRANSMEMBRANE (PHOBIUS); TRANSMEMBRANE (PHOBIUS); TMhelix (TMHMM); TMhelix (TMHMM); TMhelix (TMHMM)	no GO terms	no GO terms
415	CP022525.1	Pf-tRNA-Met	I	thymidylate synthase	65	20	3.73E-34	85.21	4	P:GO:0009058; P:GO:0034641; P:GO:0044281; F:GO:0008168	P:biosynthetic process; P:cellular nitrogen compound metabolic process; P:small molecule metabolic process; F:methyltransferase activity	EC:2.1.1.45	Thymidylate synthase	no IPS match	no IPS match	no IPS match
416	CP022525.1	Pf-tRNA-Met	I	Thymidylate synthase	265	20	0	77.45	2	F:GO:0003674	F:molecular_function	EC:2.1.1.45	Thymidylate synthase	no IPS match	no IPS match	no IPS match
417	CP022525.1	Pf-tRNA-Met	I	hypothetical protein	311	20	0	76.39						IPR041271 (PFAM)	no GO terms	no GO terms
418	CP022525.1	Pf-tRNA-Met	I	ImmA/IrrE family metallo-endopeptidase	362	20	0	74.33	1	F:GO:0003677	F:DNA binding			G3DSA:1.10.260.40 (GENE3D); IPR010359 (PFAM); IPR001387 (CDD); IPR010982 (SUPERFAMILY)	F:GO:0003677	F:DNA binding
419	CP022525.1	Pf-tRNA-Met	I	IS30 family transposase ISPpu17	320	20	0	99.08	4	P:GO:0006313; P:GO:0015074; F:GO:0003677; F:GO:0004803	P:transposition, DNA-mediated; P:DNA integration; F:DNA binding; F:transposase activity			IPR036397 (G3DSA:3.30.420.GENE3D); IPR001584 (PFAM); IPR025246 (PFAM); PTHR10948 (PANTHER); IPR001598 (PROSITE_PATTERNS); IPR001584 (PROSITE_PROFILES); IPR012337 (SUPERFAMILY)	P:GO:0006313; P:GO:0015074; F:GO:0003676; F:GO:0003677; F:GO:0004803	P:transposition, DNA-mediated; P:DNA integration; F:nucleic acid binding; F:DNA binding; F:transposase activity
420	CP022525.1	Pf-tRNA-Met	I	thymidylate synthase	173	20	1.03E-124	79.7	4	P:GO:0008150; F:GO:0008168	P:biological_process; F:methyltransferase activity	EC:2.1.1.45	Thymidylate synthase	IPR036926 (G3DSA:3.30.572.GENE3D); IPR023451 (PFAM); PTHR11548:SF9 (PANTHER); PTHR11548 (PANTHER); IPR036926 (SUPERFAMILY)	no GO terms	no GO terms
421	CP022526.1	Pf-tRNA-Gly	II	type II toxin-antitoxin system RelE/ParE family toxin	115	20	9.26E-77	97.91						IPR007712 (PFAM); IPR035093 (G3DSA:3.30.2310.GENE3D)	no GO terms	no GO terms
422	CP022526.1	Pf-tRNA-Gly	II	type II toxin-antitoxin system Phd/YefM family antitoxin	83	20	6.81E-53	96.37						PTHR33713:SF7 (PANTHER); PTHR33713 (PANTHER); IPR036165 (SUPERFAMILY)	no GO terms	no GO terms
423	CP022526.1	Pf-tRNA-Gly	II	MULTISPECIES: hypothetical protein	232	20	1.39E-174	95.54	1	C:GO:0005575	C:cellular_component			mobidb-lite (MOBIDB_LITE); mobidb-lite (MOBIDB_LITE); TRANSMEMBRANE (PHOBIUS); NON_CYTOPLASMIC_DOMAIN (PHOBIUS); CYTOPLASMIC_DOMAIN (PHOBIUS); TMhelix (TMHMM)	no GO terms	no GO terms
424	CP022526.1	Pf-tRNA-Gly	II	Uncharacterised protein	360	20	0	67.47						no IPS match	no IPS match	no IPS match
425	CP022526.1	Pf-tRNA-Met	I	hypothetical protein	136	20	1.37E-94	99.74	1	C:GO:0005575	C:cellular_component			CYTOPLASMIC_DOMAIN (PHOBIUS); CYTOPLASMIC_DOMAIN (PHOBIUS); TRANSMEMBRANE (PHOBIUS); NON_CYTOPLASMIC_DOMAIN (PHOBIUS); NON_CYTOPLASMIC_DOMAIN (PHOBIUS); TRANSMEMBRANE (PHOBIUS); TRANSMEMBRANE (PHOBIUS); TMhelix (TMHMM); TMhelix (TMHMM); TMhelix (TMHMM)	no GO terms	no GO terms
426	CP022526.1	Pf-tRNA-Met	I	thymidylate synthase	65	20	3.73E-34	85.21	4	P:GO:0009058; P:GO:0034641; P:GO:0044281; F:GO:0008168	P:biosynthetic process; P:cellular nitrogen compound metabolic process; P:small molecule metabolic process; F:methyltransferase activity	EC:2.1.1.45	Thymidylate synthase	no IPS match	no IPS match	no IPS match
427	CP022526.1	Pf-tRNA-Met	I	Thymidylate synthase	265	20	0	77.45	2	F:GO:0003674	F:molecular_function	EC:2.1.1.45	Thymidylate synthase	no IPS match	no IPS match	no IPS match
428	CP022526.1	Pf-tRNA-Met	I	hypothetical protein	326	20	0	75.25						IPR041271 (PFAM)	no GO terms	no GO terms
429	CP022526.1	Pf-tRNA-Met	I	ImmA/IrrE family metallo-endopeptidase	362	20	0	74.33	1	F:GO:0003677	F:DNA binding			G3DSA:1.10.260.40 (GENE3D); IPR010359 (PFAM); IPR001387 (CDD); IPR010982 (SUPERFAMILY)	F:GO:0003677	F:DNA binding
430	CP022526.1	Pf-tRNA-Met	I	IS30 family transposase ISPpu17	320	20	0	99.08	4	P:GO:0006313; P:GO:0015074; F:GO:0003677; F:GO:0004803	P:transposition, DNA-mediated; P:DNA integration; F:DNA binding; F:transposase activity			IPR036397 (G3DSA:3.30.420.GENE3D); IPR001584 (PFAM); IPR025246 (PFAM); PTHR10948 (PANTHER); IPR001598 (PROSITE_PATTERNS); IPR001584 (PROSITE_PROFILES); IPR012337 (SUPERFAMILY)	P:GO:0006313; P:GO:0015074; F:GO:0003676; F:GO:0003677; F:GO:0004803	P:transposition, DNA-mediated; P:DNA integration; F:nucleic acid binding; F:DNA binding; F:transposase activity
431	CP022526.1	Pf-tRNA-Met	I	thymidylate synthase	173	20	1.03E-124	79.7	4	P:GO:0008150; F:GO:0008168	P:biological_process; F:methyltransferase activity	EC:2.1.1.45	Thymidylate synthase	IPR036926 (G3DSA:3.30.572.GENE3D); IPR023451 (PFAM); PTHR11548:SF9 (PANTHER); PTHR11548 (PANTHER); IPR036926 (SUPERFAMILY)	no GO terms	no GO terms
432	CP023255.1	Pf-tRNA-Gly	I	Fis family transcriptional regulator	369	20	0	88.35	2	F:GO:0003677; F:GO:0043167	F:DNA binding; F:ion binding			Coil (COILS); IPR003593 (SMART); G3DSA:3.40.50.300 (GENE3D); IPR003959 (PFAM); IPR025246 (PFAM); PTHR23073:SF29 (PANTHER); PTHR23073 (PANTHER); cd00009 (CDD); IPR027417 (SUPERFAMILY)	F:GO:0005524	F:ATP binding
433	CP023255.1	Pf-tRNA-Gly	I	Uncharacterised protein	74	20	3.18E-47	84.96						no IPS match	no IPS match	no IPS match
434	CP023255.1	Pf-tRNA-Gly	I	Uncharacterised protein	230	20	3.91E-173	96.98	1	C:GO:0005575	C:cellular_component			mobidb-lite (MOBIDB_LITE); mobidb-lite (MOBIDB_LITE); mobidb-lite (MOBIDB_LITE); TRANSMEMBRANE (PHOBIUS); NON_CYTOPLASMIC_DOMAIN (PHOBIUS); CYTOPLASMIC_DOMAIN (PHOBIUS); TMhelix (TMHMM)	no GO terms	no GO terms
435	CP023255.1	Pf-tRNA-Gly	I	Uncharacterised protein	165	20	5.14E-96	63.27	1	C:GO:0005575	C:cellular_component			CYTOPLASMIC_DOMAIN (PHOBIUS); NON_CYTOPLASMIC_DOMAIN (PHOBIUS); TRANSMEMBRANE (PHOBIUS)	no GO terms	no GO terms
436	CP023255.1	Pf-tRNA-Gly	I	S8 family peptidase	732	20	0	88.19	1	F:GO:0008233	F:peptidase activity	EC:3.4.21	Acting on peptide bonds (peptidases)	IPR036852 (G3DSA:3.40.50.GENE3D); IPR000209 (PFAM); IPR034074 (CDD); IPR036852 (SUPERFAMILY)	P:GO:0006508; F:GO:0004252	P:proteolysis; F:serine-type endopeptidase activity
437	CP023255.1	Pf-tRNA-Met	I	Uncharacterised protein	145	20	3.37E-100	93.45	1	C:GO:0005575	C:cellular_component			Coil (COILS); TRANSMEMBRANE (PHOBIUS); CYTOPLASMIC_DOMAIN (PHOBIUS); TRANSMEMBRANE (PHOBIUS); CYTOPLASMIC_DOMAIN (PHOBIUS); NON_CYTOPLASMIC_DOMAIN (PHOBIUS); TMhelix (TMHMM); TMhelix (TMHMM)	no GO terms	no GO terms
438	CP023255.1	Pf-tRNA-Met	I	MULTISPECIES: hypothetical protein	111	20	1.68E-75	97.16						SIGNAL_PEPTIDE_H_REGION (PHOBIUS); SIGNAL_PEPTIDE_N_REGION (PHOBIUS); SIGNAL_PEPTIDE_C_REGION (PHOBIUS); NON_CYTOPLASMIC_DOMAIN (PHOBIUS); SIGNAL_PEPTIDE (PHOBIUS); SignalP-TM (SIGNALP_GRAM_POSITIVE); SignalP-noTM (SIGNALP_EUK); SignalP-noTM (SIGNALP_GRAM_NEGATIVE)	no GO terms	no GO terms
439	CP023255.1	Pf-tRNA-Met	I	AAA family ATPase	405	20	0	81.8	1	F:GO:0043167	F:ion binding			IPR003959 (PFAM); G3DSA:3.40.50.300 (GENE3D); IPR014555 (PIRSF); G3DSA:3.40.50.300 (GENE3D); PTHR32182 (PANTHER); PTHR32182:SF22 (PANTHER); PTHR32182 (PANTHER); TRANSMEMBRANE (PHOBIUS); CYTOPLASMIC_DOMAIN (PHOBIUS); NON_CYTOPLASMIC_DOMAIN (PHOBIUS); IPR027417 (SUPERFAMILY)	F:GO:0005524	F:ATP binding
440	CP023255.1	Pf-tRNA-Met	I	Uncharacterised protein	242	20	0	61.66						no IPS match	no IPS match	no IPS match
441	CP024477.1	Pf-tRNA-Gly	I	type II toxin-antitoxin system RelE/ParE family toxin	115	20	2.09E-76	98.57						IPR007712 (PFAM); IPR035093 (G3DSA:3.30.2310.GENE3D)	no GO terms	no GO terms
442	CP024477.1	Pf-tRNA-Gly	I	type II toxin-antitoxin system Phd/YefM family antitoxin	83	20	9.36E-53	96.24						PTHR33713 (PANTHER); PTHR33713:SF7 (PANTHER); IPR036165 (SUPERFAMILY)	no GO terms	no GO terms
443	CP024477.1	Pf-tRNA-Gly	I	hypothetical protein	230	20	5.62E-166	55.31	1	C:GO:0005575	C:cellular_component			TRANSMEMBRANE (PHOBIUS); NON_CYTOPLASMIC_DOMAIN (PHOBIUS); TRANSMEMBRANE (PHOBIUS); NON_CYTOPLASMIC_DOMAIN (PHOBIUS); TRANSMEMBRANE (PHOBIUS); CYTOPLASMIC_DOMAIN (PHOBIUS); CYTOPLASMIC_DOMAIN (PHOBIUS); TMhelix (TMHMM); TMhelix (TMHMM); TMhelix (TMHMM)	no GO terms	no GO terms
444	CP024477.1	Pf-tRNA-Gly	I	type 1 glutamine amidotransferase	119	7	2.02E-83	80.02	1	P:GO:0006520	P:cellular amino acid metabolic process			no IPS match	no IPS match	no IPS match
445	CP024477.1	Pf-tRNA-Met	I	hypothetical protein E6J34_00200	90	20	5.85E-13	58.96						no IPS match	no IPS match	no IPS match
446	CP024477.1	Pf-tRNA-Met	I	AAA family ATPase	376	20	0	71.31	1	F:GO:0043167	F:ion binding			IPR003593 (SMART); IPR003959 (PFAM); G3DSA:3.40.50.300 (GENE3D); PTHR23073 (PANTHER); PTHR23073:SF29 (PANTHER); cd00009 (CDD); IPR027417 (SUPERFAMILY)	F:GO:0005524	F:ATP binding
447	CP024477.1	Pf-tRNA-Met	I	hypothetical protein	136	20	1.76E-94	99.74	1	C:GO:0005575	C:cellular_component			CYTOPLASMIC_DOMAIN (PHOBIUS); CYTOPLASMIC_DOMAIN (PHOBIUS); TRANSMEMBRANE (PHOBIUS); NON_CYTOPLASMIC_DOMAIN (PHOBIUS); NON_CYTOPLASMIC_DOMAIN (PHOBIUS); TRANSMEMBRANE (PHOBIUS); TRANSMEMBRANE (PHOBIUS); TMhelix (TMHMM); TMhelix (TMHMM); TMhelix (TMHMM)	no GO terms	no GO terms
448	CP024477.1	Pf-tRNA-Met	I	hypothetical protein	111	20	6.21E-75	97.84						SIGNAL_PEPTIDE_H_REGION (PHOBIUS); SIGNAL_PEPTIDE_N_REGION (PHOBIUS); SIGNAL_PEPTIDE_C_REGION (PHOBIUS); NON_CYTOPLASMIC_DOMAIN (PHOBIUS); SIGNAL_PEPTIDE (PHOBIUS); SignalP-TM (SIGNALP_GRAM_POSITIVE); SignalP-noTM (SIGNALP_EUK); SignalP-noTM (SIGNALP_GRAM_NEGATIVE)	no GO terms	no GO terms
449	CP024477.1	Pf-tRNA-Met	I	hypothetical protein	93	20	5.04E-60	67.27	1	C:GO:0005575	C:cellular_component			CYTOPLASMIC_DOMAIN (PHOBIUS); NON_CYTOPLASMIC_DOMAIN (PHOBIUS); NON_CYTOPLASMIC_DOMAIN (PHOBIUS); TRANSMEMBRANE (PHOBIUS); TRANSMEMBRANE (PHOBIUS); TRANSMEMBRANE (PHOBIUS); CYTOPLASMIC_DOMAIN (PHOBIUS); TMhelix (TMHMM); TMhelix (TMHMM); TMhelix (TMHMM)	no GO terms	no GO terms
450	CP024477.1	Pf-tRNA-Met	I	S8 family peptidase	735	20	0	76.99	1	F:GO:0008233	F:peptidase activity	EC:3.4.21	Acting on peptide bonds (peptidases)	Coil (COILS); IPR000209 (PFAM); IPR036852 (G3DSA:3.40.50.GENE3D); PTHR43399 (PANTHER); IPR034074 (CDD); IPR036852 (SUPERFAMILY)	P:GO:0006508; F:GO:0004252	P:proteolysis; F:serine-type endopeptidase activity
451	CP025049.1	Pf-tRNA-Gly	I	Uncharacterised protein	128	20	8.49E-84	86.62	1	C:GO:0005575	C:cellular_component			mobidb-lite (MOBIDB_LITE); mobidb-lite (MOBIDB_LITE); NON_CYTOPLASMIC_DOMAIN (PHOBIUS); TRANSMEMBRANE (PHOBIUS); CYTOPLASMIC_DOMAIN (PHOBIUS); TMhelix (TMHMM)	no GO terms	no GO terms
452	CP025049.1	Pf-tRNA-Gly	I	type II toxin-antitoxin system RelE/ParE family toxin	115	20	1.41E-76	98.87						IPR035093 (G3DSA:3.30.2310.GENE3D); IPR007712 (PFAM)	no GO terms	no GO terms
453	CP025049.1	Pf-tRNA-Gly	I	type II toxin-antitoxin system Phd/YefM family antitoxin	83	20	6.81E-53	96.37						PTHR33713:SF7 (PANTHER); PTHR33713 (PANTHER); IPR036165 (SUPERFAMILY)	no GO terms	no GO terms
454	CP025049.1	Pf-tRNA-Gly	I	FRG domain-containing protein	254	20	0	71.61						IPR014966 (SMART); IPR014966 (PFAM)	no GO terms	no GO terms
455	CP025049.1	Pf-tRNA-Gly	I	DUF262 domain-containing protein	350	20	0	68.51						IPR004919 (PFAM); PTHR39639 (PANTHER)	no GO terms	no GO terms
456	CP025050.1	Pf-tRNA-Gly	I	type II toxin-antitoxin system RelE/ParE family toxin	115	20	1.41E-76	98.87						IPR035093 (G3DSA:3.30.2310.GENE3D); IPR007712 (PFAM)	no GO terms	no GO terms
457	CP025050.1	Pf-tRNA-Gly	I	type II toxin-antitoxin system Phd/YefM family antitoxin	83	20	6.81E-53	96.37						PTHR33713:SF7 (PANTHER); PTHR33713 (PANTHER); IPR036165 (SUPERFAMILY)	no GO terms	no GO terms
458	CP025050.1	Pf-tRNA-Gly	I	Uncharacterised protein	128	20	8.49E-84	86.62	1	C:GO:0005575	C:cellular_component			mobidb-lite (MOBIDB_LITE); mobidb-lite (MOBIDB_LITE); NON_CYTOPLASMIC_DOMAIN (PHOBIUS); TRANSMEMBRANE (PHOBIUS); CYTOPLASMIC_DOMAIN (PHOBIUS); TMhelix (TMHMM)	no GO terms	no GO terms
459	CP025050.1	Pf-tRNA-Gly	I	FRG domain-containing protein	254	20	0	71.61						IPR014966 (SMART); IPR014966 (PFAM)	no GO terms	no GO terms
460	CP025050.1	Pf-tRNA-Gly	I	DUF262 domain-containing protein	350	20	0	68.51						IPR004919 (PFAM); PTHR39639 (PANTHER)	no GO terms	no GO terms
461	CP027165.1	Pf-tRNA-Gly	I	pyruvate:ferredoxin (flavodoxin) oxidoreductase	68	20	9.53E-24	80.75	5	P:GO:0006091; P:GO:0007155; F:GO:0016491; F:GO:0043167; C:GO:0005575	P:generation of precursor metabolites and energy; P:cell adhesion; F:oxidoreductase activity; F:ion binding; C:cellular_component			mobidb-lite (MOBIDB_LITE)	no GO terms	no GO terms
462	CP027165.1	Pf-tRNA-Gly	I	Uncharacterised protein	43	20	1.16E-15	72.39						no IPS match	no IPS match	no IPS match
463	CP027165.1	Pf-tRNA-Gly	I	AAA family ATPase	441	20	0	78.05	1	F:GO:0043167	F:ion binding			IPR003593 (SMART); IPR038729 (PFAM); IPR003959 (PFAM); G3DSA:3.40.50.300 (GENE3D); PTHR43581:SF2 (PANTHER); PTHR43581 (PANTHER); IPR027417 (SUPERFAMILY)	F:GO:0005524	F:ATP binding
464	CP027165.1	Pf-tRNA-Gly	I	RNA-directed DNA polymerase	283	20	0	80.53	4	P:GO:0006259; P:GO:0009058; F:GO:0003723; F:GO:0016779	P:DNA metabolic process; P:biosynthetic process; F:RNA binding; F:nucleotidyltransferase activity	EC:2.7.7.49	RNA-directed DNA polymerase	IPR000123 (PRINTS); IPR000477 (PFAM); PTHR34047:SF6 (PANTHER); PTHR34047 (PANTHER); IPR000477 (PROSITE_PROFILES); cd03487 (CDD); SSF56672 (SUPERFAMILY)	P:GO:0006278; F:GO:0003723; F:GO:0003964	P:RNA-dependent DNA biosynthetic process; F:RNA binding; F:RNA-directed DNA polymerase activity
465	CP027165.1	Pf-tRNA-Met	II	hypothetical protein	136	20	1.66E-94	99.74	1	C:GO:0005575	C:cellular_component			CYTOPLASMIC_DOMAIN (PHOBIUS); CYTOPLASMIC_DOMAIN (PHOBIUS); TRANSMEMBRANE (PHOBIUS); NON_CYTOPLASMIC_DOMAIN (PHOBIUS); NON_CYTOPLASMIC_DOMAIN (PHOBIUS); TRANSMEMBRANE (PHOBIUS); TRANSMEMBRANE (PHOBIUS); TMhelix (TMHMM); TMhelix (TMHMM); TMhelix (TMHMM)	no GO terms	no GO terms
466	CP027165.1	Pf-tRNA-Met	II	Cro/CI family transcriptional regulator	264	20	0	86.3	7	P:GO:0001666; P:GO:0019217; P:GO:0045892; P:GO:0045926; P:GO:0045927; F:GO:0003677	P:response to hypoxia; P:regulation of fatty acid metabolic process; P:negative regulation of transcription, DNA-templated; P:negative regulation of growth; P:positive regulation of growth; F:DNA binding			IPR001387 (SMART); IPR007374 (SMART); IPR007374 (PFAM); IPR001387 (PFAM); G3DSA:1.10.260.40 (GENE3D); G3DSA:2.30.130.30 (GENE3D); IPR001387 (PROSITE_PROFILES); IPR001387 (CDD); IPR015947 (SUPERFAMILY); IPR010982 (SUPERFAMILY)	F:GO:0003677	F:DNA binding
467	CP027165.1	Pf-tRNA-Met	II	GNAT family N-acetyltransferase	512	20	0	98.36	1	F:GO:0016746	F:transferase activity, transferring acyl groups	EC:2.3.1.5	Arylamine N-acetyltransferase	IPR000182 (PFAM); G3DSA:3.40.630.30 (GENE3D); IPR000182 (PROSITE_PROFILES); cd18699 (CDD); IPR016181 (SUPERFAMILY)	F:GO:0008080	F:N-acetyltransferase activity
468	CP027165.1	Pf-tRNA-Met	II	AAA family ATPase	175	20	2.76E-124	83.12				EC:2.7.4.3	Adenylate kinase	PF13207 (PFAM); G3DSA:3.40.50.300 (GENE3D); IPR027417 (SUPERFAMILY)	no GO terms	no GO terms
469	CP027172.1	Pf-tRNA-Gly	I	type II toxin-antitoxin system RelE/ParE family toxin	115	20	2.09E-76	98.57						IPR007712 (PFAM); IPR035093 (G3DSA:3.30.2310.GENE3D)	no GO terms	no GO terms
470	CP027172.1	Pf-tRNA-Gly	I	type II toxin-antitoxin system Phd/YefM family antitoxin	83	20	6.81E-53	96.37						PTHR33713:SF7 (PANTHER); PTHR33713 (PANTHER); IPR036165 (SUPERFAMILY)	no GO terms	no GO terms
471	CP027172.1	Pf-tRNA-Gly	I	Uncharacterised protein	109	16	2.61E-73	69.06						no IPS match	no IPS match	no IPS match
472	CP027172.1	Pf-tRNA-Gly	I	Uncharacterised protein	227	20	5.69E-163	62.41	1	C:GO:0005575	C:cellular_component			Coil (COILS); TRANSMEMBRANE (PHOBIUS); NON_CYTOPLASMIC_DOMAIN (PHOBIUS); TRANSMEMBRANE (PHOBIUS); NON_CYTOPLASMIC_DOMAIN (PHOBIUS); CYTOPLASMIC_DOMAIN (PHOBIUS); TMhelix (TMHMM); TMhelix (TMHMM)	no GO terms	no GO terms
473	CP027172.1	Pf-tRNA-Gly	I	Uncharacterised protein	98	20	1.43E-64	56.51						no IPS match	no IPS match	no IPS match
474	CP027172.1	Pf-tRNA-Gly	I	Uncharacterised protein	244	20	3.24E-180	67.32	1	C:GO:0005575	C:cellular_component			Coil (COILS); SIGNAL_PEPTIDE_C_REGION (PHOBIUS); SIGNAL_PEPTIDE_N_REGION (PHOBIUS); TRANSMEMBRANE (PHOBIUS); CYTOPLASMIC_DOMAIN (PHOBIUS); NON_CYTOPLASMIC_DOMAIN (PHOBIUS); SIGNAL_PEPTIDE (PHOBIUS); SIGNAL_PEPTIDE_H_REGION (PHOBIUS); SignalP-TM (SIGNALP_EUK); TMhelix (TMHMM); TMhelix (TMHMM)	no GO terms	no GO terms
475	CP027172.1	Pf-tRNA-Gly	I	Uncharacterised protein	275	20	0	73.59						no IPS match	no IPS match	no IPS match
476	CP027172.1	Pf-tRNA-Gly	I	Uncharacterised protein	232	20	1.20E-174	83.08	1	C:GO:0005575	C:cellular_component			mobidb-lite (MOBIDB_LITE); NON_CYTOPLASMIC_DOMAIN (PHOBIUS); CYTOPLASMIC_DOMAIN (PHOBIUS); TRANSMEMBRANE (PHOBIUS); TMhelix (TMHMM)	no GO terms	no GO terms
477	CP027172.1	Pf-tRNA-Gly	I	Uncharacterised protein	97	20	1.56E-65	92.06						no IPS match	no IPS match	no IPS match
478	CP027172.1	Pf-tRNA-Met	I	Uncharacterised protein	330	20	0	84.71						no IPS match	no IPS match	no IPS match
479	CP027172.1	Pf-tRNA-Met	I	nucleotidyltransferase	421	20	0	89.25	1	F:GO:0003674	F:molecular_function			PF18144 (PFAM); G3DSA:3.30.460.10 (GENE3D); IPR040511 (PFAM); cd05400 (CDD); SSF81301 (SUPERFAMILY)	no GO terms	no GO terms
480	CP027172.1	Pf-tRNA-Met	I	SLATT domain-containing protein	196	20	2.86E-141	81.86	1	C:GO:0005575	C:cellular_component			IPR041119 (PFAM); NON_CYTOPLASMIC_DOMAIN (PHOBIUS); CYTOPLASMIC_DOMAIN (PHOBIUS); TRANSMEMBRANE (PHOBIUS); TRANSMEMBRANE (PHOBIUS); NON_CYTOPLASMIC_DOMAIN (PHOBIUS); TRANSMEMBRANE (PHOBIUS); CYTOPLASMIC_DOMAIN (PHOBIUS); TMhelix (TMHMM); TMhelix (TMHMM); TMhelix (TMHMM)	no GO terms	no GO terms
481	CP027172.1	Pf-tRNA-Met	I	Uncharacterised protein	111	20	3.44E-74	91.97						SIGNAL_PEPTIDE_C_REGION (PHOBIUS); SIGNAL_PEPTIDE_H_REGION (PHOBIUS); SIGNAL_PEPTIDE_N_REGION (PHOBIUS); NON_CYTOPLASMIC_DOMAIN (PHOBIUS); SIGNAL_PEPTIDE (PHOBIUS); SignalP-noTM (SIGNALP_EUK); SignalP-TM (SIGNALP_GRAM_POSITIVE); SignalP-noTM (SIGNALP_GRAM_NEGATIVE)	no GO terms	no GO terms
482	CP027172.1	Pf-tRNA-Met	I	Uncharacterised protein	145	20	6.89E-100	95.34	1	C:GO:0005575	C:cellular_component			TRANSMEMBRANE (PHOBIUS); CYTOPLASMIC_DOMAIN (PHOBIUS); TRANSMEMBRANE (PHOBIUS); CYTOPLASMIC_DOMAIN (PHOBIUS); NON_CYTOPLASMIC_DOMAIN (PHOBIUS); TMhelix (TMHMM); TMhelix (TMHMM)	no GO terms	no GO terms
483	CP028584.2	Pf-tRNA-Gly	II	type II toxin-antitoxin system RelE/ParE family toxin	115	20	1.69E-76	97.87						IPR035093 (G3DSA:3.30.2310.GENE3D); IPR007712 (PFAM)	no GO terms	no GO terms
484	CP028584.2	Pf-tRNA-Gly	II	type II toxin-antitoxin system Phd/YefM family antitoxin	83	20	6.81E-53	96.37						PTHR33713:SF7 (PANTHER); PTHR33713 (PANTHER); IPR036165 (SUPERFAMILY)	no GO terms	no GO terms
485	CP028584.2	Pf-tRNA-Gly	II	Uncharacterised protein	242	20	1.41E-174	96.08	1	C:GO:0005575	C:cellular_component			mobidb-lite (MOBIDB_LITE); mobidb-lite (MOBIDB_LITE); mobidb-lite (MOBIDB_LITE); mobidb-lite (MOBIDB_LITE); mobidb-lite (MOBIDB_LITE); TRANSMEMBRANE (PHOBIUS); CYTOPLASMIC_DOMAIN (PHOBIUS); NON_CYTOPLASMIC_DOMAIN (PHOBIUS); TMhelix (TMHMM)	no GO terms	no GO terms
486	CP028584.2	Pf-tRNA-Gly	II	Uncharacterised protein	200	20	1.75E-145	70.47						no IPS match	no IPS match	no IPS match
487	CP028584.2	Pf-tRNA-Gly	II	Uncharacterised protein	211	20	4.45E-155	73.36						no IPS match	no IPS match	no IPS match
488	CP028917.1	Pf-tRNA-Gly	II	DUF4238 domain-containing protein	344	20	0	82.72						IPR025332 (PFAM)	no GO terms	no GO terms
489	CP028917.1	Pf-tRNA-Gly	II	hypothetical protein Q038_00049	560	20	0	88.2	1	C:GO:0005575	C:cellular_component			no IPS match	no IPS match	no IPS match
490	CP028917.1	Pf-tRNA-Gly	II	Uncharacterised protein	242	20	1.47E-174	97.37	1	C:GO:0005575	C:cellular_component			mobidb-lite (MOBIDB_LITE); mobidb-lite (MOBIDB_LITE); mobidb-lite (MOBIDB_LITE); mobidb-lite (MOBIDB_LITE); TRANSMEMBRANE (PHOBIUS); CYTOPLASMIC_DOMAIN (PHOBIUS); NON_CYTOPLASMIC_DOMAIN (PHOBIUS); TMhelix (TMHMM)	no GO terms	no GO terms
491	CP028917.1	Pf-tRNA-Gly	II	DUF3892 domain-containing protein	94	20	1.20E-63	92.56						IPR024997 (PFAM); mobidb-lite (MOBIDB_LITE)	no GO terms	no GO terms
492	CP028917.1	Pf-tRNA-Met	I	MULTISPECIES: hypothetical protein	270	20	0	76.01						mobidb-lite (MOBIDB_LITE)	no GO terms	no GO terms
493	CP028917.1	Pf-tRNA-Met	I	Uncharacterised protein	110	20	2.86E-74	89.86						SIGNAL_PEPTIDE_H_REGION (PHOBIUS); NON_CYTOPLASMIC_DOMAIN (PHOBIUS); SIGNAL_PEPTIDE_N_REGION (PHOBIUS); SIGNAL_PEPTIDE_C_REGION (PHOBIUS); SIGNAL_PEPTIDE (PHOBIUS); SignalP-noTM (SIGNALP_EUK); SignalP-noTM (SIGNALP_GRAM_NEGATIVE); SignalP-TM (SIGNALP_GRAM_POSITIVE)	no GO terms	no GO terms
494	CP028917.1	Pf-tRNA-Met	I	ATP-dependent DNA helicase RecG	628	20	0	83.91	2	F:GO:0003674	F:molecular_function	EC:3.6.4.12	DNA helicase	IPR000504 (SMART); IPR007421 (PFAM); IPR038475 (G3DSA:3.30.565.GENE3D); IPR000504 (PFAM); IPR025831 (PFAM); IPR012677 (G3DSA:3.30.70.GENE3D); IPR038461 (G3DSA:3.30.950.GENE3D); PTHR30595 (PANTHER); PTHR30595:SF6 (PANTHER); IPR000504 (PROSITE_PROFILES); IPR035979 (SUPERFAMILY)	F:GO:0003676	F:nucleic acid binding
495	CP028917.1	Pf-tRNA-Met	I	hypothetical protein	48	20	1.03E-18	93.39						no IPS match	no IPS match	no IPS match
496	CP028917.1	Pf-tRNA-Met	I	hypothetical protein	136	20	1.68E-94	99.74	1	C:GO:0005575	C:cellular_component			CYTOPLASMIC_DOMAIN (PHOBIUS); CYTOPLASMIC_DOMAIN (PHOBIUS); TRANSMEMBRANE (PHOBIUS); NON_CYTOPLASMIC_DOMAIN (PHOBIUS); NON_CYTOPLASMIC_DOMAIN (PHOBIUS); TRANSMEMBRANE (PHOBIUS); TRANSMEMBRANE (PHOBIUS); TMhelix (TMHMM); TMhelix (TMHMM); TMhelix (TMHMM)	no GO terms	no GO terms
497	CP029088.1	Pf-tRNA-Met	I	hypothetical protein	136	20	1.37E-94	99.74	1	C:GO:0005575	C:cellular_component			CYTOPLASMIC_DOMAIN (PHOBIUS); CYTOPLASMIC_DOMAIN (PHOBIUS); TRANSMEMBRANE (PHOBIUS); TRANSMEMBRANE (PHOBIUS); NON_CYTOPLASMIC_DOMAIN (PHOBIUS); NON_CYTOPLASMIC_DOMAIN (PHOBIUS); TRANSMEMBRANE (PHOBIUS); TMhelix (TMHMM); TMhelix (TMHMM); TMhelix (TMHMM)	no GO terms	no GO terms
498	CP029088.1	Pf-tRNA-Met	I	hypothetical protein	111	20	1.24E-74	98.56						mobidb-lite (MOBIDB_LITE); mobidb-lite (MOBIDB_LITE); SIGNAL_PEPTIDE_H_REGION (PHOBIUS); SIGNAL_PEPTIDE_N_REGION (PHOBIUS); SIGNAL_PEPTIDE_C_REGION (PHOBIUS); NON_CYTOPLASMIC_DOMAIN (PHOBIUS); SIGNAL_PEPTIDE (PHOBIUS); SignalP-noTM (SIGNALP_EUK); SignalP-TM (SIGNALP_GRAM_POSITIVE); SignalP-noTM (SIGNALP_GRAM_NEGATIVE)	no GO terms	no GO terms
499	CP029088.1	Pf-tRNA-Met	I	Uncharacterised protein	363	20	0	72.55	1	C:GO:0005575	C:cellular_component			CYTOPLASMIC_DOMAIN (PHOBIUS); TRANSMEMBRANE (PHOBIUS); NON_CYTOPLASMIC_DOMAIN (PHOBIUS); CYTOPLASMIC_DOMAIN (PHOBIUS); TRANSMEMBRANE (PHOBIUS); TMhelix (TMHMM); TMhelix (TMHMM)	no GO terms	no GO terms
500	CP029088.1	Pf-tRNA-Met	I	hypothetical protein	130	20	2.19E-89	98.14						no IPS match	no IPS match	no IPS match
501	CP029090.1	Pf-tRNA-Gly	I	Fis family transcriptional regulator	369	20	0	88.35	2	F:GO:0003677; F:GO:0043167	F:DNA binding; F:ion binding			Coil (COILS); IPR003593 (SMART); G3DSA:3.40.50.300 (GENE3D); IPR003959 (PFAM); IPR025246 (PFAM); PTHR23073:SF29 (PANTHER); PTHR23073 (PANTHER); cd00009 (CDD); IPR027417 (SUPERFAMILY)	F:GO:0005524	F:ATP binding
502	CP029090.1	Pf-tRNA-Gly	I	Uncharacterised protein	74	20	3.18E-47	84.96						no IPS match	no IPS match	no IPS match
503	CP029090.1	Pf-tRNA-Gly	I	Uncharacterised protein	230	20	3.91E-173	96.98	1	C:GO:0005575	C:cellular_component			mobidb-lite (MOBIDB_LITE); mobidb-lite (MOBIDB_LITE); mobidb-lite (MOBIDB_LITE); TRANSMEMBRANE (PHOBIUS); NON_CYTOPLASMIC_DOMAIN (PHOBIUS); CYTOPLASMIC_DOMAIN (PHOBIUS); TMhelix (TMHMM)	no GO terms	no GO terms
504	CP029090.1	Pf-tRNA-Gly	I	Uncharacterised protein	165	20	5.14E-96	63.27	1	C:GO:0005575	C:cellular_component			CYTOPLASMIC_DOMAIN (PHOBIUS); NON_CYTOPLASMIC_DOMAIN (PHOBIUS); TRANSMEMBRANE (PHOBIUS)	no GO terms	no GO terms
505	CP029090.1	Pf-tRNA-Gly	I	S8 family peptidase	732	20	0	88.19	1	F:GO:0008233	F:peptidase activity	EC:3.4.21	Acting on peptide bonds (peptidases)	IPR036852 (G3DSA:3.40.50.GENE3D); IPR000209 (PFAM); IPR034074 (CDD); IPR036852 (SUPERFAMILY)	P:GO:0006508; F:GO:0004252	P:proteolysis; F:serine-type endopeptidase activity
506	CP029097.1	Pf-tRNA-Met	II	Cro/CI family transcriptional regulator	264	20	0	86.3	7	P:GO:0001666; P:GO:0019217; P:GO:0045892; P:GO:0045926; P:GO:0045927; F:GO:0003677	P:response to hypoxia; P:regulation of fatty acid metabolic process; P:negative regulation of transcription, DNA-templated; P:negative regulation of growth; P:positive regulation of growth; F:DNA binding			IPR007374 (SMART); IPR001387 (SMART); IPR001387 (PFAM); IPR007374 (PFAM); G3DSA:1.10.260.40 (GENE3D); G3DSA:2.30.130.30 (GENE3D); IPR001387 (PROSITE_PROFILES); IPR001387 (CDD); IPR015947 (SUPERFAMILY); IPR010982 (SUPERFAMILY)	F:GO:0003677	F:DNA binding
507	CP029097.1	Pf-tRNA-Met	II	GNAT family N-acetyltransferase	512	20	0	98.42	1	F:GO:0016746	F:transferase activity, transferring acyl groups	EC:2.3.1.5	Arylamine N-acetyltransferase	G3DSA:3.40.630.30 (GENE3D); IPR000182 (PFAM); IPR000182 (PROSITE_PROFILES); cd18699 (CDD); IPR016181 (SUPERFAMILY)	F:GO:0008080	F:N-acetyltransferase activity
508	CP029097.1	Pf-tRNA-Met	II	AAA family ATPase	175	20	2.76E-124	83.12				EC:2.7.4.3	Adenylate kinase	PF13207 (PFAM); G3DSA:3.40.50.300 (GENE3D); IPR027417 (SUPERFAMILY)	no GO terms	no GO terms
509	CP029097.1	Pf-tRNA-Met	II	MULTISPECIES: hypothetical protein	136	20	1.03E-93	98.01	1	C:GO:0005575	C:cellular_component			CYTOPLASMIC_DOMAIN (PHOBIUS); TRANSMEMBRANE (PHOBIUS); CYTOPLASMIC_DOMAIN (PHOBIUS); NON_CYTOPLASMIC_DOMAIN (PHOBIUS); NON_CYTOPLASMIC_DOMAIN (PHOBIUS); TRANSMEMBRANE (PHOBIUS); TRANSMEMBRANE (PHOBIUS); TMhelix (TMHMM); TMhelix (TMHMM); TMhelix (TMHMM)	no GO terms	no GO terms
510	CP029605.1c	Pf-tRNA-Gly	I	Uncharacterised protein	74	20	4.51E-47	86.4						no IPS match	no IPS match	no IPS match
511	CP029605.1c	Pf-tRNA-Gly	I	DUF262 domain-containing protein	347	20	0	82.13						IPR004919 (PFAM); PTHR39639 (PANTHER)	no GO terms	no GO terms
512	CP029605.1c	Pf-tRNA-Gly	I	Uncharacterised protein	252	20	0	66.65						IPR041519 (PFAM)	no GO terms	no GO terms
513	CP029605.1c	Pf-tRNA-Gly	I	Uncharacterised protein	234	14	4.94E-172	61.39						TRANSMEMBRANE (PHOBIUS); CYTOPLASMIC_DOMAIN (PHOBIUS); NON_CYTOPLASMIC_DOMAIN (PHOBIUS); TMhelix (TMHMM)	no GO terms	no GO terms
514	CP029660.1	Pf-tmRNA	I	Uncharacterised protein	232	20	3.07E-174	98.02	1	C:GO:0005575	C:cellular_component			mobidb-lite (MOBIDB_LITE); mobidb-lite (MOBIDB_LITE); mobidb-lite (MOBIDB_LITE); mobidb-lite (MOBIDB_LITE); mobidb-lite (MOBIDB_LITE); TRANSMEMBRANE (PHOBIUS); NON_CYTOPLASMIC_DOMAIN (PHOBIUS); CYTOPLASMIC_DOMAIN (PHOBIUS); TMhelix (TMHMM)	no GO terms	no GO terms
515	CP029660.1	Pf-tmRNA	I	Adenylylsulfate kinase	207	20	8.83E-121	56.42						no IPS match	no IPS match	no IPS match
516	CP029660.1	Pf-tmRNA	I	DUF91 domain-containing protein	234	20	1.12E-172	78.63	3	P:GO:0090305; F:GO:0003676; F:GO:0004519	P:nucleic acid phosphodiester bond hydrolysis; F:nucleic acid binding; F:endonuclease activity			IPR002793 (PFAM); IPR011856 (G3DSA:3.40.1350.GENE3D); IPR002793 (PANTHER)	F:GO:0003676; F:GO:0004518; F:GO:0004519	F:nucleic acid binding; F:nuclease activity; F:endonuclease activity
517	CP030327.1	Pf-tRNA-Gly	I	transposase	502	20	0	99.4	1	P:GO:0006259	P:DNA metabolic process			IPR001584 (PFAM); PTHR35004 (PANTHER); PTHR35004:SF6 (PANTHER); IPR017894 (PROSITE_PROFILES)	P:GO:0015074	P:DNA integration
518	CP030327.1	Pf-tRNA-Gly	I	AAA family ATPase	267	20	0	99.74	1	F:GO:0043167	F:ion binding			IPR003593 (SMART); IPR028350 (PIRSF); G3DSA:3.40.50.300 (GENE3D); IPR002611 (PFAM); mobidb-lite (MOBIDB_LITE); mobidb-lite (MOBIDB_LITE); PTHR30050 (PANTHER); PTHR30050:SF4 (PANTHER); cd00009 (CDD); IPR027417 (SUPERFAMILY)	F:GO:0005524	F:ATP binding
519	CP030328.1	Pf-tRNA-Gly	II	type II toxin-antitoxin system RelE/ParE family toxin	115	20	1.41E-76	98.87						IPR035093 (G3DSA:3.30.2310.GENE3D); IPR007712 (PFAM)	no GO terms	no GO terms
520	CP030328.1	Pf-tRNA-Gly	II	type II toxin-antitoxin system Phd/YefM family antitoxin	83	20	6.81E-53	96.37						PTHR33713:SF7 (PANTHER); PTHR33713 (PANTHER); IPR036165 (SUPERFAMILY)	no GO terms	no GO terms
521	CP030328.1	Pf-tRNA-Gly	II	Uncharacterised protein	232	20	1.83E-174	96.75	1	C:GO:0005575	C:cellular_component			mobidb-lite (MOBIDB_LITE); mobidb-lite (MOBIDB_LITE); mobidb-lite (MOBIDB_LITE); NON_CYTOPLASMIC_DOMAIN (PHOBIUS); TRANSMEMBRANE (PHOBIUS); CYTOPLASMIC_DOMAIN (PHOBIUS); TMhelix (TMHMM)	no GO terms	no GO terms
522	CP030328.1	Pf-tRNA-Gly	II	ATP-dependent endonuclease	586	20	0	88.94	2	F:GO:0004518; F:GO:0043167	F:nuclease activity; F:ion binding			Coil (COILS); G3DSA:3.40.50.300 (GENE3D); IPR041685 (PFAM); PTHR43581:SF1 (PANTHER); PTHR43581:SF1 (PANTHER); PTHR43581 (PANTHER); PTHR43581 (PANTHER); IPR034139 (CDD); cd00267 (CDD); IPR027417 (SUPERFAMILY)	no GO terms	no GO terms
523	CP030351.1	Pf-tRNA-Gly	II	Uncharacterised protein	74	20	4.51E-47	86.4						no IPS match	no IPS match	no IPS match
524	CP030351.1	Pf-tRNA-Gly	II	Uncharacterised protein	232	20	1.49E-174	96.85	1	C:GO:0005575	C:cellular_component			mobidb-lite (MOBIDB_LITE); mobidb-lite (MOBIDB_LITE); mobidb-lite (MOBIDB_LITE); TRANSMEMBRANE (PHOBIUS); NON_CYTOPLASMIC_DOMAIN (PHOBIUS); CYTOPLASMIC_DOMAIN (PHOBIUS); TMhelix (TMHMM)	no GO terms	no GO terms
525	CP030351.1	Pf-tRNA-Gly	II	ATP-dependent endonuclease	545	20	0	89.89	2	F:GO:0004518; F:GO:0043167	F:nuclease activity; F:ion binding			Coil (COILS); IPR041685 (PFAM); G3DSA:3.40.50.300 (GENE3D); PTHR43581:SF1 (PANTHER); PTHR43581 (PANTHER); PTHR43581:SF1 (PANTHER); PTHR43581 (PANTHER); IPR034139 (CDD); cd00267 (CDD); IPR027417 (SUPERFAMILY)	no GO terms	no GO terms
526	CP030910.1	Pf-tRNA-Met	II	MULTISPECIES: hypothetical protein	136	20	1.03E-93	98.01	1	C:GO:0005575	C:cellular_component			CYTOPLASMIC_DOMAIN (PHOBIUS); TRANSMEMBRANE (PHOBIUS); CYTOPLASMIC_DOMAIN (PHOBIUS); NON_CYTOPLASMIC_DOMAIN (PHOBIUS); NON_CYTOPLASMIC_DOMAIN (PHOBIUS); TRANSMEMBRANE (PHOBIUS); TRANSMEMBRANE (PHOBIUS); TMhelix (TMHMM); TMhelix (TMHMM); TMhelix (TMHMM)	no GO terms	no GO terms
527	CP030910.1	Pf-tRNA-Met	II	Cro/CI family transcriptional regulator	264	20	0	86.3	7	P:GO:0001666; P:GO:0019217; P:GO:0045892; P:GO:0045926; P:GO:0045927; F:GO:0003677	P:response to hypoxia; P:regulation of fatty acid metabolic process; P:negative regulation of transcription, DNA-templated; P:negative regulation of growth; P:positive regulation of growth; F:DNA binding			IPR001387 (SMART); IPR007374 (SMART); IPR007374 (PFAM); IPR001387 (PFAM); G3DSA:1.10.260.40 (GENE3D); G3DSA:2.30.130.30 (GENE3D); IPR001387 (PROSITE_PROFILES); IPR001387 (CDD); IPR015947 (SUPERFAMILY); IPR010982 (SUPERFAMILY)	F:GO:0003677	F:DNA binding
528	CP030910.1	Pf-tRNA-Met	II	GNAT family N-acetyltransferase	512	20	0	98.42	1	F:GO:0016746	F:transferase activity, transferring acyl groups	EC:2.3.1.5	Arylamine N-acetyltransferase	IPR000182 (PFAM); G3DSA:3.40.630.30 (GENE3D); IPR000182 (PROSITE_PROFILES); cd18699 (CDD); IPR016181 (SUPERFAMILY)	F:GO:0008080	F:N-acetyltransferase activity
529	CP030910.1	Pf-tRNA-Met	II	AAA family ATPase	175	20	2.76E-124	83.12				EC:2.7.4.3	Adenylate kinase	PF13207 (PFAM); G3DSA:3.40.50.300 (GENE3D); IPR027417 (SUPERFAMILY)	no GO terms	no GO terms
530	CP030910.1	Pf-DR	II	Uncharacterised protein	227	20	4.91E-161	77.55						no IPS match	no IPS match	no IPS match
531	CP030910.1b	Pf-tRNA-Gly	II	hypothetical protein	155	20	2.89E-104	76.22	1	C:GO:0005575	C:cellular_component			TRANSMEMBRANE (PHOBIUS); NON_CYTOPLASMIC_DOMAIN (PHOBIUS); TRANSMEMBRANE (PHOBIUS); TRANSMEMBRANE (PHOBIUS); CYTOPLASMIC_DOMAIN (PHOBIUS); CYTOPLASMIC_DOMAIN (PHOBIUS); CYTOPLASMIC_DOMAIN (PHOBIUS); TRANSMEMBRANE (PHOBIUS); NON_CYTOPLASMIC_DOMAIN (PHOBIUS); TMhelix (TMHMM); TMhelix (TMHMM); TMhelix (TMHMM); TMhelix (TMHMM)	no GO terms	no GO terms
532	CP030910.1b	Pf-tRNA-Gly	II	Uncharacterised protein	211	20	9.89E-156	73.29						no IPS match	no IPS match	no IPS match
533	CP030910.1b	Pf-tRNA-Gly	II	Uncharacterised protein	200	20	1.75E-145	70.47						no IPS match	no IPS match	no IPS match
534	CP030910.1b	Pf-tRNA-Gly	II	Uncharacterised protein	232	20	3.54E-174	96.47	1	C:GO:0005575	C:cellular_component			mobidb-lite (MOBIDB_LITE); mobidb-lite (MOBIDB_LITE); mobidb-lite (MOBIDB_LITE); NON_CYTOPLASMIC_DOMAIN (PHOBIUS); TRANSMEMBRANE (PHOBIUS); CYTOPLASMIC_DOMAIN (PHOBIUS); TMhelix (TMHMM)	no GO terms	no GO terms
535	CP030910.1b	Pf-tRNA-Gly	II	Uncharacterised protein	74	20	4.51E-47	86.4						no IPS match	no IPS match	no IPS match
536	CP030911.1	Pf-tRNA-Sec	II	Uncharacterised protein	75	20	6.64E-48	88.82						no IPS match	no IPS match	no IPS match
537	CP030911.1	Pf-tRNA-Sec	II	hypothetical protein	264	20	0	55.32	1	C:GO:0005575	C:cellular_component			NON_CYTOPLASMIC_DOMAIN (PHOBIUS); TRANSMEMBRANE (PHOBIUS); TRANSMEMBRANE (PHOBIUS); CYTOPLASMIC_DOMAIN (PHOBIUS); CYTOPLASMIC_DOMAIN (PHOBIUS); TRANSMEMBRANE (PHOBIUS); NON_CYTOPLASMIC_DOMAIN (PHOBIUS); TRANSMEMBRANE (PHOBIUS); NON_CYTOPLASMIC_DOMAIN (PHOBIUS); TRANSMEMBRANE (PHOBIUS); CYTOPLASMIC_DOMAIN (PHOBIUS); TMhelix (TMHMM); TMhelix (TMHMM); TMhelix (TMHMM); TMhelix (TMHMM); TMhelix (TMHMM)	no GO terms	no GO terms
538	CP030911.1	Pf-tRNA-Sec	II	hypothetical protein	133	20	1.39E-72	84.2	1	C:GO:0005575	C:cellular_component			mobidb-lite (MOBIDB_LITE); mobidb-lite (MOBIDB_LITE)	no GO terms	no GO terms
539	CP030911.1	Pf-tRNA-Sec	II	Uncharacterised protein	275	20	0	73.57	1	C:GO:0005575	C:cellular_component			TRANSMEMBRANE (PHOBIUS); TRANSMEMBRANE (PHOBIUS); CYTOPLASMIC_DOMAIN (PHOBIUS); CYTOPLASMIC_DOMAIN (PHOBIUS); NON_CYTOPLASMIC_DOMAIN (PHOBIUS); TMhelix (TMHMM); TMhelix (TMHMM)	no GO terms	no GO terms
540	CP031660.1	Pf-DR	II	DUF262 domain-containing protein	599	20	0	81.15						IPR004919 (PFAM); IPR038461 (G3DSA:3.30.950.GENE3D); PTHR39639 (PANTHER)	no GO terms	no GO terms
541	CP032126.1	Pf-tRNA-Gly	I	RNA-directed DNA polymerase	283	20	0	80.44	4	P:GO:0006259; P:GO:0009058; F:GO:0003723; F:GO:0016779	P:DNA metabolic process; P:biosynthetic process; F:RNA binding; F:nucleotidyltransferase activity	EC:2.7.7.49	RNA-directed DNA polymerase	IPR000123 (PRINTS); IPR000477 (PFAM); PTHR34047:SF6 (PANTHER); PTHR34047 (PANTHER); IPR000477 (PROSITE_PROFILES); cd03487 (CDD); SSF56672 (SUPERFAMILY)	P:GO:0006278; F:GO:0003723; F:GO:0003964	P:RNA-dependent DNA biosynthetic process; F:RNA binding; F:RNA-directed DNA polymerase activity
542	CP032126.1	Pf-tRNA-Gly	I	AAA family ATPase	441	20	0	78.07	1	F:GO:0043167	F:ion binding			IPR003593 (SMART); G3DSA:3.40.50.300 (GENE3D); IPR038729 (PFAM); IPR003959 (PFAM); PTHR43581:SF2 (PANTHER); PTHR43581 (PANTHER); IPR027417 (SUPERFAMILY)	F:GO:0005524	F:ATP binding
543	CP032126.1	Pf-tRNA-Gly	I	type II toxin-antitoxin system RelE/ParE family toxin	115	20	2.09E-76	98.57						IPR007712 (PFAM); IPR035093 (G3DSA:3.30.2310.GENE3D)	no GO terms	no GO terms
544	CP032126.1	Pf-tRNA-Gly	I	type II toxin-antitoxin system Phd/YefM family antitoxin	83	20	6.81E-53	96.37						PTHR33713:SF7 (PANTHER); PTHR33713 (PANTHER); IPR036165 (SUPERFAMILY)	no GO terms	no GO terms
545	CP032126.1	Pf-tRNA-Met	I	serine/threonine-protein phosphatase	365	20	0	63.24	2	P:GO:0006470; F:GO:0004722	P:protein dephosphorylation; F:protein serine/threonine phosphatase activity	EC:3.1.3.16	Protein-serine/threonine phosphatase	IPR001932 (SMART); IPR036457 (G3DSA:3.60.40.GENE3D); PF13672 (PFAM); mobidb-lite (MOBIDB_LITE); mobidb-lite (MOBIDB_LITE); mobidb-lite (MOBIDB_LITE); IPR015655 (PANTHER); PTHR13832:SF278 (PANTHER); IPR001932 (PROSITE_PROFILES); IPR001932 (CDD); IPR036457 (SUPERFAMILY)	P:GO:0006470; F:GO:0003824; F:GO:0004722	P:protein dephosphorylation; F:catalytic activity; F:protein serine/threonine phosphatase activity
546	CP032126.1	Pf-tRNA-Met	I	serine/threonine protein kinase	349	20	0	69.97	3	P:GO:0006468; F:GO:0004674; F:GO:0005524	P:protein phosphorylation; F:protein serine/threonine kinase activity; F:ATP binding	EC:2.7.11	Transferring phosphorus-containing groups	IPR000719 (SMART); G3DSA:1.10.510.10 (GENE3D); IPR000719 (PFAM); PTHR24346 (PANTHER); IPR008271 (PROSITE_PATTERNS); IPR000719 (PROSITE_PROFILES); cd14014 (CDD); IPR011009 (SUPERFAMILY)	P:GO:0006468; F:GO:0004672; F:GO:0005524	P:protein phosphorylation; F:protein kinase activity; F:ATP binding
547	CP032126.1	Pf-tRNA-Met	I	serine/threonine protein kinase	357	20	0	78.68	3	P:GO:0006464; F:GO:0016301; F:GO:0043167	P:cellular protein modification process; F:kinase activity; F:ion binding			IPR000719 (SMART); IPR000719 (PFAM); G3DSA:1.10.510.10 (GENE3D); PIRSF000654 (PIRSF); G3DSA:3.30.200.20 (GENE3D); PTHR43289:SF11 (PANTHER); PTHR43289 (PANTHER); IPR008271 (PROSITE_PATTERNS); IPR000719 (PROSITE_PROFILES); cd14014 (CDD); IPR012340 (SUPERFAMILY); IPR011009 (SUPERFAMILY)	P:GO:0006468; F:GO:0004672; F:GO:0005524	P:protein phosphorylation; F:protein kinase activity; F:ATP binding
548	CP032552.1	Pf-tmRNA	I	hypothetical protein	133	20	1.39E-73	85.62	1	C:GO:0005575	C:cellular_component			Coil (COILS); mobidb-lite (MOBIDB_LITE); mobidb-lite (MOBIDB_LITE)	no GO terms	no GO terms
549	CP032552.1	Pf-tmRNA	I	hypothetical protein	372	20	0	73.82						no IPS match	no IPS match	no IPS match
550	CP032569.1	Pf-tRNA-Gly	I	type II toxin-antitoxin system RelE/ParE family toxin	76	20	5.15E-47	99.28						IPR007712 (PFAM)	no GO terms	no GO terms
551	CP032569.1	Pf-tRNA-Gly	I	type II toxin-antitoxin system Phd/YefM family antitoxin	83	20	6.81E-53	96.37						PTHR33713:SF7 (PANTHER); PTHR33713 (PANTHER); IPR036165 (SUPERFAMILY)	no GO terms	no GO terms
552	CP032569.1	Pf-tRNA-Gly	I	Uncharacterised protein	171	20	9.01E-122	66.88	1	C:GO:0005575	C:cellular_component			SIGNAL_PEPTIDE_H_REGION (PHOBIUS); NON_CYTOPLASMIC_DOMAIN (PHOBIUS); SIGNAL_PEPTIDE_C_REGION (PHOBIUS); SIGNAL_PEPTIDE_N_REGION (PHOBIUS); SIGNAL_PEPTIDE (PHOBIUS)	no GO terms	no GO terms
553	CP032569.1	Pf-tRNA-Gly	I	Uncharacterised protein	256	20	0	85.4	1	C:GO:0005575	C:cellular_component			mobidb-lite (MOBIDB_LITE); mobidb-lite (MOBIDB_LITE); CYTOPLASMIC_DOMAIN (PHOBIUS); NON_CYTOPLASMIC_DOMAIN (PHOBIUS); TRANSMEMBRANE (PHOBIUS); TMhelix (TMHMM)	no GO terms	no GO terms
554	CP032569.1	Pf-tRNA-Gly	I	Uncharacterised protein	94	3	2.60E-63	68.62						no IPS match	no IPS match	no IPS match
555	CP033432.1	Pf-tRNA-Gly	I	type II toxin-antitoxin system RelE/ParE family toxin	115	20	1.41E-76	98.87						IPR035093 (G3DSA:3.30.2310.GENE3D); IPR007712 (PFAM)	no GO terms	no GO terms
556	CP033432.1	Pf-tRNA-Gly	I	type II toxin-antitoxin system Phd/YefM family antitoxin	83	20	6.81E-53	96.37						PTHR33713:SF7 (PANTHER); PTHR33713 (PANTHER); IPR036165 (SUPERFAMILY)	no GO terms	no GO terms
557	CP033432.1	Pf-tRNA-Gly	I	Uncharacterised protein	94	3	2.60E-63	68.62						no IPS match	no IPS match	no IPS match
558	CP033432.1	Pf-tRNA-Gly	I	Uncharacterised protein	256	20	0	85.4	1	C:GO:0005575	C:cellular_component			mobidb-lite (MOBIDB_LITE); mobidb-lite (MOBIDB_LITE); CYTOPLASMIC_DOMAIN (PHOBIUS); NON_CYTOPLASMIC_DOMAIN (PHOBIUS); TRANSMEMBRANE (PHOBIUS); TMhelix (TMHMM)	no GO terms	no GO terms
559	CP033432.1	Pf-tRNA-Gly	I	Uncharacterised protein	242	20	2.44E-177	65.05	1	C:GO:0005575	C:cellular_component			Coil (COILS); Coil (COILS); SIGNAL_PEPTIDE_H_REGION (PHOBIUS); SIGNAL_PEPTIDE_C_REGION (PHOBIUS); SIGNAL_PEPTIDE_N_REGION (PHOBIUS); CYTOPLASMIC_DOMAIN (PHOBIUS); TRANSMEMBRANE (PHOBIUS); NON_CYTOPLASMIC_DOMAIN (PHOBIUS); SIGNAL_PEPTIDE (PHOBIUS); TMhelix (TMHMM); TMhelix (TMHMM)	no GO terms	no GO terms
560	CP033432.1	Pf-tRNA-Gly	I	Uncharacterised protein	265	20	0	66.81						no IPS match	no IPS match	no IPS match
561	CP033439.1	Pf-tRNA-Gly	I	Uncharacterised protein	265	20	0	65.13	1	C:GO:0005575	C:cellular_component			TRANSMEMBRANE (PHOBIUS); CYTOPLASMIC_DOMAIN (PHOBIUS); NON_CYTOPLASMIC_DOMAIN (PHOBIUS); CYTOPLASMIC_DOMAIN (PHOBIUS); TRANSMEMBRANE (PHOBIUS); TRANSMEMBRANE (PHOBIUS); NON_CYTOPLASMIC_DOMAIN (PHOBIUS); TMhelix (TMHMM); TMhelix (TMHMM); TMhelix (TMHMM)	no GO terms	no GO terms
562	CP033439.1	Pf-tRNA-Gly	I	type II toxin-antitoxin system Phd/YefM family antitoxin	83	20	6.81E-53	96.37						PTHR33713:SF7 (PANTHER); PTHR33713 (PANTHER); IPR036165 (SUPERFAMILY)	no GO terms	no GO terms
563	CP033439.1	Pf-tRNA-Gly	I	type II toxin-antitoxin system RelE/ParE family toxin	115	20	1.41E-76	98.87						IPR035093 (G3DSA:3.30.2310.GENE3D); IPR007712 (PFAM)	no GO terms	no GO terms
564	CP033684.1	Pf-DR	II	ATPase	413	20	0	91.07	1	F:GO:0043167	F:ion binding			IPR041685 (PFAM); G3DSA:3.40.50.300 (GENE3D); G3DSA:3.40.50.300 (GENE3D); IPR014555 (PIRSF); IPR003959 (PFAM); PTHR40396 (PANTHER); IPR027417 (SUPERFAMILY)	F:GO:0005524	F:ATP binding
565	CP033684.1	Pf-DR	II	DUF4276 family protein	200	20	4.62E-145	84.59						IPR025455 (PFAM)	no GO terms	no GO terms
566	CP033685.1	Pf-tRNA-Met	I	Uncharacterised protein	145	20	3.37E-100	93.45	1	C:GO:0005575	C:cellular_component			Coil (COILS); TRANSMEMBRANE (PHOBIUS); CYTOPLASMIC_DOMAIN (PHOBIUS); TRANSMEMBRANE (PHOBIUS); CYTOPLASMIC_DOMAIN (PHOBIUS); NON_CYTOPLASMIC_DOMAIN (PHOBIUS); TMhelix (TMHMM); TMhelix (TMHMM)	no GO terms	no GO terms
567	CP033685.1	Pf-tRNA-Met	I	sce7726 family protein	193	20	4.42E-138	76.55						no IPS match	no IPS match	no IPS match
568	CP033685.1	Pf-tRNA-Met	I	Uncharacterised protein	356	20	0	87.11						IPR025683 (PFAM)	no GO terms	no GO terms
569	CP033685.1	Pf-tRNA-Met	I	ImmA/IrrE family metallo-endopeptidase	278	20	0	89.91						IPR010359 (PFAM)	no GO terms	no GO terms
570	CP033685.1	Pf-tRNA-Met	I	N-acetyltransferase	224	20	7.43E-168	70.71	1	F:GO:0003674	F:molecular_function			IPR016181 (SUPERFAMILY)	no GO terms	no GO terms
571	CP033685.1	Pf-tRNA-Met	I	MULTISPECIES: hypothetical protein	135	20	1.99E-94	98.03						no IPS match	no IPS match	no IPS match
572	CP033685.1	Pf-DR	I	Uncharacterised protein	202	20	1.41E-145	74.69						IPR041519 (PFAM)	no GO terms	no GO terms
573	CP033685.1	Pf-DR	I	ParA family protein	343	20	0	82.15	4	P:GO:0071941; F:GO:0016491; C:GO:0005622; C:GO:0032991	P:nitrogen cycle metabolic process; F:oxidoreductase activity; C:intracellular; C:protein-containing complex	EC:1.18.6.1; EC:1.18.6	Nitrogenase; Acting on iron-sulfur proteins as donors	Coil (COILS); IPR025669 (PFAM); G3DSA:3.40.50.300 (GENE3D); PTHR13696:SF52 (PANTHER); PTHR13696 (PANTHER); cd02042 (CDD); IPR027417 (SUPERFAMILY)	no GO terms	no GO terms
574	CP033686.1	Pf-tRNA-Gly	II	Uncharacterised protein	140	20	3.92E-100	57.2						no IPS match	no IPS match	no IPS match
575	CP033686.1	Pf-tRNA-Gly	II	MULTISPECIES: hypothetical protein	232	20	1.39E-174	95.54	1	C:GO:0005575	C:cellular_component			mobidb-lite (MOBIDB_LITE); mobidb-lite (MOBIDB_LITE); TRANSMEMBRANE (PHOBIUS); NON_CYTOPLASMIC_DOMAIN (PHOBIUS); CYTOPLASMIC_DOMAIN (PHOBIUS); TMhelix (TMHMM)	no GO terms	no GO terms
576	CP033686.1	Pf-tRNA-Gly	II	Uncharacterised protein	360	20	0	67.47						no IPS match	no IPS match	no IPS match
577	CP033686.1	Pf-tRNA-Met	I	Uncharacterised protein	145	20	6.89E-100	95.34	1	C:GO:0005575	C:cellular_component			TRANSMEMBRANE (PHOBIUS); CYTOPLASMIC_DOMAIN (PHOBIUS); TRANSMEMBRANE (PHOBIUS); CYTOPLASMIC_DOMAIN (PHOBIUS); NON_CYTOPLASMIC_DOMAIN (PHOBIUS); TMhelix (TMHMM); TMhelix (TMHMM)	no GO terms	no GO terms
578	CP033686.1	Pf-tRNA-Met	I	ATP-dependent DNA helicase RecG	628	20	0	83.91	2	F:GO:0003674	F:molecular_function	EC:3.6.4.12	DNA helicase	IPR000504 (SMART); IPR007421 (PFAM); IPR038475 (G3DSA:3.30.565.GENE3D); IPR000504 (PFAM); IPR025831 (PFAM); IPR012677 (G3DSA:3.30.70.GENE3D); IPR038461 (G3DSA:3.30.950.GENE3D); PTHR30595 (PANTHER); PTHR30595:SF6 (PANTHER); IPR000504 (PROSITE_PROFILES); IPR035979 (SUPERFAMILY)	F:GO:0003676	F:nucleic acid binding
579	CP033686.1	Pf-tRNA-Met	I	hypothetical protein	48	20	1.03E-18	93.39						no IPS match	no IPS match	no IPS match
580	CP033686.1	Pf-tRNA-Met	I	Uncharacterised protein	110	20	2.86E-74	89.86						SIGNAL_PEPTIDE_H_REGION (PHOBIUS); NON_CYTOPLASMIC_DOMAIN (PHOBIUS); SIGNAL_PEPTIDE_N_REGION (PHOBIUS); SIGNAL_PEPTIDE_C_REGION (PHOBIUS); SIGNAL_PEPTIDE (PHOBIUS); SignalP-TM (SIGNALP_GRAM_POSITIVE); SignalP-noTM (SIGNALP_GRAM_NEGATIVE); SignalP-noTM (SIGNALP_EUK)	no GO terms	no GO terms
581	CP033686.1	Pf-tRNA-Met	I	MULTISPECIES: hypothetical protein	270	20	0	76.01						mobidb-lite (MOBIDB_LITE)	no GO terms	no GO terms
582	CP033771.1	Pf-tRNA-Met	II	MULTISPECIES: hypothetical protein	136	20	1.03E-93	98.01	1	C:GO:0005575	C:cellular_component			CYTOPLASMIC_DOMAIN (PHOBIUS); TRANSMEMBRANE (PHOBIUS); CYTOPLASMIC_DOMAIN (PHOBIUS); NON_CYTOPLASMIC_DOMAIN (PHOBIUS); NON_CYTOPLASMIC_DOMAIN (PHOBIUS); TRANSMEMBRANE (PHOBIUS); TRANSMEMBRANE (PHOBIUS); TMhelix (TMHMM); TMhelix (TMHMM); TMhelix (TMHMM)	no GO terms	no GO terms
583	CP033771.1	Pf-tRNA-Met	II	Cro/CI family transcriptional regulator	264	20	0	86.3	7	P:GO:0001666; P:GO:0019217; P:GO:0045892; P:GO:0045926; P:GO:0045927; F:GO:0003677	P:response to hypoxia; P:regulation of fatty acid metabolic process; P:negative regulation of transcription, DNA-templated; P:negative regulation of growth; P:positive regulation of growth; F:DNA binding			IPR001387 (SMART); IPR007374 (SMART); IPR007374 (PFAM); IPR001387 (PFAM); G3DSA:1.10.260.40 (GENE3D); G3DSA:2.30.130.30 (GENE3D); IPR001387 (PROSITE_PROFILES); IPR001387 (CDD); IPR015947 (SUPERFAMILY); IPR010982 (SUPERFAMILY)	F:GO:0003677	F:DNA binding
584	CP033771.1	Pf-tRNA-Met	II	hypothetical protein CDO36_28305, partial	34	3	8.14E-17	100						no IPS match	no IPS match	no IPS match
585	CP033771.1	Pf-tRNA-Met	II	GNAT family N-acetyltransferase	177	20	1.74E-129	99.33	1	F:GO:0016746	F:transferase activity, transferring acyl groups	EC:2.3.1.5	Arylamine N-acetyltransferase	no IPS match	no IPS match	no IPS match
586	CP033771.1	Pf-tRNA-Met	II	AAA family ATPase	175	20	2.76E-124	83.12				EC:2.7.4.3	Adenylate kinase	PF13207 (PFAM); G3DSA:3.40.50.300 (GENE3D); IPR027417 (SUPERFAMILY)	no GO terms	no GO terms
587	CP033771.1	Pf-tRNA-Met	II	IS30 family transposase IS1394	338	20	0	91.47	4	P:GO:0006313; P:GO:0015074; F:GO:0003677; F:GO:0004803	P:transposition, DNA-mediated; P:DNA integration; F:DNA binding; F:transposase activity			IPR025246 (PFAM); IPR036397 (G3DSA:3.30.420.GENE3D); IPR001584 (PFAM); PTHR10948 (PANTHER); IPR001598 (PROSITE_PATTERNS); IPR001584 (PROSITE_PROFILES); IPR012337 (SUPERFAMILY)	P:GO:0006313; P:GO:0015074; F:GO:0003676; F:GO:0003677; F:GO:0004803	P:transposition, DNA-mediated; P:DNA integration; F:nucleic acid binding; F:DNA binding; F:transposase activity
588	CP033832.1	Pf-tRNA-Met	II	MULTISPECIES: hypothetical protein	136	20	1.03E-93	98.01	1	C:GO:0005575	C:cellular_component			CYTOPLASMIC_DOMAIN (PHOBIUS); TRANSMEMBRANE (PHOBIUS); CYTOPLASMIC_DOMAIN (PHOBIUS); NON_CYTOPLASMIC_DOMAIN (PHOBIUS); NON_CYTOPLASMIC_DOMAIN (PHOBIUS); TRANSMEMBRANE (PHOBIUS); TRANSMEMBRANE (PHOBIUS); TMhelix (TMHMM); TMhelix (TMHMM); TMhelix (TMHMM)	no GO terms	no GO terms
589	CP033832.1	Pf-tRNA-Met	II	Cro/CI family transcriptional regulator	264	20	0	86.3	7	P:GO:0001666; P:GO:0019217; P:GO:0045892; P:GO:0045926; P:GO:0045927; F:GO:0003677	P:response to hypoxia; P:regulation of fatty acid metabolic process; P:negative regulation of transcription, DNA-templated; P:negative regulation of growth; P:positive regulation of growth; F:DNA binding			IPR001387 (SMART); IPR007374 (SMART); IPR007374 (PFAM); IPR001387 (PFAM); G3DSA:1.10.260.40 (GENE3D); G3DSA:2.30.130.30 (GENE3D); IPR001387 (PROSITE_PROFILES); IPR001387 (CDD); IPR015947 (SUPERFAMILY); IPR010982 (SUPERFAMILY)	F:GO:0003677	F:DNA binding
590	CP033832.1	Pf-tRNA-Met	II	GNAT family N-acetyltransferase	512	20	0	98.42	1	F:GO:0016746	F:transferase activity, transferring acyl groups	EC:2.3.1.5	Arylamine N-acetyltransferase	G3DSA:3.40.630.30 (GENE3D); IPR000182 (PFAM); IPR000182 (PROSITE_PROFILES); cd18699 (CDD); IPR016181 (SUPERFAMILY)	F:GO:0008080	F:N-acetyltransferase activity
591	CP033832.1	Pf-tRNA-Met	II	AAA family ATPase	175	20	2.76E-124	83.12				EC:2.7.4.3	Adenylate kinase	PF13207 (PFAM); G3DSA:3.40.50.300 (GENE3D); IPR027417 (SUPERFAMILY)	no GO terms	no GO terms
592	CP033835.1a	Pf-tRNA-Gly	I	hypothetical protein	230	20	2.64E-165	55.33	1	C:GO:0005575	C:cellular_component			TRANSMEMBRANE (PHOBIUS); CYTOPLASMIC_DOMAIN (PHOBIUS); CYTOPLASMIC_DOMAIN (PHOBIUS); NON_CYTOPLASMIC_DOMAIN (PHOBIUS); TRANSMEMBRANE (PHOBIUS); TMhelix (TMHMM); TMhelix (TMHMM)	no GO terms	no GO terms
593	CP034354.1	Pf-tRNA-Gly	I	type II toxin-antitoxin system RelE/ParE family toxin	115	20	2.09E-76	98.57						IPR007712 (PFAM); IPR035093 (G3DSA:3.30.2310.GENE3D)	no GO terms	no GO terms
594	CP034354.1	Pf-tRNA-Gly	I	type II toxin-antitoxin system Phd/YefM family antitoxin	83	20	6.81E-53	96.37						PTHR33713:SF7 (PANTHER); PTHR33713 (PANTHER); IPR036165 (SUPERFAMILY)	no GO terms	no GO terms
595	CP034354.1	Pf-tRNA-Gly	I	hypothetical protein	232	20	3.78E-174	93.06	1	C:GO:0005575	C:cellular_component			mobidb-lite (MOBIDB_LITE); mobidb-lite (MOBIDB_LITE); mobidb-lite (MOBIDB_LITE); mobidb-lite (MOBIDB_LITE); TRANSMEMBRANE (PHOBIUS); CYTOPLASMIC_DOMAIN (PHOBIUS); NON_CYTOPLASMIC_DOMAIN (PHOBIUS); TMhelix (TMHMM)	no GO terms	no GO terms
596	CP034354.1	Pf-tRNA-Gly	I	DUF3365 domain-containing protein	84	18	5.44E-56	82.27	3	P:GO:0008150; F:GO:0003674; C:GO:0005575	P:biological_process; F:molecular_function; C:cellular_component			no IPS match	no IPS match	no IPS match
597	CP034354.1	Pf-tRNA-Gly	I	hypothetical protein	172	11	2.12E-119	74.96	1	C:GO:0005575	C:cellular_component			TRANSMEMBRANE (PHOBIUS); NON_CYTOPLASMIC_DOMAIN (PHOBIUS); CYTOPLASMIC_DOMAIN (PHOBIUS); TMhelix (TMHMM)	no GO terms	no GO terms
598	CP034354.1	Pf-tRNA-Gly	I	hypothetical protein	266	20	0	71.61						TRANSMEMBRANE (PHOBIUS); NON_CYTOPLASMIC_DOMAIN (PHOBIUS); CYTOPLASMIC_DOMAIN (PHOBIUS)	no GO terms	no GO terms
599	CP034354.1	Pf-tRNA-Gly	I	ATP-binding protein	479	20	0	59.1	2	F:GO:0043167	F:ion binding	EC:3.6.4.12	DNA helicase	IPR038461 (G3DSA:3.30.950.GENE3D); IPR007421 (PFAM); PTHR30595:SF6 (PANTHER); PTHR30595 (PANTHER); NON_CYTOPLASMIC_DOMAIN (PHOBIUS); TRANSMEMBRANE (PHOBIUS); CYTOPLASMIC_DOMAIN (PHOBIUS); TMhelix (TMHMM)	no GO terms	no GO terms
600	CP034368.1	Pf-tRNA-Gly	I	type II toxin-antitoxin system RelE/ParE family toxin	115	20	1.41E-76	98.87						IPR035093 (G3DSA:3.30.2310.GENE3D); IPR007712 (PFAM)	no GO terms	no GO terms
601	CP034368.1	Pf-tRNA-Gly	I	type II toxin-antitoxin system Phd/YefM family antitoxin	83	20	6.81E-53	96.37						PTHR33713:SF7 (PANTHER); PTHR33713 (PANTHER); IPR036165 (SUPERFAMILY)	no GO terms	no GO terms
602	CP034368.1	Pf-tRNA-Gly	I	Uncharacterised protein	209	20	6.03E-147	64.66	1	C:GO:0005575	C:cellular_component			NON_CYTOPLASMIC_DOMAIN (PHOBIUS); TRANSMEMBRANE (PHOBIUS); NON_CYTOPLASMIC_DOMAIN (PHOBIUS); TRANSMEMBRANE (PHOBIUS); CYTOPLASMIC_DOMAIN (PHOBIUS); TRANSMEMBRANE (PHOBIUS); NON_CYTOPLASMIC_DOMAIN (PHOBIUS); CYTOPLASMIC_DOMAIN (PHOBIUS); TMhelix (TMHMM); TMhelix (TMHMM); TMhelix (TMHMM)	no GO terms	no GO terms
603	CP034368.1	Pf-tRNA-Gly	I	Uncharacterised protein	66	20	1.65E-33	66.89	1	C:GO:0005575	C:cellular_component			no IPS match	no IPS match	no IPS match
604	CP034369.1	Pf-tRNA-Gly	I	type II toxin-antitoxin system RelE/ParE family toxin	115	20	1.41E-76	98.87						IPR035093 (G3DSA:3.30.2310.GENE3D); IPR007712 (PFAM)	no GO terms	no GO terms
605	CP034369.1	Pf-tRNA-Gly	I	type II toxin-antitoxin system Phd/YefM family antitoxin	83	20	6.81E-53	96.37						PTHR33713:SF7 (PANTHER); PTHR33713 (PANTHER); IPR036165 (SUPERFAMILY)	no GO terms	no GO terms
606	CP034369.1	Pf-tRNA-Gly	I	Uncharacterised protein	265	20	0	65.13	1	C:GO:0005575	C:cellular_component			TRANSMEMBRANE (PHOBIUS); CYTOPLASMIC_DOMAIN (PHOBIUS); NON_CYTOPLASMIC_DOMAIN (PHOBIUS); CYTOPLASMIC_DOMAIN (PHOBIUS); TRANSMEMBRANE (PHOBIUS); TRANSMEMBRANE (PHOBIUS); NON_CYTOPLASMIC_DOMAIN (PHOBIUS); TMhelix (TMHMM); TMhelix (TMHMM); TMhelix (TMHMM)	no GO terms	no GO terms
607	CP034409.1	Pf-tRNA-Gly	I	type II toxin-antitoxin system RelE/ParE family toxin	115	20	1.41E-76	98.87						IPR035093 (G3DSA:3.30.2310.GENE3D); IPR007712 (PFAM)	no GO terms	no GO terms
608	CP034409.1	Pf-tRNA-Gly	I	type II toxin-antitoxin system Phd/YefM family antitoxin	83	20	6.81E-53	96.37						PTHR33713:SF7 (PANTHER); PTHR33713 (PANTHER); IPR036165 (SUPERFAMILY)	no GO terms	no GO terms
609	CP034409.1	Pf-tRNA-Gly	I	Uncharacterised protein	103	20	1.65E-68	67.68	1	C:GO:0005575	C:cellular_component			no IPS match	no IPS match	no IPS match
610	CP034429.1	Pf-tRNA-Gly	I	RNA-directed DNA polymerase	283	20	0	80.44	4	P:GO:0006259; P:GO:0009058; F:GO:0003723; F:GO:0016779	P:DNA metabolic process; P:biosynthetic process; F:RNA binding; F:nucleotidyltransferase activity	EC:2.7.7.49	RNA-directed DNA polymerase	IPR000123 (PRINTS); IPR000477 (PFAM); PTHR34047:SF6 (PANTHER); PTHR34047 (PANTHER); IPR000477 (PROSITE_PROFILES); cd03487 (CDD); SSF56672 (SUPERFAMILY)	P:GO:0006278; F:GO:0003723; F:GO:0003964	P:RNA-dependent DNA biosynthetic process; F:RNA binding; F:RNA-directed DNA polymerase activity
611	CP034429.1	Pf-tRNA-Gly	I	AAA family ATPase	441	20	0	78.07	1	F:GO:0043167	F:ion binding			IPR003593 (SMART); G3DSA:3.40.50.300 (GENE3D); IPR038729 (PFAM); IPR003959 (PFAM); PTHR43581:SF2 (PANTHER); PTHR43581 (PANTHER); IPR027417 (SUPERFAMILY)	F:GO:0005524	F:ATP binding
612	CP034429.1	Pf-tRNA-Gly	I	type II toxin-antitoxin system RelE/ParE family toxin	115	20	2.09E-76	98.57						IPR007712 (PFAM); IPR035093 (G3DSA:3.30.2310.GENE3D)	no GO terms	no GO terms
613	CP034429.1	Pf-tRNA-Gly	I	type II toxin-antitoxin system Phd/YefM family antitoxin	83	20	6.81E-53	96.37						PTHR33713:SF7 (PANTHER); PTHR33713 (PANTHER); IPR036165 (SUPERFAMILY)	no GO terms	no GO terms
614	CP034429.1	Pf-tRNA-Met	I	serine/threonine-protein phosphatase	368	20	0	63.94	2	P:GO:0006470; F:GO:0004722	P:protein dephosphorylation; F:protein serine/threonine phosphatase activity	EC:3.1.3.16	Protein-serine/threonine phosphatase	IPR001932 (SMART); PF13672 (PFAM); IPR036457 (G3DSA:3.60.40.GENE3D); mobidb-lite (MOBIDB_LITE); mobidb-lite (MOBIDB_LITE); mobidb-lite (MOBIDB_LITE); mobidb-lite (MOBIDB_LITE); IPR015655 (PANTHER); PTHR13832:SF278 (PANTHER); IPR001932 (PROSITE_PROFILES); IPR001932 (CDD); IPR036457 (SUPERFAMILY)	P:GO:0006470; F:GO:0003824; F:GO:0004722	P:protein dephosphorylation; F:catalytic activity; F:protein serine/threonine phosphatase activity
615	CP034429.1	Pf-tRNA-Met	I	serine/threonine protein kinase	349	20	0	69.97	3	P:GO:0006468; F:GO:0004674; F:GO:0005524	P:protein phosphorylation; F:protein serine/threonine kinase activity; F:ATP binding	EC:2.7.11	Transferring phosphorus-containing groups	IPR000719 (SMART); G3DSA:1.10.510.10 (GENE3D); IPR000719 (PFAM); PTHR24346 (PANTHER); IPR008271 (PROSITE_PATTERNS); IPR000719 (PROSITE_PROFILES); cd14014 (CDD); IPR011009 (SUPERFAMILY)	P:GO:0006468; F:GO:0004672; F:GO:0005524	P:protein phosphorylation; F:protein kinase activity; F:ATP binding

616	CP034429.1	Pf-tRNA-Met	I	serine/threonine protein kinase	357	20	0	78.68	3	P:GO:0006464; F:GO:0016301; F:GO:0043167	P:cellular protein modification process; F:kinase activity; F:ion binding			IPR000719 (SMART); IPR000719 (PFAM); G3DSA:1.10.510.10 (GENE3D); PIRSF000654 (PIRSF); G3DSA:3.30.200.20 (GENE3D); PTHR43289:SF11 (PANTHER); PTHR43289 (PANTHER); IPR008271 (PROSITE_PATTERNS); IPR000719 (PROSITE_PROFILES); cd14014 (CDD); IPR012340 (SUPERFAMILY); IPR011009 (SUPERFAMILY)	P:GO:0006468; F:GO:0004672; F:GO:0005524	P:protein phosphorylation; F:protein kinase activity; F:ATP binding
617	CP034434.1	Pf-tRNA-Gly	I	type II toxin-antitoxin system RelE/ParE family toxin	115	20	1.41E-76	98.87						IPR035093 (G3DSA:3.30.2310.GENE3D); IPR007712 (PFAM)	no GO terms	no GO terms
618	CP034434.1	Pf-tRNA-Gly	I	type II toxin-antitoxin system Phd/YefM family antitoxin	83	20	6.81E-53	96.37						PTHR33713:SF7 (PANTHER); PTHR33713 (PANTHER); IPR036165 (SUPERFAMILY)	no GO terms	no GO terms
619	CP034434.1	Pf-tRNA-Gly	I	Uncharacterised protein	265	20	0	66.81						no IPS match	no IPS match	no IPS match
620	CP034434.1	Pf-tRNA-Gly	I	Uncharacterised protein	242	20	1.94E-177	65.51	1	C:GO:0005575	C:cellular_component			Coil (COILS); Coil (COILS); SIGNAL_PEPTIDE_H_REGION (PHOBIUS); SIGNAL_PEPTIDE_C_REGION (PHOBIUS); SIGNAL_PEPTIDE_N_REGION (PHOBIUS); CYTOPLASMIC_DOMAIN (PHOBIUS); TRANSMEMBRANE (PHOBIUS); NON_CYTOPLASMIC_DOMAIN (PHOBIUS); SIGNAL_PEPTIDE (PHOBIUS); TMhelix (TMHMM); TMhelix (TMHMM)	no GO terms	no GO terms
621	CP034434.1	Pf-tRNA-Gly	I	Uncharacterised protein	256	20	0	85.4	1	C:GO:0005575	C:cellular_component			mobidb-lite (MOBIDB_LITE); mobidb-lite (MOBIDB_LITE); CYTOPLASMIC_DOMAIN (PHOBIUS); NON_CYTOPLASMIC_DOMAIN (PHOBIUS); TRANSMEMBRANE (PHOBIUS); TMhelix (TMHMM)	no GO terms	no GO terms
622	CP034434.1	Pf-tRNA-Gly	I	Uncharacterised protein	94	3	2.60E-63	68.62						no IPS match	no IPS match	no IPS match
623	CP034435.1	Pf-tRNA-Gly	I	Uncharacterised protein	94	3	2.60E-63	68.62						no IPS match	no IPS match	no IPS match
624	CP034435.1	Pf-tRNA-Gly	I	Uncharacterised protein	256	20	0	85.4	1	C:GO:0005575	C:cellular_component			mobidb-lite (MOBIDB_LITE); mobidb-lite (MOBIDB_LITE); CYTOPLASMIC_DOMAIN (PHOBIUS); NON_CYTOPLASMIC_DOMAIN (PHOBIUS); TRANSMEMBRANE (PHOBIUS); TMhelix (TMHMM)	no GO terms	no GO terms
625	CP034435.1	Pf-tRNA-Gly	I	Uncharacterised protein	242	20	2.44E-177	65.05	1	C:GO:0005575	C:cellular_component			Coil (COILS); Coil (COILS); SIGNAL_PEPTIDE_H_REGION (PHOBIUS); SIGNAL_PEPTIDE_C_REGION (PHOBIUS); SIGNAL_PEPTIDE_N_REGION (PHOBIUS); CYTOPLASMIC_DOMAIN (PHOBIUS); TRANSMEMBRANE (PHOBIUS); NON_CYTOPLASMIC_DOMAIN (PHOBIUS); SIGNAL_PEPTIDE (PHOBIUS); TMhelix (TMHMM); TMhelix (TMHMM)	no GO terms	no GO terms
626	CP034435.1	Pf-tRNA-Gly	I	Uncharacterised protein	265	20	0	66.81						no IPS match	no IPS match	no IPS match
627	CP034435.1	Pf-tRNA-Gly	I	type II toxin-antitoxin system Phd/YefM family antitoxin	83	20	6.81E-53	96.37						PTHR33713:SF7 (PANTHER); PTHR33713 (PANTHER); IPR036165 (SUPERFAMILY)	no GO terms	no GO terms
628	CP034435.1	Pf-tRNA-Gly	I	type II toxin-antitoxin system RelE/ParE family toxin	115	20	1.41E-76	98.87						IPR035093 (G3DSA:3.30.2310.GENE3D); IPR007712 (PFAM)	no GO terms	no GO terms
629	CP034436.1	Pf-tRNA-Gly	I	type II toxin-antitoxin system RelE/ParE family toxin	115	20	1.41E-76	98.87						IPR035093 (G3DSA:3.30.2310.GENE3D); IPR007712 (PFAM)	no GO terms	no GO terms
630	CP034436.1	Pf-tRNA-Gly	I	type II toxin-antitoxin system Phd/YefM family antitoxin	83	20	6.81E-53	96.37						PTHR33713:SF7 (PANTHER); PTHR33713 (PANTHER); IPR036165 (SUPERFAMILY)	no GO terms	no GO terms
631	CP034436.1	Pf-tRNA-Gly	I	Uncharacterised protein	94	3	2.60E-63	68.62						no IPS match	no IPS match	no IPS match
632	CP034436.1	Pf-tRNA-Gly	I	Uncharacterised protein	256	20	0	85.4	1	C:GO:0005575	C:cellular_component			mobidb-lite (MOBIDB_LITE); mobidb-lite (MOBIDB_LITE); CYTOPLASMIC_DOMAIN (PHOBIUS); NON_CYTOPLASMIC_DOMAIN (PHOBIUS); TRANSMEMBRANE (PHOBIUS); TMhelix (TMHMM)	no GO terms	no GO terms
633	CP034436.1	Pf-tRNA-Gly	I	Uncharacterised protein	242	20	2.44E-177	65.05	1	C:GO:0005575	C:cellular_component			Coil (COILS); Coil (COILS); SIGNAL_PEPTIDE_H_REGION (PHOBIUS); SIGNAL_PEPTIDE_C_REGION (PHOBIUS); SIGNAL_PEPTIDE_N_REGION (PHOBIUS); CYTOPLASMIC_DOMAIN (PHOBIUS); TRANSMEMBRANE (PHOBIUS); NON_CYTOPLASMIC_DOMAIN (PHOBIUS); SIGNAL_PEPTIDE (PHOBIUS); TMhelix (TMHMM); TMhelix (TMHMM)	no GO terms	no GO terms
634	CP034436.1	Pf-tRNA-Gly	I	Uncharacterised protein	265	20	0	66.81						no IPS match	no IPS match	no IPS match
635	CP037925.1	Pf-tRNA-Gly	II	hypothetical protein	467	20	0	80.77						no IPS match	no IPS match	no IPS match
636	CP037925.1	Pf-tRNA-Gly	II	cytoplasmic protein	368	20	0	62.6						G3DSA:3.10.450.50 (GENE3D); IPR004027 (PFAM); SSF103642 (SUPERFAMILY)	no GO terms	no GO terms
637	CP037925.1	Pf-tRNA-Gly	II	type II toxin-antitoxin system Phd/YefM family antitoxin	83	20	6.81E-53	96.37						PTHR33713:SF7 (PANTHER); PTHR33713 (PANTHER); IPR036165 (SUPERFAMILY)	no GO terms	no GO terms
638	CP037925.1	Pf-tRNA-Gly	II	type II toxin-antitoxin system RelE/ParE family toxin	115	20	1.06E-76	97.61						IPR007712 (PFAM); IPR035093 (G3DSA:3.30.2310.GENE3D)	no GO terms	no GO terms
639	CP037926.1	Pf-tRNA-Gly	II	DNA-binding protein	65	20	0.516456	69.41	1	F:GO:0003677	F:DNA binding			no IPS match	no IPS match	no IPS match
640	CP037926.1	Pf-tRNA-Gly	II	type II toxin-antitoxin system RelE/ParE family toxin	115	20	1.06E-76	97.61						IPR007712 (PFAM); IPR035093 (G3DSA:3.30.2310.GENE3D)	no GO terms	no GO terms
641	CP037926.1	Pf-tRNA-Gly	II	type II toxin-antitoxin system Phd/YefM family antitoxin	83	20	6.81E-53	96.37						PTHR33713:SF7 (PANTHER); PTHR33713 (PANTHER); IPR036165 (SUPERFAMILY)	no GO terms	no GO terms
642	CP037926.1	Pf-tRNA-Gly	II	cytoplasmic protein	368	20	0	62.6						G3DSA:3.10.450.50 (GENE3D); IPR004027 (PFAM); SSF103642 (SUPERFAMILY)	no GO terms	no GO terms
643	CP037926.1	Pf-tRNA-Gly	II	hypothetical protein	467	20	0	80.77						no IPS match	no IPS match	no IPS match
644	CP039293.1	Pf-tRNA-Gly	II	type II toxin-antitoxin system RelE/ParE family toxin	115	20	1.41E-76	98.87						IPR035093 (G3DSA:3.30.2310.GENE3D); IPR007712 (PFAM)	no GO terms	no GO terms
645	CP039293.1	Pf-tRNA-Gly	II	type II toxin-antitoxin system Phd/YefM family antitoxin	83	20	6.81E-53	96.37						PTHR33713:SF7 (PANTHER); PTHR33713 (PANTHER); IPR036165 (SUPERFAMILY)	no GO terms	no GO terms
646	CP039293.1	Pf-tRNA-Gly	II	Uncharacterised protein	232	20	1.83E-174	96.75	1	C:GO:0005575	C:cellular_component			mobidb-lite (MOBIDB_LITE); mobidb-lite (MOBIDB_LITE); mobidb-lite (MOBIDB_LITE); TRANSMEMBRANE (PHOBIUS); NON_CYTOPLASMIC_DOMAIN (PHOBIUS); CYTOPLASMIC_DOMAIN (PHOBIUS); TMhelix (TMHMM)	no GO terms	no GO terms
647	CP039293.1	Pf-tRNA-Gly	II	ATP-dependent endonuclease	586	20	0	88.94	2	F:GO:0004518; F:GO:0043167	F:nuclease activity; F:ion binding			Coil (COILS); G3DSA:3.40.50.300 (GENE3D); IPR041685 (PFAM); PTHR43581:SF1 (PANTHER); PTHR43581:SF1 (PANTHER); PTHR43581 (PANTHER); PTHR43581 (PANTHER); cd00267 (CDD); IPR034139 (CDD); IPR027417 (SUPERFAMILY)	no GO terms	no GO terms
648	CP039749.1	Pf-tRNA-Met	II	Uncharacterised protein	303	20	0	74.91						no IPS match	no IPS match	no IPS match
649	CP039749.1	Pf-tRNA-Met	II	hypothetical protein	136	20	1.37E-94	99.74	1	C:GO:0005575	C:cellular_component			no IPS match	no IPS match	no IPS match
650	CP039749.1	Pf-tRNA-Met	II	Uncharacterised protein	111	20	8.63E-75	95.32						no IPS match	no IPS match	no IPS match
651	CP039749.1	Pf-tRNA-Met	II	sce7725 family protein	304	20	0	70.96	1	F:GO:0043167	F:ion binding			no IPS match	no IPS match	no IPS match
652	CP039749.1	Pf-tRNA-Sec	II	sce7726 family protein	295	20	0	76.79						no IPS match	no IPS match	no IPS match
653	CP039749.1	Pf-tRNA-Sec	II	Predicted HD superfamily hydrolase	109	20	2.42E-76	60.93	4	P:GO:0006464; F:GO:0016301; F:GO:0043167; C:GO:0005575	P:cellular protein modification process; F:kinase activity; F:ion binding; C:cellular_component			Coil (COILS)	no GO terms	no GO terms
654	CP040127.1	Pf-tRNA-Gly	II	type II toxin-antitoxin system RelE/ParE family toxin	115	20	1.69E-76	97.87						IPR035093 (G3DSA:3.30.2310.GENE3D); IPR007712 (PFAM)	no GO terms	no GO terms
655	CP040127.1	Pf-tRNA-Gly	II	type II toxin-antitoxin system Phd/YefM family antitoxin	83	20	6.81E-53	96.37						PTHR33713:SF7 (PANTHER); PTHR33713 (PANTHER); IPR036165 (SUPERFAMILY)	no GO terms	no GO terms
656	CP040127.1	Pf-tRNA-Gly	II	hypothetical protein	155	20	2.89E-104	76.22	1	C:GO:0005575	C:cellular_component			TRANSMEMBRANE (PHOBIUS); NON_CYTOPLASMIC_DOMAIN (PHOBIUS); TRANSMEMBRANE (PHOBIUS); TRANSMEMBRANE (PHOBIUS); CYTOPLASMIC_DOMAIN (PHOBIUS); CYTOPLASMIC_DOMAIN (PHOBIUS); CYTOPLASMIC_DOMAIN (PHOBIUS); TRANSMEMBRANE (PHOBIUS); NON_CYTOPLASMIC_DOMAIN (PHOBIUS); TMhelix (TMHMM); TMhelix (TMHMM); TMhelix (TMHMM); TMhelix (TMHMM)	no GO terms	no GO terms
657	CP040127.1	Pf-tRNA-Gly	II	Uncharacterised protein	242	20	1.41E-174	96.08	1	C:GO:0005575	C:cellular_component			mobidb-lite (MOBIDB_LITE); mobidb-lite (MOBIDB_LITE); mobidb-lite (MOBIDB_LITE); mobidb-lite (MOBIDB_LITE); mobidb-lite (MOBIDB_LITE); TRANSMEMBRANE (PHOBIUS); CYTOPLASMIC_DOMAIN (PHOBIUS); NON_CYTOPLASMIC_DOMAIN (PHOBIUS); TMhelix (TMHMM)	no GO terms	no GO terms
658	CP040127.1	Pf-tRNA-Gly	II	Uncharacterised protein	200	20	1.75E-145	70.47						no IPS match	no IPS match	no IPS match
659	CP040127.1	Pf-tRNA-Gly	II	Uncharacterised protein	211	20	4.45E-155	73.36						no IPS match	no IPS match	no IPS match
660	FM209186.1	Pf-DR	II	ATP-binding protein	598	20	0	83.27	1	F:GO:0043167	F:ion binding			Coil (COILS); G3DSA:3.40.50.300 (GENE3D); IPR041685 (PFAM); IPR027417 (SUPERFAMILY)	no GO terms	no GO terms
661	FM209186.1	Pf-DR	II	Uncharacterised protein	62	20	6.48E-38	89.21						no IPS match	no IPS match	no IPS match
662	LN831024.1	Pf-DR	I	hypothetical protein	137	20	3.07E-96	84.52	1	C:GO:0005575	C:cellular_component			Coil (COILS); mobidb-lite (MOBIDB_LITE); mobidb-lite (MOBIDB_LITE); mobidb-lite (MOBIDB_LITE)	no GO terms	no GO terms
663	LN831024.1	Pf-DR	I	RNA-directed DNA polymerase	325	20	0	76.85	4	P:GO:0006259; P:GO:0009058; F:GO:0003723; F:GO:0016779	P:DNA metabolic process; P:biosynthetic process; F:RNA binding; F:nucleotidyltransferase activity	EC:2.7.7.49	RNA-directed DNA polymerase	IPR000123 (PRINTS); IPR000477 (PFAM); PTHR34047:SF6 (PANTHER); PTHR34047 (PANTHER); cd03487 (CDD)	P:GO:0006278; F:GO:0003723; F:GO:0003964	P:RNA-dependent DNA biosynthetic process; F:RNA binding; F:RNA-directed DNA polymerase activity
664	LN831024.1	Pf-DR	I	helix-turn-helix transcriptional regulator	108	20	1.77E-71	87.52	1	F:GO:0003677	F:DNA binding			IPR001387 (SMART); IPR001387 (PFAM); G3DSA:1.10.260.40 (GENE3D); PTHR46558 (PANTHER); IPR001387 (PROSITE_PROFILES); IPR001387 (CDD); IPR010982 (SUPERFAMILY)	F:GO:0003677	F:DNA binding
665	LN831024.1	Pf-DR	I	DNA polymerase III subunit alpha	55	3	1.04E-10	55.76	1	C:GO:0005575	C:cellular_component			CYTOPLASMIC_DOMAIN (PHOBIUS); NON_CYTOPLASMIC_DOMAIN (PHOBIUS); TRANSMEMBRANE (PHOBIUS); TMhelix (TMHMM)	no GO terms	no GO terms
666	LN831024.1	Pf-tRNA-Sec	II	Uncharacterised protein	227	20	3.23E-161	79.68						no IPS match	no IPS match	no IPS match
667	LN870292.1	Pf-tmRNA	I	Uncharacterised protein	75	20	9.13E-48	78.93						no IPS match	no IPS match	no IPS match
668	LN870292.1	Pf-tmRNA	I	Uncharacterised protein	232	20	3.10E-174	98.36	1	C:GO:0005575	C:cellular_component			mobidb-lite (MOBIDB_LITE); mobidb-lite (MOBIDB_LITE); mobidb-lite (MOBIDB_LITE); TRANSMEMBRANE (PHOBIUS); NON_CYTOPLASMIC_DOMAIN (PHOBIUS); CYTOPLASMIC_DOMAIN (PHOBIUS); TMhelix (TMHMM)	no GO terms	no GO terms
669	LN870292.1	Pf-tmRNA	I	ATP-binding protein	412	20	0	76.22						IPR027417 (SUPERFAMILY)	no GO terms	no GO terms
670	LN871187.1	Pf-tRNA-Gly	I	RNA-directed DNA polymerase	318	20	0	79.16	4	P:GO:0006259; P:GO:0009058; F:GO:0003723; F:GO:0016779	P:DNA metabolic process; P:biosynthetic process; F:RNA binding; F:nucleotidyltransferase activity	EC:2.7.7.49	RNA-directed DNA polymerase	IPR000123 (PRINTS); IPR000477 (PFAM); PTHR34047:SF6 (PANTHER); PTHR34047 (PANTHER); IPR000477 (PROSITE_PROFILES); cd03487 (CDD); SSF56672 (SUPERFAMILY)	P:GO:0006278; F:GO:0003723; F:GO:0003964	P:RNA-dependent DNA biosynthetic process; F:RNA binding; F:RNA-directed DNA polymerase activity
671	LN871187.1	Pf-tRNA-Gly	I	AAA family ATPase	441	20	0	78.07	1	F:GO:0043167	F:ion binding			IPR003593 (SMART); G3DSA:3.40.50.300 (GENE3D); IPR038729 (PFAM); IPR003959 (PFAM); PTHR43581:SF2 (PANTHER); PTHR43581 (PANTHER); IPR027417 (SUPERFAMILY)	F:GO:0005524	F:ATP binding
672	LN871187.1	Pf-tRNA-Gly	I	type II toxin-antitoxin system RelE/ParE family toxin	115	20	2.09E-76	98.57						IPR007712 (PFAM); IPR035093 (G3DSA:3.30.2310.GENE3D)	no GO terms	no GO terms
673	LN871187.1	Pf-tRNA-Gly	I	type II toxin-antitoxin system Phd/YefM family antitoxin	83	20	6.81E-53	96.37						PTHR33713:SF7 (PANTHER); PTHR33713 (PANTHER); IPR036165 (SUPERFAMILY)	no GO terms	no GO terms
674	LN871187.1	Pf-tRNA-Gly	I	DNA mismatch repair protein MutS	162	20	1.01E-11	74.28	9	P:GO:0006259; P:GO:0006810; P:GO:0006950; P:GO:0051276; F:GO:0003677; F:GO:0004386; F:GO:0004518; F:GO:0043167; C:GO:0005739	P:DNA metabolic process; P:transport; P:response to stress; P:chromosome organization; F:DNA binding; F:helicase activity; F:nuclease activity; F:ion binding; C:mitochondrion	EC:3.6.1.3; EC:3.6.4.12; EC:3.6.1.15	Adenosinetriphosphatase; DNA helicase; Nucleoside-triphosphate phosphatase	mobidb-lite (MOBIDB_LITE); mobidb-lite (MOBIDB_LITE); SIGNAL_PEPTIDE_N_REGION (PHOBIUS); SIGNAL_PEPTIDE (PHOBIUS); NON_CYTOPLASMIC_DOMAIN (PHOBIUS); SIGNAL_PEPTIDE_H_REGION (PHOBIUS); SIGNAL_PEPTIDE_C_REGION (PHOBIUS); SignalP-noTM (SIGNALP_EUK)	no GO terms	no GO terms
675	LN871187.1	Pf-tRNA-Met	I	serine/threonine-protein phosphatase	365	20	0	63.24	2	P:GO:0006470; F:GO:0004722	P:protein dephosphorylation; F:protein serine/threonine phosphatase activity	EC:3.1.3.16	Protein-serine/threonine phosphatase	IPR001932 (SMART); IPR036457 (G3DSA:3.60.40.GENE3D); PF13672 (PFAM); mobidb-lite (MOBIDB_LITE); mobidb-lite (MOBIDB_LITE); mobidb-lite (MOBIDB_LITE); IPR015655 (PANTHER); PTHR13832:SF278 (PANTHER); IPR001932 (PROSITE_PROFILES); IPR001932 (CDD); IPR036457 (SUPERFAMILY)	P:GO:0006470; F:GO:0003824; F:GO:0004722	P:protein dephosphorylation; F:catalytic activity; F:protein serine/threonine phosphatase activity
676	LN871187.1	Pf-tRNA-Met	I	serine/threonine protein kinase	349	20	0	69.97	3	P:GO:0006468; F:GO:0004674; F:GO:0005524	P:protein phosphorylation; F:protein serine/threonine kinase activity; F:ATP binding	EC:2.7.11	Transferring phosphorus-containing groups	IPR000719 (SMART); G3DSA:1.10.510.10 (GENE3D); IPR000719 (PFAM); PTHR24346 (PANTHER); IPR008271 (PROSITE_PATTERNS); IPR000719 (PROSITE_PROFILES); cd14014 (CDD); IPR011009 (SUPERFAMILY)	P:GO:0006468; F:GO:0004672; F:GO:0005524	P:protein phosphorylation; F:protein kinase activity; F:ATP binding
677	LN871187.1	Pf-tRNA-Met	I	serine/threonine protein kinase	357	20	0	78.68	3	P:GO:0006464; F:GO:0016301; F:GO:0043167	P:cellular protein modification process; F:kinase activity; F:ion binding			IPR000719 (SMART); IPR000719 (PFAM); G3DSA:1.10.510.10 (GENE3D); PIRSF000654 (PIRSF); G3DSA:3.30.200.20 (GENE3D); PTHR43289:SF11 (PANTHER); PTHR43289 (PANTHER); IPR008271 (PROSITE_PATTERNS); IPR000719 (PROSITE_PROFILES); cd14014 (CDD); IPR012340 (SUPERFAMILY); IPR011009 (SUPERFAMILY)	P:GO:0006468; F:GO:0004672; F:GO:0005524	P:protein phosphorylation; F:protein kinase activity; F:ATP binding
678	LR130527.1	Pf-tRNA-Met	II	MULTISPECIES: hypothetical protein	136	20	1.03E-93	98.01	1	C:GO:0005575	C:cellular_component			CYTOPLASMIC_DOMAIN (PHOBIUS); TRANSMEMBRANE (PHOBIUS); CYTOPLASMIC_DOMAIN (PHOBIUS); NON_CYTOPLASMIC_DOMAIN (PHOBIUS); NON_CYTOPLASMIC_DOMAIN (PHOBIUS); TRANSMEMBRANE (PHOBIUS); TRANSMEMBRANE (PHOBIUS); TMhelix (TMHMM); TMhelix (TMHMM); TMhelix (TMHMM)	no GO terms	no GO terms
679	LR130527.1	Pf-tRNA-Met	II	hypothetical protein	111	20	3.66E-75	97.57						SIGNAL_PEPTIDE_H_REGION (PHOBIUS); SIGNAL_PEPTIDE_N_REGION (PHOBIUS); SIGNAL_PEPTIDE_C_REGION (PHOBIUS); NON_CYTOPLASMIC_DOMAIN (PHOBIUS); SIGNAL_PEPTIDE (PHOBIUS); SignalP-noTM (SIGNALP_GRAM_NEGATIVE); SignalP-noTM (SIGNALP_EUK); SignalP-TM (SIGNALP_GRAM_POSITIVE)	no GO terms	no GO terms
680	LR130527.1	Pf-tRNA-Met	II	hypothetical protein	223	20	3.33E-155	63.93						no IPS match	no IPS match	no IPS match
681	LR130527.1	Pf-tRNA-Met	II	hypothetical protein	375	20	0	75.37						Coil (COILS); mobidb-lite (MOBIDB_LITE)	no GO terms	no GO terms
682	LR130527.1	Pf-tRNA-Met	II	hypothetical protein	325	20	0	77.3						no IPS match	no IPS match	no IPS match
683	LR130527.1	Pf-tRNA-Sec	II	chromate transporter	96	13	1.66E-59	72.65	1	C:GO:0005575	C:cellular_component			no IPS match	no IPS match	no IPS match
684	LR130527.1	Pf-tRNA-Sec	II	Uncharacterised protein	230	20	6.56E-166	65.94						no IPS match	no IPS match	no IPS match
685	LR130527.1	Pf-tRNA-Sec	II	AraC family transcriptional regulator	90	20	7.04E-59	64.65	1	C:GO:0005575	C:cellular_component			SIGNAL_PEPTIDE (PHOBIUS); SIGNAL_PEPTIDE_H_REGION (PHOBIUS); NON_CYTOPLASMIC_DOMAIN (PHOBIUS); SIGNAL_PEPTIDE_N_REGION (PHOBIUS); SIGNAL_PEPTIDE_C_REGION (PHOBIUS)	no GO terms	no GO terms
686	LR130527.1	Pf-tRNA-Sec	II	ParA family protein	297	20	0	78.73						G3DSA:3.40.50.300 (GENE3D); IPR025669 (PFAM); PTHR13696:SF52 (PANTHER); PTHR13696 (PANTHER); cd02042 (CDD); IPR027417 (SUPERFAMILY)	no GO terms	no GO terms
687	LR130530.1	Pf-tRNA-Met	II	MULTISPECIES: hypothetical protein	136	20	1.03E-93	98.01	1	C:GO:0005575	C:cellular_component			CYTOPLASMIC_DOMAIN (PHOBIUS); TRANSMEMBRANE (PHOBIUS); CYTOPLASMIC_DOMAIN (PHOBIUS); NON_CYTOPLASMIC_DOMAIN (PHOBIUS); NON_CYTOPLASMIC_DOMAIN (PHOBIUS); TRANSMEMBRANE (PHOBIUS); TRANSMEMBRANE (PHOBIUS); TMhelix (TMHMM); TMhelix (TMHMM); TMhelix (TMHMM)	no GO terms	no GO terms
688	LR130530.1	Pf-tRNA-Met	II	hypothetical protein	111	20	3.66E-75	97.57						SIGNAL_PEPTIDE_H_REGION (PHOBIUS); SIGNAL_PEPTIDE_N_REGION (PHOBIUS); SIGNAL_PEPTIDE_C_REGION (PHOBIUS); NON_CYTOPLASMIC_DOMAIN (PHOBIUS); SIGNAL_PEPTIDE (PHOBIUS); SignalP-noTM (SIGNALP_EUK); SignalP-noTM (SIGNALP_GRAM_NEGATIVE); SignalP-TM (SIGNALP_GRAM_POSITIVE)	no GO terms	no GO terms
689	LR130530.1	Pf-tRNA-Met	II	hypothetical protein	228	20	2.36E-158	63.94						no IPS match	no IPS match	no IPS match
690	LR130530.1	Pf-tRNA-Met	II	hypothetical protein	375	20	0	75.37						Coil (COILS); mobidb-lite (MOBIDB_LITE)	no GO terms	no GO terms
691	LR130530.1	Pf-tRNA-Met	II	hypothetical protein	325	20	0	77.3						no IPS match	no IPS match	no IPS match
692	LR130530.1	Pf-tRNA-Sec	II	Uncharacterised protein	230	20	6.56E-166	65.94						no IPS match	no IPS match	no IPS match
693	LR130530.1	Pf-tRNA-Sec	II	3-hydroxyisobutyryl-CoA hydrolase	176	13	9.86E-123	72.11	1	F:GO:0003674	F:molecular_function			SIGNAL_PEPTIDE (PHOBIUS); SIGNAL_PEPTIDE_H_REGION (PHOBIUS); NON_CYTOPLASMIC_DOMAIN (PHOBIUS); SIGNAL_PEPTIDE_N_REGION (PHOBIUS); SIGNAL_PEPTIDE_C_REGION (PHOBIUS)	no GO terms	no GO terms
694	LR130530.1	Pf-tRNA-Sec	II	ParA family protein	297	20	0	78.73						G3DSA:3.40.50.300 (GENE3D); IPR025669 (PFAM); PTHR13696:SF52 (PANTHER); PTHR13696 (PANTHER); cd02042 (CDD); IPR027417 (SUPERFAMILY)	no GO terms	no GO terms
695	LR130531.1	Pf-tRNA-Met	II	MULTISPECIES: hypothetical protein	136	20	1.03E-93	98.01	1	C:GO:0005575	C:cellular_component			CYTOPLASMIC_DOMAIN (PHOBIUS); TRANSMEMBRANE (PHOBIUS); CYTOPLASMIC_DOMAIN (PHOBIUS); NON_CYTOPLASMIC_DOMAIN (PHOBIUS); NON_CYTOPLASMIC_DOMAIN (PHOBIUS); TRANSMEMBRANE (PHOBIUS); TRANSMEMBRANE (PHOBIUS); TMhelix (TMHMM); TMhelix (TMHMM); TMhelix (TMHMM)	no GO terms	no GO terms
696	LR130531.1	Pf-tRNA-Met	II	hypothetical protein	111	20	3.66E-75	97.57						SIGNAL_PEPTIDE_H_REGION (PHOBIUS); SIGNAL_PEPTIDE_N_REGION (PHOBIUS); SIGNAL_PEPTIDE_C_REGION (PHOBIUS); NON_CYTOPLASMIC_DOMAIN (PHOBIUS); SIGNAL_PEPTIDE (PHOBIUS); SignalP-noTM (SIGNALP_GRAM_NEGATIVE); SignalP-noTM (SIGNALP_EUK); SignalP-TM (SIGNALP_GRAM_POSITIVE)	no GO terms	no GO terms
697	LR130531.1	Pf-tRNA-Met	II	hypothetical protein	228	20	2.36E-158	63.94						no IPS match	no IPS match	no IPS match
698	LR130531.1	Pf-tRNA-Met	II	hypothetical protein	375	20	0	75.37						Coil (COILS); mobidb-lite (MOBIDB_LITE)	no GO terms	no GO terms
699	LR130531.1	Pf-tRNA-Met	II	hypothetical protein	299	20	0	78.01						no IPS match	no IPS match	no IPS match
700	LR130531.1	Pf-tRNA-Sec	II	Uncharacterised protein	230	20	6.56E-166	65.94						no IPS match	no IPS match	no IPS match
701	LR130531.1	Pf-tRNA-Sec	II	3-hydroxyisobutyryl-CoA hydrolase	176	13	9.86E-123	72.11	1	F:GO:0003674	F:molecular_function			SIGNAL_PEPTIDE (PHOBIUS); SIGNAL_PEPTIDE_H_REGION (PHOBIUS); NON_CYTOPLASMIC_DOMAIN (PHOBIUS); SIGNAL_PEPTIDE_N_REGION (PHOBIUS); SIGNAL_PEPTIDE_C_REGION (PHOBIUS)	no GO terms	no GO terms
702	LR130531.1	Pf-tRNA-Sec	II	ParA family protein	297	20	0	78.73						G3DSA:3.40.50.300 (GENE3D); IPR025669 (PFAM); PTHR13696:SF52 (PANTHER); PTHR13696 (PANTHER); cd02042 (CDD); IPR027417 (SUPERFAMILY)	no GO terms	no GO terms
703	LR130533.1	Pf-DR	I	hypothetical protein	159	20	1.01E-111	79.81	1	C:GO:0005575	C:cellular_component			SIGNAL_PEPTIDE (PHOBIUS); SIGNAL_PEPTIDE_N_REGION (PHOBIUS); SIGNAL_PEPTIDE_C_REGION (PHOBIUS); SIGNAL_PEPTIDE_H_REGION (PHOBIUS); NON_CYTOPLASMIC_DOMAIN (PHOBIUS); TMhelix (TMHMM)	no GO terms	no GO terms
704	LR130533.1	Pf-DR	I	Uncharacterised protein	543	20	0	74.49	1	C:GO:0005575	C:cellular_component			TRANSMEMBRANE (PHOBIUS); TRANSMEMBRANE (PHOBIUS); CYTOPLASMIC_DOMAIN (PHOBIUS); NON_CYTOPLASMIC_DOMAIN (PHOBIUS); NON_CYTOPLASMIC_DOMAIN (PHOBIUS); CYTOPLASMIC_DOMAIN (PHOBIUS); TRANSMEMBRANE (PHOBIUS); TMhelix (TMHMM); TMhelix (TMHMM); TMhelix (TMHMM)	no GO terms	no GO terms
705	LR130534.1	Pf-DR	I	hypothetical protein	159	20	1.01E-111	79.81	1	C:GO:0005575	C:cellular_component			SIGNAL_PEPTIDE (PHOBIUS); SIGNAL_PEPTIDE_N_REGION (PHOBIUS); SIGNAL_PEPTIDE_C_REGION (PHOBIUS); SIGNAL_PEPTIDE_H_REGION (PHOBIUS); NON_CYTOPLASMIC_DOMAIN (PHOBIUS); TMhelix (TMHMM)	no GO terms	no GO terms
706	LR130534.1	Pf-DR	I	Uncharacterised protein	543	20	0	74.49	1	C:GO:0005575	C:cellular_component			TRANSMEMBRANE (PHOBIUS); TRANSMEMBRANE (PHOBIUS); CYTOPLASMIC_DOMAIN (PHOBIUS); NON_CYTOPLASMIC_DOMAIN (PHOBIUS); NON_CYTOPLASMIC_DOMAIN (PHOBIUS); CYTOPLASMIC_DOMAIN (PHOBIUS); TRANSMEMBRANE (PHOBIUS); TMhelix (TMHMM); TMhelix (TMHMM); TMhelix (TMHMM)	no GO terms	no GO terms
707	LR130535.1	Pf-tRNA-Met	II	MULTISPECIES: hypothetical protein	136	20	1.03E-93	98.01	1	C:GO:0005575	C:cellular_component			CYTOPLASMIC_DOMAIN (PHOBIUS); TRANSMEMBRANE (PHOBIUS); CYTOPLASMIC_DOMAIN (PHOBIUS); NON_CYTOPLASMIC_DOMAIN (PHOBIUS); NON_CYTOPLASMIC_DOMAIN (PHOBIUS); TRANSMEMBRANE (PHOBIUS); TRANSMEMBRANE (PHOBIUS); TMhelix (TMHMM); TMhelix (TMHMM); TMhelix (TMHMM)	no GO terms	no GO terms
708	LR130535.1	Pf-tRNA-Met	II	hypothetical protein	111	20	3.66E-75	97.57						SIGNAL_PEPTIDE_H_REGION (PHOBIUS); SIGNAL_PEPTIDE_N_REGION (PHOBIUS); SIGNAL_PEPTIDE_C_REGION (PHOBIUS); NON_CYTOPLASMIC_DOMAIN (PHOBIUS); SIGNAL_PEPTIDE (PHOBIUS); SignalP-TM (SIGNALP_GRAM_POSITIVE); SignalP-noTM (SIGNALP_GRAM_NEGATIVE); SignalP-noTM (SIGNALP_EUK)	no GO terms	no GO terms
709	LR130535.1	Pf-tRNA-Met	II	hypothetical protein	223	20	3.33E-155	63.93						no IPS match	no IPS match	no IPS match
710	LR130535.1	Pf-tRNA-Met	II	hypothetical protein	375	20	0	75.37						Coil (COILS); mobidb-lite (MOBIDB_LITE)	no GO terms	no GO terms
711	LR130535.1	Pf-tRNA-Met	II	hypothetical protein	325	20	0	77.3						no IPS match	no IPS match	no IPS match
712	LR130535.1	Pf-tRNA-Sec	II	Uncharacterised protein	230	20	6.56E-166	65.94						no IPS match	no IPS match	no IPS match
713	LR130535.1	Pf-tRNA-Sec	II	3-hydroxyisobutyryl-CoA hydrolase	176	13	9.86E-123	72.11	1	F:GO:0003674	F:molecular_function			SIGNAL_PEPTIDE (PHOBIUS); SIGNAL_PEPTIDE_H_REGION (PHOBIUS); NON_CYTOPLASMIC_DOMAIN (PHOBIUS); SIGNAL_PEPTIDE_N_REGION (PHOBIUS); SIGNAL_PEPTIDE_C_REGION (PHOBIUS)	no GO terms	no GO terms
714	LR130535.1	Pf-tRNA-Sec	II	ParA family protein	297	20	0	78.73						G3DSA:3.40.50.300 (GENE3D); IPR025669 (PFAM); PTHR13696:SF52 (PANTHER); PTHR13696 (PANTHER); cd02042 (CDD); IPR027417 (SUPERFAMILY)	no GO terms	no GO terms
715	LR130536.1	Pf-tRNA-Met	II	MULTISPECIES: hypothetical protein	136	20	1.03E-93	98.01	1	C:GO:0005575	C:cellular_component			CYTOPLASMIC_DOMAIN (PHOBIUS); TRANSMEMBRANE (PHOBIUS); CYTOPLASMIC_DOMAIN (PHOBIUS); NON_CYTOPLASMIC_DOMAIN (PHOBIUS); NON_CYTOPLASMIC_DOMAIN (PHOBIUS); TRANSMEMBRANE (PHOBIUS); TRANSMEMBRANE (PHOBIUS); TMhelix (TMHMM); TMhelix (TMHMM); TMhelix (TMHMM)	no GO terms	no GO terms
716	LR130536.1	Pf-tRNA-Met	II	hypothetical protein	111	20	3.66E-75	97.57						SIGNAL_PEPTIDE_H_REGION (PHOBIUS); SIGNAL_PEPTIDE_N_REGION (PHOBIUS); SIGNAL_PEPTIDE_C_REGION (PHOBIUS); NON_CYTOPLASMIC_DOMAIN (PHOBIUS); SIGNAL_PEPTIDE (PHOBIUS); SignalP-TM (SIGNALP_GRAM_POSITIVE); SignalP-noTM (SIGNALP_GRAM_NEGATIVE); SignalP-noTM (SIGNALP_EUK)	no GO terms	no GO terms
717	LR130536.1	Pf-tRNA-Met	II	hypothetical protein	223	20	3.33E-155	63.93						no IPS match	no IPS match	no IPS match
718	LR130536.1	Pf-tRNA-Met	II	hypothetical protein	375	20	0	75.37						Coil (COILS); mobidb-lite (MOBIDB_LITE)	no GO terms	no GO terms
719	LR130536.1	Pf-tRNA-Met	II	hypothetical protein	325	20	0	77.3						no IPS match	no IPS match	no IPS match
720	LR130536.1	Pf-tRNA-Sec	II	Uncharacterised protein	230	20	6.56E-166	65.94						no IPS match	no IPS match	no IPS match
721	LR130536.1	Pf-tRNA-Sec	II	3-hydroxyisobutyryl-CoA hydrolase	176	13	9.86E-123	72.11	1	F:GO:0003674	F:molecular_function			SIGNAL_PEPTIDE (PHOBIUS); SIGNAL_PEPTIDE_H_REGION (PHOBIUS); NON_CYTOPLASMIC_DOMAIN (PHOBIUS); SIGNAL_PEPTIDE_N_REGION (PHOBIUS); SIGNAL_PEPTIDE_C_REGION (PHOBIUS)	no GO terms	no GO terms
722	LR130536.1	Pf-tRNA-Sec	II	ParA family protein	297	20	0	78.73						G3DSA:3.40.50.300 (GENE3D); IPR025669 (PFAM); PTHR13696:SF52 (PANTHER); PTHR13696 (PANTHER); cd02042 (CDD); IPR027417 (SUPERFAMILY)	no GO terms	no GO terms
723	LR130537.1	Pf-tRNA-Met	II	MULTISPECIES: hypothetical protein	136	20	1.03E-93	98.01	1	C:GO:0005575	C:cellular_component			CYTOPLASMIC_DOMAIN (PHOBIUS); TRANSMEMBRANE (PHOBIUS); CYTOPLASMIC_DOMAIN (PHOBIUS); NON_CYTOPLASMIC_DOMAIN (PHOBIUS); NON_CYTOPLASMIC_DOMAIN (PHOBIUS); TRANSMEMBRANE (PHOBIUS); TRANSMEMBRANE (PHOBIUS); TMhelix (TMHMM); TMhelix (TMHMM); TMhelix (TMHMM)	no GO terms	no GO terms
724	LR130537.1	Pf-tRNA-Met	II	hypothetical protein	111	20	3.66E-75	97.57						NON_CYTOPLASMIC_DOMAIN (PHOBIUS); SIGNAL_PEPTIDE (PHOBIUS); SIGNAL_PEPTIDE_C_REGION (PHOBIUS); SIGNAL_PEPTIDE_N_REGION (PHOBIUS); SIGNAL_PEPTIDE_H_REGION (PHOBIUS); SignalP-noTM (SIGNALP_EUK); SignalP-TM (SIGNALP_GRAM_POSITIVE); SignalP-noTM (SIGNALP_GRAM_NEGATIVE)	no GO terms	no GO terms
725	LR130537.1	Pf-tRNA-Met	II	hypothetical protein	228	20	2.36E-158	63.94						no IPS match	no IPS match	no IPS match
726	LR130537.1	Pf-tRNA-Met	II	hypothetical protein	375	20	0	75.37						Coil (COILS); mobidb-lite (MOBIDB_LITE)	no GO terms	no GO terms
727	LR130537.1	Pf-tRNA-Met	II	hypothetical protein	325	20	0	77.3						no IPS match	no IPS match	no IPS match
728	LR130537.1	Pf-tRNA-Sec	II	Uncharacterised protein	230	20	6.56E-166	65.94						no IPS match	no IPS match	no IPS match
729	LR130537.1	Pf-tRNA-Sec	II	3-hydroxyisobutyryl-CoA hydrolase	176	13	9.86E-123	72.11	1	F:GO:0003674	F:molecular_function			SIGNAL_PEPTIDE (PHOBIUS); SIGNAL_PEPTIDE_H_REGION (PHOBIUS); NON_CYTOPLASMIC_DOMAIN (PHOBIUS); SIGNAL_PEPTIDE_N_REGION (PHOBIUS); SIGNAL_PEPTIDE_C_REGION (PHOBIUS)	no GO terms	no GO terms
730	LR130537.1	Pf-tRNA-Sec	II	ParA family protein	297	20	0	78.73						G3DSA:3.40.50.300 (GENE3D); IPR025669 (PFAM); PTHR13696:SF52 (PANTHER); PTHR13696 (PANTHER); cd02042 (CDD); IPR027417 (SUPERFAMILY)	no GO terms	no GO terms
731	LR134308.1	Pf-tRNA-Gly	I	DUF3800 domain-containing protein	285	20	0	89.93						IPR024524 (PFAM)	no GO terms	no GO terms
732	LR134308.1	Pf-tRNA-Gly	I	Uncharacterised protein	232	20	5.49E-174	99.16	1	C:GO:0005575	C:cellular_component			mobidb-lite (MOBIDB_LITE); mobidb-lite (MOBIDB_LITE); mobidb-lite (MOBIDB_LITE); TRANSMEMBRANE (PHOBIUS); NON_CYTOPLASMIC_DOMAIN (PHOBIUS); CYTOPLASMIC_DOMAIN (PHOBIUS); TMhelix (TMHMM)	no GO terms	no GO terms
733	LR134308.1	Pf-tRNA-Gly	I	YfbU family protein	167	20	3.52E-120	62.38	6	P:GO:0006950; P:GO:0006974; C:GO:0005623; C:GO:0005829	P:response to stress; P:cellular response to DNA damage stimulus; C:cell; C:cytosol			IPR005587 (PFAM); IPR023146 (G3DSA:1.10.3190.GENE3D); SSF116960 (SUPERFAMILY)	no GO terms	no GO terms
734	LR134309.1	Pf-tRNA-Met	II	MULTISPECIES: hypothetical protein	136	20	1.03E-93	98.01	1	C:GO:0005575	C:cellular_component			CYTOPLASMIC_DOMAIN (PHOBIUS); TRANSMEMBRANE (PHOBIUS); CYTOPLASMIC_DOMAIN (PHOBIUS); NON_CYTOPLASMIC_DOMAIN (PHOBIUS); NON_CYTOPLASMIC_DOMAIN (PHOBIUS); TRANSMEMBRANE (PHOBIUS); TRANSMEMBRANE (PHOBIUS); TMhelix (TMHMM); TMhelix (TMHMM); TMhelix (TMHMM)	no GO terms	no GO terms
735	LR134309.1	Pf-tRNA-Met	II	Cro/CI family transcriptional regulator	264	20	0	86.3	7	P:GO:0001666; P:GO:0019217; P:GO:0045892; P:GO:0045926; P:GO:0045927; F:GO:0003677	P:response to hypoxia; P:regulation of fatty acid metabolic process; P:negative regulation of transcription, DNA-templated; P:negative regulation of growth; P:positive regulation of growth; F:DNA binding			IPR001387 (SMART); IPR007374 (SMART); IPR007374 (PFAM); IPR001387 (PFAM); G3DSA:1.10.260.40 (GENE3D); G3DSA:2.30.130.30 (GENE3D); IPR001387 (PROSITE_PROFILES); IPR001387 (CDD); IPR015947 (SUPERFAMILY); IPR010982 (SUPERFAMILY)	F:GO:0003677	F:DNA binding
736	LR134309.1	Pf-tRNA-Met	II	GNAT family N-acetyltransferase	512	20	0	98.42	1	F:GO:0016746	F:transferase activity, transferring acyl groups	EC:2.3.1.5	Arylamine N-acetyltransferase	G3DSA:3.40.630.30 (GENE3D); IPR000182 (PFAM); IPR000182 (PROSITE_PROFILES); cd18699 (CDD); IPR016181 (SUPERFAMILY)	F:GO:0008080	F:N-acetyltransferase activity
737	LR134309.1	Pf-tRNA-Met	II	AAA family ATPase	175	20	2.76E-124	83.12				EC:2.7.4.3	Adenylate kinase	PF13207 (PFAM); G3DSA:3.40.50.300 (GENE3D); IPR027417 (SUPERFAMILY)	no GO terms	no GO terms
738	LR134309.1c	Pf-tRNA-Gly	II	Uncharacterised protein	230	20	2.58E-173	96.26	1	C:GO:0005575	C:cellular_component			mobidb-lite (MOBIDB_LITE); mobidb-lite (MOBIDB_LITE); mobidb-lite (MOBIDB_LITE); TRANSMEMBRANE (PHOBIUS); NON_CYTOPLASMIC_DOMAIN (PHOBIUS); CYTOPLASMIC_DOMAIN (PHOBIUS); TMhelix (TMHMM)	no GO terms	no GO terms
739	LR134309.1c	Pf-tRNA-Gly	II	Uncharacterised protein	504	20	0	74.14						mobidb-lite (MOBIDB_LITE)	no GO terms	no GO terms
740	LR134309.1c	Pf-tRNA-Gly	II	type II toxin-antitoxin system Phd/YefM family antitoxin	83	20	6.81E-53	96.37						PTHR33713:SF7 (PANTHER); PTHR33713 (PANTHER); IPR036165 (SUPERFAMILY)	no GO terms	no GO terms
741	LR134309.1c	Pf-tRNA-Gly	II	type II toxin-antitoxin system RelE/ParE family toxin	115	20	5.90E-77	98.35						IPR007712 (PFAM); IPR035093 (G3DSA:3.30.2310.GENE3D)	no GO terms	no GO terms
742	LR134342.1	Pf-tRNA-Met	II	hypothetical protein	136	20	1.37E-94	99.74	1	C:GO:0005575	C:cellular_component			CYTOPLASMIC_DOMAIN (PHOBIUS); CYTOPLASMIC_DOMAIN (PHOBIUS); TRANSMEMBRANE (PHOBIUS); NON_CYTOPLASMIC_DOMAIN (PHOBIUS); NON_CYTOPLASMIC_DOMAIN (PHOBIUS); TRANSMEMBRANE (PHOBIUS); TRANSMEMBRANE (PHOBIUS); TMhelix (TMHMM); TMhelix (TMHMM); TMhelix (TMHMM)	no GO terms	no GO terms
743	LR134342.1	Pf-tRNA-Met	II	Uncharacterised protein	110	20	9.07E-74	91.24						SIGNAL_PEPTIDE_H_REGION (PHOBIUS); NON_CYTOPLASMIC_DOMAIN (PHOBIUS); SIGNAL_PEPTIDE_N_REGION (PHOBIUS); SIGNAL_PEPTIDE_C_REGION (PHOBIUS); SIGNAL_PEPTIDE (PHOBIUS); SignalP-noTM (SIGNALP_GRAM_NEGATIVE); SignalP-noTM (SIGNALP_EUK); SignalP-TM (SIGNALP_GRAM_POSITIVE)	no GO terms	no GO terms
744	LR134342.1	Pf-tRNA-Met	II	Uncharacterised protein	823	20	0	70.08						IPR039444 (PFAM); IPR027417 (SUPERFAMILY)	no GO terms	no GO terms
745	LR590473.1	Pf-tRNA-Met	II	Uncharacterised protein	303	20	0	74.91						no IPS match	no IPS match	no IPS match
746	LR590473.1	Pf-tRNA-Met	II	hypothetical protein	136	20	1.37E-94	99.74	1	C:GO:0005575	C:cellular_component			no IPS match	no IPS match	no IPS match
747	LR590473.1	Pf-tRNA-Met	II	Uncharacterised protein	111	20	8.63E-75	95.32						no IPS match	no IPS match	no IPS match
748	LR590473.1	Pf-tRNA-Met	II	sce7725 family protein	304	20	0	70.96	1	F:GO:0043167	F:ion binding			no IPS match	no IPS match	no IPS match
749	LR590473.1	Pf-tRNA-Sec	II	sce7726 family protein	295	20	0	76.79						no IPS match	no IPS match	no IPS match
750	LR590473.1	Pf-tRNA-Sec	II	Predicted HD superfamily hydrolase	109	20	2.42E-76	60.93	4	P:GO:0006464; F:GO:0016301; F:GO:0043167; C:GO:0005575	P:cellular protein modification process; F:kinase activity; F:ion binding; C:cellular_component			Coil (COILS)	no GO terms	no GO terms
751	LT608330.1	Pf-DR	I	ParA family protein	343	20	0	82.15	4	P:GO:0071941; F:GO:0016491; C:GO:0005622; C:GO:0032991	P:nitrogen cycle metabolic process; F:oxidoreductase activity; C:intracellular; C:protein-containing complex	EC:1.18.6.1; EC:1.18.6	Nitrogenase; Acting on iron-sulfur proteins as donors	Coil (COILS); IPR025669 (PFAM); G3DSA:3.40.50.300 (GENE3D); PTHR13696:SF52 (PANTHER); PTHR13696 (PANTHER); cd02042 (CDD); IPR027417 (SUPERFAMILY)	no GO terms	no GO terms
752	LT608330.1	Pf-DR	I	Uncharacterised protein	175	20	4.41E-125	76.51						IPR041519 (PFAM)	no GO terms	no GO terms
753	LT673656.1	Pf-tRNA-Gly	II	Uncharacterised protein	74	20	7.08E-47	85.88						G3DSA:2.30.30.1060 (GENE3D)	no GO terms	no GO terms
754	LT673656.1	Pf-tRNA-Gly	II	hypothetical protein	339	20	0	64.96						no IPS match	no IPS match	no IPS match
755	LT673656.1	Pf-tRNA-Gly	II	hypothetical protein	180	20	1.75E-119	66.39	1	C:GO:0005575	C:cellular_component			TRANSMEMBRANE (PHOBIUS); CYTOPLASMIC_DOMAIN (PHOBIUS); CYTOPLASMIC_DOMAIN (PHOBIUS); NON_CYTOPLASMIC_DOMAIN (PHOBIUS); TRANSMEMBRANE (PHOBIUS); TRANSMEMBRANE (PHOBIUS); CYTOPLASMIC_DOMAIN (PHOBIUS); NON_CYTOPLASMIC_DOMAIN (PHOBIUS); TRANSMEMBRANE (PHOBIUS); TMhelix (TMHMM); TMhelix (TMHMM); TMhelix (TMHMM); TMhelix (TMHMM)	no GO terms	no GO terms
756	LT673656.1	Pf-tRNA-Gly	II	hypothetical protein PERCYII10_4380	311	20	0	68.19						Coil (COILS)	no GO terms	no GO terms
757	LT673656.1	Pf-tRNA-Met	I	Uncharacterised protein	145	20	6.74E-100	94.9	1	C:GO:0005575	C:cellular_component			Coil (COILS); TRANSMEMBRANE (PHOBIUS); CYTOPLASMIC_DOMAIN (PHOBIUS); TRANSMEMBRANE (PHOBIUS); CYTOPLASMIC_DOMAIN (PHOBIUS); NON_CYTOPLASMIC_DOMAIN (PHOBIUS); TMhelix (TMHMM); TMhelix (TMHMM)	no GO terms	no GO terms
758	LT673656.1	Pf-tRNA-Met	I	DUF4338 domain-containing protein	399	20	0	79.75						IPR025639 (PFAM)	no GO terms	no GO terms
759	LT673656.1	Pf-tRNA-Met	I	hypothetical protein PERCYII10_4858	386	20	0	67.95	1	C:GO:0005575	C:cellular_component			NON_CYTOPLASMIC_DOMAIN (PHOBIUS); TRANSMEMBRANE (PHOBIUS); CYTOPLASMIC_DOMAIN (PHOBIUS); CYTOPLASMIC_DOMAIN (PHOBIUS); TRANSMEMBRANE (PHOBIUS); TMhelix (TMHMM); TMhelix (TMHMM)	no GO terms	no GO terms
760	LT673656.1	Pf-tRNA-Met	I	hypothetical protein PERCYII10_4859	378	20	0	58.52						no IPS match	no IPS match	no IPS match
761	LT673656.1	Pf-tRNA-Met	I	protein kinase family protein	883	20	0	70.05	3	P:GO:0006464; F:GO:0016301; F:GO:0043167	P:cellular protein modification process; F:kinase activity; F:ion binding			IPR000719 (SMART); G3DSA:1.10.510.10 (GENE3D); IPR000719 (PROSITE_PROFILES); cd00180 (CDD); IPR011009 (SUPERFAMILY)	P:GO:0006468; F:GO:0004672; F:GO:0005524	P:protein phosphorylation; F:protein kinase activity; F:ATP binding
762	LT673656.1	Pf-tRNA-Met	I	hypothetical protein PERCYII10_4861	258	20	0	70.46	1	C:GO:0005575	C:cellular_component			mobidb-lite (MOBIDB_LITE); mobidb-lite (MOBIDB_LITE); CYTOPLASMIC_DOMAIN (PHOBIUS); NON_CYTOPLASMIC_DOMAIN (PHOBIUS); TRANSMEMBRANE (PHOBIUS); TMhelix (TMHMM)	no GO terms	no GO terms
763	LT673656.1	Pf-tRNA-Met	I	ribonuclease inhibitor	95	20	3.87E-64	69.9						IPR035905 (G3DSA:3.30.370.GENE3D); IPR000468 (PFAM); IPR035905 (SUPERFAMILY)	no GO terms	no GO terms
764	LT673656.1	Pf-tRNA-Met	I	hypothetical protein	128	20	3.53E-89	67.78						no IPS match	no IPS match	no IPS match
765	LT883143.1	Pf-tRNA-Met	I	Uncharacterised protein	294	20	0	77.63	1	C:GO:0005575	C:cellular_component			NON_CYTOPLASMIC_DOMAIN (PHOBIUS); NON_CYTOPLASMIC_DOMAIN (PHOBIUS); TRANSMEMBRANE (PHOBIUS); NON_CYTOPLASMIC_DOMAIN (PHOBIUS); CYTOPLASMIC_DOMAIN (PHOBIUS); TRANSMEMBRANE (PHOBIUS); CYTOPLASMIC_DOMAIN (PHOBIUS); CYTOPLASMIC_DOMAIN (PHOBIUS); TRANSMEMBRANE (PHOBIUS); NON_CYTOPLASMIC_DOMAIN (PHOBIUS); TRANSMEMBRANE (PHOBIUS); TRANSMEMBRANE (PHOBIUS); SignalP-TM (SIGNALP_EUK); TMhelix (TMHMM); TMhelix (TMHMM); TMhelix (TMHMM); TMhelix (TMHMM); TMhelix (TMHMM); TMhelix (TMHMM)	no GO terms	no GO terms
766	LT883143.1	Pf-tRNA-Met	I	Uncharacterised protein	111	20	6.27E-75	97.52						mobidb-lite (MOBIDB_LITE); mobidb-lite (MOBIDB_LITE); SIGNAL_PEPTIDE_H_REGION (PHOBIUS); SIGNAL_PEPTIDE_N_REGION (PHOBIUS); SIGNAL_PEPTIDE_C_REGION (PHOBIUS); NON_CYTOPLASMIC_DOMAIN (PHOBIUS); SIGNAL_PEPTIDE (PHOBIUS); SignalP-TM (SIGNALP_GRAM_POSITIVE); SignalP-noTM (SIGNALP_EUK); SignalP-noTM (SIGNALP_GRAM_NEGATIVE)	no GO terms	no GO terms
767	LT883143.1	Pf-tRNA-Met	I	restriction endonuclease	220	20	6.99E-158	85.85	8	P:GO:0006265; P:GO:0009307; P:GO:0090305; F:GO:0003677; F:GO:0003916; F:GO:0004519; C:GO:0005694; C:GO:0016021	P:DNA topological change; P:DNA restriction-modification system; P:nucleic acid phosphodiester bond hydrolysis; F:DNA binding; F:DNA topoisomerase activity; F:endonuclease activity; C:chromosome; C:integral component of membrane			IPR007560 (PFAM); IPR011856 (G3DSA:3.40.1350.GENE3D); PTHR30015 (PANTHER); TRANSMEMBRANE (PHOBIUS); TRANSMEMBRANE (PHOBIUS); CYTOPLASMIC_DOMAIN (PHOBIUS); NON_CYTOPLASMIC_DOMAIN (PHOBIUS); CYTOPLASMIC_DOMAIN (PHOBIUS); IPR011335 (SUPERFAMILY); TMhelix (TMHMM); TMhelix (TMHMM)	P:GO:0009307; F:GO:0003676; F:GO:0003677; F:GO:0004518; F:GO:0004519	P:DNA restriction-modification system; F:nucleic acid binding; F:DNA binding; F:nuclease activity; F:endonuclease activity
768	LT883143.1	Pf-tRNA-Met	I	MULTISPECIES: hypothetical protein	137	20	1.21E-95	97.88						no IPS match	no IPS match	no IPS match
769	LT883143.1	Pf-tRNA-Met	I	cytidine deaminase	588	20	0	88.11	4	F:GO:0008270; F:GO:0016787	F:zinc ion binding; F:hydrolase activity	EC:3.5.4.12; EC:3.5.4.5	dCMP deaminase; Cytidine deaminase	G3DSA:3.40.140.10 (GENE3D); IPR002125 (PFAM); G3DSA:3.40.50.300 (GENE3D); IPR015517 (PANTHER); PTHR11086:SF18 (PANTHER); IPR016192 (PROSITE_PATTERNS); IPR002125 (PROSITE_PROFILES); IPR035105 (CDD); IPR016193 (SUPERFAMILY)	F:GO:0003824; F:GO:0008270; F:GO:0016787	F:catalytic activity; F:zinc ion binding; F:hydrolase activity
770	LT883143.1	Pf-tRNA-Met	I	ATPase	413	20	0	91.07	1	F:GO:0043167	F:ion binding			IPR041685 (PFAM); G3DSA:3.40.50.300 (GENE3D); G3DSA:3.40.50.300 (GENE3D); IPR014555 (PIRSF); IPR003959 (PFAM); PTHR40396 (PANTHER); IPR027417 (SUPERFAMILY)	F:GO:0005524	F:ATP binding
771	LT883143.1	Pf-tRNA-Met	I	DUF4276 family protein	200	20	4.62E-145	84.59						IPR025455 (PFAM)	no GO terms	no GO terms
772	LT969520.1	Pf-DR	II	ATP-binding protein	598	20	0	83.3	1	F:GO:0043167	F:ion binding			Coil (COILS); IPR041685 (PFAM); G3DSA:3.40.50.300 (GENE3D); IPR027417 (SUPERFAMILY)	no GO terms	no GO terms
773	LT969520.1	Pf-DR	II	Uncharacterised protein	62	20	6.48E-38	89.21						no IPS match	no IPS match	no IPS match

Table S3. Distribution of accessory proteins (n=773) in the Pf prophages.

# of Pf prophages	Protein	Lenght	Conserved Domain	#GO	GO Ids	GO Names	EC	Enzyme name	InterPro Ids	InterPro GO IDs	InterPro GO names
2	HAD hydrolase-like protein	411	Gph_COG0546<br>HAD_like_cd01427<br>PRK13288_PRK13288<br>HAD_2_pfam13419<br>ComFC_COG1040<br>HAD-SF-IA-v1_TIGR01549	4	P:GO:0034641; P:GO:0044281	P:cellular nitrogen compound metabolic process; P:small molecule metabolic process	EC:3.1.3.18; EC:3.6.4.12	Phosphoglycolate phosphatase; DNA helicase	G3DSA:3.40.50.2020 (GENE3D); IPR023214 (G3DSA:3.40.50.GENE3D); IPR041492 (PFAM); IPR000836 (CDD); cd01427 (CDD); IPR029057 (SUPERFAMILY); IPR036412 (SUPERFAMILY)	P:GO:0009116	P:nucleoside metabolic process
2	NYN domain-containing protein	219	No Domains	_	_	_	_	_	no IPS match	no IPS match	no IPS match
2	thymidylate synthase	65	No Domains	4	P:GO:0009058; P:GO:0034641; P:GO:0044281; F:GO:0008168	P:biosynthetic process; P:cellular nitrogen compound metabolic process; P:small molecule metabolic process; F:methyltransferase activity	EC:2.1.1.45	Thymidylate synthase	no IPS match	no IPS match	no IPS match
2	hypothetical protein	48	wac_PHA02607	_	_	_	_	_	no IPS match	no IPS match	no IPS match
2	sce7725 family protein	317	No Domains	_	_	_	_	_	no IPS match	no IPS match	no IPS match
2	helix-turn-helix transcriptional regulator	108	HTH_XRE_cd00093<br>HTH_XRE_smart00530<br>HTH_3_pfam01381<br>XRE_COG1476<br>PRK09943_PRK09943	1	F:GO:0003677	F:DNA binding	_	_	IPR001387 (SMART); IPR001387 (PFAM); G3DSA:1.10.260.40 (GENE3D); PTHR46558 (PANTHER); IPR001387 (PROSITE_PROFILES); IPR001387 (CDD); IPR010982 (SUPERFAMILY)	F:GO:0003677	F:DNA binding
2	cytidine deaminase	588	deoxycytidylate_deaminase_cd01286<br>ComEB_COG2131<br>cd_PHA02588<br>dCMP_cyt_deam_1_pfam00383<br>ComEB_TIGR02571<br>CoaE_COG0237	4	F:GO:0008270; F:GO:0016787	F:zinc ion binding; F:hydrolase activity	EC:3.5.4.12; EC:3.5.4.5	dCMP deaminase; Cytidine deaminase	G3DSA:3.40.140.10 (GENE3D); IPR002125 (PFAM); G3DSA:3.40.50.300 (GENE3D); IPR015517 (PANTHER); PTHR11086:SF18 (PANTHER); IPR016192 (PROSITE_PATTERNS); IPR002125 (PROSITE_PROFILES); IPR035105 (CDD); IPR016193 (SUPERFAMILY)	F:GO:0003824; F:GO:0008270; F:GO:0016787	F:catalytic activity; F:zinc ion binding; F:hydrolase activity
2	DNA polymerase III subunit alpha	55	No Domains	1	C:GO:0005575	C:cellular_component	_	_	CYTOPLASMIC_DOMAIN (PHOBIUS); NON_CYTOPLASMIC_DOMAIN (PHOBIUS); TRANSMEMBRANE (PHOBIUS); TMhelix (TMHMM)	no GO terms	no GO terms
2	DUF3892 domain-containing protein	94	DUF3892_pfam13031	_	_	_	_	_	IPR024997 (PFAM); mobidb-lite (MOBIDB_LITE)	no GO terms	no GO terms
2	thymidylate synthase	173	TS_Pyrimidine_HMase_cd00351<br>ThyA_COG0207<br>thyA_PRK00956<br>thym_sym_TIGR03284<br>Thymidylat_synt_pfam00303	4	P:GO:0008150; F:GO:0008168	P:biological_process; F:methyltransferase activity	EC:2.1.1.45	Thymidylate synthase	IPR036926 (G3DSA:3.30.572.GENE3D); IPR023451 (PFAM); PTHR11548:SF9 (PANTHER); PTHR11548 (PANTHER); IPR036926 (SUPERFAMILY)	no GO terms	no GO terms
2	restriction endonuclease	245	HNH_pfam01844<br>HNHc_cd00085<br>HNHc_smart00507<br>COG3440_COG3440	1	F:GO:0004518	F:nuclease activity	_	_	Coil (COILS); IPR003615 (SMART); G3DSA:3.30.40.60 (GENE3D); IPR002711 (PFAM); mobidb-lite (MOBIDB_LITE); mobidb-lite (MOBIDB_LITE); PTHR33877:SF1 (PANTHER); PTHR33877 (PANTHER); IPR003615 (CDD)	F:GO:0003676; F:GO:0004519	F:nucleic acid binding; F:endonuclease activity
2	Thymidylate synthase	265	No Domains	2	F:GO:0003674	F:molecular_function	EC:2.1.1.45	Thymidylate synthase	no IPS match	no IPS match	no IPS match
2	hypothetical protein	311	No Domains	_	_	_	_	_	IPR041271 (PFAM)	no GO terms	no GO terms
2	DUF2290 domain-containing protein	224	DUF2290_pfam10053<br>COG5619_COG5619	_	_	_	_	_	IPR018742 (PFAM)	no GO terms	no GO terms
2	MULTISPECIES: hypothetical protein	270	No Domains	_	_	_	_	_	mobidb-lite (MOBIDB_LITE)	no GO terms	no GO terms
2	GNAT family N-acetyltransferase	179	No Domains	1	F:GO:0016746	F:transferase activity, transferring acyl groups	EC:2.3.1.5	Arylamine N-acetyltransferase	no IPS match	no IPS match	no IPS match
2	ATP-dependent DNA helicase RecG	628	COG2865_COG2865<br>HATPase_c_4_pfam13749<br>RRM_smart00360<br>RRM_DAZL_BOULE_cd12412<br>RRM_1_pfam00076<br>RRM_COG0724<br>PABP-1234_TIGR01628	2	F:GO:0003674	F:molecular_function	EC:3.6.4.12	DNA helicase	IPR000504 (SMART); IPR007421 (PFAM); IPR038475 (G3DSA:3.30.565.GENE3D); IPR000504 (PFAM); IPR025831 (PFAM); IPR012677 (G3DSA:3.30.70.GENE3D); IPR038461 (G3DSA:3.30.950.GENE3D); PTHR30595 (PANTHER); PTHR30595:SF6 (PANTHER); IPR000504 (PROSITE_PROFILES); IPR035979 (SUPERFAMILY)	F:GO:0003676	F:nucleic acid binding
2	ATP-binding protein	598	AAA_15_pfam13175<br>YbjD_COG3593	1	F:GO:0043167	F:ion binding	_	_	Coil (COILS); G3DSA:3.40.50.300 (GENE3D); IPR041685 (PFAM); IPR027417 (SUPERFAMILY)	no GO terms	no GO terms
2	DNA-binding protein	88	No Domains	1	F:GO:0003677	F:DNA binding	_	_	G3DSA:1.10.260.40 (GENE3D); mobidb-lite (MOBIDB_LITE); mobidb-lite (MOBIDB_LITE)	no GO terms	no GO terms
2	sce7726 family protein	285	No Domains	_	_	_	_	_	no IPS match	no IPS match	no IPS match
2	SEC-C domain-containing protein (SWI2 ATPases)	368	PRK05590_PRK05590<br>SEC-C_pfam02810<br>SWIM_PBPRA1643_TIGR04102<br>YecA_COG3318	_	_	_	_	_	G3DSA:3.10.450.50 (GENE3D); IPR004027 (PFAM); SSF103642 (SUPERFAMILY)	no GO terms	no GO terms
2	Superfamily I DNA and RNA helicases	735	COG3972_COG3972<br>UvrD_C_2_pfam13538<br>SF1_C_UvrD_cd18807<br>DEXDc_smart00487	4	P:GO:0051276; F:GO:0003677; F:GO:0004386; F:GO:0043167	P:chromosome organization; F:DNA binding; F:helicase activity; F:ion binding	EC:3.6.1.3; EC:3.6.4.12; EC:3.6.1.15	Adenosinetriphosphatase; DNA helicase; Nucleoside-triphosphate phosphatase	IPR027785 (PFAM); G3DSA:3.40.50.300 (GENE3D); G3DSA:3.40.50.300 (GENE3D); IPR000212 (PANTHER); PTHR11070:SF3 (PANTHER); cd18807 (CDD); IPR027417 (SUPERFAMILY)	F:GO:0003677; F:GO:0003678; F:GO:0005524	F:DNA binding; F:DNA helicase activity; F:ATP binding
2	YfbU family protein	167	YfbU_pfam03887<br>PRK05445_PRK05445<br>YfbU_COG3013	6	P:GO:0006950; P:GO:0006974; C:GO:0005623; C:GO:0005829	P:response to stress; P:cellular response to DNA damage stimulus; C:cell; C:cytosol	_	_	IPR023146 (G3DSA:1.10.3190.GENE3D); IPR005587 (PFAM); SSF116960 (SUPERFAMILY)	no GO terms	no GO terms
2	IS30 family transposase ISPpu17	320	Tra8_COG2826<br>HTH_38_pfam13936	4	P:GO:0006313; P:GO:0015074; F:GO:0003677; F:GO:0004803	P:transposition, DNA-mediated; P:DNA integration; F:DNA binding; F:transposase activity	_	_	IPR036397 (G3DSA:3.30.420.GENE3D); IPR001584 (PFAM); IPR025246 (PFAM); PTHR10948 (PANTHER); IPR001598 (PROSITE_PATTERNS); IPR001584 (PROSITE_PROFILES); IPR012337 (SUPERFAMILY)	P:GO:0006313; P:GO:0015074; F:GO:0003676; F:GO:0003677; F:GO:0004803	P:transposition, DNA-mediated; P:DNA integration; F:nucleic acid binding; F:DNA binding; F:transposase activity
2	ankyrin repeat domain-containing protein	186	Ank_2_pfam12796<br>ANKYR_COG0666<br>PTZ00322_PTZ00322<br>KTSC_pfam13619	1	F:GO:0003674	F:molecular_function	_	_	IPR002110 (SMART); IPR036770 (G3DSA:1.25.40.GENE3D); IPR020683 (PFAM); IPR025309 (PFAM); PTHR24134 (PANTHER); IPR020683 (PROSITE_PROFILES); IPR002110 (PROSITE_PROFILES); IPR036770 (SUPERFAMILY)	F:GO:0005515	F:protein binding
2	RNA-directed DNA polymerase	325	RT_Bac_retron_II_cd03487<br>RVT_1_pfam00078	4	P:GO:0006259; P:GO:0009058; F:GO:0003723; F:GO:0016779	P:DNA metabolic process; P:biosynthetic process; F:RNA binding; F:nucleotidyltransferase activity	EC:2.7.7.49	RNA-directed DNA polymerase	IPR000123 (PRINTS); IPR000477 (PFAM); PTHR34047:SF6 (PANTHER); PTHR34047 (PANTHER); cd03487 (CDD)	P:GO:0006278; F:GO:0003723; F:GO:0003964	P:RNA-dependent DNA biosynthetic process; F:RNA binding; F:RNA-directed DNA polymerase activity
2	ImmA/IrrE family metallo-endopeptidase	362	Peptidase_M78_pfam06114<br>antidote_HigA_TIGR02607<br>ImmA_COG2856<br>VapI_COG3093	1	F:GO:0003677	F:DNA binding	_	_	G3DSA:1.10.260.40 (GENE3D); IPR010359 (PFAM); IPR001387 (CDD); IPR010982 (SUPERFAMILY)	F:GO:0003677	F:DNA binding
2	NACHT domain-containing protein	714	NACHT_pfam05729	_	_	_	_	_	IPR027417 (SUPERFAMILY)	no GO terms	no GO terms
2	Uncharacterised protein	823	SIR2-like_cd01406<br>SIR2_2_pfam13289	_	_	_	_	_	IPR039444 (PFAM); IPR027417 (SUPERFAMILY)	no GO terms	no GO terms
2	Uncharacterised protein	66	No Domains	1	C:GO:0005575	C:cellular_component	_	_	no IPS match	no IPS match	no IPS match
2	Uncharacterised protein	294	No Domains	1	C:GO:0005575	C:cellular_component	_	_	NON_CYTOPLASMIC_DOMAIN (PHOBIUS); NON_CYTOPLASMIC_DOMAIN (PHOBIUS); TRANSMEMBRANE (PHOBIUS); NON_CYTOPLASMIC_DOMAIN (PHOBIUS); CYTOPLASMIC_DOMAIN (PHOBIUS); TRANSMEMBRANE (PHOBIUS); CYTOPLASMIC_DOMAIN (PHOBIUS); CYTOPLASMIC_DOMAIN (PHOBIUS); TRANSMEMBRANE (PHOBIUS); NON_CYTOPLASMIC_DOMAIN (PHOBIUS); TRANSMEMBRANE (PHOBIUS); TRANSMEMBRANE (PHOBIUS); TRANSMEMBRANE (PHOBIUS); SignalP-TM (SIGNALP_EUK); TMhelix (TMHMM); TMhelix (TMHMM); TMhelix (TMHMM); TMhelix (TMHMM); TMhelix (TMHMM); TMhelix (TMHMM)	no GO terms	no GO terms
2	hypothetical protein	467	No Domains	_	_	_	_	_	no IPS match	no IPS match	no IPS match
2	DNA mismatch repair protein MutS	162	AAA_17_pfam13207<br>AdkA_COG2019<br>PRK04040_PRK04040	9	P:GO:0006259; P:GO:0006810; P:GO:0006950; P:GO:0051276; F:GO:0003677; F:GO:0004386; F:GO:0004518; F:GO:0043167; C:GO:0005739	P:DNA metabolic process; P:transport; P:response to stress; P:chromosome organization; F:DNA binding; F:helicase activity; F:nuclease activity; F:ion binding; C:mitochondrion	EC:3.6.1.3; EC:3.6.4.12; EC:3.6.1.15	Adenosinetriphosphatase; DNA helicase; Nucleoside-triphosphate phosphatase	mobidb-lite (MOBIDB_LITE); mobidb-lite (MOBIDB_LITE); SIGNAL_PEPTIDE_N_REGION (PHOBIUS); SIGNAL_PEPTIDE (PHOBIUS); NON_CYTOPLASMIC_DOMAIN (PHOBIUS); SIGNAL_PEPTIDE_H_REGION (PHOBIUS); SIGNAL_PEPTIDE_C_REGION (PHOBIUS); SignalP-noTM (SIGNALP_EUK)	no GO terms	no GO terms
2	hypothetical protein	258	No Domains	1	C:GO:0005575	C:cellular_component	_	_	mobidb-lite (MOBIDB_LITE); mobidb-lite (MOBIDB_LITE); mobidb-lite (MOBIDB_LITE); NON_CYTOPLASMIC_DOMAIN (PHOBIUS); CYTOPLASMIC_DOMAIN (PHOBIUS); TRANSMEMBRANE (PHOBIUS); TMhelix (TMHMM)	no GO terms	no GO terms
2	DUF262 domain-containing protein [Pseudomonas aeruginosa]	350	DUF262_pfam03235	_	_	_	_	_	IPR014966 (SMART); IPR014966 (PFAM)	no GO terms	no GO terms
2	FRG domain-containing protein	254	FRG_smart00901<br>FRG_pfam08867	_	_	_	_	_	IPR014966 (SMART); IPR014966 (PFAM)	no GO terms	no GO terms
3	Fis family transcriptional regulator	369	AAA_pfam00004<br>SpoVK_COG0464<br>CDC48_TIGR01243<br>hflB_PRK10733<br>AAA_cd00009<br>AAA_smart00382<br>HTH_38_pfam13936<br>Tra8_COG2826<br>RepA_COG3598<br>spore_sigH_TIGR02859	2	F:GO:0003677; F:GO:0043167	F:DNA binding; F:ion binding	_	_	Coil (COILS); IPR003593 (SMART); G3DSA:3.40.50.300 (GENE3D); IPR003959 (PFAM); IPR025246 (PFAM); PTHR23073:SF29 (PANTHER); PTHR23073 (PANTHER); cd00009 (CDD); IPR027417 (SUPERFAMILY)	F:GO:0005524	F:ATP binding
3	restriction endonuclease	220	Mrr_cat_pfam04471<br>Mrr_COG1715<br>PRK06319_PRK06319	8	P:GO:0006265; P:GO:0009307; P:GO:0090305; F:GO:0003677; F:GO:0003916; F:GO:0004519; C:GO:0005694; C:GO:0016021	P:DNA topological change; P:DNA restriction-modification system; P:nucleic acid phosphodiester bond hydrolysis; F:DNA binding; F:DNA topoisomerase activity; F:endonuclease activity; C:chromosome; C:integral component of membrane	_	_	IPR007560 (PFAM); IPR011856 (G3DSA:3.40.1350.GENE3D); PTHR30015 (PANTHER); TRANSMEMBRANE (PHOBIUS); TRANSMEMBRANE (PHOBIUS); CYTOPLASMIC_DOMAIN (PHOBIUS); NON_CYTOPLASMIC_DOMAIN (PHOBIUS); CYTOPLASMIC_DOMAIN (PHOBIUS); IPR011335 (SUPERFAMILY); TMhelix (TMHMM); TMhelix (TMHMM)	P:GO:0009307; F:GO:0003676; F:GO:0003677; F:GO:0004518; F:GO:0004519	P:DNA restriction-modification system; F:nucleic acid binding; F:DNA binding; F:nuclease activity; F:endonuclease activity
3	DUF4276 family protein	200	DUF4276_pfam14103	_	_	_	_	_	IPR025455 (PFAM)	no GO terms	no GO terms
3	sce7726 family protein	193	No Domains	_	_	_	_	_	no IPS match	no IPS match	no IPS match
3	Uncharacterised protein	360	No Domains	_	_	_	_	_	no IPS match	no IPS match	no IPS match
3	DNA cytosine methyltransferase	347	Dcm_COG0270<br>DNA_methylase_pfam00145<br>Cyt_C5_DNA_methylase_cd00315<br>dcm_TIGR00675<br>PRK10458_PRK10458	2	P:GO:0090116; F:GO:0003886	P:C-5 methylation of cytosine; F:DNA (cytosine-5-)-methyltransferase activity	EC:2.1.1.37	DNA (cytosine-5-)-methyltransferase	IPR001525 (PRINTS); G3DSA:3.90.120.10 (GENE3D); IPR001525 (PFAM); G3DSA:3.40.50.150 (GENE3D); IPR001525 (TIGRFAM); PTHR10629 (PANTHER); PTHR10629:SF11 (PANTHER); IPR031303 (PROSITE_PATTERNS); IPR018117 (PROSITE_PATTERNS); IPR001525 (PROSITE_PROFILES); IPR029063 (SUPERFAMILY)	F:GO:0008168	F:methyltransferase activity
3	S8 family peptidase	732	Peptidases_S8_Subtilisin_like_2 cd04847<br>Peptidase_S8 pfam00082	1	F:GO:0008233	F:peptidase activity	EC:3.4.21	Acting on peptide bonds (peptidases)	IPR036852 (G3DSA:3.40.50.GENE3D); IPR000209 (PFAM); IPR034074 (CDD); IPR036852 (SUPERFAMILY)	P:GO:0006508; F:GO:0004252	P:proteolysis; F:serine-type endopeptidase activity
3	Uncharacterised protein	227	No Domains	_	_	_	_	_	no IPS match	no IPS match	no IPS match
3	DNA-processing protein DprA	323	DNA_processg_A_pfam02481<br>Smf_COG0758<br>dprA_TIGR00732<br>PRK10736_PRK10736	1	P:GO:0008150	P:biological_process	_	_	G3DSA:3.40.50.450 (GENE3D); IPR003488 (PFAM); IPR003488 (PANTHER); SSF102405 (SUPERFAMILY)	P:GO:0009294	P:DNA mediated transformation
3	ImmA/IrrE family metallo-endopeptidase	278	ImmA_COG2856<br>Peptidase_M78_pfam06114	_	_	_	_	_	IPR010359 (PFAM)	no GO terms	no GO terms
3	Uncharacterised protein	165	MET3_COG2046_ATP sulfurylase (sulfate adenylyltransferase)<br>sopT_TIGR00339_ATP sulphurylase	1	C:GO:0005575	C:cellular_component	_	_	CYTOPLASMIC_DOMAIN (PHOBIUS); NON_CYTOPLASMIC_DOMAIN (PHOBIUS); TRANSMEMBRANE (PHOBIUS)	no GO terms	no GO terms
3	type 1 glutamine amidotransferase	119	No Domains	1	P:GO:0006520	P:cellular amino acid metabolic process	_	_	no IPS match	no IPS match	no IPS match
3	N-acetyltransferase	224	No Domains	1	F:GO:0003674	F:molecular_function	_	_	IPR016181 (SUPERFAMILY)	no GO terms	no GO terms
3	Uncharacterised protein	109	_	_	_	_	_	no IPS match	no IPS match	no IPS match	347
3	Uncharacterised protein	265	No Domains	1	C:GO:0005575	C:cellular_component	_	_	TRANSMEMBRANE (PHOBIUS); CYTOPLASMIC_DOMAIN (PHOBIUS); NON_CYTOPLASMIC_DOMAIN (PHOBIUS); CYTOPLASMIC_DOMAIN (PHOBIUS); TRANSMEMBRANE (PHOBIUS); TRANSMEMBRANE (PHOBIUS); NON_CYTOPLASMIC_DOMAIN (PHOBIUS); TMhelix (TMHMM); TMhelix (TMHMM); TMhelix (TMHMM)	no GO terms	no GO terms
3	Uncharacterised protein	356	Beta_protein_pfam14350	_	_	_	_	IPR025683 (PFAM)	no GO terms	no GO terms	89
3	(deoxy)nucleoside triphosphate pyrophosphohydrolase	129	MutT_pyrophosphohydrolase_cd03425<br>PRK10546_PRK10546<br>NUDIX_4_pfam14815<br>mutt TIGR00586<br>YjhB COG1051<br>PRK10546_PRK10546<br>NUDIX_4_pfam14815	4	P:GO:0006281; F:GO:0008413	P:DNA repair; F:8-oxo-7,8-dihydroguanosine triphosphate pyrophosphatase activity	EC:3.6.1.55; EC:3.6.1.65; EC:3.6.1.9	8-oxo-dGTP diphosphatase; (d)CTP diphosphatase; Nucleotide diphosphatase	IPR020476 (PRINTS); IPR000086 (PFAM); G3DSA:3.90.79.10 (GENE3D); PTHR47707 (PANTHER); IPR020084 (PROSITE_PATTERNS); IPR000086 (PROSITE_PROFILES); cd03425 (CDD); IPR015797 (SUPERFAMILY)	F:GO:0016787	F:hydrolase activity
3	ATP-dependent endonuclease	586	AAA_21_pfam13304<br>YbjD_COG3593<br>TOPRIM_OLD_cd01026<br>ABC_SMC3_euk_cd03272<br>AAA15_COG1106<br>SMC_N_pfam02463<br>ABC_SMC_barmotin_cd03278<br>recF_PRK00064<br>cbiO_TIGR01166<br>PRK14242_PRK14242	2	F:GO:0004518; F:GO:0043167	F:nuclease activity; F:ion binding	_	_	Coil (COILS); G3DSA:3.40.50.300 (GENE3D); IPR041685 (PFAM); PTHR43581:SF1 (PANTHER); PTHR43581:SF1 (PANTHER); PTHR43581 (PANTHER); PTHR43581 (PANTHER); IPR034139 (CDD); cd00267 (CDD); IPR027417 (SUPERFAMILY)	no GO terms	no GO terms
3	ATPase	413	COG4637_COG4637<br>AAA_21_pfam13304<br>restrict_AAA_1_TIGR04435<br>ABC_Rad50_cd03240<br>recF_PRK00064	1	F:GO:0043167	F:ion binding	_	_	IPR041685 (PFAM); G3DSA:3.40.50.300 (GENE3D); G3DSA:3.40.50.300 (GENE3D); IPR014555 (PIRSF); IPR003959 (PFAM); PTHR40396 (PANTHER); IPR027417 (SUPERFAMILY)	F:GO:0005524	F:ATP binding
3	DUF4238 domain-containing protein	344	DUF4238_pfam14022<br>LMWPTP_cd16343	_	_	_	_	_	IPR025332 (PFAM)	no GO terms	no GO terms
4	serine/threonine protein kinase	349	STKc_PknB_like_cd14014<br>S_TKc_smart00220<br>Pkinase_pfam00069<br>SPS1_COG0515<br>PLN00009_PLN00009<br>TOMM_kin_cyc_TIGR03903	3	P:GO:0006468; F:GO:0004674; F:GO:0005524	P:protein phosphorylation; F:protein serine/threonine kinase activity; F:ATP binding	EC:2.7.11	Transferring phosphorus-containing groups	IPR000719 (SMART); G3DSA:1.10.510.10 (GENE3D); IPR000719 (PFAM); PTHR24346 (PANTHER); IPR008271 (PROSITE_PATTERNS); IPR000719 (PROSITE_PROFILES); cd14014 (CDD); IPR011009 (SUPERFAMILY)	P:GO:0006468; F:GO:0004672; F:GO:0005524	P:protein phosphorylation; F:protein kinase activity; F:ATP binding
4	serine/threonine protein kinase	357	STKc_PknB_like_cd14014<br>S_TKc_smart00220<br>Pkinase_pfam00069<br>SPS1_COG0515<br>pknD_PRK13184<br>TOMM_kin_cyc_TIGR03903	3	P:GO:0006464; F:GO:0016301; F:GO:0043167	P:cellular protein modification process; F:kinase activity; F:ion binding	_	_	IPR000719 (SMART); IPR000719 (PFAM); G3DSA:1.10.510.10 (GENE3D); PIRSF000654 (PIRSF); G3DSA:3.30.200.20 (GENE3D); PTHR43289:SF11 (PANTHER); PTHR43289 (PANTHER); IPR008271 (PROSITE_PATTERNS); IPR000719 (PROSITE_PROFILES); cd14014 (CDD); IPR012340 (SUPERFAMILY); IPR011009 (SUPERFAMILY)	P:GO:0006468; F:GO:0004672; F:GO:0005524	P:protein phosphorylation; F:protein kinase activity; F:ATP binding
4	hypothetical protein	159	No Domains	1	C:GO:0005575	C:cellular_component	_	_	NON_CYTOPLASMIC_DOMAIN (PHOBIUS); SIGNAL_PEPTIDE_C_REGION (PHOBIUS); SIGNAL_PEPTIDE_N_REGION (PHOBIUS); SIGNAL_PEPTIDE_H_REGION (PHOBIUS); SIGNAL_PEPTIDE (PHOBIUS); TMhelix (TMHMM)	no GO terms	no GO terms
4	hypothetical protein	137	cdk7_TIGR00570	1	C:GO:0005575	C:cellular_component	_	_	Coil (COILS); mobidb-lite (MOBIDB_LITE); mobidb-lite (MOBIDB_LITE); mobidb-lite (MOBIDB_LITE)	no GO terms	no GO terms
4	serine/threonine-protein phosphatase	365	PTC1_COG0631<br>PP2Cc_cd00143<br>PP2Cc_smart00332<br>PRK14559_PRK14559<br>PP2C_pfam00481	2	P:GO:0006470; F:GO:0004722	P:protein dephosphorylation; F:protein serine/threonine phosphatase activity	EC:3.1.3.16	Protein-serine/threonine phosphatase	IPR001932 (SMART); IPR036457 (G3DSA:3.60.40.GENE3D); PF13672 (PFAM); mobidb-lite (MOBIDB_LITE); mobidb-lite (MOBIDB_LITE); mobidb-lite (MOBIDB_LITE); IPR015655 (PANTHER); PTHR13832:SF278 (PANTHER); IPR001932 (PROSITE_PROFILES); IPR001932 (CDD); IPR036457 (SUPERFAMILY)	P:GO:0006470; F:GO:0003824; F:GO:0004722	P:protein dephosphorylation; F:catalytic activity; F:protein serine/threonine phosphatase activity
4	Uncharacterised protein	62	No Domains	_	_	_	_	_	no IPS match	no IPS match	no IPS match
4	Uncharacterised protein	75	No Domains	_	_	_	_	_	no IPS match	no IPS match	no IPS match
4	Uncharacterised protein	543	No Domains	1	C:GO:0005575	C:cellular_component	_	_	TRANSMEMBRANE (PHOBIUS); TRANSMEMBRANE (PHOBIUS); CYTOPLASMIC_DOMAIN (PHOBIUS); NON_CYTOPLASMIC_DOMAIN (PHOBIUS); NON_CYTOPLASMIC_DOMAIN (PHOBIUS); CYTOPLASMIC_DOMAIN (PHOBIUS); TRANSMEMBRANE (PHOBIUS); TMhelix (TMHMM); TMhelix (TMHMM); TMhelix (TMHMM)	no GO terms	no GO terms
4	hypothetical protein	230	No Domains	1	C:GO:0005575	C:cellular_component	_	_	CYTOPLASMIC_DOMAIN (PHOBIUS); TRANSMEMBRANE (PHOBIUS); NON_CYTOPLASMIC_DOMAIN (PHOBIUS); CYTOPLASMIC_DOMAIN (PHOBIUS); TRANSMEMBRANE (PHOBIUS); NON_CYTOPLASMIC_DOMAIN (PHOBIUS); TRANSMEMBRANE (PHOBIUS); TMhelix (TMHMM); TMhelix (TMHMM); TMhelix (TMHMM)	no GO terms	no GO terms
4	IS30 family transposase	342	Tra8_COG2826<br>HTH_38_pfam13936	4	P:GO:0006313; P:GO:0015074; F:GO:0003677; F:GO:0004803	P:transposition, DNA-mediated; P:DNA integration; F:DNA binding; F:transposase activity	_	_	IPR036397 (G3DSA:3.30.420.GENE3D); IPR025246 (PFAM); IPR001584 (PFAM); PTHR10948 (PANTHER); IPR001598 (PROSITE_PATTERNS); IPR001584 (PROSITE_PROFILES); IPR012337 (SUPERFAMILY)	P:GO:0006313; P:GO:0015074; F:GO:0003676; F:GO:0003677; F:GO:0004803	P:transposition, DNA-mediated; P:DNA integration; F:nucleic acid binding; F:DNA binding; F:transposase activity
5	Uncharacterised protein	227	wac_PHA02607	1	C:GO:0005575	C:cellular_component	_	_	Coil (COILS); NON_CYTOPLASMIC_DOMAIN (PHOBIUS); NON_CYTOPLASMIC_DOMAIN (PHOBIUS); CYTOPLASMIC_DOMAIN (PHOBIUS); TRANSMEMBRANE (PHOBIUS); TRANSMEMBRANE (PHOBIUS); TMhelix (TMHMM); TMhelix (TMHMM)	no GO terms	no GO terms
5	Uncharacterised protein	244	No Domains	1	C:GO:0005575	C:cellular_component	_	_	Coil (COILS); SIGNAL_PEPTIDE_C_REGION (PHOBIUS); SIGNAL_PEPTIDE_N_REGION (PHOBIUS); TRANSMEMBRANE (PHOBIUS); CYTOPLASMIC_DOMAIN (PHOBIUS); NON_CYTOPLASMIC_DOMAIN (PHOBIUS); SIGNAL_PEPTIDE (PHOBIUS); SIGNAL_PEPTIDE_H_REGION (PHOBIUS); SignalP-TM (SIGNALP_EUK); TMhelix (TMHMM); TMhelix (TMHMM)	no GO terms	no GO terms
5	Uncharacterised protein	98	No Domains	_	_	_	_	_	no IPS match	no IPS match	no IPS match
5	Uncharacterised protein	330	No Domains	_	_	_	_	_	no IPS match	no IPS match	no IPS match
5	ParA family protein	343	BcsQ_COG1192<br>AAA_31_pfam13614<br>ParAB_family_cd02042<br>PHA02518_PHA02518<br>partition_RepA_TIGR03453	4	P:GO:0071941; F:GO:0016491; C:GO:0005622; C:GO:0032991	P:nitrogen cycle metabolic process; F:oxidoreductase activity; C:intracellular; C:protein-containing complex	EC:1.18.6.1; EC:1.18.6	Nitrogenase; Acting on iron-sulfur proteins as donors	Coil (COILS); IPR025669 (PFAM); G3DSA:3.40.50.300 (GENE3D); PTHR13696:SF52 (PANTHER); PTHR13696 (PANTHER); cd02042 (CDD); IPR027417 (SUPERFAMILY)	no GO terms	no GO terms
5	ATP-binding protein	412	No Domains	_	_	_	_	_	IPR027417 (SUPERFAMILY)	no GO terms	no GO terms
5	Uncharacterised protein	175	No Domains	_	_	_	_	_	IPR041519 (PFAM)	no GO terms	no GO terms
5	Uncharacterised protein	97	No Domains	_	_	_	_	_	no IPS match	no IPS match	no IPS match
5	nucleotidyltransferase	421	NT_2-5OAS_ClassI-CCAase_cd05400	1	F:GO:0003674	F:molecular_function	_	_	PF18144 (PFAM); G3DSA:3.30.460.10 (GENE3D); IPR040511 (PFAM); cd05400 (CDD); SSF81301 (SUPERFAMILY)	no GO terms	no GO terms
5	hypothetical protein	155	No Domains	1	C:GO:0005575	C:cellular_component	_	_	TRANSMEMBRANE (PHOBIUS); NON_CYTOPLASMIC_DOMAIN (PHOBIUS); TRANSMEMBRANE (PHOBIUS); TRANSMEMBRANE (PHOBIUS); CYTOPLASMIC_DOMAIN (PHOBIUS); CYTOPLASMIC_DOMAIN (PHOBIUS); CYTOPLASMIC_DOMAIN (PHOBIUS); TRANSMEMBRANE (PHOBIUS); NON_CYTOPLASMIC_DOMAIN (PHOBIUS); TMhelix (TMHMM); TMhelix (TMHMM); TMhelix (TMHMM); TMhelix (TMHMM)	no GO terms	no GO terms
5	SLATT domain-containing protein	196	No Domains	1	C:GO:0005575	C:cellular_component	_	_	IPR041119 (PFAM); NON_CYTOPLASMIC_DOMAIN (PHOBIUS); CYTOPLASMIC_DOMAIN (PHOBIUS); TRANSMEMBRANE (PHOBIUS); TRANSMEMBRANE (PHOBIUS); NON_CYTOPLASMIC_DOMAIN (PHOBIUS); TRANSMEMBRANE (PHOBIUS); CYTOPLASMIC_DOMAIN (PHOBIUS); TMhelix (TMHMM); TMhelix (TMHMM); TMhelix (TMHMM)	no GO terms	no GO terms
5	Uncharacterised protein	363	No Domains	1	C:GO:0005575	C:cellular_component	_	_	CYTOPLASMIC_DOMAIN (PHOBIUS); TRANSMEMBRANE (PHOBIUS); NON_CYTOPLASMIC_DOMAIN (PHOBIUS); CYTOPLASMIC_DOMAIN (PHOBIUS); TRANSMEMBRANE (PHOBIUS); TMhelix (TMHMM); TMhelix (TMHMM)	no GO terms	no GO terms
5	3-hydroxyisobutyryl-CoA hydrolase	176	No Domains	1	F:GO:0003674	F:molecular_function	_	_	SIGNAL_PEPTIDE (PHOBIUS); SIGNAL_PEPTIDE_H_REGION (PHOBIUS); NON_CYTOPLASMIC_DOMAIN (PHOBIUS); SIGNAL_PEPTIDE_N_REGION (PHOBIUS); SIGNAL_PEPTIDE_C_REGION (PHOBIUS)	no GO terms	no GO terms
5	Uncharacterised protein	242	No Domains	1	C:GO:0005575	C:cellular_component	_	_	Coil (COILS); Coil (COILS); SIGNAL_PEPTIDE_H_REGION (PHOBIUS); SIGNAL_PEPTIDE_C_REGION (PHOBIUS); SIGNAL_PEPTIDE_N_REGION (PHOBIUS); CYTOPLASMIC_DOMAIN (PHOBIUS); TRANSMEMBRANE (PHOBIUS); NON_CYTOPLASMIC_DOMAIN (PHOBIUS); SIGNAL_PEPTIDE (PHOBIUS); TMhelix (TMHMM); TMhelix (TMHMM)	no GO terms	no GO terms
6	AAA family ATPase	441	AAA_21_pfam13304<br>ABC_NikE_OppD_transporters_cd03257<br>AAA_23_pfam13476<br>LivF_COG0410<br>urea_trans_UrtE_TIGR03410<br>ZnuC_COG1121<br>AAA_smart00382<br>modC_PRK11144	1	F:GO:0043167	F:ion binding	_	_	IPR003593 (SMART); G3DSA:3.40.50.300 (GENE3D); IPR038729 (PFAM); IPR003959 (PFAM); PTHR43581:SF2 (PANTHER); PTHR43581 (PANTHER); IPR027417 (SUPERFAMILY)	F:GO:0005524	F:ATP binding
6	ParA family protein	297	BcsQ_COG1192<br>AAA_31_pfam13614<br>ParAB_family_cd02042<br>partition_RepA_TIGR03453<br>PRK13869_PRK13869	_	_	_	_	_	G3DSA:3.40.50.300 (GENE3D); IPR025669 (PFAM); PTHR13696:SF52 (PANTHER); PTHR13696 (PANTHER); cd02042 (CDD); IPR027417 (SUPERFAMILY)	no GO terms	no GO terms
6	Uncharacterised protein	94	No Domains	_	_	_	_	_	no IPS match	no IPS match	no IPS match
6	Uncharacterised protein	230	No Domains	_	_	_	_	_	no IPS match	no IPS match	no IPS match
6	RNA-directed DNA polymerase	306	RT_Bac_retron_II_cd03487<br>YkfC_COG3344<br>RVT_1_pfam00078	4	P:GO:0006259; P:GO:0009058; F:GO:0003723; F:GO:0016779	P:DNA metabolic process; P:biosynthetic process; F:RNA binding; F:nucleotidyltransferase activity	EC:2.7.7.49	RNA-directed DNA polymerase	IPR000123 (PRINTS); IPR000477 (PFAM); PTHR34047 (PANTHER); PTHR34047:SF6 (PANTHER); IPR000477 (PROSITE_PROFILES); cd03487 (CDD); SSF56672 (SUPERFAMILY)	P:GO:0006278; F:GO:0003723; F:GO:0003964	P:RNA-dependent DNA biosynthetic process; F:RNA binding; F:RNA-directed DNA polymerase activity
7	hypothetical protein	325	No Domains	_	_	_	_	_	no IPS match	no IPS match	no IPS match
7	hypothetical protein	228	No Domains	_	_	_	_	_	no IPS match	no IPS match	no IPS match
7	Uncharacterised protein	504	No Domains	_	_	_	_	_	mobidb-lite (MOBIDB_LITE)	no GO terms	no GO terms
7	hypothetical protein	375	No Domains	_	_	_	_	_	Coil (COILS); mobidb-lite (MOBIDB_LITE)	no GO terms	no GO terms
8	Uncharacterised protein	200	No Domains	_	_	_	_	_	no IPS match	no IPS match	no IPS match
8	Uncharacterised protein	211	No Domains	_	_	_	_	_	no IPS match	no IPS match	no IPS match
10	GNAT family N-acetyltransferase	348	PIN_VapC_like_cd18699	1	F:GO:0016746	F:transferase activity, transferring acyl groups	EC:2.3.1.5	Arylamine N-acetyltransferase	IPR000182 (PFAM); G3DSA:3.40.630.30 (GENE3D); IPR000182 (PROSITE_PROFILES); cd18699 (CDD); IPR016181 (SUPERFAMILY)	F:GO:0008080	F:N-acetyltransferase activity
11	Uncharacterised protein	171	No Domains								
11	Cro/CI family transcriptional regulator	264	COG4933_COG4933<br>PRK12279_PRK12279<br>HTH_31_pfam13560<br>HTH_XRE_smart00530<br>HTH_XRE_cd00093<br>couple_hipB_TIGR03070<br>ASCH_smart01022<br>COG3620_COG3620<br>PRK09726_PRK09726<br>ASCH_ASC-1_like_cd06554	7	P:GO:0001666; P:GO:0019217; P:GO:0045892; P:GO:0045926; P:GO:0045927; F:GO:0003677	P:response to hypoxia; P:regulation of fatty acid metabolic process; P:negative regulation of transcription, DNA-templated; P:negative regulation of growth; P:positive regulation of growth; F:DNA binding	_	_	IPR001387 (SMART); IPR007374 (SMART); IPR007374 (PFAM); IPR001387 (PFAM); G3DSA:1.10.260.40 (GENE3D); G3DSA:2.30.130.30 (GENE3D); IPR001387 (PROSITE_PROFILES); IPR001387 (CDD); IPR015947 (SUPERFAMILY); IPR010982 (SUPERFAMILY)	F:GO:0003677	F:DNA binding
11	AAA family ATPase	175	AAA_17_pfam13207<br>AdkA_COG2019<br>PRK04040_PRK04040	_	_	_	EC:2.7.4.3	Adenylate kinase	PF13207 (PFAM); G3DSA:3.40.50.300 (GENE3D); IPR027417 (SUPERFAMILY)	no GO terms	no GO terms
14	Uncharacterised protein	74	No Domains	_	_	_	_	_	G3DSA:2.30.30.1060 (GENE3D)	no GO terms	no GO terms
12	hypothetical protein	125	No Domains	_	_	_	_	_	no IPS match	no IPS match	no IPS match
16	Uncharacterised protein	145	No Domains	1	C:GO:0005575	C:cellular_component	_	_	Coil (COILS); TRANSMEMBRANE (PHOBIUS); NON_CYTOPLASMIC_DOMAIN (PHOBIUS); CYTOPLASMIC_DOMAIN (PHOBIUS); TRANSMEMBRANE (PHOBIUS); CYTOPLASMIC_DOMAIN (PHOBIUS); TMhelix (TMHMM); TMhelix (TMHMM)	no GO terms	no GO terms
38	hypothetical protein	111	No Domains	_	_	_	_	_	SIGNAL_PEPTIDE_H_REGION (PHOBIUS); SIGNAL_PEPTIDE_N_REGION (PHOBIUS); SIGNAL_PEPTIDE_C_REGION (PHOBIUS); NON_CYTOPLASMIC_DOMAIN (PHOBIUS); SIGNAL_PEPTIDE (PHOBIUS); SignalP-noTM (SIGNALP_GRAM_NEGATIVE); SignalP-noTM (SIGNALP_EUK); SignalP-TM (SIGNALP_GRAM_POSITIVE)	no GO terms	no GO terms
41	hypothetical protein	136	No Domains	1	C:GO:0005575	C:cellular_component	_	_	CYTOPLASMIC_DOMAIN (PHOBIUS); CYTOPLASMIC_DOMAIN (PHOBIUS); TRANSMEMBRANE (PHOBIUS); NON_CYTOPLASMIC_DOMAIN (PHOBIUS); NON_CYTOPLASMIC_DOMAIN (PHOBIUS); TRANSMEMBRANE (PHOBIUS); TRANSMEMBRANE (PHOBIUS); TMhelix (TMHMM); TMhelix (TMHMM); TMhelix (TMHMM)	no GO terms	no GO terms
48	type II toxin-antitoxin system Phd/YefM family antitoxin	83	PhdYeFM_antitox_pfam02604	_	_	_	_	_	PTHR33713:SF7 (PANTHER); PTHR33713 (PANTHER); IPR036165 (SUPERFAMILY)	no GO terms	no GO terms
49	type II toxin-antitoxin system RelE/ParE family toxin	115	ParE_COG3668<br>ParE_toxin_pfam05016	_	_	_	_	_	IPR007712 (PFAM); IPR035093 (G3DSA:3.30.2310.GENE3D)	no GO terms	no GO terms
55	Uncharacterised protein (DNA polymerase III subunit)	256	No Domains	1	C:GO:0005575	C:cellular_component	_	_	mobidb-lite (MOBIDB_LITE); mobidb-lite (MOBIDB_LITE); CYTOPLASMIC_DOMAIN (PHOBIUS); NON_CYTOPLASMIC_DOMAIN (PHOBIUS); TRANSMEMBRANE (PHOBIUS); TMhelix (TMHMM)	no GO terms	no GO terms

Table S4. Characteristics of 12 most common (i.e. present in ≥ 5% of Pf prophages) accessory proteins; proteins being a part of putative (Cro/Cl family transcriptional regulator, GNAT family N-acetyltransferase and adenylate kinase) or well recognized (Phd/YefM family antitoxin and RelE/ParE family; toxin-antitoxin operon) operons are highlighted in grey.

# of Pf prophages	Pf prophage Group	BLAST Hit	Length [aa]	Conserved Domain	#GO	GO Names	Other features
11	Pf-tRNA-Gly	uncharacterised protein	171	-			
11	Pf-tRNA-Met	Cro/Cl family transcriptional regulator	264	COG4933-COG4933 PRK12279-PRK12279 HTH-31-pfam13560 HTH-XRE-smart00530 HTH-XRE-cd00093 couple-hipB-TIGR03070 ASCH-smart01022 COG3620-COG3620 PRK09726-PRK09726 ASCH-ASC-1-like-cd06554	7	P: response to hypoxia; P: regulation of fatty acid metabolic process; P: negative regulation of transcription, DNA-templated; P: negative regulation of growth; P: positive regulation of growth; F :DNA binding	-
11	Pf-tRNA-Met	AAA family ATPase	175	AAA-17-pfam13207 AdkA-COG2019 PRK04040-PRK04040	-	-	(Adenylate kinase (EC:2.7.4.3))
10	Pf-tRNA-Met	GNAT family N-acetyltransferase	348	PIN-VapC-like-cd18699	1	F: transferase activity, transferring acyl groups	Arylamine N-acetyltransferase (EC:2.3.1.5)
14	Pf-tRNA-Gly	uncharacterised protein	74	-	-	-	-
12	Pf-tRNA-Met	hypothetical protein	125	-	-	-	-
16	Pf-tRNA-Met	uncharacterised protein	145	-	1	C:cellular-component	TMhelix
38	Pf-tRNA-Met	hypothetical protein	111	-	-	-	Signal peptde
41	Pf-tRNA-Met	hypothetical protein	136	-	1	C:cellular-component	TMhelix
48	Pf-tRNA-Gly	type II toxin-antitoxin system Phd/YefM family antitoxin	83	PhdYeFM-antitox-pfam02604	-	-	-
49	Pf-tRNA-Gly	type II toxin-antitoxin system RelE/ParE family toxin	115	ParE-COG3668 ParE-toxin-pfam05016	-	-	-
55	Pf-tRNA-Gly (n=49) Pf-tmRNA (n=5) Pf-tRNA-Sec (n=1)	uncharacterized protein	256	-	1	C:cellular-component	TMhelix

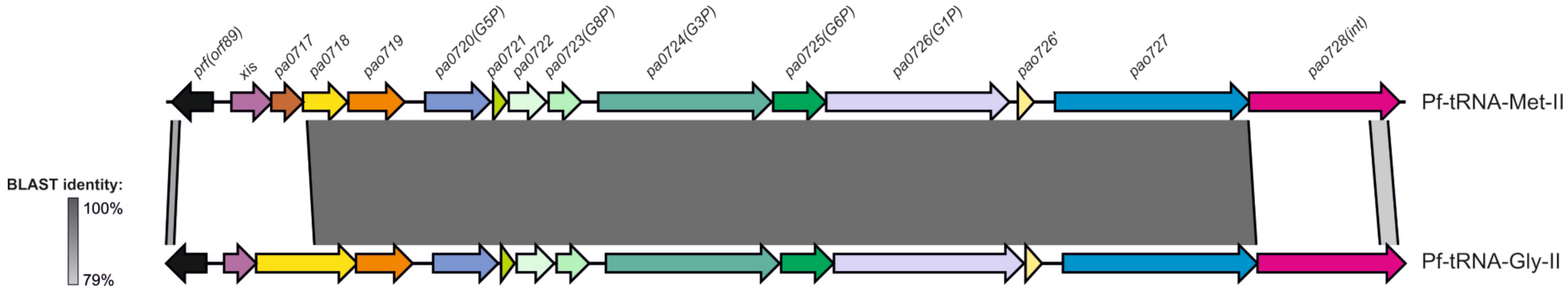

Fig. S1. Comparison of two prophages from the lineage II (Pf-tRNA-Met-II and Pf-tRNA-Gly-II) present in P. aeruginosa YL84 (CP007147).

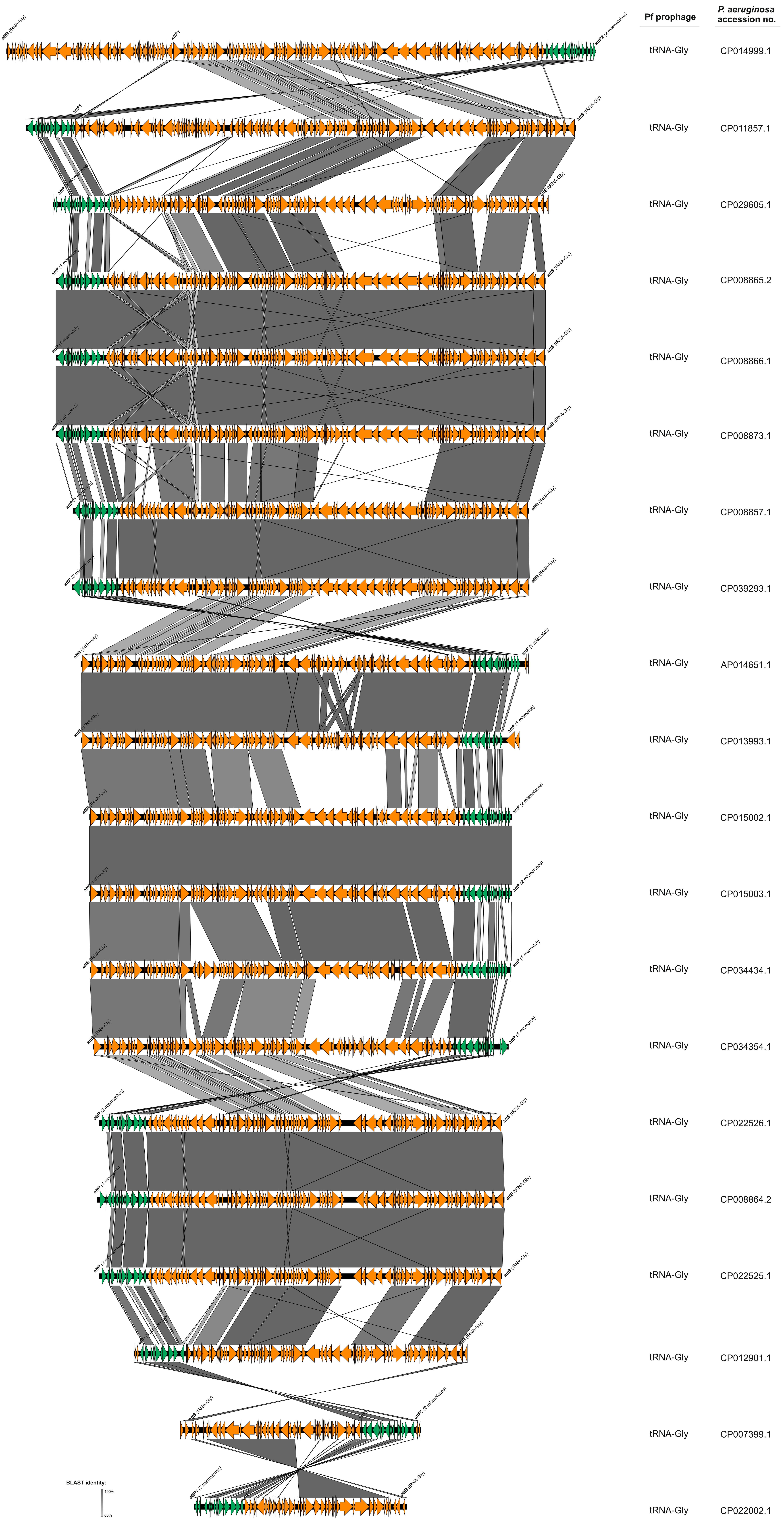

Fig. S2. Pf prophages with duplicated and/or atypical location of *attP* sites.

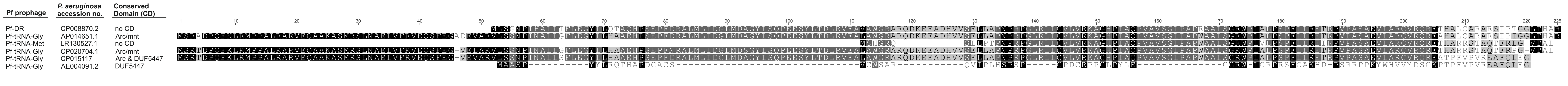

Fig. S3. Alignment of PA0718 variants present in the Pf prophages.

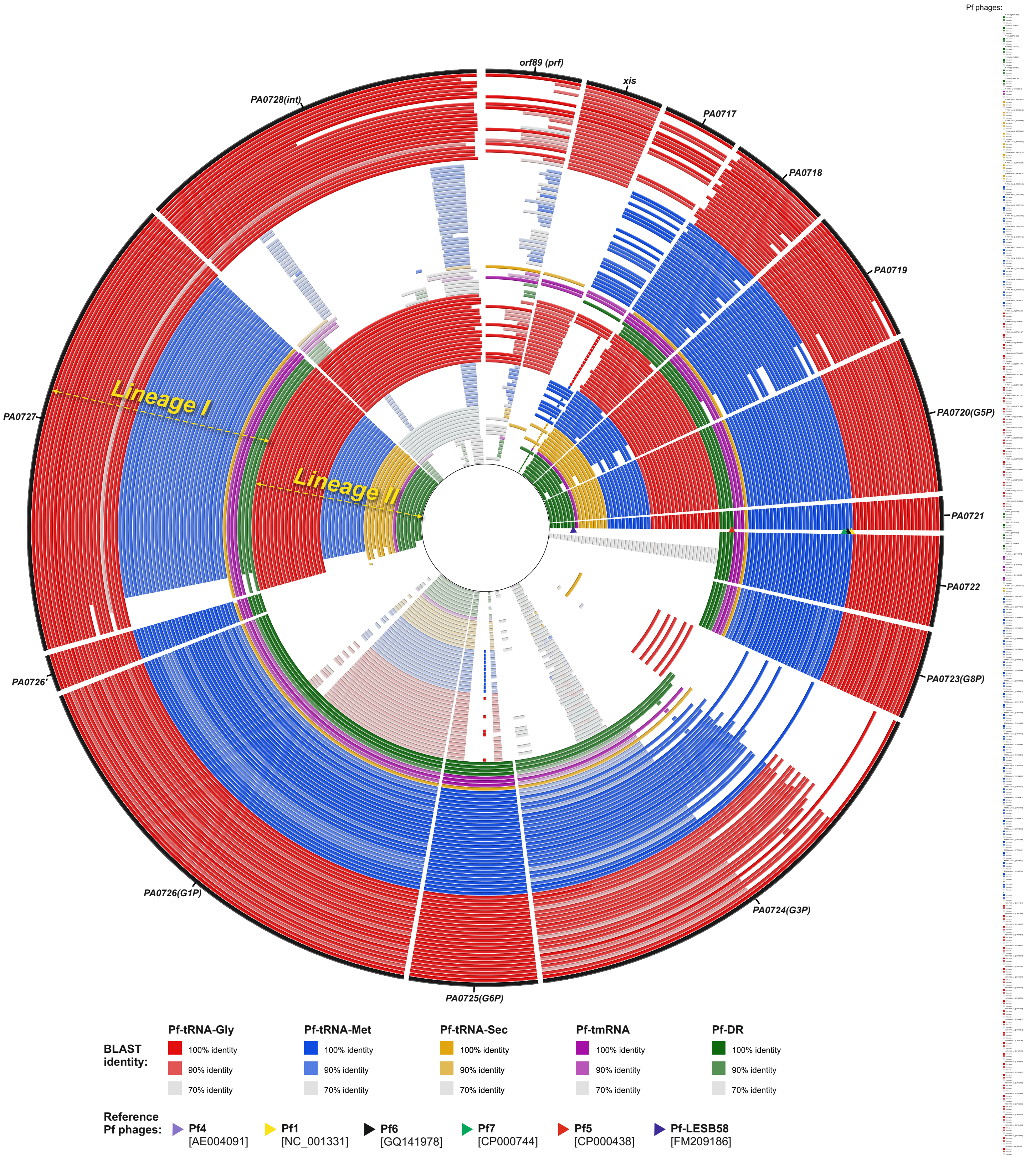

Fig. S4. Comparison of core gene similarity of the Pf (pro)phages (n=108) from the lineage I (outer rings) and the lineage II (inner ring). Genes from the Pf4 prophage (the outermost black circle) were used as references. The red, blue, orange, purple and green circles represent Pf-tRNA-Gly, Pf-tRNA-Met, Pf-tRNA-Sec, Pf-tmRNA and Pf-DR prophages, respectively. The comparison was performed using BRIG software with blastn algorithm (evalue 0.1, word size 7 options). Identical Pf prophages, occupying the same branch of the tree presented in Fig. 4, were excluded for the clarity. The position of reference Pf prophages: Pf1, Pf6, Pf5, Pf7 and Pf-LESB58, are indicated by coloured triangles (yellow, black, green, red and dark blue) at the *PA0721* gene position.

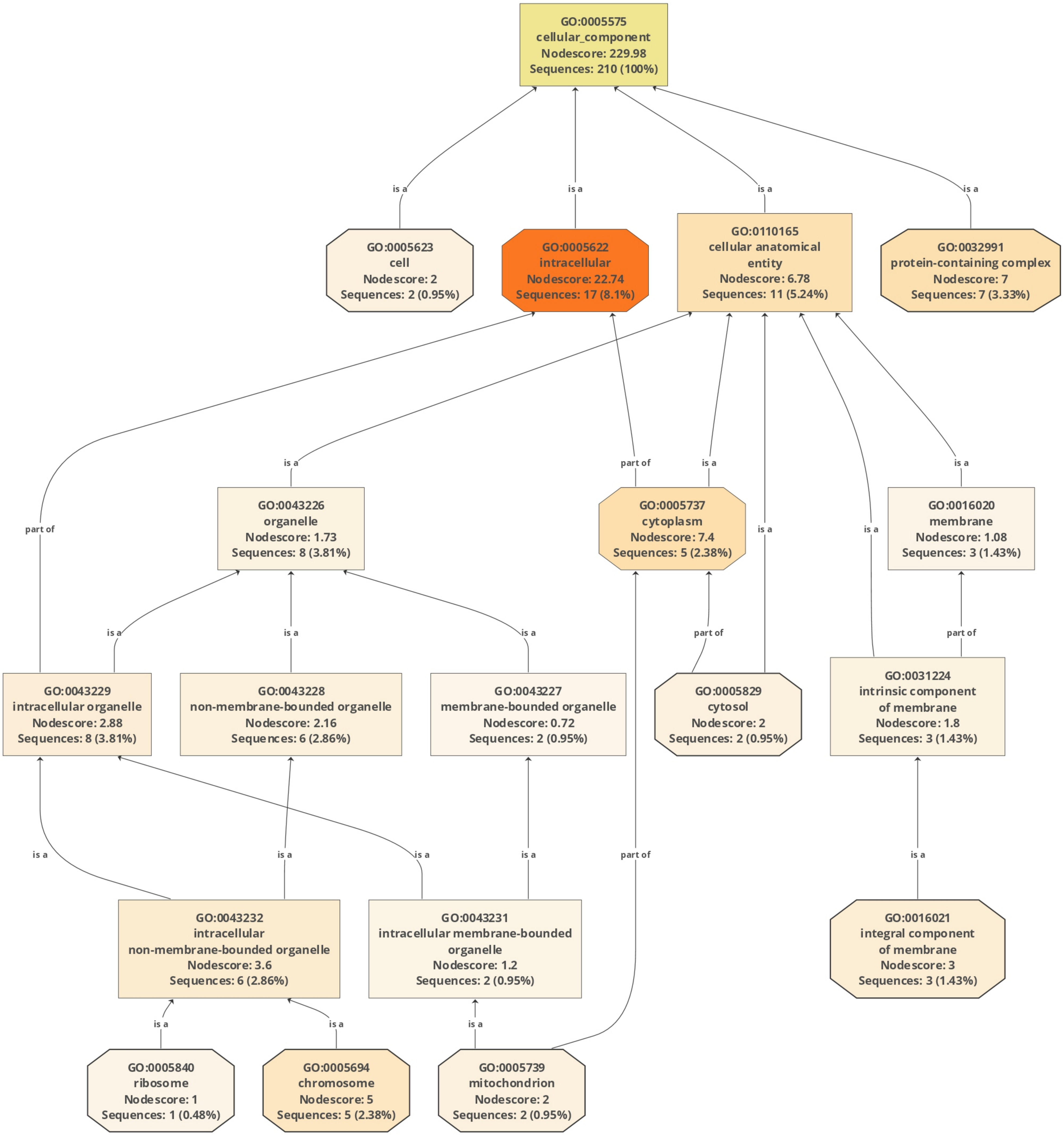

Fig. S5a. Accessory proteins of the Pf prophages classified into Cellular Component (CC) category (n=210).

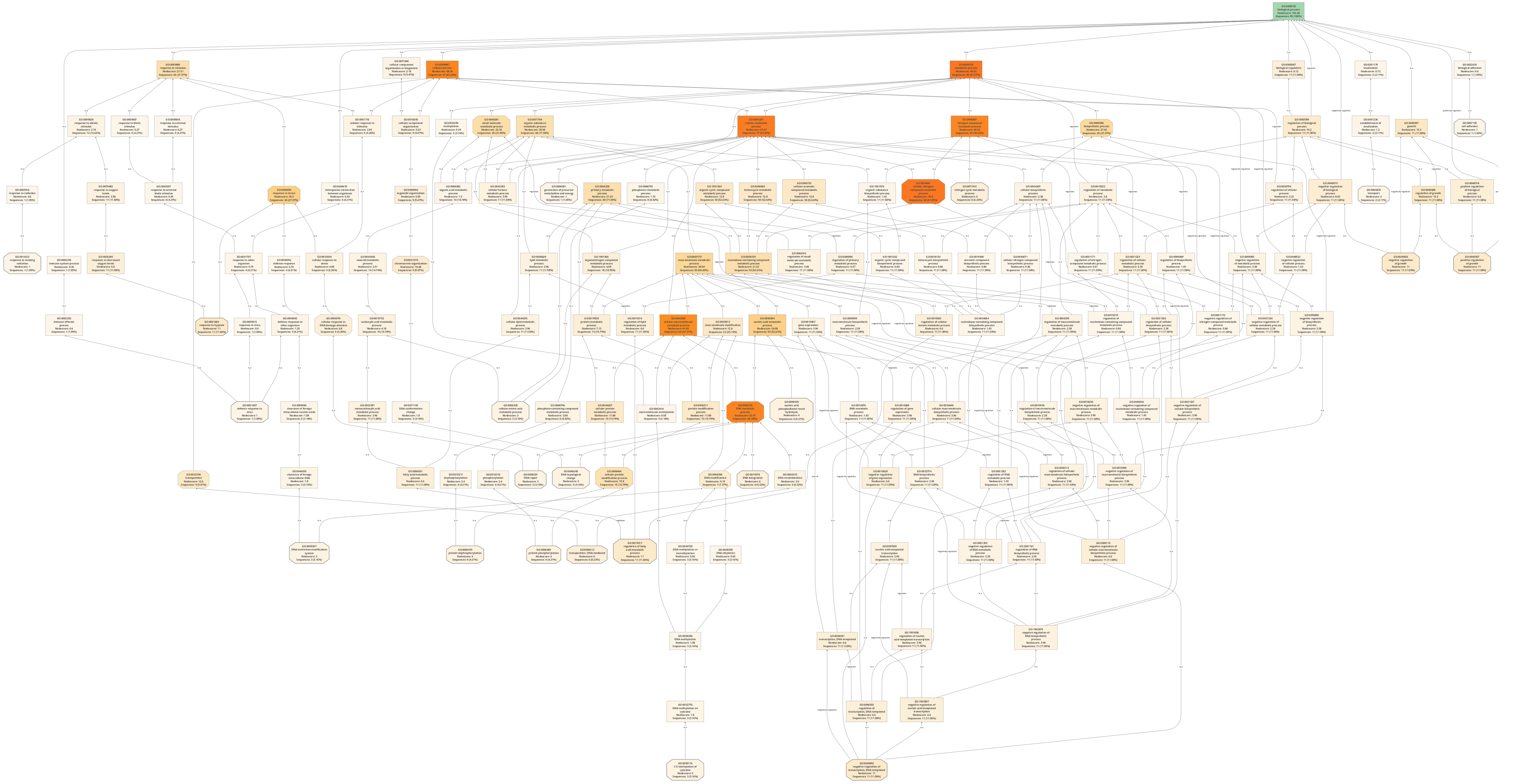

Fig. S5b. Accessory proteins of the Pf prophages classified into Biological Function (BF) category (n=95).

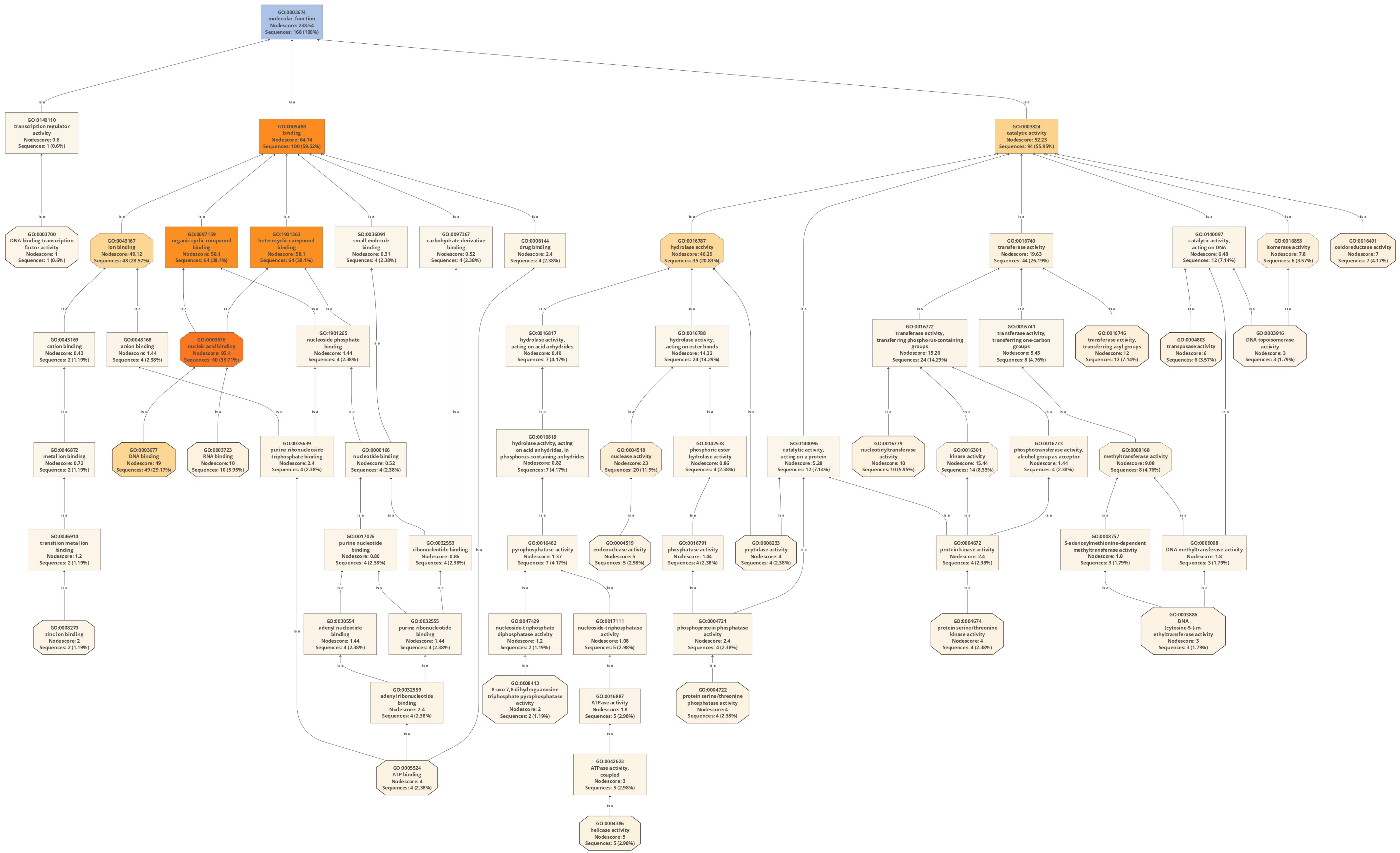

Fig. S5c. Accessory proteins of the Pf prophages classified into Molecular Function (MF) category (n=168).

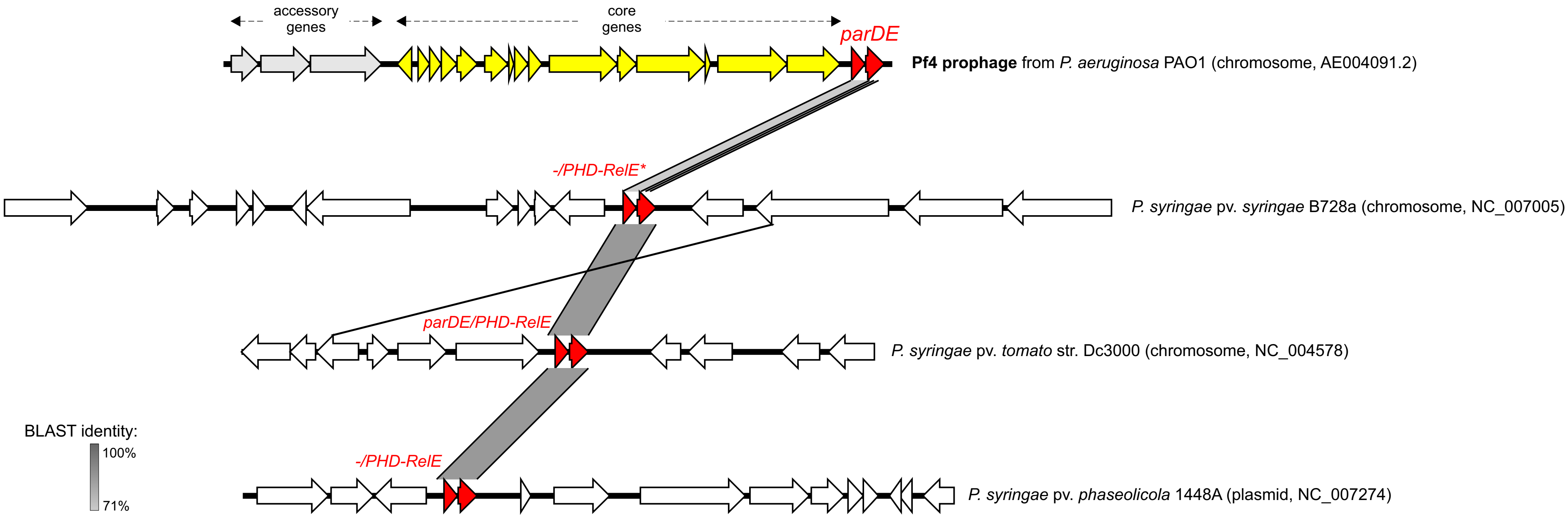

*, according to TADB 2.0 (https://bioinfo-mml.sjtu.edu.cn/TADB2/)

Fig. S6. *ParDE* toxin-antitoxin operon and its distribution in other *Pseudomonas spp.*

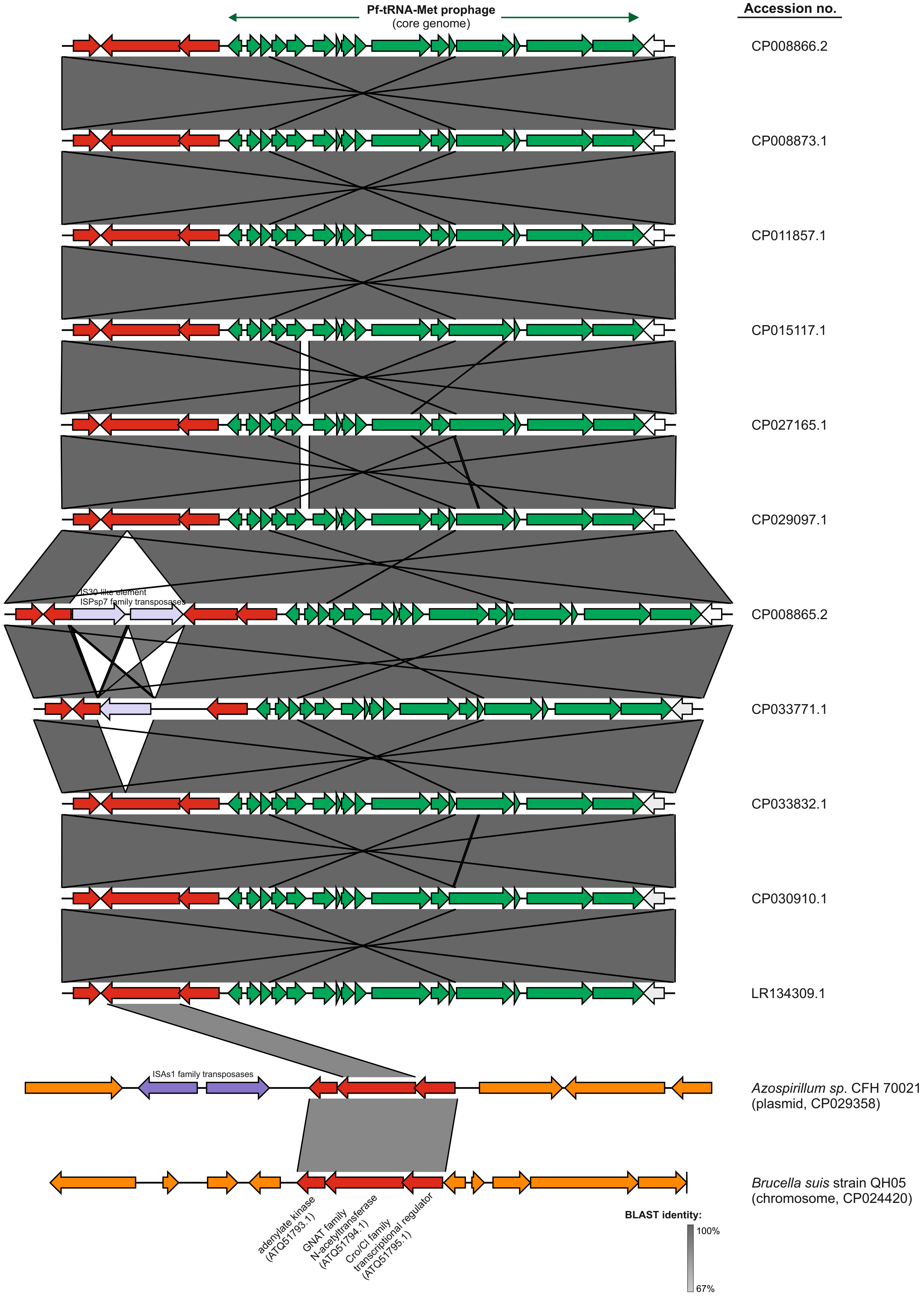

Fig. S7. Operon composed of a putative Cro/CI family transcriptional regulator, GNAT family N-acetyltransferase and adenylate kinase present in certain Pf-tRNA-Met prophages, as well as its possible origin.

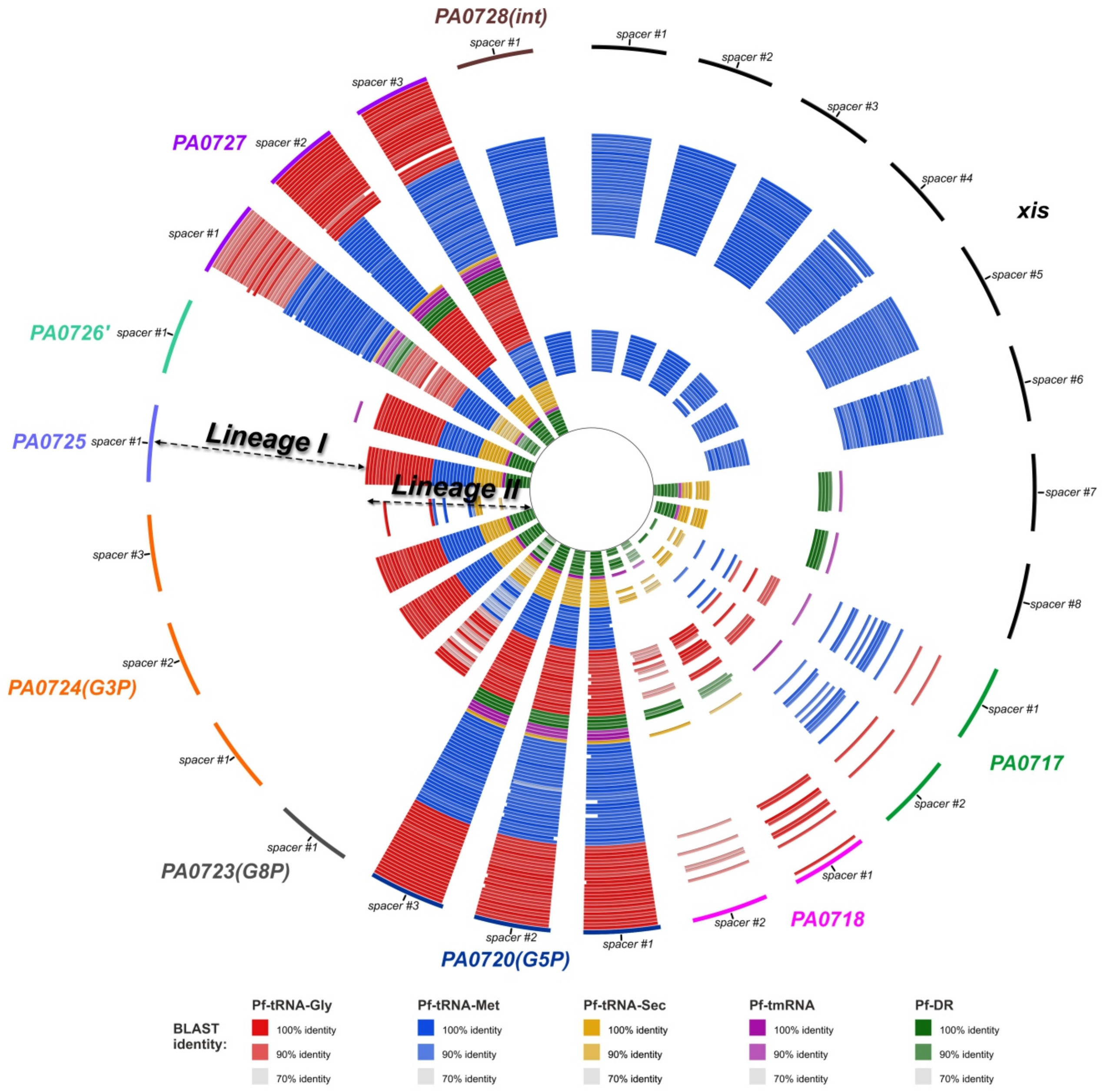

Target gene	Spacer #	Spacer sequence	CRISPR-Cas type	*P. aeruginosa* carrying CRISPR-Cas
xis	1	CGGCGACGGCCAATCTTGGCGGTCGGGATCGT	I-E	CP008858.2, CP006245.1
xis	2	ACCCAGCCCCGCACCACGTCTTCCGTGATGTC	I-F	LR134308.1
xis	3	GCCCCGCACCACGTCTTCCGTGATGTCTTCCA	I-F	LR134308.1
xis	4	AAGAGTCTGGAATAGTGGGGTTCACCGTTACA	I-F	LR134308.1
xis	5	TGCAGGTTCACCACGCGGCGACGACCAATCTT	I-F	LR134308.1
xis	6	GCCCCGGTCGAGGTCACGGCGAATACGGTGCA	I-F	LR134308.1
xis	7	CCATCAACTGATGGAAAAGGAGGAGTTCTGAT	I-F	LR134308.1
xis	8	ACGGACGGTTTCGTGGTCTTCCCCCATGCCAA	I-F	LR134308.1
PA0717	1	GTGCTGATGGAAACCTTCCAATTCTGCTTCGC	I-F	LR134308.1
PA0717	2	CTGCCAACGATGCCCGCGAAGCAGAATTGGAA	I-F	LR134308.1
PA0718	1	GCGTGCAGCAGATAGCCCTCAAGGAAGCTCAA	I-F	LR590473.1, CP039749.1
PA0718	2	TTCTTGGAAGGTTATCTGCTGCAAACGGCACA	I-F	LR134308.1
PA0719	1	CGCTGCTCCTGCTCTGCCTGATCCAGGCCGCC	I-E	CP008858.2, CP006245.1
PA0720 (G5P)	1	CCGACACCTACACCTCGACCAAGACCGGGGAA	I-F	CP008858.2, CP006245.1
PA0720 (G5P)	2	AACATCGAGCCGACCCTGCTGGACGCCATCGT	I-E	CP008858.2, CP006245.1
PA0723 (G8P)	1	TCAGTGCTGGCTTGCGCCAGAACCCCCTTGGT	I-F	LR134308.1
PA0724 (G3P)	1	CCGAAGCAAGGCTCCTTCGACAAGACCATCAA	I-F	LR134308.1
PA0724 (G3P)	2	TACGCCCAAGAACTCTCCGTCATCCGCTACGT	I-F	LR134308.1
PA0724 (G3P)	3	AATAAAACGTTTTGCCCGGATCGTTCTTCGAC	I-F	LR134308.1
PA0725 (G6P)	1	GCGAGAATGTCGGAGAGAAAGGGAATGTCCAT	I-F	LR134308.1
PA0726'	1	GCGAAGAAAAAGCCCGCCAGATAGGCCAGGAA	I-F	CP029745.1, CP029089.1, CP032257.1, CP027174.1
PA0727	1	CAGCCGTGCATATCGCGCTGGATGTGCAGGGC	I-E	CP006245.1, CP008858.2, LS483497.1
PA0727	2	TGGCTGATCAGGCTCCAGAACGGATCGTAGAC	I-F	CP029745.1, CP029089.1, CP032257.1, CP027174.1
PA0727	3	TGGAGCCTGATCAGCCAGGACGCCCGCGTCCA	I-F	CP029745.1, CP029089.1, CP032257.1, CP027174.1
PA0728 (int)	1	CAACGCTTGGCGCGCTGCGAGGCGCTGGCGAA	I-E	CP006245.1, CP008858.2

Fig. S8. Distribution of CRISPR-Cas spacer sequences in genes of the Pf (pro)phages (n=108) from the lineage I (outer rings) and the lineage II (inner rings). List of the spacer sequences, their affiliation to CRISPR-Cas type and P. aeruginosa strains carrying them are included in the table below. The red, blue, orange, purple and green circles represent Pf-tRNA-Gly, Pf-tRNA-Met, Pf-tRNA-Sec, Pf-tmRNA and Pf-DR prophages, respectively. The comparison was performed using BRIG software with blastn algorithm (evalue 0.1, word size 7 options). Identical Pf prophages, i.e. occupying the same branch of the tree presented in Fig. 4, were excluded for the clarity. The list of Pf prophages was included in supplementary figure S3, Supplementary Material online.

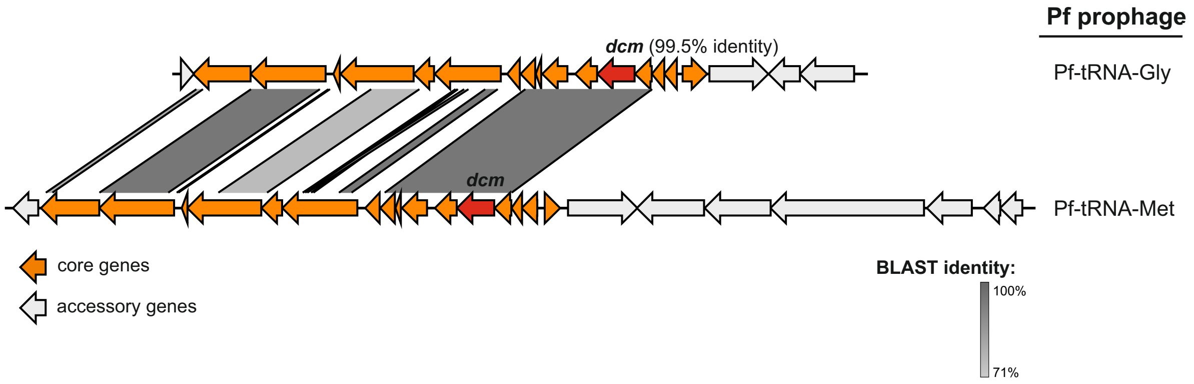

Fig. S9. Identity of *dcm* genes carried by Pf-tRNA-Gly and Pf-tRNA-Met prophages present in *P. aeruginosa* isolate PcyII-1 (LT673656.1).

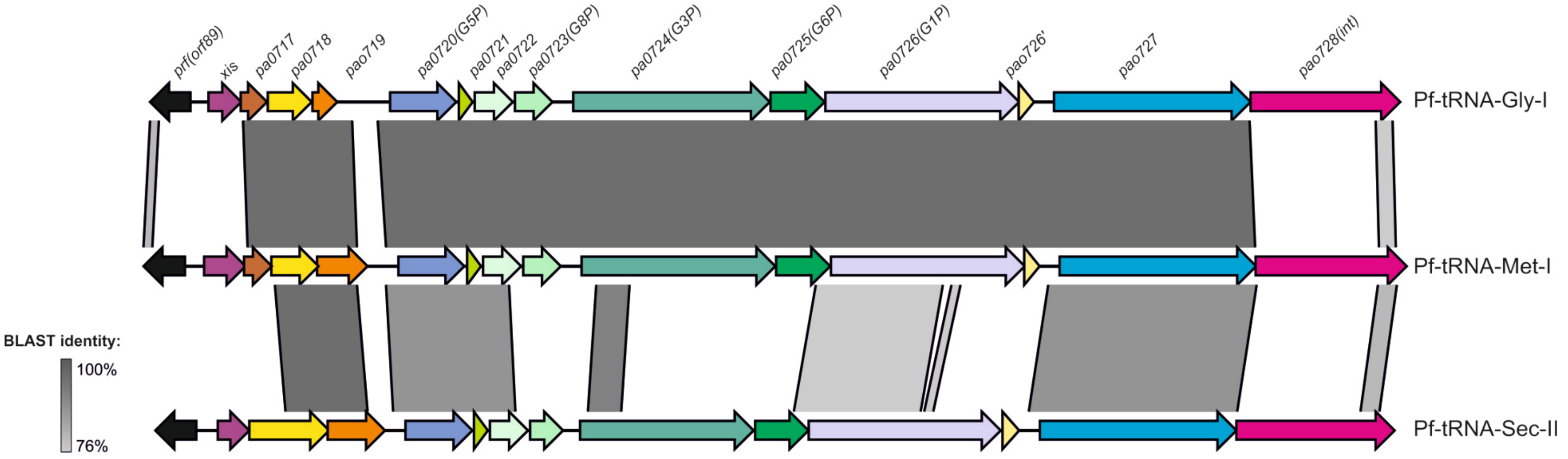

Fig. S10. Comparison of three prophages from the lineage I (Pf-tRNA-Met-I and Pf-tRNA-Gly-I) and lineage II (Pf-tRNA-Sec-II) present in *P. aeruginosa* strain X78812 (CP008872).

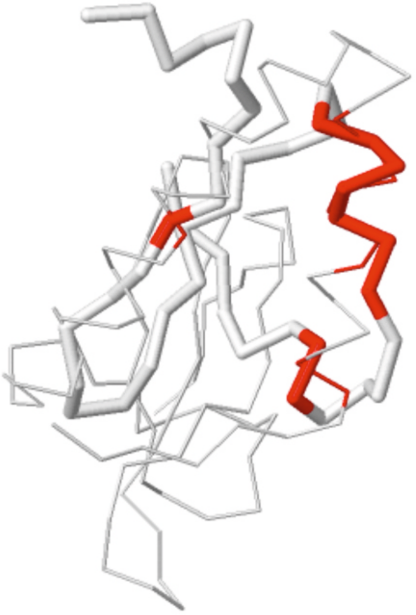

Fig. S11. Visualization of TM-score (a metric for measuring the similarity of two protein structures) superposition of *P. aeruginosa* Phd (white thick wirefames) and Phd/YefM family antitoxin from Pf prophages (white thin wirefames), red colour denotes the residue pairs of distance < 5.0 Angstrom (https://zhanglab.ccmb.med.umich.edu/TM-score/). 3D models of the proteins were predicted with SWISS-MODEL tool (https://swissmodel.expasy.org/).
